# Stepwise cycloaddition reaction of *N*-phenacylbenzothiazolium bromides and nitroalkenes for tetrahydro-, dihydro- and benzo[*d*]pyrrolo[2,1-*b*]thiazoles

**DOI:** 10.1038/srep46470

**Published:** 2017-04-13

**Authors:** Gong Jin, Jing Sun, Ren-Yin Yang, Chao-Guo Yan

**Affiliations:** 1 College of Chemistry & Chemical Engineering, Yangzhou University, Yangzhou 225002, China

## Abstract

The triethylamine promoted stepwise 1,3-dipolar cycloaddition reaction of *N*-phenacylbenzothiazolium bromides with nitroalkenes in ethanol resulted in a mixture of two isomeric tetrahydrobenzo[*d*]pyrrolo[2,1-*b*]thiazoles with *cis/trans/cis*- and *all-trans-*configurations. More importantly, the corresponding dihydrobenzo[*d*]pyrrolo[2,1-*b*]thiazoles can be selectively prepared in refluxing ethanol and the benzo[*d*]pyrrolo[2,1-*b*]thiazoles can be obtained in satisfactory yields by sequential dehydrogenation with DDQ as oxidizer. On the other hand, the similar cycloaddition reaction of *N*-phenacylbenzothiazolium bromides with 1-methy-1-nitroalkenes in refluxing ethanol afforded benzo[d]pyrrolo[2,1-b]thiazoles with splitting out of nitro group. The stereochemistry of the spiro compounds was clearly elucidated on the basis of NMR spectra and sixteen single crystal structures.

Pyrrolo[2,1-*b*]thiazole and its polyhydro- derivatives is one of the privileged N,S-containing heterocyclic motif that forms the core structure of many pharmacological reagents. It has been reported that pyrrolo[2,1-*b*]thiazole derivatives have been used as hepatoprotective, antidiabetic, antibiotic, anticonvulsant, antiinflammatory, bactericides and antitumor agents^1^,^2^,^3^,^4^,^5^,^6^. Therefore, many efficient synthetic methods have been exploited for the synthesis of diverse pyrrolo[2,1-*b*]thiazole and its bezno- derivatives^7^,^8^,^9^,^10^,^11^,^12^,^13^,^14^,^15^,^16^,^17^. In recent years, the development of new and efficient methods for their syntheses remains an attractive area for synthetic and medicinal chemists^18^,^19^,^20^,^21^,^22^,^23^,^24^,^25^,^26^,^27^,^28^. 1,3-Dipolar cycloaddition of azomethine ylides with various dienophiles is the one of most powerful methods for the construction of nitrogen-containing five-membered heterocycles. In this respect, besides most common using imidazolium ylides, pyridinium ylide and isoquinolinium ylides, the 1,3-dipolar cycloaddition of thiazolium ylides or benzothiazolium ylides were also employed for preparing some pyrrolo[2,1-*b*]thiazole and its bezno- derivatives^29^,^30^,^31^,^32^,^33^,^34^,^35^,^36^. Recently, we found that the 1,3-dipolar cycloadidtion reaction of base mediated *N*-phenacylbenzothiazolium bromides with 3-methyleneoxindoles afforded spiro[benzo[*d*]pyrrolo[2,1-b]thiazole-3,3′-indolines] in good yields and with high diastereoselectivity^37^. Similarly, the base promoted three-component reaction of *N*-phenacylbenzothiazolium bromides, aromatic aldehydes and indane-1,3-dione resulted in functionalized spiro[benzo[*d*]pyrrolo[2,1-*b*]thiazole-3,2′-indenes]^38^. In order to further provide efficient synthetic methodology for the biologically important benzo[*d*]pyrrolo[2,1-*b*]thiazoles and in continuation of our project on developing new multicomponent reactions based on the heteroaromatic *N*-ylides^39^,^40^,^41^,^42^,^43^,^44^, herein we wish to report stepwise 1,3-dipolar cycloaddition reaction of *N-*phenacylbenzothiazolium bromides with various nitroalkenes for selective synthesis of functionalized benzo[*d*]pyrrolo[2,1-*b*]thiazoles and their tetrahydro- and dihydro- derivatives.

## Results and Discussion

**Figure 1:**
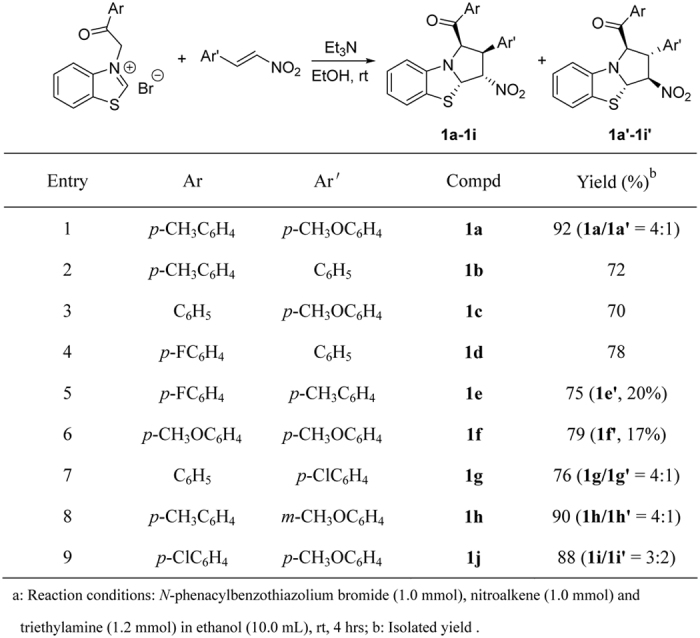
Synthesis of tetrahydrobenzo[*d*]pyrrolo[2,1-*b*]thiazoles^a^.

**Figure 2:**
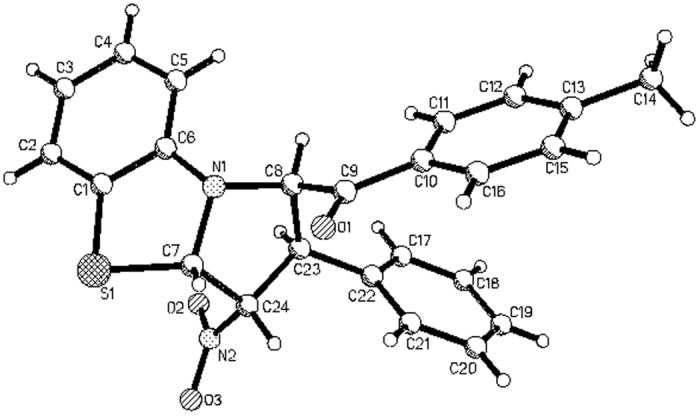
Molecular structure of 1b (major isomer).

**Figure 3:**
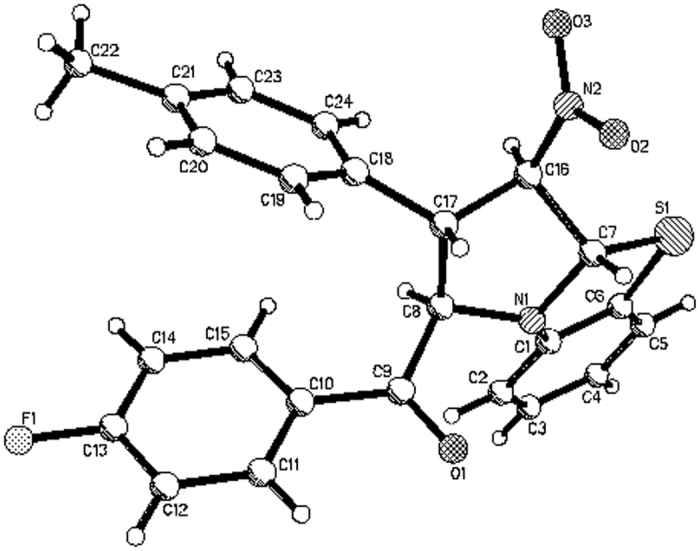
Molecular structure of 1e’ (minor isomer).

We began our investigation by carrying out the reaction of *N*-phenacylbenzothiazolium bromides with nitroalkenes according to our previously established base promoted procedure^37^,^38^. When a mixture of *N*-(4-methylphenacyl)benzothiazolium bromide with 1-nitro-2-(*p*-methoxyphenyl)ethene in ethanol with triethylamine as base was stirred at room temperature, the reaction can be finished in four hours to give the expected tetrahydrobenzo[*d*]pyrrolo[2,1-*b*]thiazole **1a** in very high yield. However, the spectroscopic analysis indicated that the product containing two stereoisomers **1a/1a’** with a molecular ratio of 4:1 (Fig. 1, entry 1). Because there are four chiral carbon atoms in the newly-formed pyrrolidine ring, several diastereoisomers might be formed in the 1,3-dipolar cycloaddition reaction. Attempting to increase the diastereoselectivity was not successfully after testing different reaction conditions including solvent and base. The reaction in DCM, THF and CH_3_CN afforded isomeric mixture **1a/1a’** still with ratio of 4:1 in 32%, 53% and 68% yields. By using piperidine, DABCO and DBU as base, similar results were obtained. Although the 1,3-dipolar cycloaddition of cyclic *N*-ylides with nitroalkenes have been reported in several articles^45^,^46^,^47^,^48^,^49^,^50^,^51^, the formation of isomeric five-membered heterocyclic system have not been investigated in detail. Thus, we turned our attention to elucidate the relative configurations of the obtained two diastereoisomers. The similar reactions of various *N*-phenacylbenzothiazolium bromides and nitroalkenes also resulted in a mixture of two diastereoisomers. In some cases, only major isomers **1b-1d** were successfully obtained, while the yields of minor isomers were too lower to separation (Fig. 1, entries 2–4). Both major isomers **1e**-**1f** and minor isomers **1e’-1f’** were successfully separated out by column chromatography (Fig. 1, entries 5–6). Additionally, a mixture of major isomer **1g-1h** and **1g’-1h’** were obtained and their ratios were calculated from the integration of ^1^H NMR spectra (Fig. 1, entries 7–9). The structures of all obtained products were fully characterized by IR, HRMS, ^1^H and ^13^C NMR spectroscopy. More importantly, the single crystal structures of major isomers **1a, 1b** (Fig. 2), **1c**, **1d**, **1f** and minor isomer of **1e**’ (Fig. 3) were successfully determined by X-ray diffraction method. From Fig. 2, it can be seen that the phenyl group and *p*-methylbenzoyl group exist on the same side of the newly-formed pyrrolidine ring. The nitro group and phenylsulfanyl group exist on the other side of the newly-formed pyrrolidine ring. Thus, the major isomer has *cis*/*trans*/*trans*-configuration. On the other hand, the four substituted groups exist in *all*-*trans*-configuration in the minor isomer of **1e**’ (Fig. 3). Thus, the relative configurations for the major and minor isomers of the polysubstituted tetrahydrobenzo[*d*]pyrrolo[2,1-*b*]thiazoles were ambiguously established by the determination of single crystal structures.

**Figure 4:**
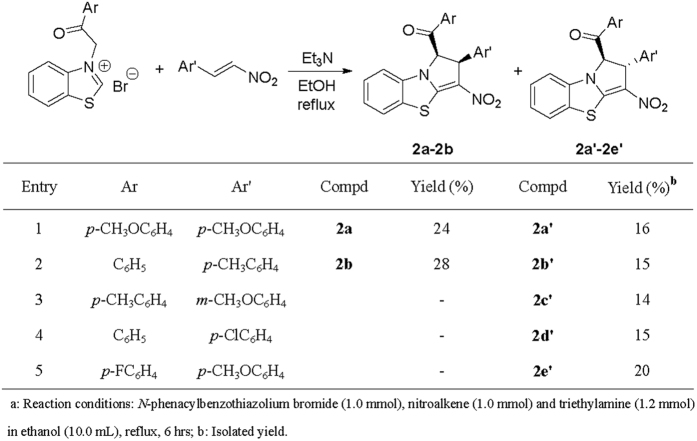
Synthesis of dihydrobenzo[*d*]pyrrolo[2,1-*b*]thiazoles^a^.

**Figure 5:**
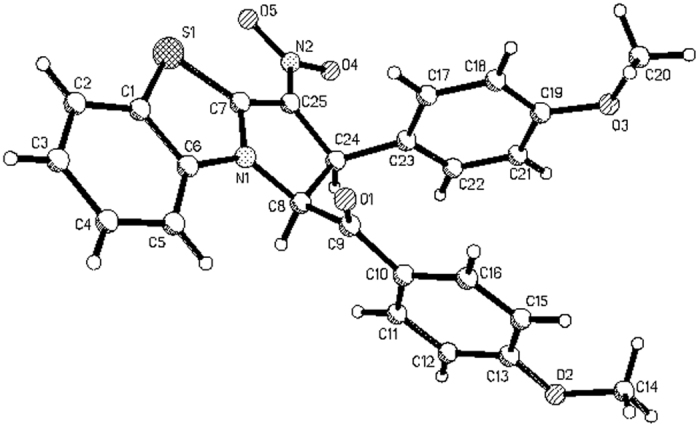
Molecular structure of 2a (major isomer).

**Figure 6:**
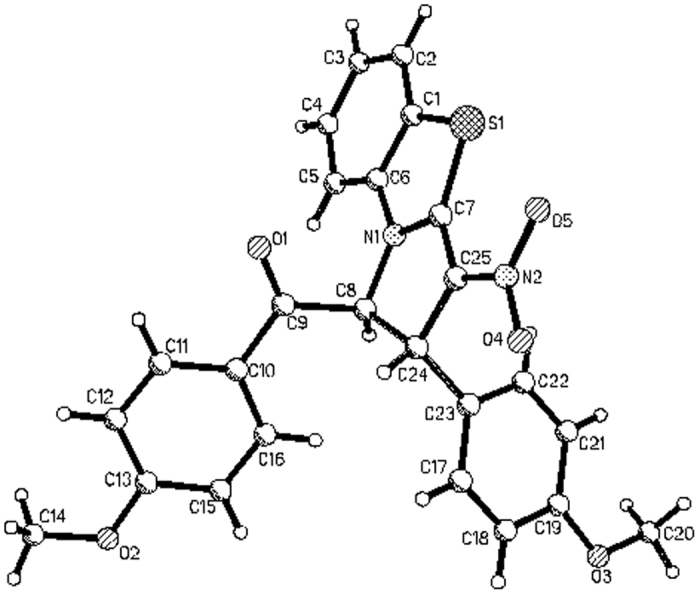
Molecular structure of 2a’ (major isomer).

When the above cycloaddition reaction of *N*-phenacylbenzothiazolium bromides and nitroalkenes were carried out in refluxing ethanol in the presence of triethylamine for about six hours. Besides formation of above mentioned tetrahydrobenzo[*d*]pyrrolo[2,1-*b*]thiazoles, some partially dehydrogenated dihydrobenzo[*d*]pyrrolo[2,1-*b*]thiazoles containing isomers **2a-2b** and isomers **2a’-2e’** were successfully separated out in lower yields from the reaction mixture (Fig. 4). It is interesting to find that partial dehydrogenation only occurred at 3,4-positions of the pyrrolidine ring, while the two hydrogen atoms at 1,2-position of pyrrolidine ring cannot be eliminated by prolonging refluxing time. The isomers **2a-2b** and isomers **2a’-2e’** could be clearly assigned by comparing of their ^1^H NMR spectra. As for an example, ^1^H NMR spectrum of isomer **2b** displays two doublets at 6.54, and 5.50 ppm with *J* = 10.7 Hz for the two neighboring protons in the newly-formed dihydropyrrole ring. The two protons in isomer **2b’** showed two slightly broad singlets at 6.79 and 4.70 ppm. The single crystal structures of the isomer **2a** (Fig. 5) and isomer **2a’** (Fig. 6), **2b’** were determined by X-ray diffraction method. The single crystal structures clearly showed that the major isomer has *cis*-configuration and minor isomer has *trans-*configuration. It should be pointed out that the major isomer **2** were clearly coming from the dehydrogenation of the major isomer **1**. The minor isomer **2’** also has same relative configuration to that of minor isomer **1’**.

**Figure 7:**
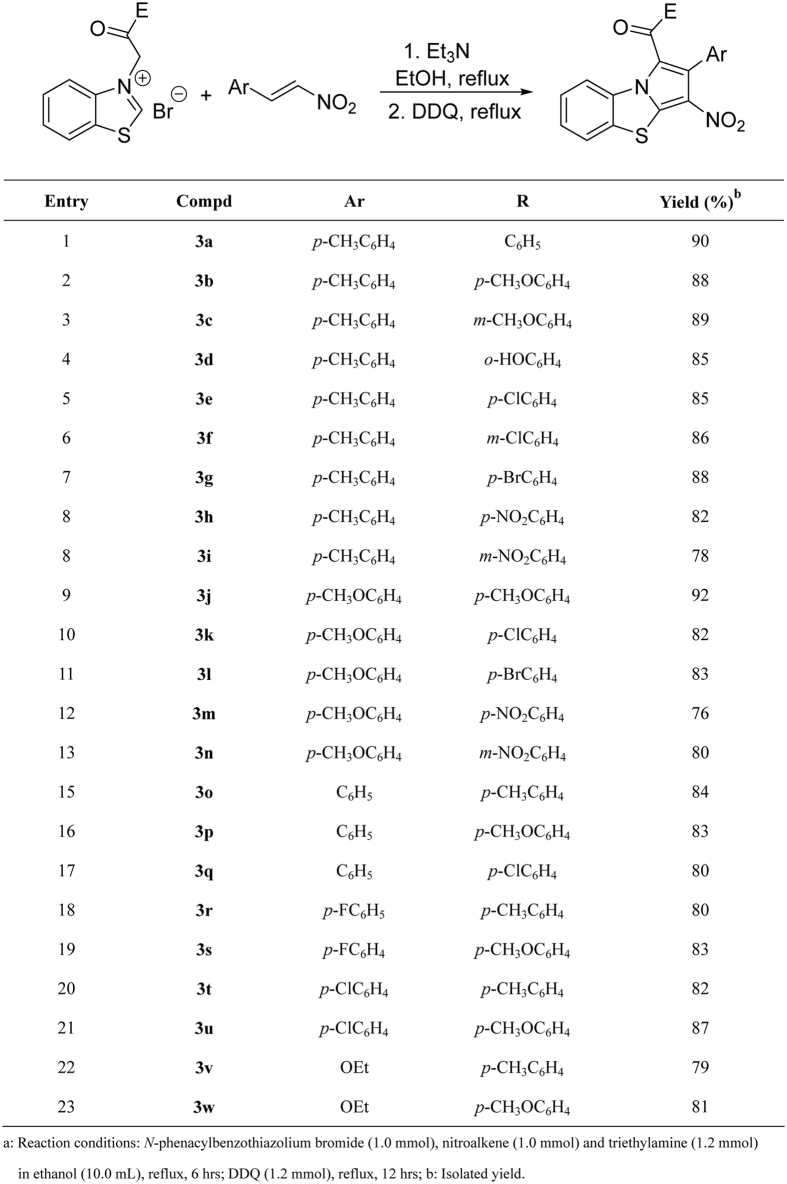
Synthesis of benzo[d]pyrrolo[2,1-b]thiazoles^a^.

**Figure 8:**
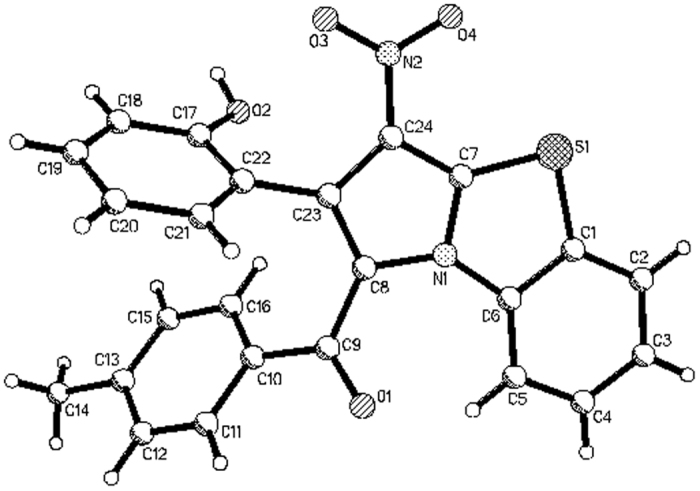
Single crystal structure of molecular 3d.

The above results clearly indicated that the reaction of *N*-phenacylbenzothiazolium bromides with nitroalkenes resulted in a mixture of isomeric tetrahydro- and dihydrobenzo[*d*]pyrrolo[2,1-*b*]thiazoles with various molecular ratios, which caused great difficulty for us to isolation and characterization of the obtained isomers. In order to avoid the influence of the complicate diastereoisomers, the aromatization process for above products was studied. Thus, after finishing the cycloaddition reaction, the oxidizing reagent DDQ was introduced in the reaction and the reaction mixture was refluxed for twelve hours. After workup, the expected aromatized benzo[*d*]pyrrolo[2,1-*b*]thiazoles **3a-3u** were successfully obtained in satisfactory yields (Fig. 7). Because there is only one possible isomer for the products **3a-3u**, the characterization for them was much easily finished. Various *N*-phenacylbenzothiazolium bromides and nitroalkenes with different substituents reacted smoothly to give the desired products. The substituents on the both substrates showed marginal effect on the yields of the products. Additionally, *N*-ethoxycarbonylmethyllbenzothiazolium salts was also employed in the reaction to give the substituted benzo[d]pyrrolo[2,1-b]thiazolecarboxylates (**3v-3w**) in good yields. The structures of the obtained products were fully established on the spectroscopic methods. Five single crystal structures of the compounds **3d** (Fig. 8), **3e**, **3j**, **3p**, and **3w** were also determined by X-ray diffraction method.

**Figure 9:**
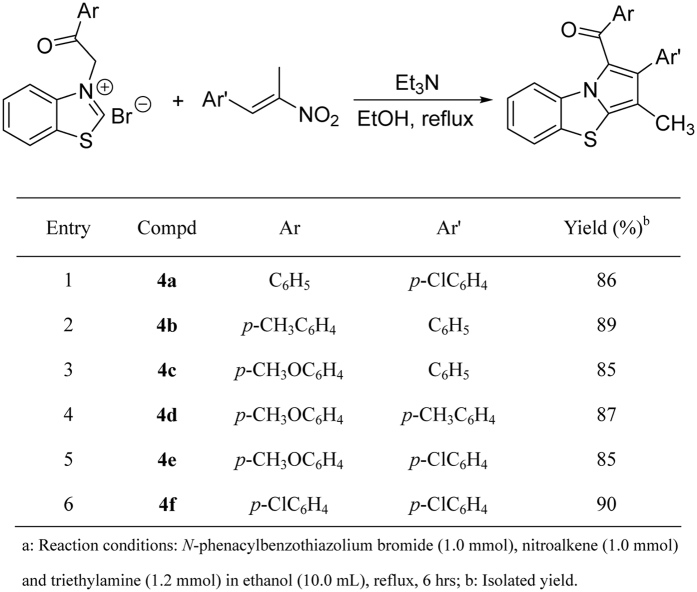
Synthesis of methylbenzo[*d*]pyrrolo[2,1-*b*]thiazoles^a^.

**Figure 10:**
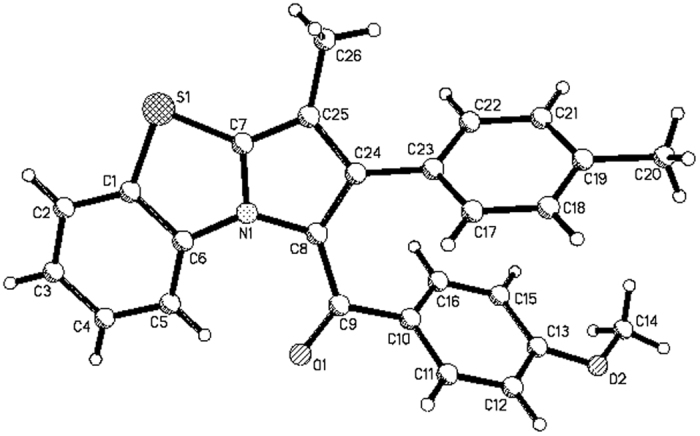
Single crystal structure of molecular 4d.

In order to develop the synthetic value of the reaction, 1-methyl-1-nitroalkenes derived from the condensation reaction of aromatic aldehydes and nitroethane were also employed in the reaction. In the presence of triethylamine, a mixture of *N*-phenacylbenzothiazolium bromide and 1-methyl-1-nitroalkene was refluxed for about six hours, the methyl-substituted benzo[d]pyrrolo[2,1-b]thiazoles **4a-4f** were successfully prepared in high yields (Fig. 9). The spectroscopic analysis indicated the nitro group was splitted off in the aromatization process, which is different to that of the above prepared compounds **3a-3w**, in which the nitro group is retained in the final product. The structures of the benzo[*d*]pyrrolo[2,1-*b*]thiazoles **4a-4f** were unambiguously determined by the single crystal structures of the compounds **4d** (Fig. 10) and **4e**.

**Figure 11:**
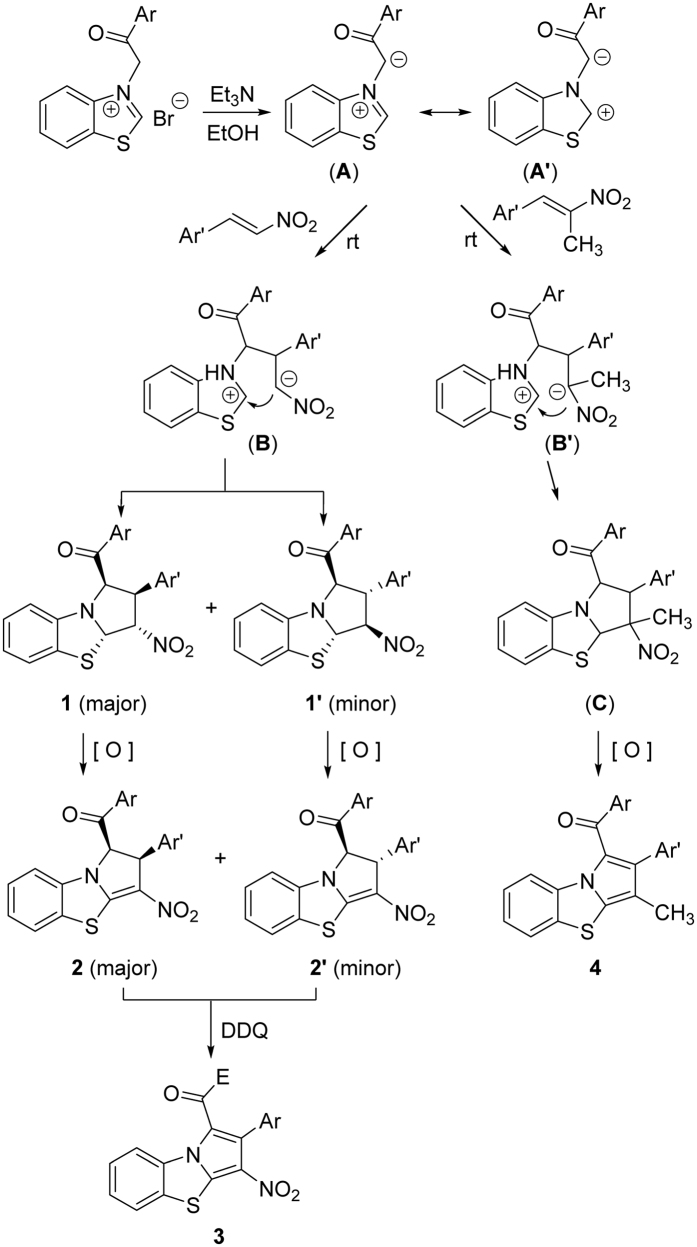
Proposed reaction mechanism for stepwise cycloaddition reaction.

For explaining the formation mechanism of the obtained various benzo[*d*]pyrrolo[2,1-*b*]thiazole derivatives, a plausible reaction mechanism is proposed on the basis of the present results and the related reactions (Fig. 11). At first, *N*-phenacylbenzothiazolium bromide was deprotonated by triethylamine to give a benzothiazolium ylide (**A**), which has a resonance form (**A’**). Secondly, the nucleophilic addition of the ylide (**A**) to nitroalkene gave a intermediate (**B**). Then, the intramolecular cyclization of intermediate (**B**) resulted in two isomeric tetrahydrobenzo[*d*]pyrrolo[2,1-*b*]thiazoles **1** and **1’**. Because this process gave less thermodynamic stable *cis*/*trans*/*trans*-isomer as major isomer and more stable *all*-*trans*-isomer as minor isomer, it is a dynamic control reaction. The partial dehydrogenation of two isomeric products **1** and **1’** by oxidation of air produces corresponding dihydrobenzo[*d*]pyrrolo[2,1-*b*]thiazoles **2** and **2’**, respectively. Finally, oxidation with DDQ resulted in the benzo[*d*]pyrrolo[2,1-*b*]thiazoles **3**. The cycloaddition reaction with 1-methyl-1-alkene preceded with a similar path, the initially formed intermediate (**C**) was much easily oxidized in air to give the 3-methylbenzo[*d*]pyrrolo[2,1-*b*]thiazole **4** by eliminating nitro group.

## Conclusion

In summary, we have systemically investigated stepwise1,3-dipolar cycloaddition reaction of *N*-phenacylbenzothiazolium bromides with nitroalkenes and successfully provided a convenient protocol for selective syntheses of functionalized benzo[*d*]pyrrolo[2,1-*b*]thiazole and its tetrahydro-, dihydro- derivatives. The relative configuration of tetrahydro-, and dihydro benzo[*d*]pyrrolo[2,1-*b*]thiazoles were clearly elucidated on the basis of NMR spectra and single crystal structures. The advantages of the reaction included using readily avaiable starting material, milder reaction condition, good yields and a widely variety of substrates. This reaction might be found potentail applications for the synthesis of the *N*,*S*-containing heterocycles in synthetic and medicinal chemistry.

## Methods

### Materials

All reactions were performed in atmosphere unless noted. All reagents were commercially available and use as supplied without further purification. NMR spectra were collected on either an Agilent DD2400 MHz spectrometer or a Bruker AV-600 MHz spectrometer with internal standard tetramethylsilane (TMS) and signals as internal references, and the chemical shifts (δ) were expressed in ppm. High-resolution Mass (ESI) spectra were obtained with Bruker Micro-TOF spectrometer. The Fourier transform infrared (FTIR) samples were prepared as thin films on KBr plates, and spectra were recorded on a Bruker Tensor 27 spectrometer and are reported in terms of frequency of absorption (cm^−1^). X-ray data were collected on a Bruker Smart APEX-2 CCD diffractometer.

### General procedure for the synthesis of tetrahydrobenzo[*d*]pyrrolo[2,1-*b*]thiazoles 1a-1f

To a 50 mL round flask was added *N*-phenacylbenzothiazolium bromide (1.0 mmol), nitroalkene (1.0 mmol) and triethylamine (1.2 mmol) in ethanol (10.0 mL). The solution was stirred at room temperature for four hours. The solvent was removed by rotatory evaporation at reduced pressure. The residue was subjected to column with a mixture of light petroleum and ethyl acetate as eluent to give the pure products for analysis.

### General procedure for the synthesis of dihydrobenzo[*d*]pyrrolo[2,1-*b*]thiazoles 2a-2b

To a 50 mL round flask was added *N*-phenacylbenzothiazolium bromide (1.0 mmol), nitroalkene (1.0 mmol) and triethylamine (1.2 mmol) in ethanol (10.0 mL). The solution was heated to refluxing for six hours. The solvent was removed by rotatory evaporation at reduced pressure. The residue was subjected to column with a mixture of light petroleum and ethyl acetate (V/V = 3:1) as eluent to give the pure products for analysis.

### General procedure for the synthesis of benzo[*d*]pyrrolo[2,1-*b*]thiazoles 3a-3w

To a 50 mL round flask was added *N*-phenacylbenzothiazolium bromide (1.0 mmol), nitroalkene (1.0 mmol) and triethylamine (1.2 mmol) in ethanol (10.0 mL). The solution was heated to refluxing for six hours. Then, DDQ (1.2 mmol) was added and the mixture was refluxed for twelve hours. The solvent was removed by rotatory evaporation at reduced pressure. The residue was subjected to column with a mixture of light petroleum and ethyl acetate (V/V = 3:1) as eluent to give the pure products for analysis.

### General procedure for the synthesis of hydrobenzo[*d*]pyrrolo[2,1-*b*]thiazoles 4a-4f

To a 50 mL round flask was added *N*-phenacylbenzothiazolium bromide (1.0 mmol), 1-methy-1-nitroalkenes (1.0 mmol) and triethylamine (1.2 mmol) in ethanol (10.0 mL). The solution was heated to refluxing for six hours. The solvent was removed by rotatory evaporation at reduced pressure. The residue was subjected to column with a mixture of light petroleum and ethyl acetate (V/V = 3:1) as eluent to give the pure products for analysis.

## Additional Information

**Accession Codes**: Crystallographic data **1a** (CCDC 1507670); **1b** (CCDC 1507671); **1c** (CCDC 1507672); **1d** (CCDC1507673); **1e′** (CCDC 1507674); **1f** (CCDC 1507675); **2a** (CCDC 1507676); **2a′** (CCDC 1507677); **2b′** (CCDC 1507678); **3d** (CCDC 1507679); **3e** (CCDC 1507680); **3j** (CCDC 1507681); **3p** (CCDC 1507682); **3w** (CCDC 1507683), **4d** (CCDC 1507684) and **4e** (CCDC 1507685) have been deposited at the Cambridge Crystallographic Database Centre and is available on request (http//www.ccdc.cam.ac.uk).

**How to cite this article**: Jin, G. *et al*. Stepwise cycloaddition reaction of *N*-phenacylbenzothiazolium bromides and nitroalkenes for tetrahydro-, dihydro- and benzo[*d*]pyrrolo[2,1-*b*]thiazoles. *Sci. Rep.* **7**, 46470; doi: 10.1038/srep46470 (2017).

**Publisher's note:** Springer Nature remains neutral with regard to jurisdictional claims in published maps and institutional affiliations.

## Supplementary Material

Supplementary Information
