# Supplementary material for: Stepwise cycloaddition reaction of N-phenacylbenzothiazolium bromides and nitroalkenes for tetrahydro-, dihydro- and benzo[d]pyrrolo[2,1-b]thiazoles
Source: Sci Rep. 2017 Apr 13;7:46470. doi: 10.1038/srep46470 (PMC5390295; doi:10.1038/srep46470)
Supplement: Supplementary Information [file srep46470-s1.pdf]

Stepwise cycloaddition reaction of *N*-phenacylbenzothiazolium bromides and nitroalkenes for tetrahydro-, dihydro- and benzo[*d*]pyrrolo[2,1-*b*]thiazoles

Gong Jin, Jing Sun*, Ren-Yin Yang, Chao-Guo Yan*

Supporting Information

Figures of single crystal structures **2-5**

Characterization data and spectra of the compounds **6-99**

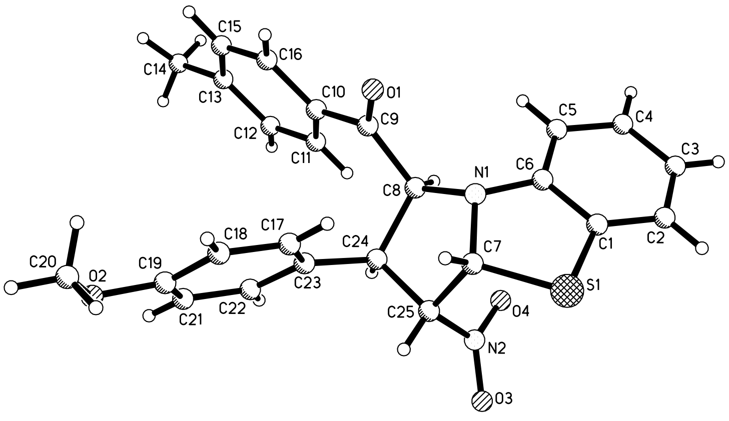

Fig. s1 Molecular structure of **3a** (major isomer)

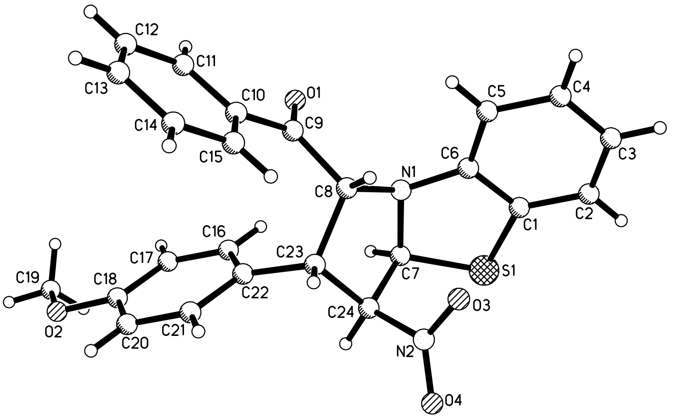

Fig. s2 Molecular structure of **3c** (major isomer)

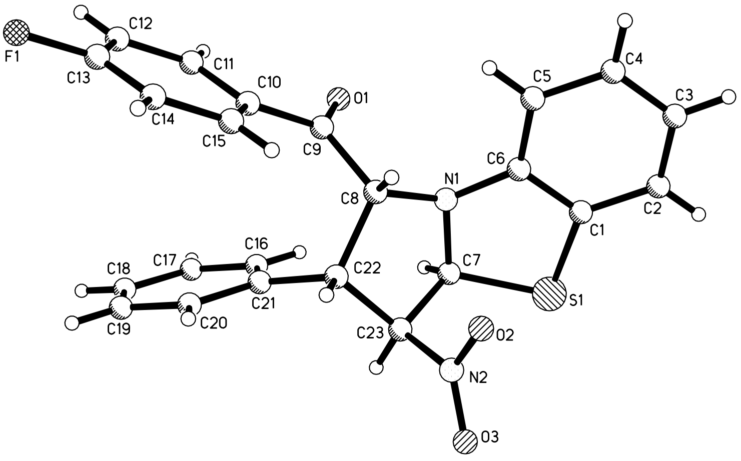

Fig. s3 Molecular structure of **3d** (major isomer)

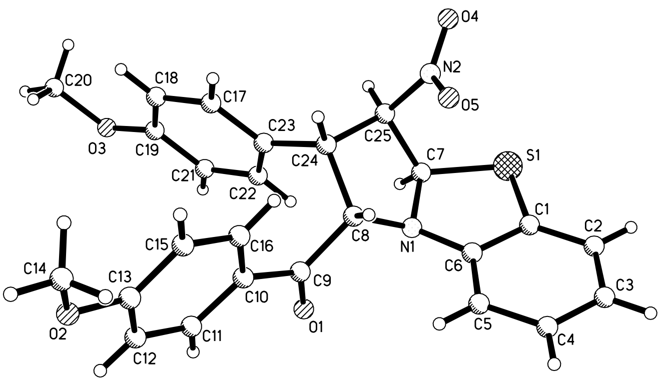

Fig. s4 Molecular structure of **3f** (major isomer)

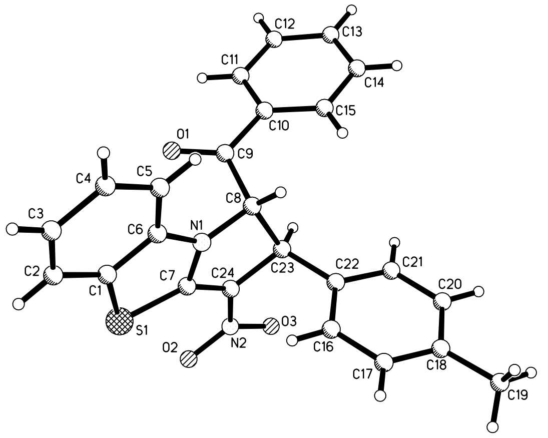

Fig. s5 Molecular structure of **4b'** (minor isomer)

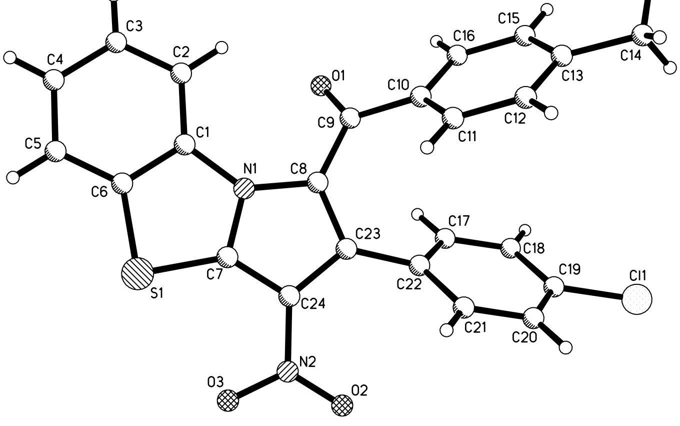

Fig. s6 Molecular structure of compound **5e**

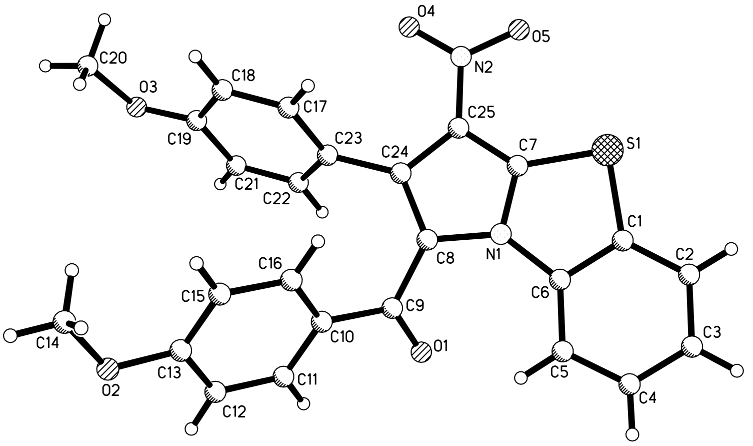

Fig. s7 Molecular structure of compound **5j**

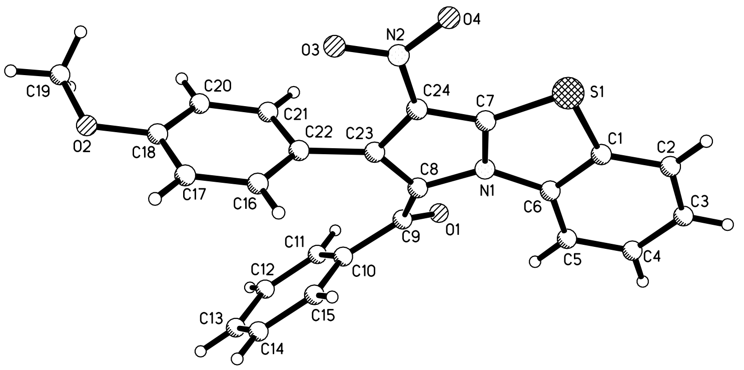

Fig. s8 Molecular structure of compound **5p**

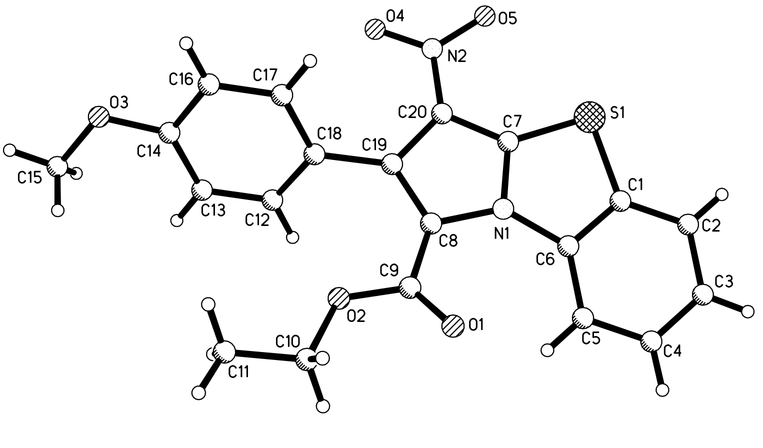

Fig. s9 Molecular structure of compound **5w**

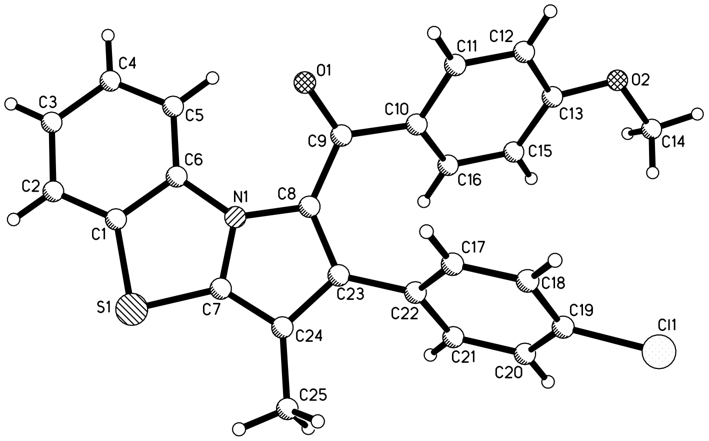

Fig. s10 Molecular structure of compound **6e**

(2-(4-methoxyphenyl)-3-nitro-1,2,3,3a-tetrahydrobenzo[d]pyrrolo[2,1-b]thiazol-1-yl)(p-tolyl) methanone (1a+1a'): yellow solid, 92%, m.p. 159~161℃; ^{1}H NMR (600 MHz, DMSO-d_6) δ: major isomer: 7.78 (d, J = 7.8 Hz, 2H, ArH), 7.24 (d, J = 7.8 Hz, 2H, ArH), 7.16 (d, J = 7.2 Hz, 1H, ArH), 7.13 (d, J = 8.4 Hz, 2H, ArH), 7.03~7.00 (m, 1H, ArH), 6.84~6.81 (m, 1H, ArH), 6.71~6.68 (m, 2H, ArH), 6.66~6.64 (m, 1H, ArH), 6.38~6.37 (m, 1H, CH), 5.98~5.97 (m, 1H, CH), 5.85~5.83 (m, 1H, CH), 4.70~4.65 (m, 1H, CH), 3.63 (s, 3H, OCH_3), 2.33 (s, 3H, CH_3); Minor isomer: δ: 7.72 (d, J = 7.8 Hz, 2H, ArH), 7.20 (d, J = 7.4 Hz, 1H, ArH), 6.96~6.94 (m, 3H, ArH), 6.87~6.86 (m, 1H, ArH), 6.33~6.32 (m, 1H, CH), 6.02~6.01 (m, 1H, CH), 5.66~5.65 (m, 1H, CH), 3.64 (s, 3H, OCH_3). Ratio of 3a/3a' = 4:1. ^{13}C NMR (100 MHz, DMSO-d_6) δ: 195.9, 158.2, 147.0, 144.0, 132.8, 129.4, 129.1, 128.2, 126.8, 126.1, 125.6, 121.5, 121.1, 113.6, 111.2, 94.3, 72.1, 71.7, 54.8, 49.8, 21.0; IR (KBr) υ: 3053, 2941, 1695, 1606, 1549, 1511, 1463, 1370, 1336, 1300, 1250, 1182, 1147, 1031, 989, 890, 831, 752 cm^{-1}; MS (m/z): HRMS (ESI) Calcd. for $C_{25}H_{23}N_2O_4S$ ([M+H]$^{+}$): 447.1373. Found: 447.1376.

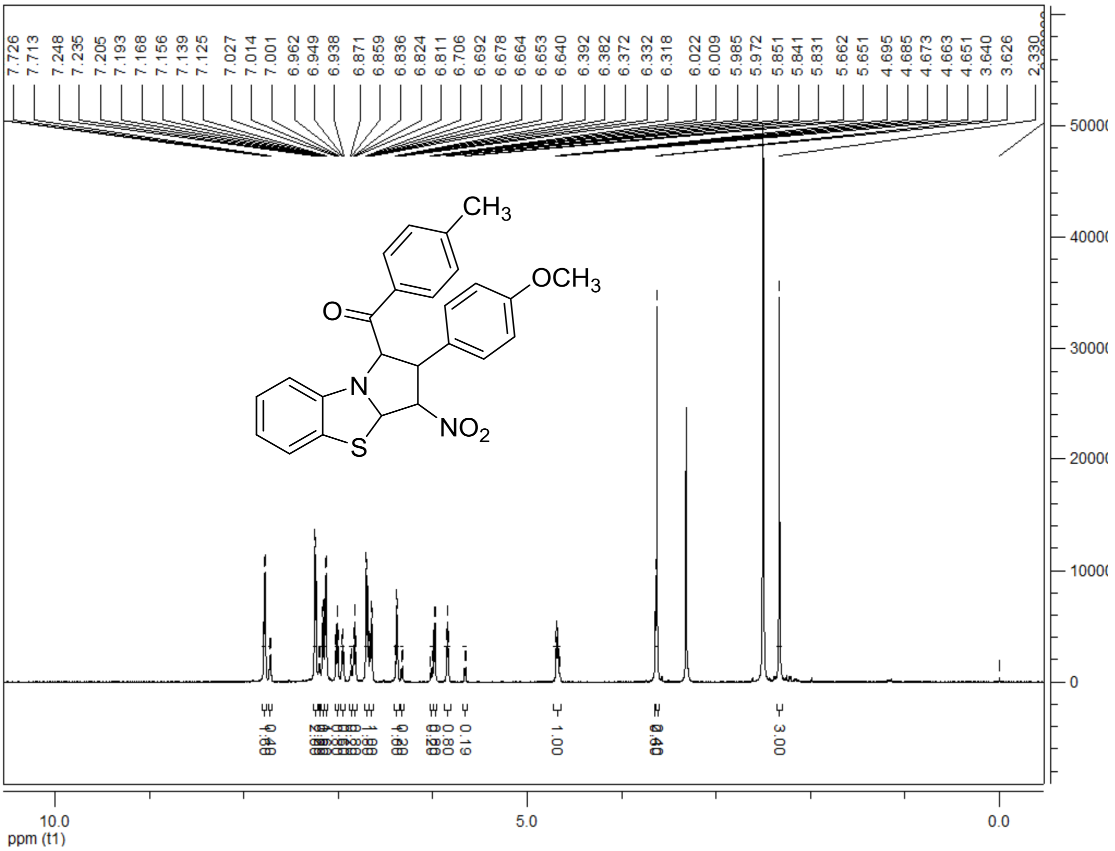

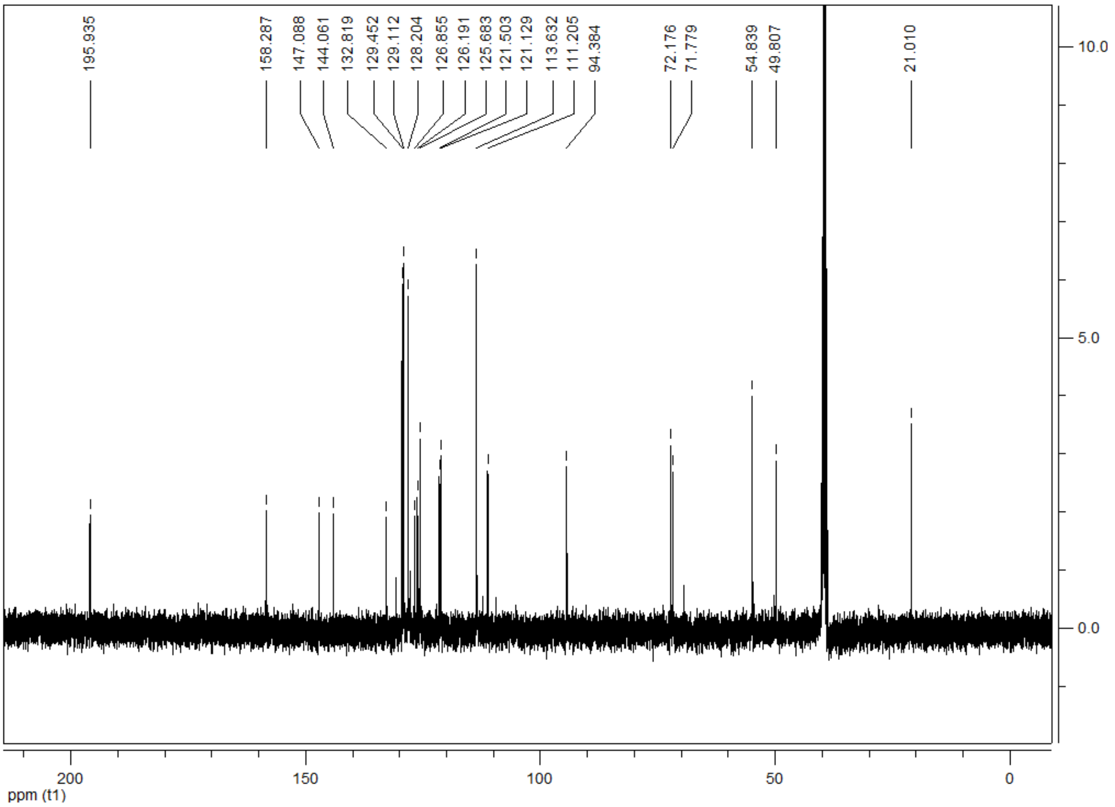

195.935
158.287
147.088
144.061
132.819
129.452
129.112
128.204
126.855
126.191
125.683
121.503
121.129
113.632
111.205
94.384
72.176
71.779
54.839
49.807
21.010
10.0
5.0
0.0
200
150
100
50
0
ppm (t1)

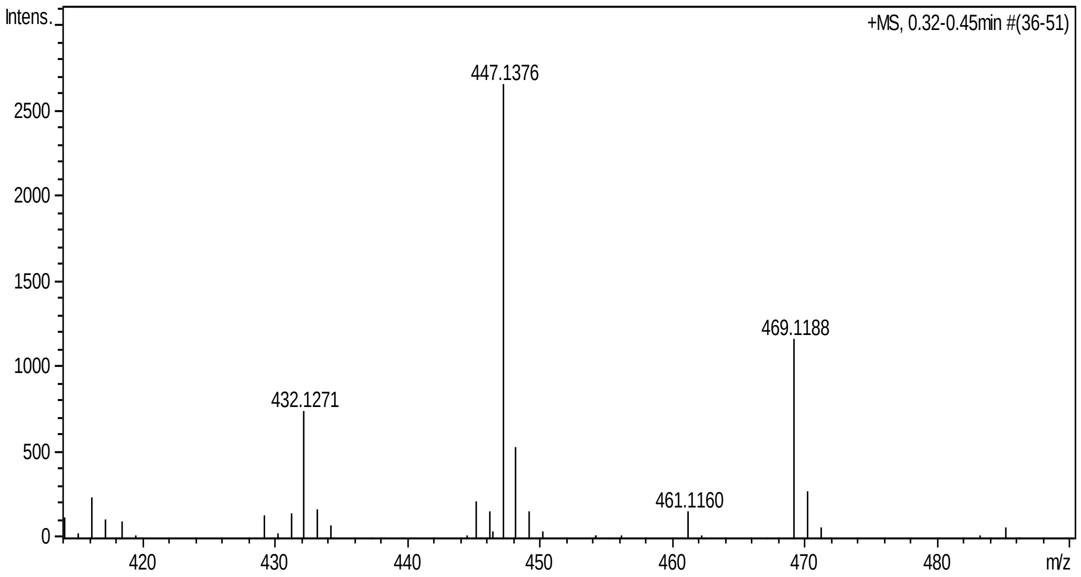

Intens.
+MS, 0.32-0.45min #(36-51)
447.1376
469.1188
432.1271
461.1160
2500
2000
1500
1000
500
0
420
430
440
450
460
470
480
m/z

(3-nitro-2-phenyl-1,2,3,3a-tetrahydrobenzo[d]pyrrolo[2,1-b]thiazol-1-yl)(p-tolyl)methanone (1b): yellow solid, 72%, m.p. 165~167℃; ^{1}H NMR (600 MHz, DMSO-d_6) δ: 7.77 (d, J = 7.8 Hz, 2H, ArH), 7.23~7.22 (m, 4H, ArH), 7.18~7.13 (m, 3H, ArH), 7.11~7.09 (m, 1H, ArH), 7.05~7.02 (m, 1H, ArH), 6.85~6.82 (m, 1H, ArH), 6.71 (d, J = 7.8 Hz, 1H, ArH), 6.39 (d, J = 6.6 Hz, 1H, CH), 6.07 (d, J = 8.4 Hz, 1H, CH), 5.95 (t, J = 6.0 Hz, 1H, CH), 4.70 (t, J = 6.6 Hz, 1H, CH), 2.32 (s, 3H, CH_3); ^{13}C NMR (150 MHz, DMSO-d_6) δ: 195.9, 147.1, 144.2, 134.5, 132.8, 129.2, 128.3, 128.2, 127.4, 126.8, 125.8, 121.7, 121.3, 111.4, 94.1, 72.2, 71.6, 50.3, 21.1; IR (KBr) υ: 3015, 2913, 1672, 1605, 1550, 1460, 1366, 1308, 1236, 1186, 1120, 1035, 986, 928, 886, 846, 792, 747 cm^{-1}; MS (m/z): HRMS (ESI) Calcd. for $C_{24}H_{20}N_2NaO_3S$ ($[M+Na]^+$): 439.1087. Found: 439.1082.

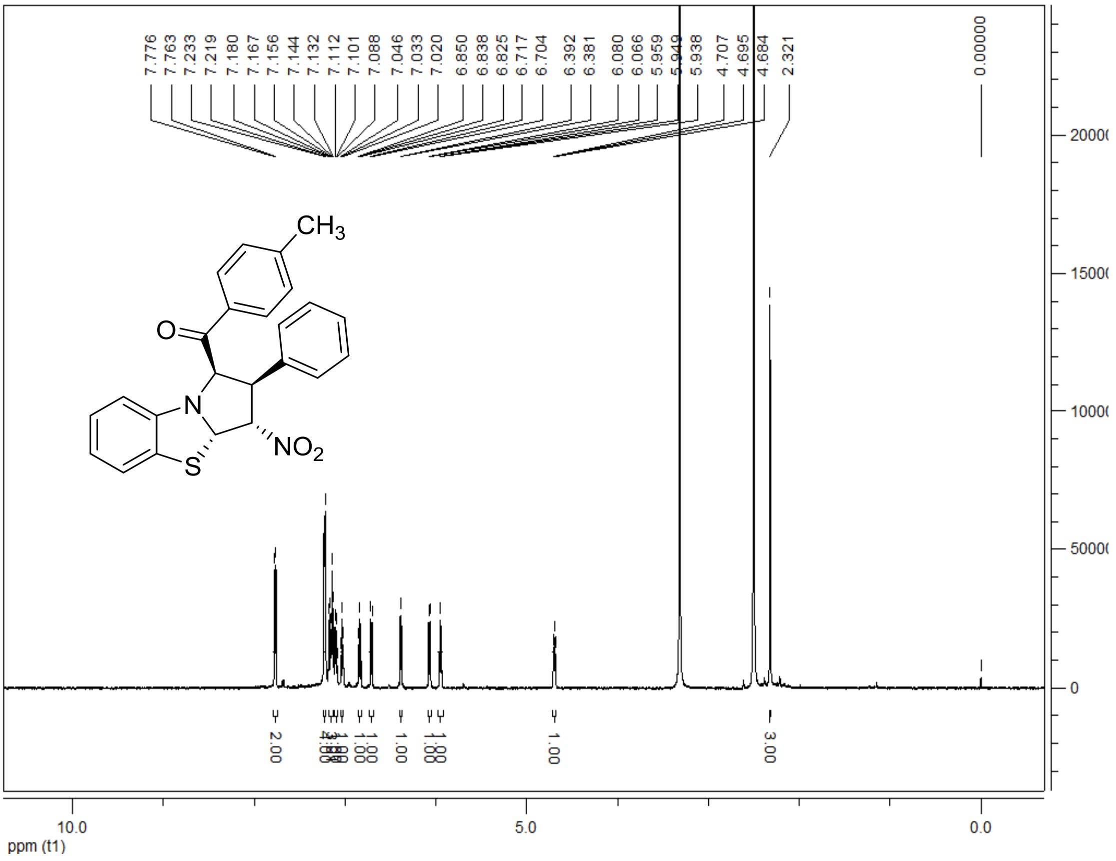

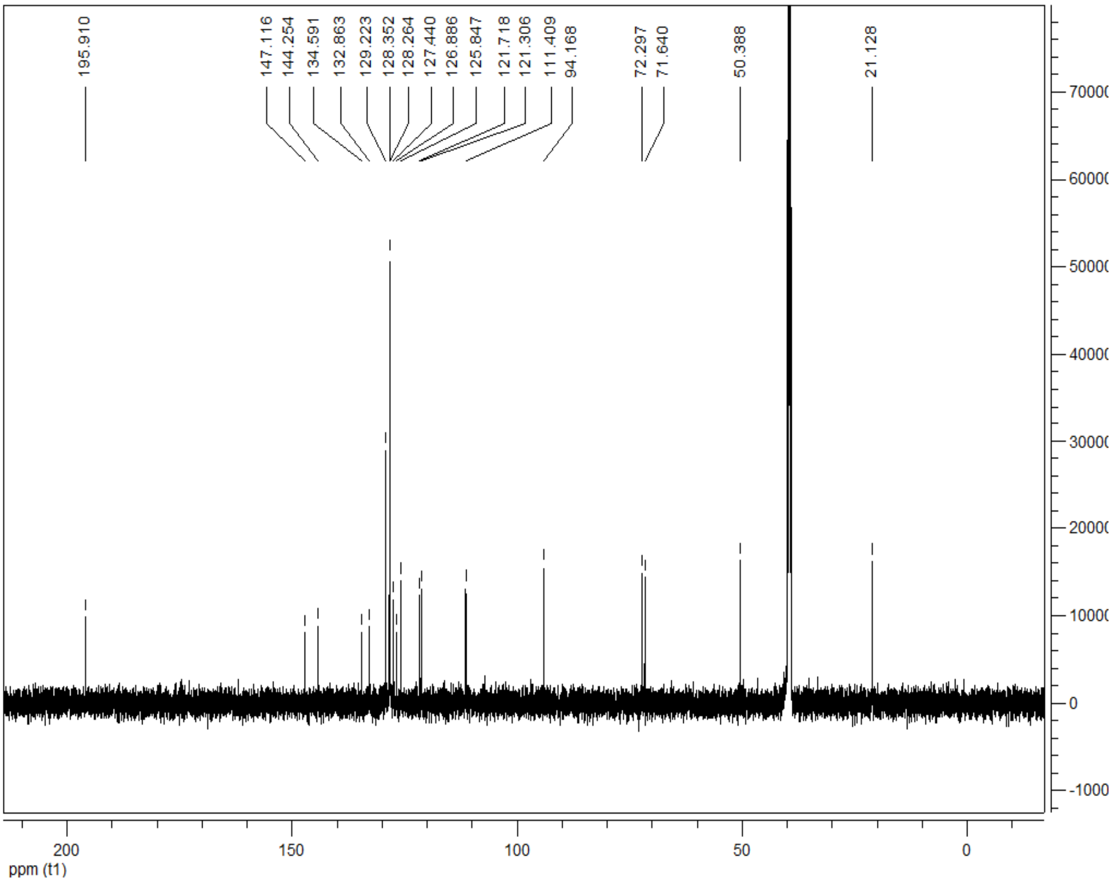

195.910
147.116
144.254
134.591
132.863
129.223
128.352
128.264
127.440
126.886
125.847
121.718
121.306
111.409
94.168
72.297
71.640
50.388
21.128
ppm (t1)

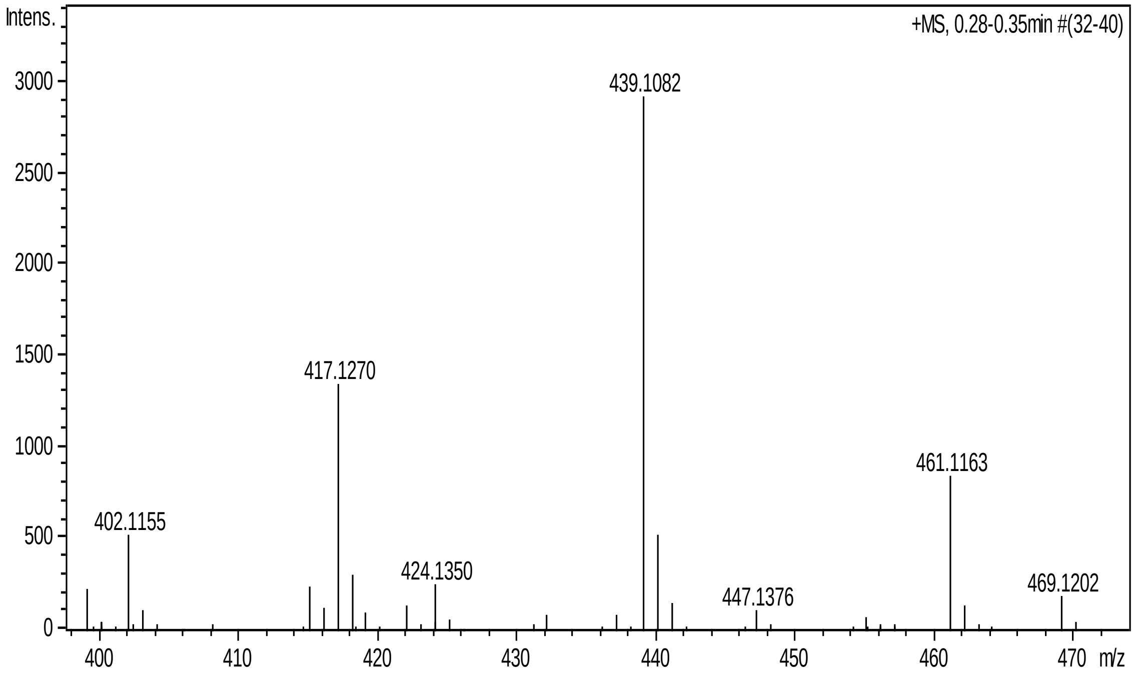

Intens.
+MS, 0.28-0.35min #(32-40)
439.1082
417.1270
461.1163
402.1155
424.1350
447.1376
469.1202
m/z

(2-(4-methoxyphenyl)-3-nitro-1,2,3,3a-tetrahydrobenzo[d]pyrrolo[2,1-b]thiazol-1-yl)(phenyl) methanone (1c): yellow solid, 70%, m.p. 136~138℃; ^{1}H NMR (400 MHz, DMSO-d_6) δ: 7.85~7.81 (m, 2H, ArH), 7.59~7.55 (m, 1H, ArH), 7.44~7.40 (m, 2H, ArH), 7.17~7.10 (m, 3H, ArH), 7.04~7.00 (m, 1H, ArH), 6.85~6.81 (m, 1H, ArH), 6.69~6.65 (m, 3H, ArH), 6.39 (d, J = 6.4 Hz, 1H, CH), 6.01 (d, J = 8.4 Hz, 1H, CH), 5.82 (t, J = 5.8 Hz, 1H, CH), 4.71~4.68 (m, 1H, CH), 3.62 (s, 3H, OCH_3); ^{13}C NMR (100 MHz, DMSO-d_6) δ: 196.5, 158.3, 147.0, 135.3, 133.4, 130.7, 129.4, 128.4, 127.9, 127.4, 126.1, 125.6, 121.4, 121.1, 113.6, 113.4, 111.2, 94.3, 72.2, 72.1, 54.8, 49.8; IR (KBr) υ: 3059, 2929, 1692, 1607, 1551, 1512, 1461, 1372, 1335, 1302, 1252, 1217, 1183, 1148, 1030, 989, 884, 835, 755 cm^{-1}; MS (m/z): HRMS (ESI) Calcd. for $C_{24}H_{20}N_2NaO_4S$ ($[M+H]^+$): 455.1036. Found: 455.1030.

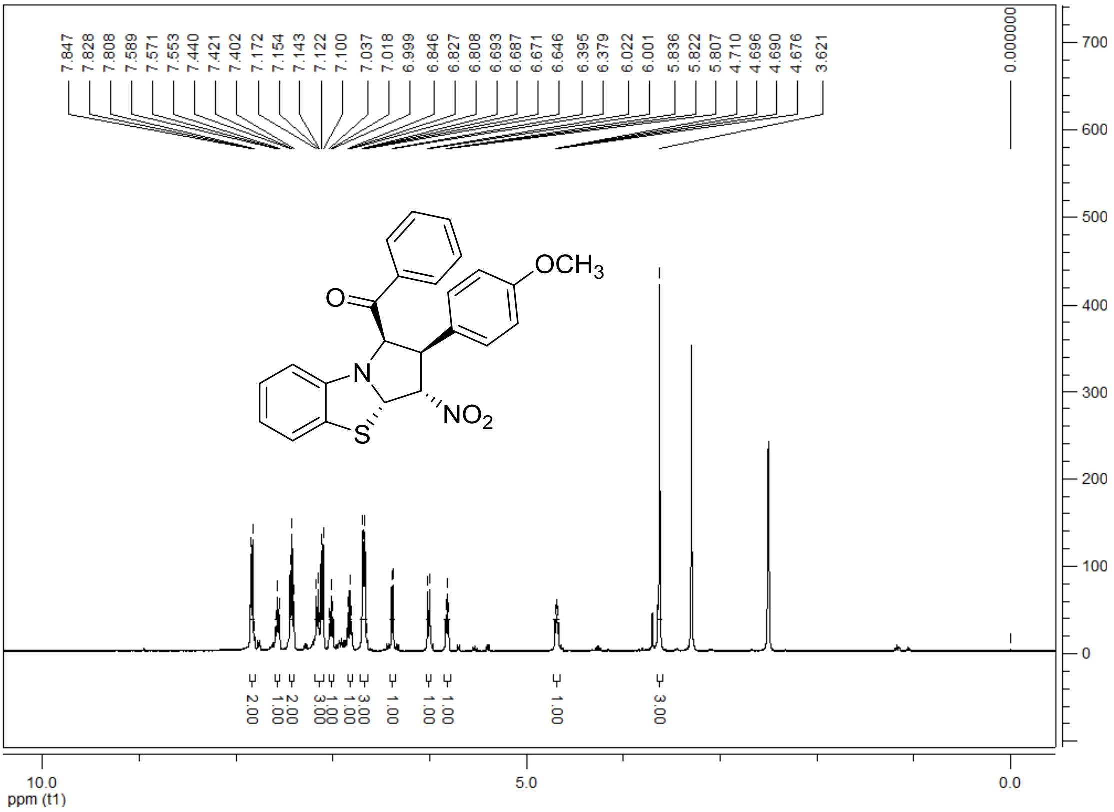

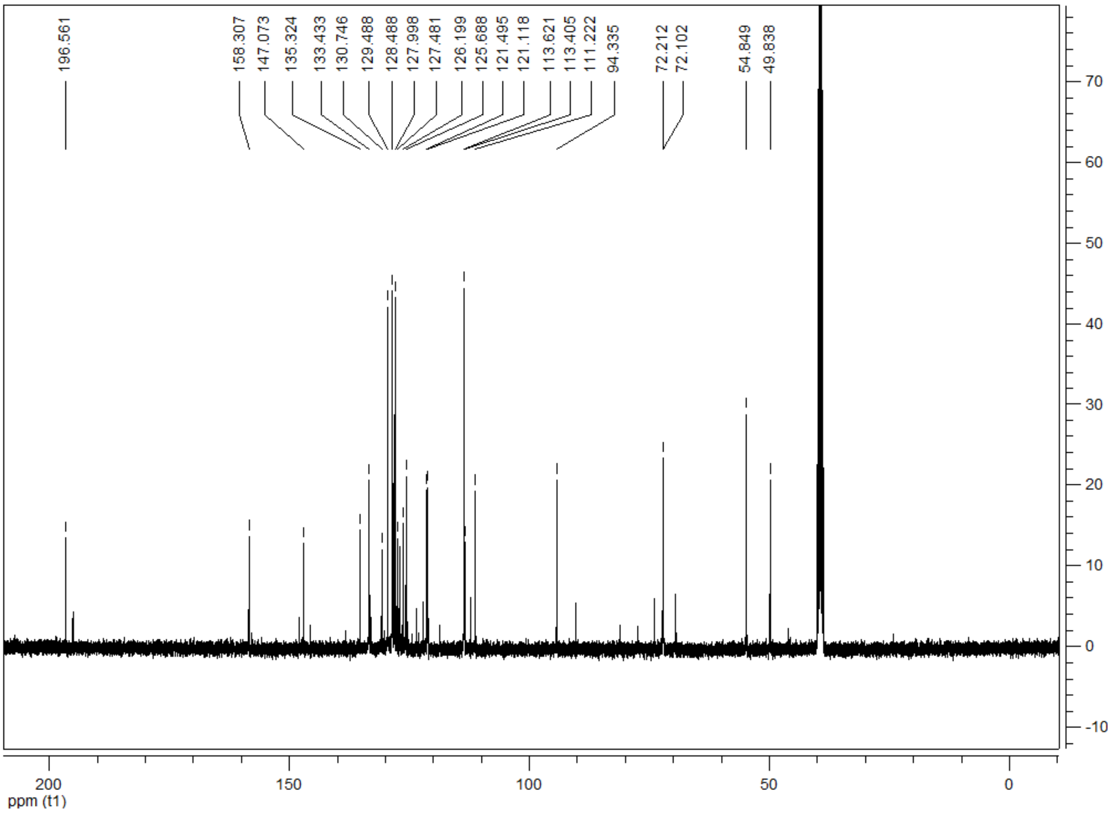
196.561
158.307
147.073
135.324
133.433
130.746
129.488
128.488
127.998
127.481
126.199
125.688
121.495
121.118
113.621
113.405
111.222
94.335
72.212
72.102
54.849
49.838
70
60
50
40
30
20
10
0
-10
200
150
100
50
0
ppm (t1)

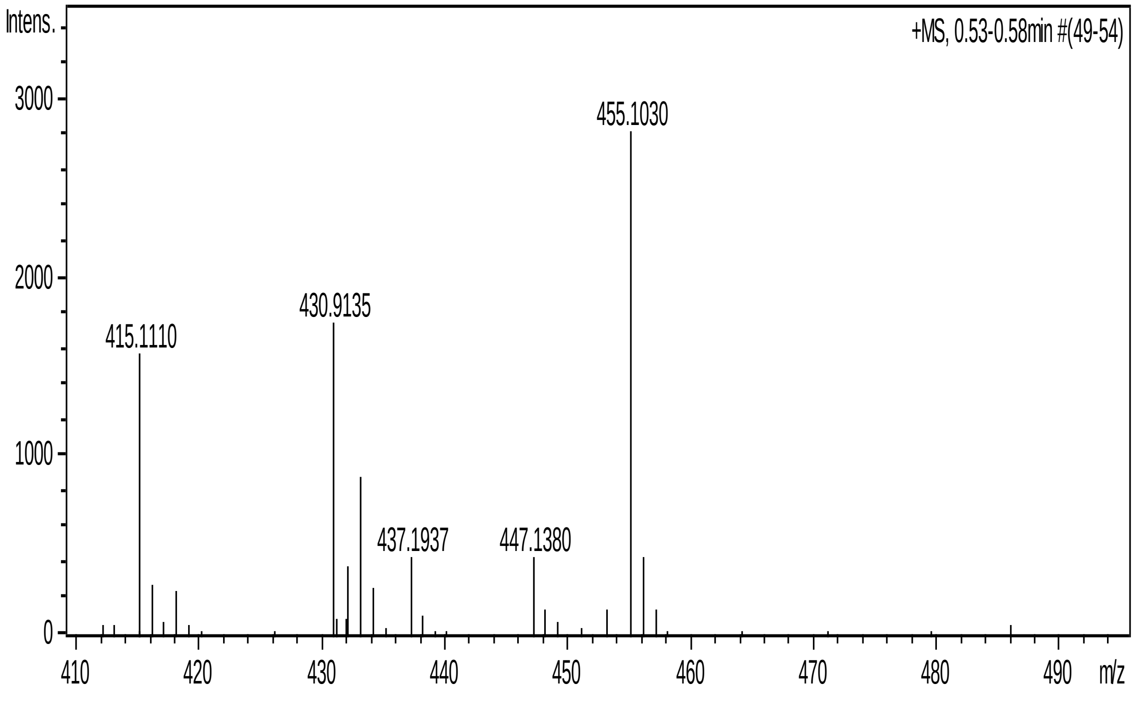
Intens.
+MS, 0.53-0.58min #(49-54)
3000
2000
1000
0
455.1030
430.9135
415.1110
437.1937
447.1380
410
420
430
440
450
460
470
480
490
m/z

(4-fluorophenyl)(3-nitro-2-phenyl-1,2,3,3a-tetrahydrobenzo[d]pyrrolo[2,1-b]thiazol-1-yl)methanone (1d): yellow solid, 78%, m.p. 162~164℃; ^{1}H NMR (600 MHz, $CDCl_3$) δ: 7.73~7.70 (m, 2H, ArH), 7.17~7.14 (m, 3H, ArH), 7.11 (d, J = 7.8 Hz, 1H, ArH), 7.08~7.07 (m, 2H, ArH), 7.04~7.00 (m, 3H, ArH), 6.92~6.89 (m, 1H, ArH), 6.64 (d, J = 7.8 Hz, 1H, ArH), 6.43 (d, J = 6.6 Hz, 1H, CH), 5.79 (t, J = 6.6 Hz, 1H, CH), 5.69 (d, J = 7.8 Hz, 1H, CH), 4.50 (t, J = 7.2 Hz, 1H, CH); ^{13}C NMR (100 MHz, $CDCl_3$) δ: 195.5, 147.1, 133.2, 130.7, 130.6, 128.9, 128.2, 128.1, 125.9, 122.9, 121.7, 116.0, 115.7, 112.0, 93.8, 73.3, 72.6, 51.3; IR (KBr) υ: 3052, 1773, 1692, 1592, 1550, 1476, 1408, 1364, 1301, 1227, 1157, 1110, 1038, 990, 929, 816, 748, 702 cm^{-1}; MS (m/z): HRMS (ESI) Calcd. for $C_{23}H_{17}FN_2NaO_3S$ ($[M+Na]^+$): 443.0836. Found: 443.0836.

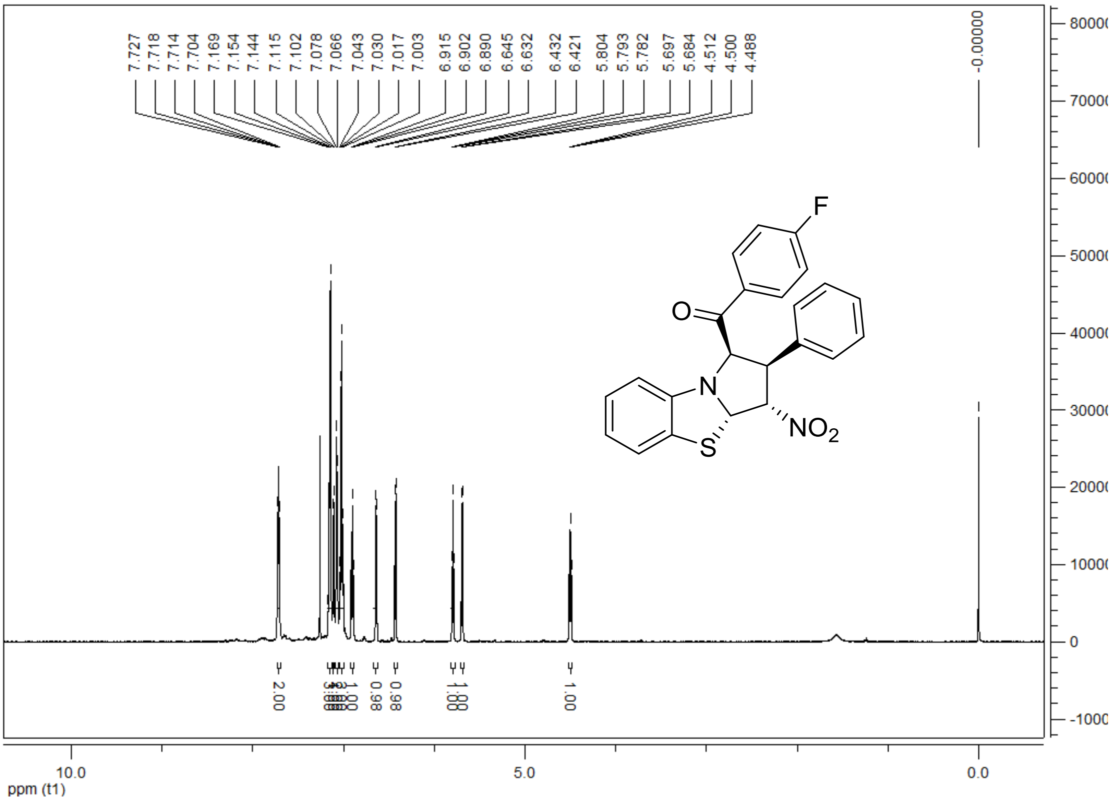

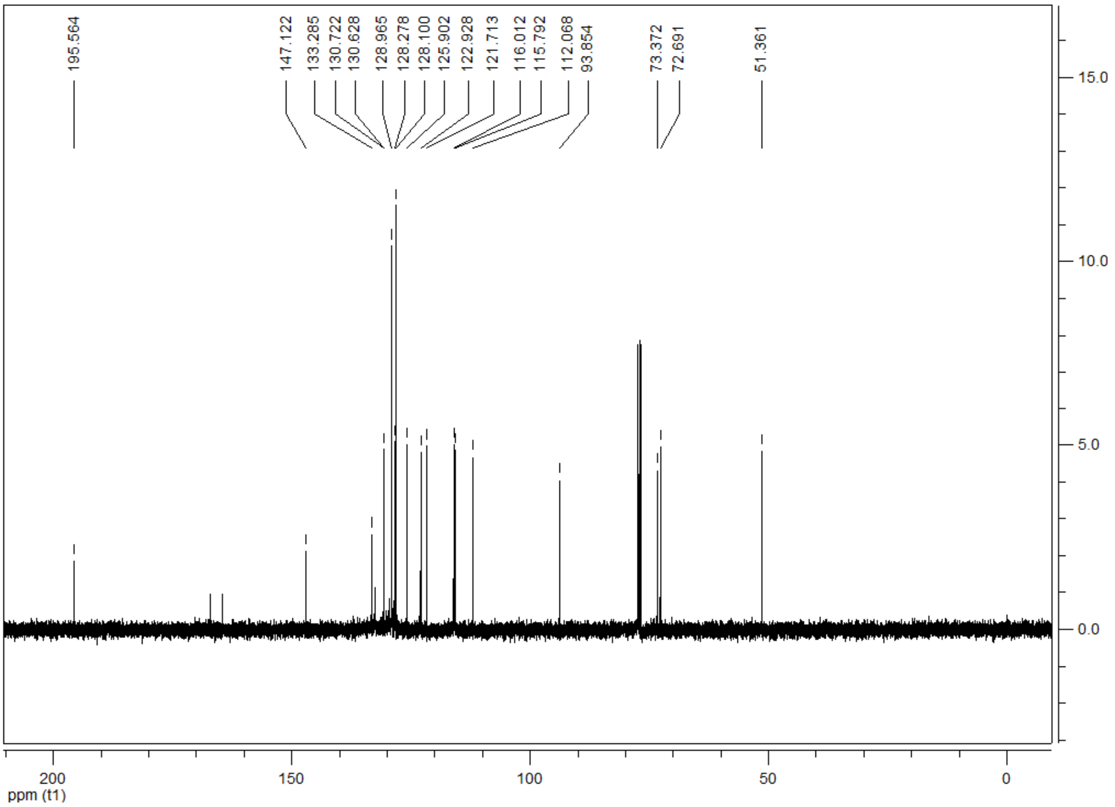
195.564
147.122
133.285
130.722
130.628
128.965
128.278
128.100
125.902
122.928
121.713
116.012
115.792
112.068
93.854
73.372
72.691
51.361
15.0
10.0
5.0
0.0
200
150
100
50
0
ppm (t1)

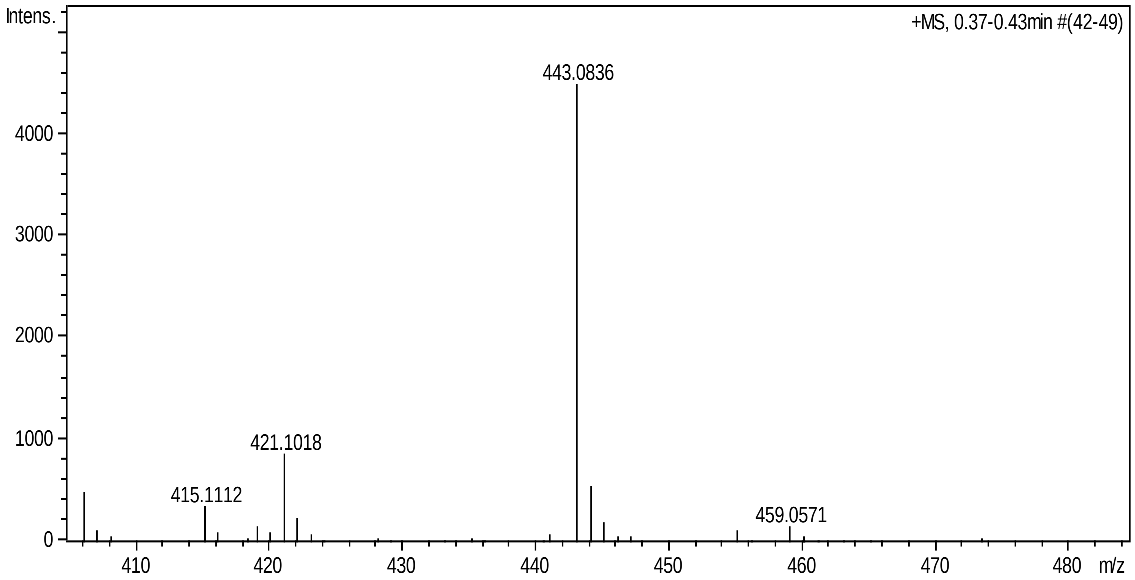
Intens.
+MS, 0.37-0.43min #(42-49)
443.0836
421.1018
415.1112
459.0571
4000
3000
2000
1000
0
410
420
430
440
450
460
470
480
m/z

(4-fluorophenyl)(3-nitro-2-(p-tolyl)-1,2,3,3a-tetrahydrobenzo[d]pyrrolo[2,1-b]thiazol-1-yl)methanone (1e): yellow solid, 75%, m.p. 166~168℃; ^{1}H NMR (600 MHz, DMSO-d_6) δ: 8.98 (s, 1H, ArH), 7.82 (d, *J* = 8.4 Hz, 1H, ArH), 7.77 (s, 1H, ArH), 7.59 (brs, 2H, ArH), 7.26~7.22 (m, 3H, ArH), 7.20~7.19 (m, 1H, CH), 7.12 (t, *J* = 7.2 Hz, 1H, CH), 6.90 (d, *J* = 7.2 Hz, 2H, ArH), 6.40 (d, *J* = 7.2 Hz, 2H, ArH), 4.60 (d, *J* = 5.4 Hz, 1H, CH), 4.14 (d, *J* = 5.4 Hz, 1H, CH), 2.17 (s, 3H, CH_3); ^{13}C NMR (100 MHz, DMSO-d_6) δ: 195.4, 163.3, 160.9, 145.7, 137.9, 135.8, 134.5, 132.9, 130.2, 129.5, 128.8, 126.4, 125.5, 124.6, 123.0, 118.7, 115.2, 115.0, 80.8, 77.2, 46.5, 20.4; IR (KBr) υ: 2975, 1691, 1598, 1545, 1466, 1372, 1231, 1154, 1051, 965, 920, 822, 743 cm^{-1}; MS (*m/z*): HRMS (ESI) Calcd. for $C_{24}H_{19}FN_2NaO_3S$ ([M+Na]$^+$): 457.0993. Found: 457.0988.

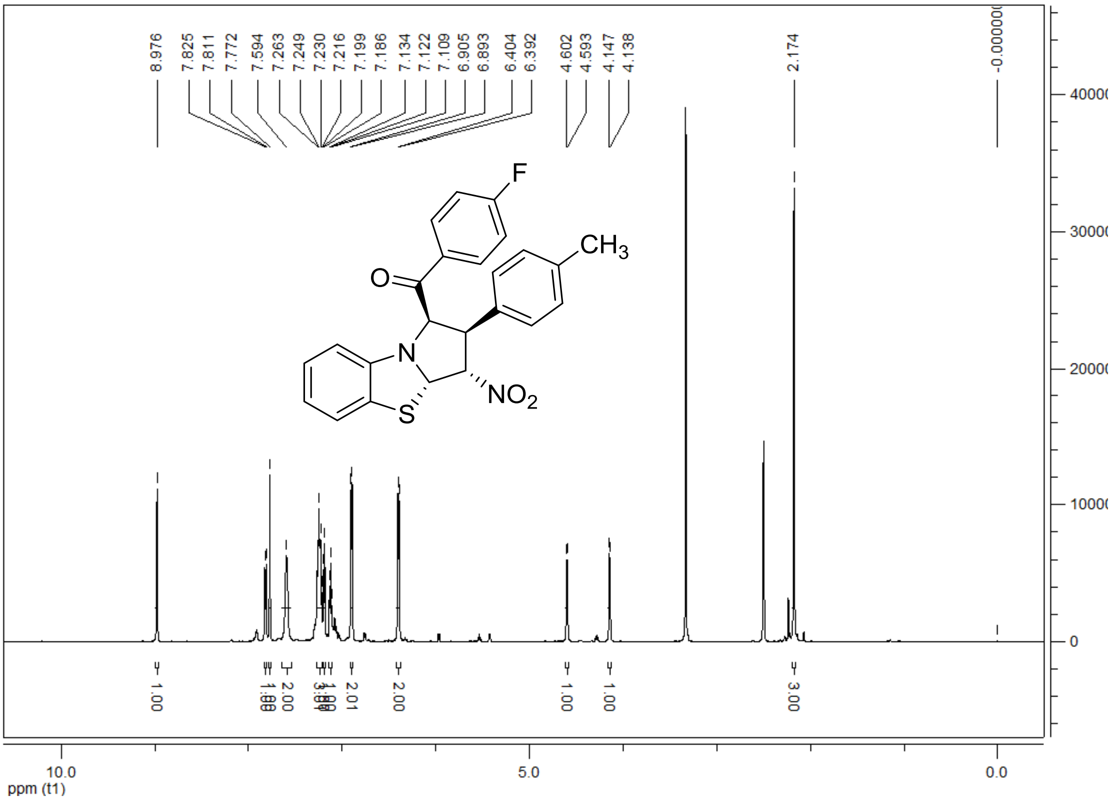

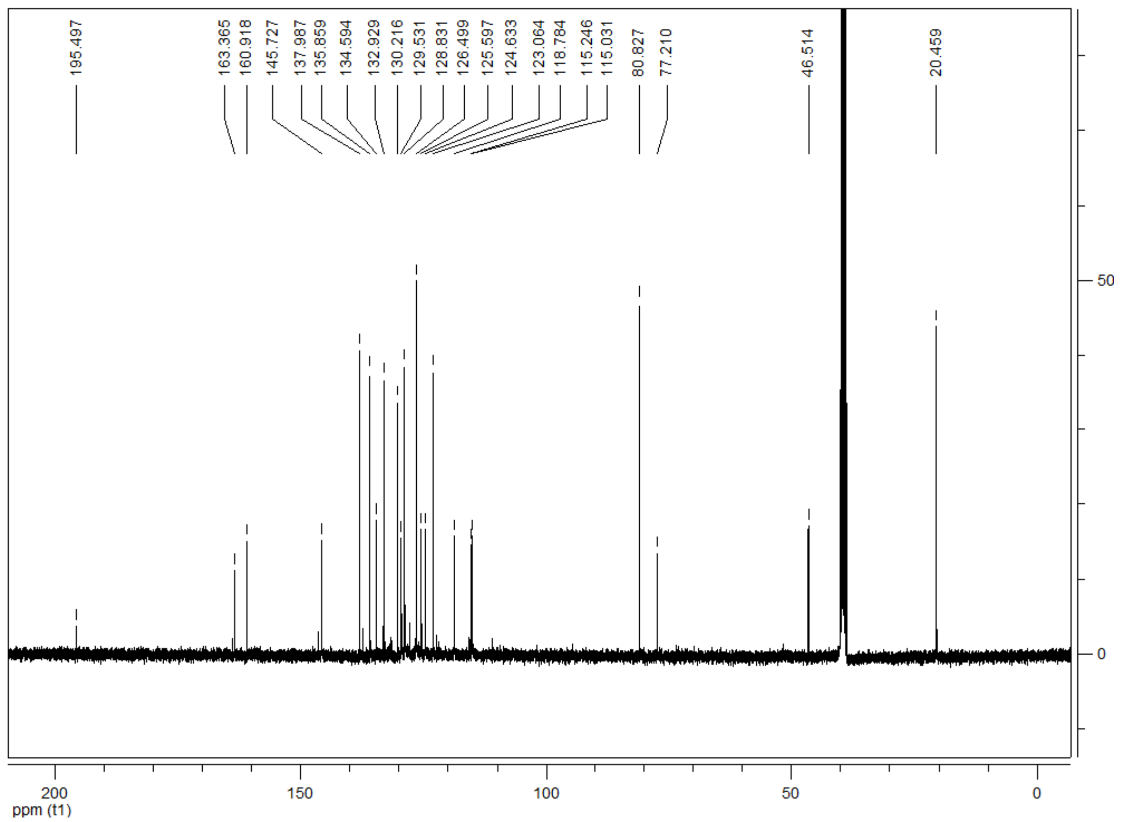
195.497
163.365
160.918
145.727
137.987
135.859
134.594
132.929
130.216
129.531
128.831
126.499
125.597
124.633
123.064
118.784
115.246
115.031
80.827
77.210
46.514
20.459
50
0
200
150
100
50
0
ppm (t1)

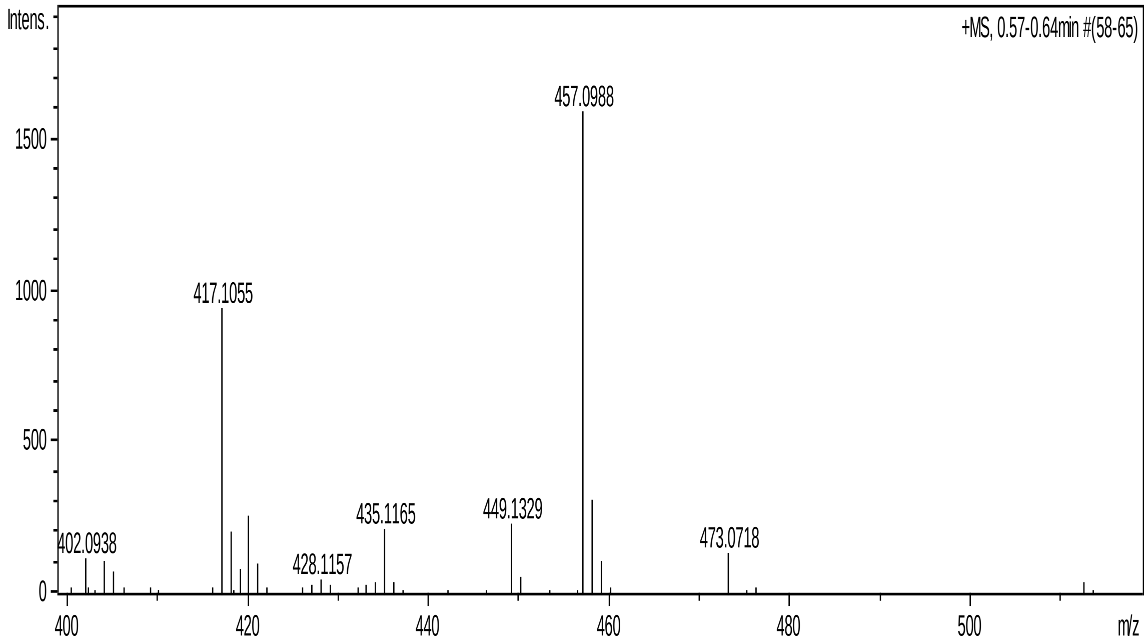
Intens.
+MS, 0.57-0.64min #(58-65)
457.0988
417.1055
435.1165
449.1329
402.0938
428.1157
473.0718
1500
1000
500
0
400
420
440
460
480
500
m/z

(4-fluorophenyl)(3-nitro-2-(p-tolyl)-1,2,3,3a-tetrahydrobenzo[d]pyrrolo[2,1-b]thiazol-1-yl)methanone (1e'): yellow solid, 20%, m.p. 135~137℃; ^{1}H NMR (600 MHz, DMSO-d_6) δ: 7.91 (brs, 2H, ArH), 7.29~7.26 (m, 3H, ArH), 7.08~7.03 (m, 5H, ArH), 6.91~6.89 (m, 1H, ArH), 6.76 (d, J = 7.2 Hz, 1H, ArH), 5.97 (d, J = 7.2 Hz, 1H, CH), 5.53 (t, J = 8.1 Hz, 1H, CH), 5.42 (d, J = 6.6 Hz, 1H, CH), 4.28 (t, J = 7.8 Hz, 1H, CH) , 2.24 (s, 3H, CH_3); ^{13}C NMR (150 MHz, DMSO-d_6) δ: 195.5, 146.4, 137.3, 133.3, 131.8, 129.4, 127.8, 126.5, 126.0, 125.4, 122.4, 122.0, 115.8, 115.6, 111.0, 94.5, 73.3, 73.0, 51.7, 39.5, 20.5; IR (KBr) υ: 3067, 2917, 1920, 1686, 1594, 1549, 1509, 1468, 1353, 1300, 1234, 1155, 1021, 982, 847, 815, 750 cm^{-1}; MS (m/z): HRMS (ESI) Calcd. for $C_{24}H_{19}FN_2NaO_3S$ ($[M+H]^+$): 457.0993. Found: 457.0994.

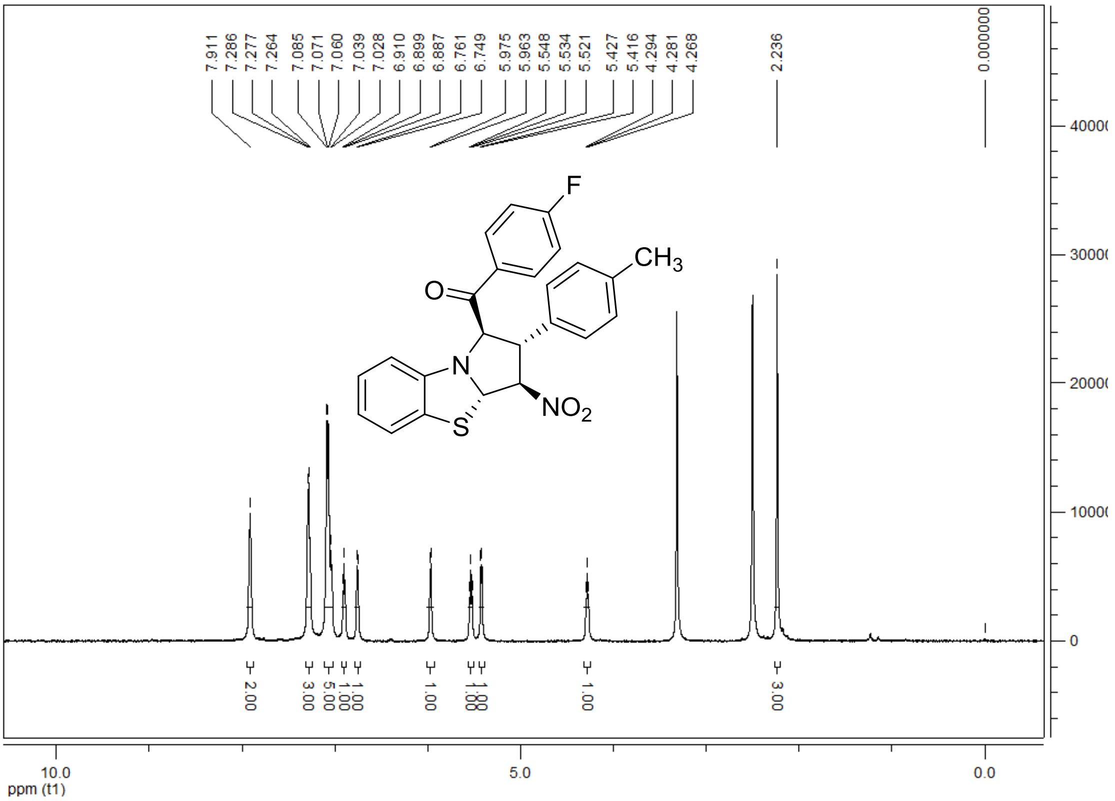

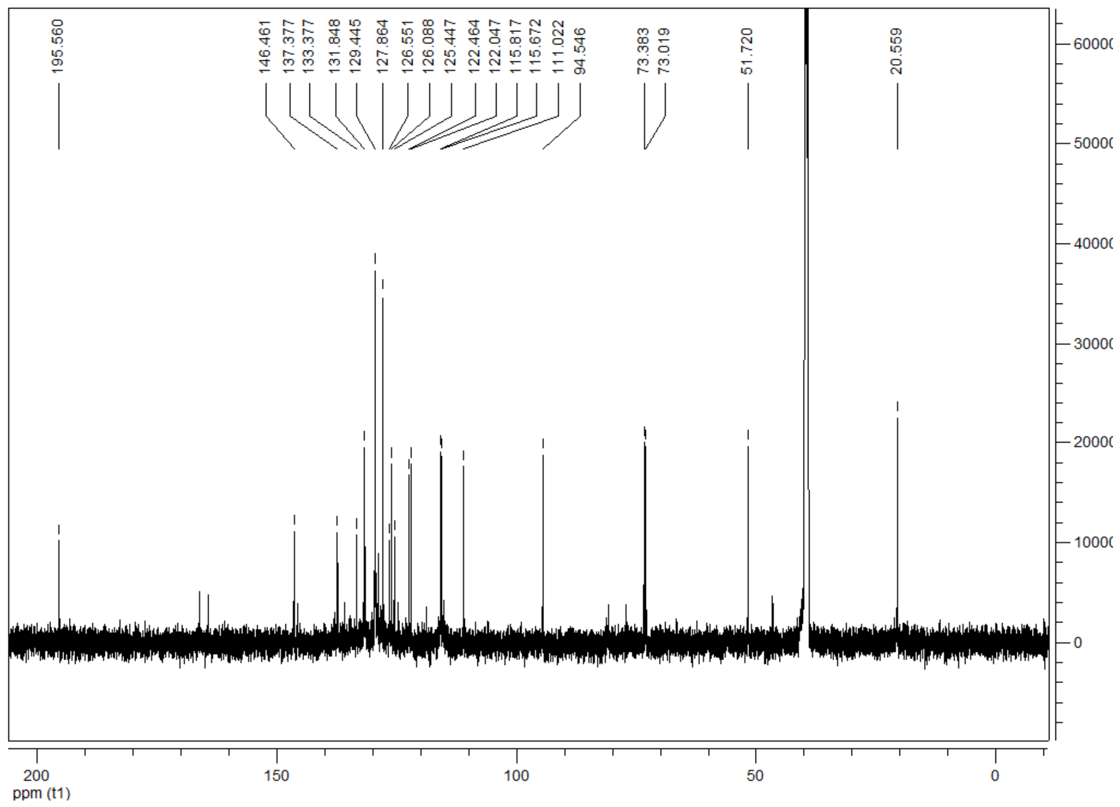
195.560
146.461
137.377
133.377
131.848
129.445
127.864
126.551
126.088
125.447
122.464
122.047
115.817
115.672
111.022
94.546
73.383
73.019
51.720
20.559
200
150
100
50
0
ppm (t1)

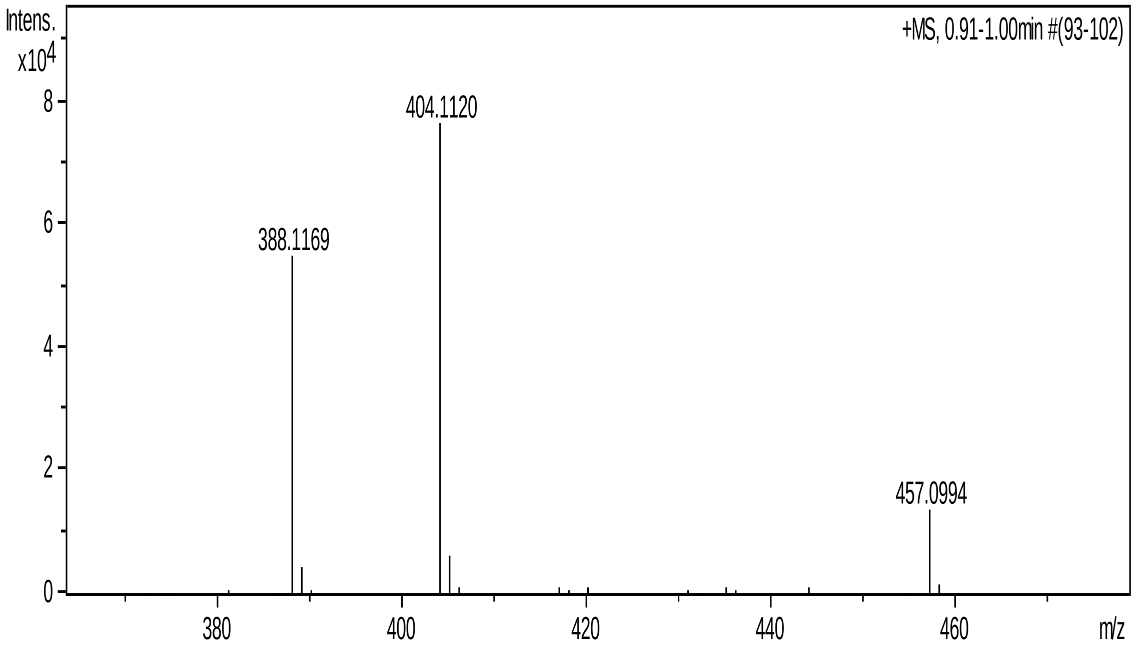
Intens.
x10^4
+MS, 0.91-1.00min #(93-102)
404.1120
388.1169
457.0994
8
6
4
2
0
380
400
420
440
460
m/z

(4-methoxyphenyl)(2-(4-methoxyphenyl)-3-nitro-1,2,3,3a-tetrahydrobenzo[d]pyrrolo[2,1-b]thiazol-1-yl)methanone (1f): yellow solid, 79%, m.p. 153~155℃; ^{1}H NMR (400 MHz, DMSO-d_6) δ: 7.89 (d, *J* = 8.8 Hz, 2H, ArH), 7.21~7.14 (m, 3H, ArH), 7.05~7.01 (m, 1H, ArH), 6.96 (d, *J* = 8.8 Hz, 2H, ArH), 6.85~6.81 (m, 1H, ArH), 6.72~6.67 (m, 3H, ArH), 6.38 (d, *J* = 6.4 Hz, 1H, CH), 6.00 (d, *J* = 8.0 Hz, 1H, CH), 5.91 (t, *J* = 6.4 Hz, 1H, CH), 4.62 (t, *J* = 6.4 Hz, 1H, CH), 3.81 (s, 3H, OCH_3), 3.63 (s, 3H, OCH_3); ^{13}C NMR (100 MHz, DMSO-d_6) δ: 194.6, 163.4, 158.2, 147.1, 130.6, 129.4, 128.2, 126.8, 126.2, 125.7, 121.5, 121.2, 113.8, 113.6, 111.3, 94.4, 72.2, 71.3, 55.4, 54.8, 49.8; IR (KBr) υ: 2988, 1689, 1600, 1551, 1513, 1466, 1371, 1257, 1174, 1027, 890, 833, 750 cm^{-1}; MS (*m/z*): HRMS (ESI) Calcd. for $C_{25}H_{22}N_2NaO_5S$ ($[M+Na]^+$): 485.1142. Found: 485.1137.

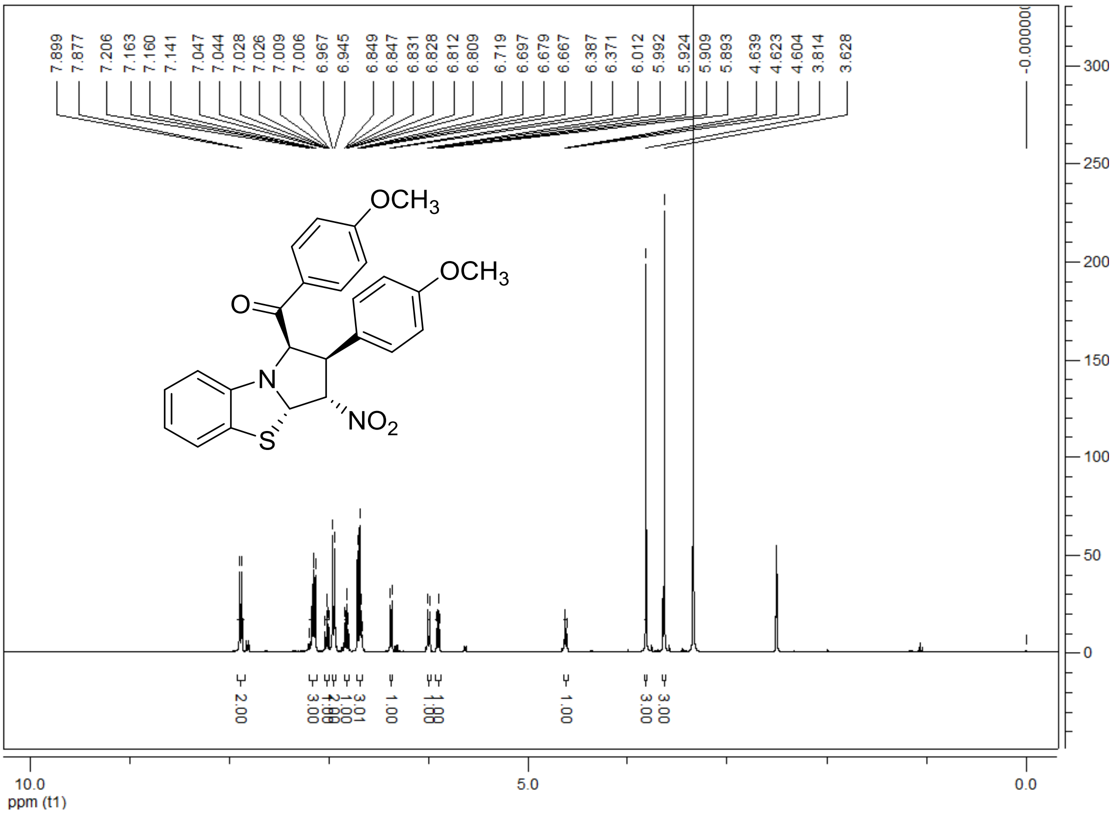

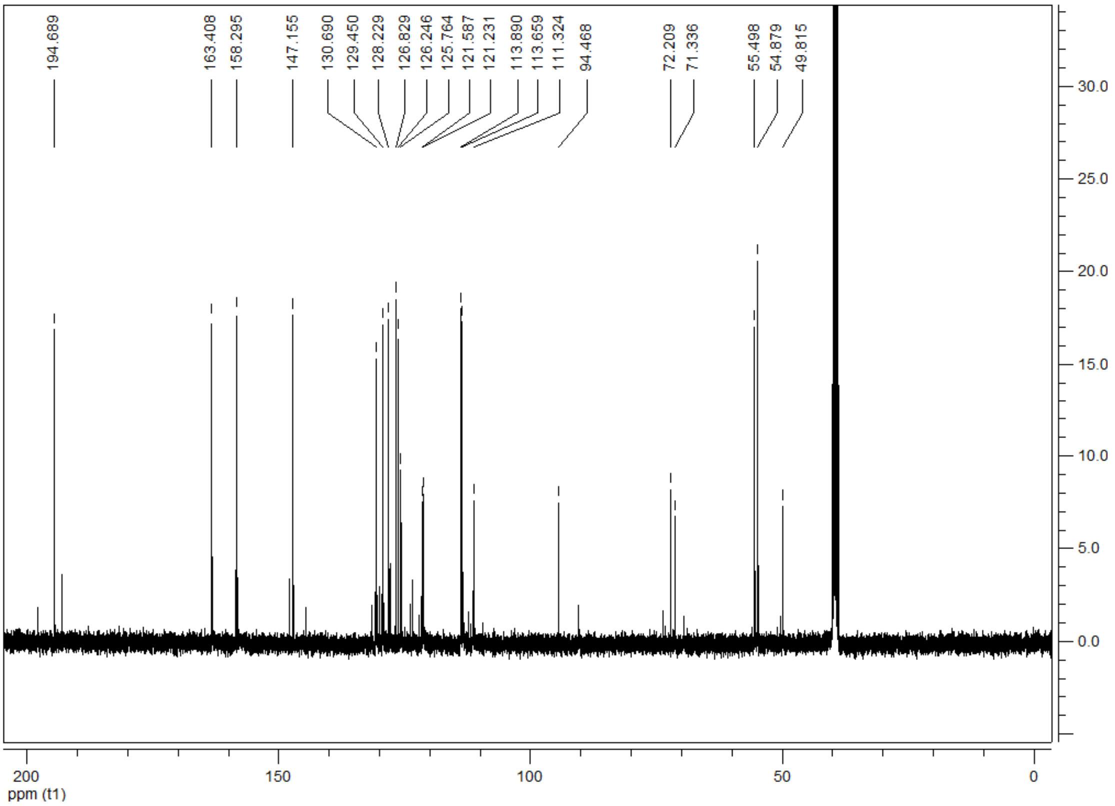
194.689
163.408
158.295
147.155
130.690
129.450
128.229
126.829
126.246
125.764
121.587
121.231
113.890
113.659
111.324
94.468
72.209
71.336
55.498
54.879
49.815
30.0
25.0
20.0
15.0
10.0
5.0
0.0
200
150
100
50
0
ppm (t1)

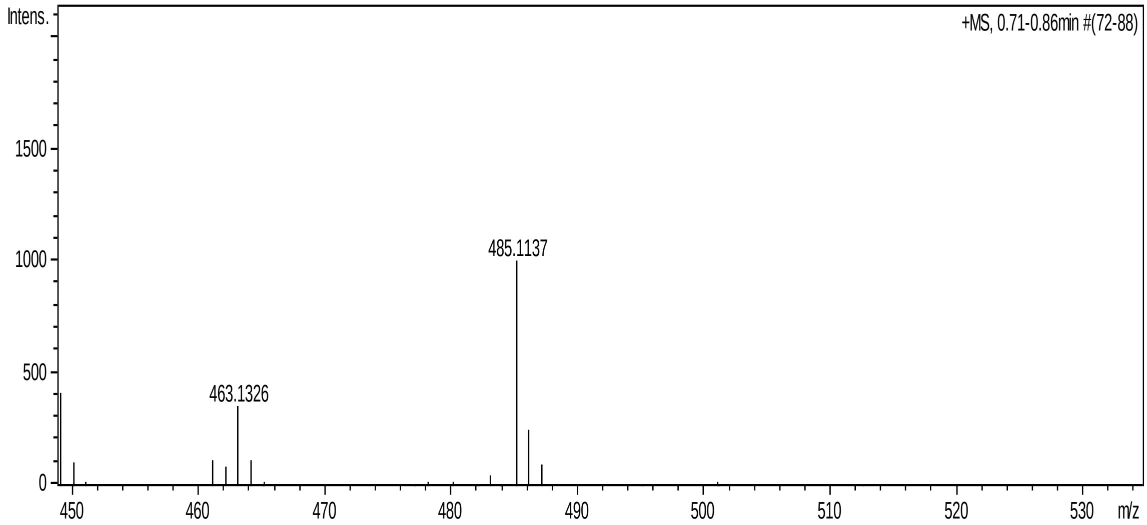
Intens.
+MS, 0.71-0.86min #(72-88)
1500
1000
500
0
485.1137
463.1326
450
460
470
480
490
500
510
520
530
m/z

(4-methoxyphenyl)(2-(4-methoxyphenyl)-3-nitro-1,2,3,3a-tetrahydrobenzo[d]pyrrolo[2,1-b]thiazol-1-yl)methanone (1f'): yellow solid, 17%, m.p. 144~146℃; ^{1}H NMR (400 MHz, DMSO-d_6) δ: 7.83~7.81 (m, 2H, ArH), 7.28 (d, J = 7.6 Hz, 1H, ArH), 7.18 (d, J = 8.4 Hz, 2H, ArH), 7.03~6.96 (m, 3H, ArH), 6.90~6.86 (m, 1H, ArH), 6.82 (d, J = 8.4 Hz, 2H, ArH), 6.65~6.60 (m, 1H, ArH), 6.01 (d, J = 7.6 Hz, 1H, CH), 5.57 (t, J = 8.8 Hz, 1H, CH), 5.36 (d, J = 8.0 Hz, 1H, CH), 4.22 (t, J = 8.8 Hz, 1H, CH), 3.82 (s, 3H, OCH_3), 3.70 (m, 3H, OCH_3); ^{13}C NMR (100 MHz, DMSO-d_6) δ: 195.0, 163.6, 158.9, 146.6, 131.1, 129.1, 127.4, 125.9, 125.6, 122.2, 121.8, 114.2, 113.9, 110.8, 94.3, 81.2, 73.5, 72.1, 55.5, 55.0, 52.1; IR (KBr) υ: 3069, 1688, 1595, 1547, 1513, 1466, 1251, 1176, 1025, 837, 757 cm^{-1}; MS (m/z): HRMS (ESI) Calcd. for $C_{25}H_{22}N_2NaO_5S$ ($[M+Na]^+$): 485.1142. Found: 485.1141.

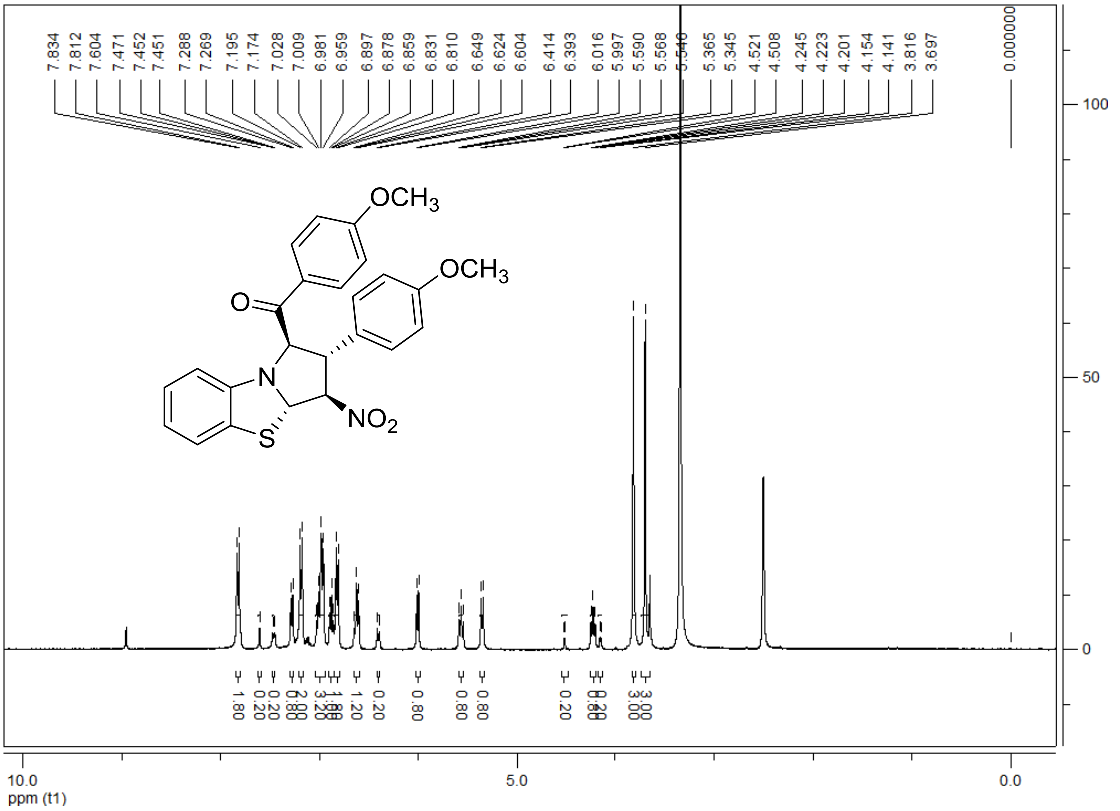

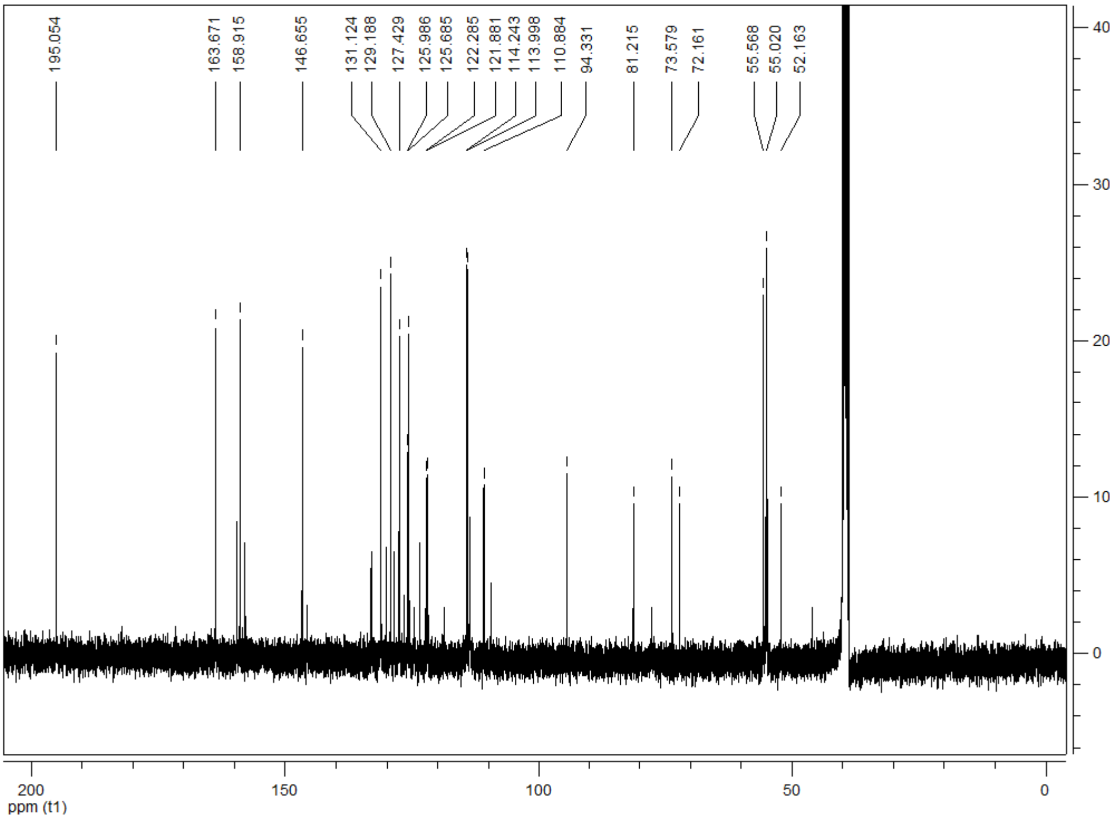
195.054
163.671
158.915
146.655
131.124
129.188
127.429
125.986
125.685
122.285
121.881
114.243
113.998
110.884
94.331
81.215
73.579
72.161
55.568
55.020
52.163
40
30
20
10
0
200
150
100
50
0
ppm (t1)

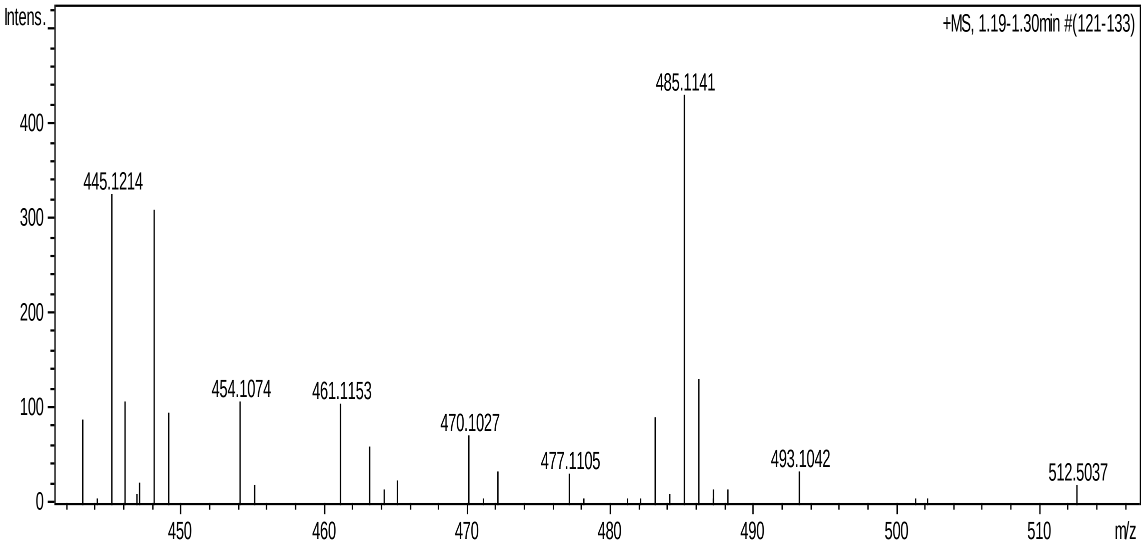
Intens.
+MS, 1.19-1.30min #(121-133)
485.1141
445.1214
454.1074
461.1153
470.1027
477.1105
493.1042
512.5037
400
300
200
100
0
450
460
470
480
490
500
510
m/z

(2-(4-chlorophenyl)-3-nitro-1,2,3,3a-tetrahydrobenzo[d]pyrrolo[2,1-b]thiazol-1-yl)(phenyl)methanone (1g): yellow solid, 76%, m.p. 161~163℃; ^{1}H NMR (400 MHz, DMSO-d_6) major isomer: δ: 7.85 (d, *J* = 7.6 Hz, 2H, ArH), 7.61~7.57 (m, 1H, ArH), 7.46~7.42 (m, 2H, ArH), 7.26~7.24 (m, 2H, ArH), 7.21~7.17 (m, 3H, ArH), 7.04 (t, *J* = 8.0 Hz, 1H, ArH), 6.86~6.83 (m, 1H, ArH), 6.74 (d, *J* = 8.0 Hz, 1H, ArH), 6.39 (d, *J* = 6.4 Hz, 1H, CH), 6.12 (d, *J* = 8.0 Hz, 1H, CH), 5.95 (t, *J* = 6.0 Hz, 1H, CH), 4.76 (t, *J* = 6.4 Hz, 1H, CH); minor isomer: δ: 7.78 (d, *J* = 7.6 Hz, 2H, ArH), 6.97 (t, *J* = 8.0 Hz, 1H, ArH), 6.46 (d, *J* = 8.0 Hz, 1H, CH), 5.77 (d, *J* = 6.4 Hz, 1H, CH). ratio of major/minor = 4:1. ^{13}C NMR (100 MHz, DMSO-d_6) δ: 196.9, 195.5, 148.2, 147.4, 135.6, 134.2, 134.1, 133.3, 132.6, 131.9, 131.5, 130.7, 129.1, 128.7, 128.6, 128.1, 127.3, 126.3, 122.9, 122.2, 121.7, 112.0, 94.2, 90.5, 74.4, 72.7, 72.4, 50.1; IR (KBr) υ: 3047, 1789, 1688, 1583, 1554, 1474, 1403, 1366, 1310, 1228, 1110, 1040, 991, 935, 820, 748, 710 cm^{-1}; MS (*m*/*z*): HRMS (ESI) Calcd. for $C_{23}H_{18}ClN_2O_3S$ ($[M+H]^+$): 437.0727. Found: 437.1933.

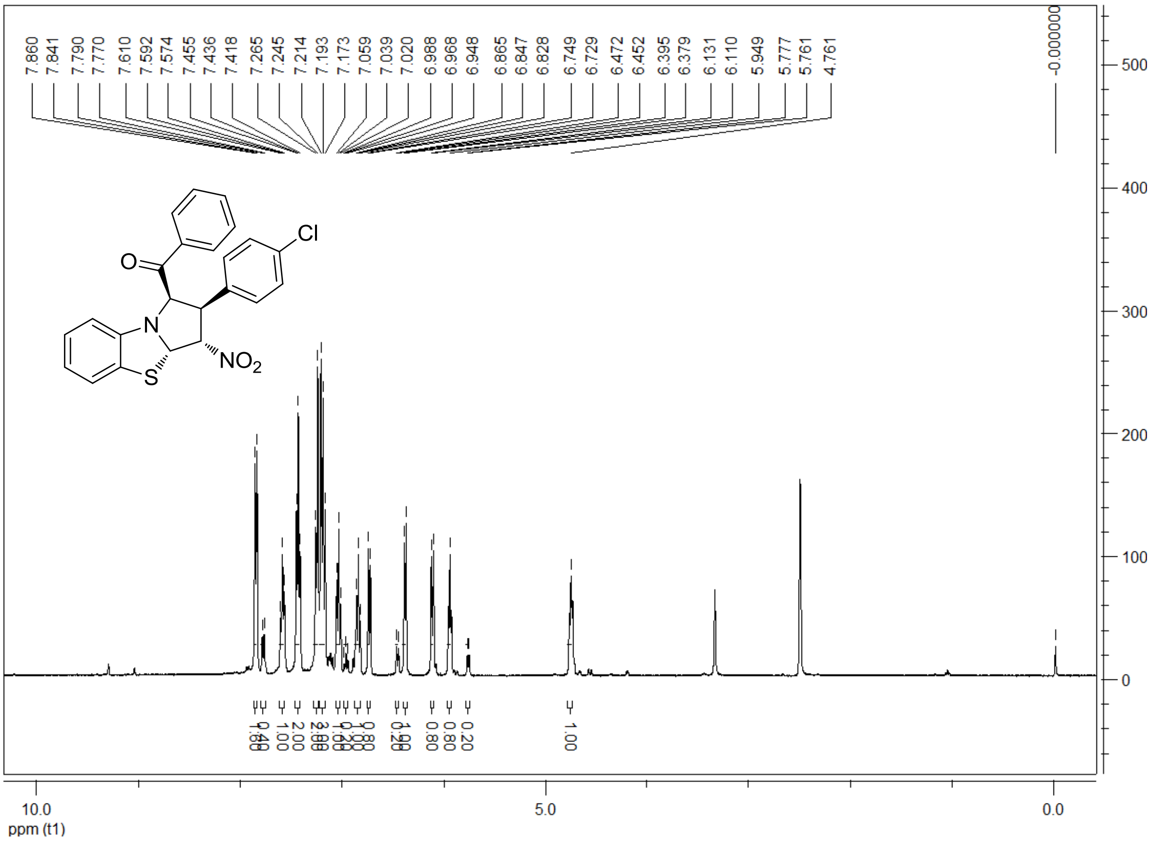

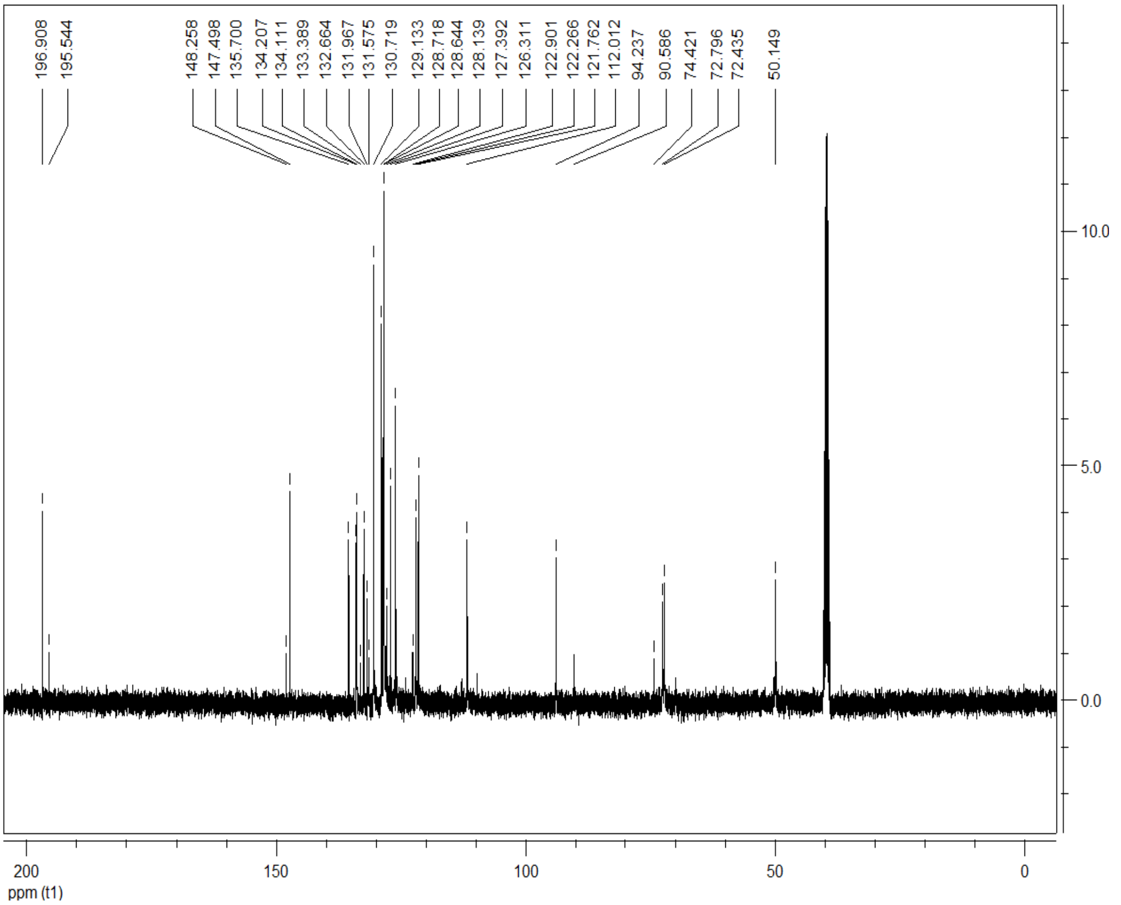
196.908
195.544
148.258
147.498
135.700
134.207
134.111
133.389
132.664
131.967
131.575
130.719
129.133
128.718
128.644
128.139
127.392
126.311
122.901
122.266
121.762
112.012
94.237
90.586
74.421
72.796
72.435
50.149
10.0
5.0
0.0
200
150
100
50
0
ppm (t1)

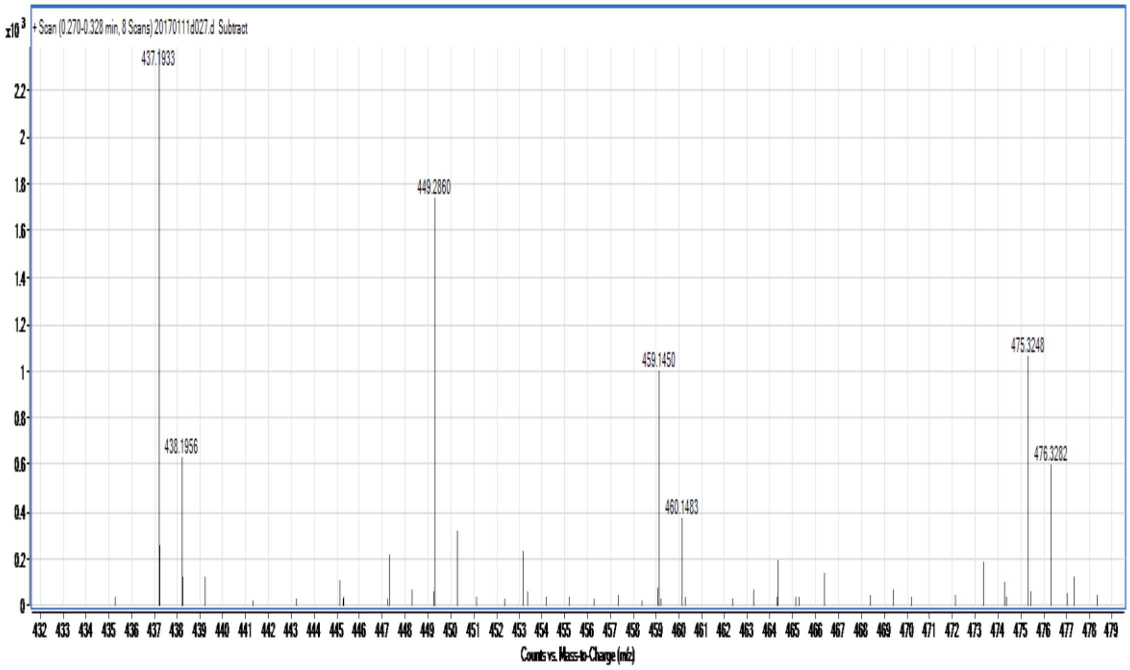
x10^3
+ Scan (0.270-0.328 min, 8 Scans) 20170111d027.d Subtract
437.1933
438.1956
449.2860
459.1450
460.1483
475.3248
476.3282
Counts vs. Mass-to-Charge (m/z)

(2-(3-methoxyphenyl)-3-nitro-1,2,3,3a-tetrahydrobenzo[d]pyrrolo[2,1-b]thiazol-1-yl)(p-tolyl) methanone (1h): yellow solid, 90%, m.p. 155~157℃; ^{1}H NMR (400 MHz, DMSO-d_6) major isomer δ: 7.76 (d, J = 7.8 Hz, 2H, ArH), 7.20 (d, J = 7.6 Hz, 2H, ArH), 7.14 (d, J = 7.6 Hz, 1H, ArH), 7.04~6.98 (m, 2H, ArH), 6.84~6.78 (m, 2H, ArH), 6.75 (d, J = 7.6 Hz, 1H, ArH), 6.69 (d, J = 7.9 Hz, 1H, ArH), 6.62 (d, J = 8.0 Hz, 1H, ArH), 6.34 (d, J = 6.8 Hz, 1H, CH), 6.06 (d, J = 8.4 Hz, 1H, CH), 5.95 (t, J = 6.0 Hz, 1H, CH), 4.61 (t, J = 6.8 Hz, 1H, CH), 3.57 (s, 3H, OCH_3), 2.29 (s, 3H, CH_3); minor isomer: δ: 7.67 (d, J = 7.7 Hz, 1H, ArH), 6.94~6.90 (m, 1H, ArH), 6.56 (d, J = 8.0 Hz, 1H, ArH), 6.32~6.29 (m, 1H, CH), 6.03~5.99 (m, 1H, CH), 5.65~5.63 (m, 1H, CH), 4.68~4.66 (m, 1H, CH). ratio of major/minor = 4:1. ^{13}C NMR (100 MHz, DMSO-d_6) δ: 196.4, 159.4, 147.5, 144.7, 136.4, 133.3, 129.9, 129.6, 128.9, 128.8, 127.2, 126.2, 122.1, 121.7, 120.5, 114.5, 113.5, 111.9, 94.5, 94.4, 72.8, 72.7, 72.0, 71.9, 55.3, 55.2, 50.8, 50.7, 21.6, 21.5; IR (KBr) υ: 3035, 2937, 1688, 1612, 1587, 1460, 1369, 1311, 1234, 1190, 1117, 994, 918, 877, 845, 790, 733 cm^{-1}; MS (m/z): HRMS (ESI) Calcd. for $C_{25}H_{22}N_2NaO_4S$ ($[M+Na]^+$): 469.1198. Found: 469.1155.

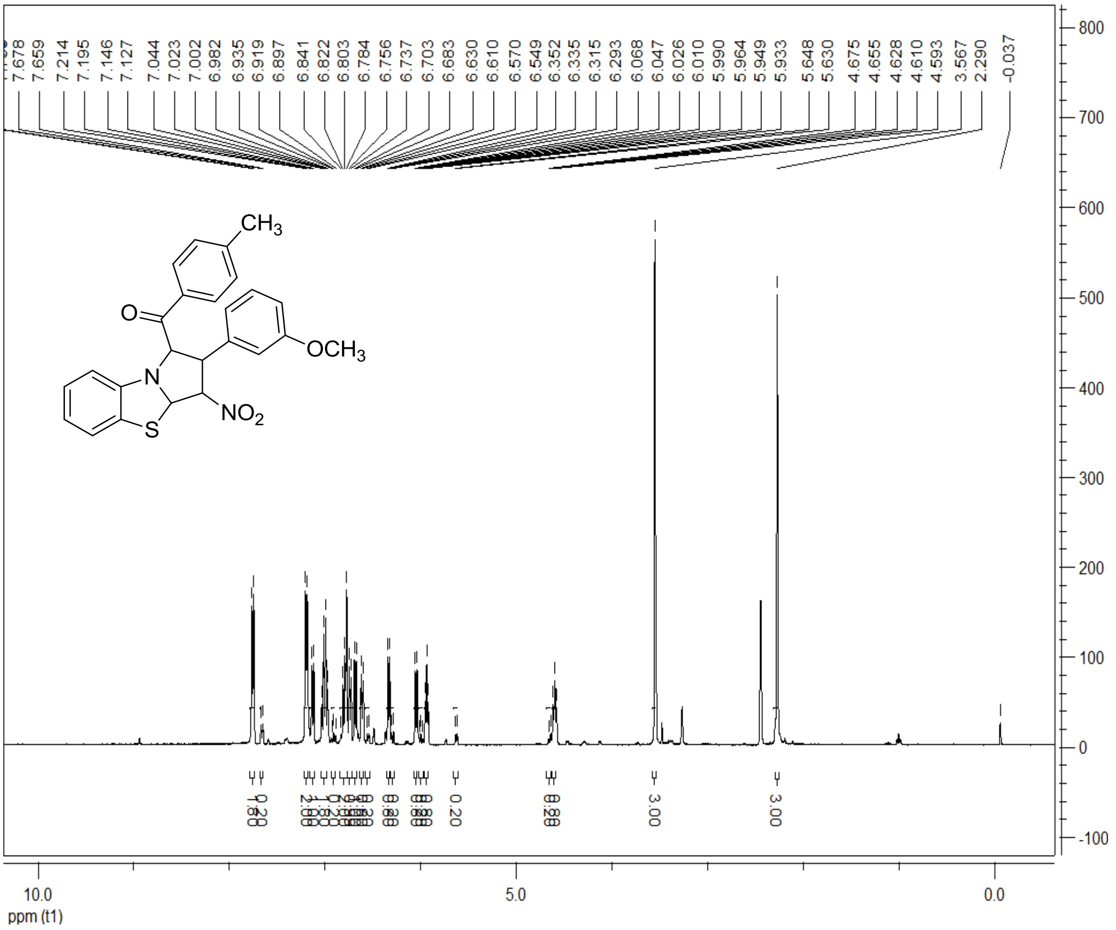

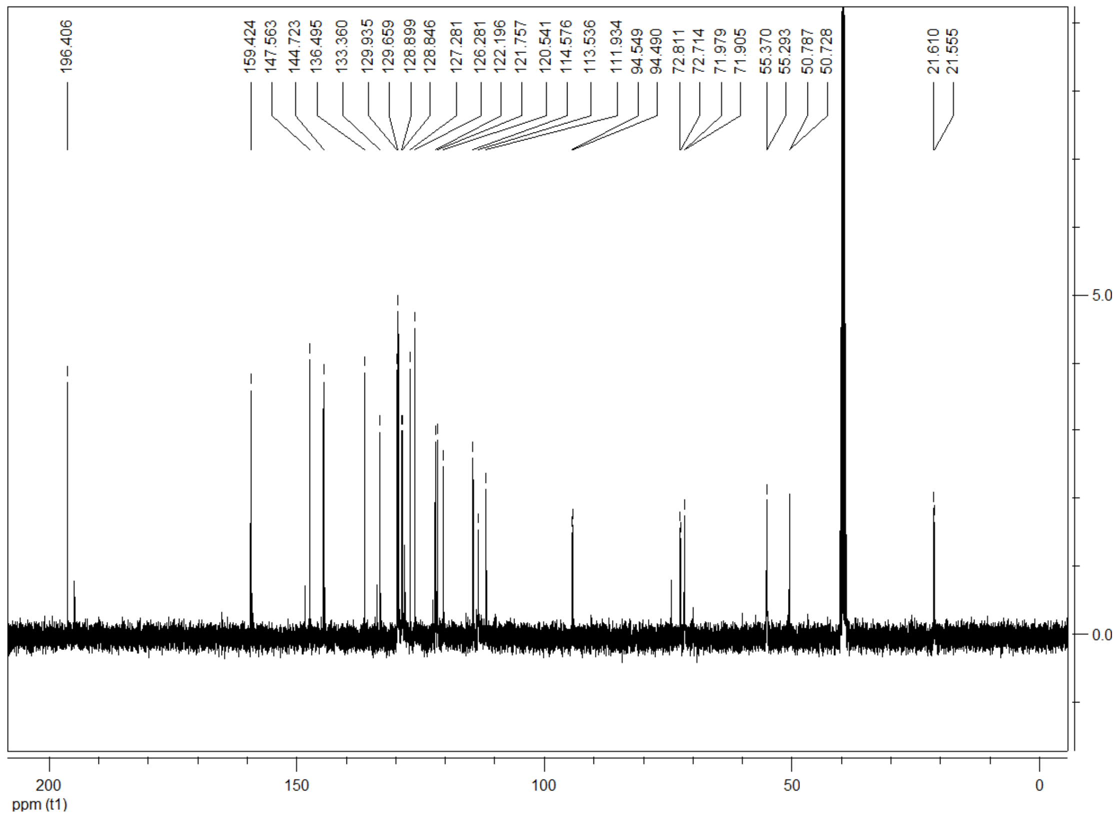
196.406
159.424
147.563
144.723
136.495
133.360
129.935
129.659
128.899
128.846
127.281
126.281
122.196
121.757
120.541
114.576
113.536
111.934
94.549
94.490
72.811
72.714
71.979
71.905
55.370
55.293
50.787
50.728
21.610
21.555
5.0
0.0
200
150
100
50
0
ppm (t1)

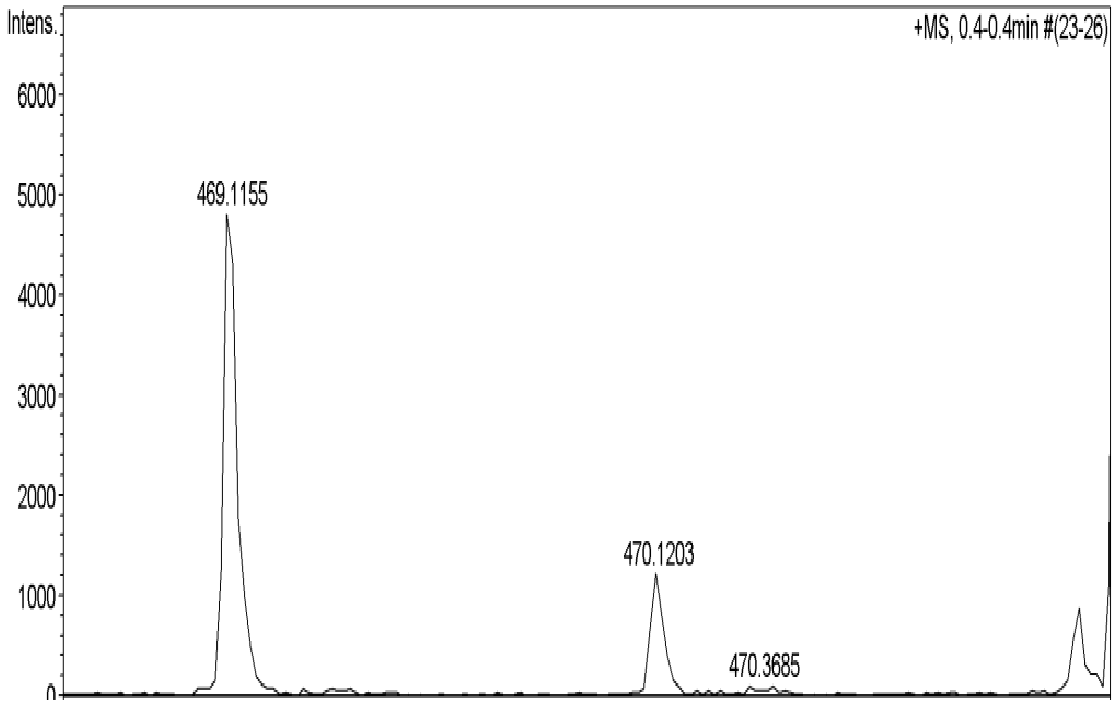
Intens.
+MS, 0.4-0.4min #(23-26)
6000
5000
4000
3000
2000
1000
0
469.1155
470.1203
470.3685

(4-chlorophenyl)(2-(4-methoxyphenyl)-3-nitro-1,2,3,3a-tetrahydrobenzo[d]pyrrolo[2,1-b]thiazol-1-yl)methanone (1i): yellow solid, 78%, m.p. 140~142℃; ^{1}H NMR (400 MHz, $CDCl_3$) major isomer δ: 7.71 (d, J = 8.4 Hz, 1H, ArH), 7.57 (d, J = 8.4 Hz, 1H, ArH), 7.37~7.32 (m, 2H, ArH), 7.03~6.86 (m, 6H, ArH), 6.80 (d, J = 8.4 Hz, 1H, ArH), 6.68~6.49 (m, 3H, ArH, CH), 5.69~5.63 (m, 1H, CH), 5.18~5.09 (m, 1H, CH), 3.71~3.65 (m, 3H, OCH_3); minor isomer: δ: 7.63 (d, J = 8.8 Hz, 2H, ArH), 7.10 (d, J = 7.2 Hz, 2H, ArH), 6.40 (d, J = 6.8 Hz, 2H, CH), 5.46 (t, J = 8.2 Hz, 1H, CH), 5.29 (d, J = 6.8 Hz, 1H, CH), 4.49~4.44 (m, 2H, CH), 3.80~3.72 (m, 3H, OCH_3). ratio of major/minor = 3:2. ^{13}C NMR (100 MHz, $CDCl_3$) δ: 195.8, 158.3, 147.0, 138.3, 134.0, 132.7, 129.8, 128.5, 126.8, 126.1, 125.6, 121.0, 113.6, 111.2, 94.2, 80.6, 72.2, 54.8, 54.8, 49.8; IR (KBr) υ: 3066, 2926, 2840, 1695, 1585, 1547, 1513, 1467, 1365, 1299, 1255, 1184, 1148, 1092, 1034, 888, 825, 752 cm^{-1}; MS (m/z): HRMS (ESI) Calcd. for $C_{24}H_{20}ClN_2O_4S$ ($[M+H]^+$): 467.0827. Found: 467.0826.

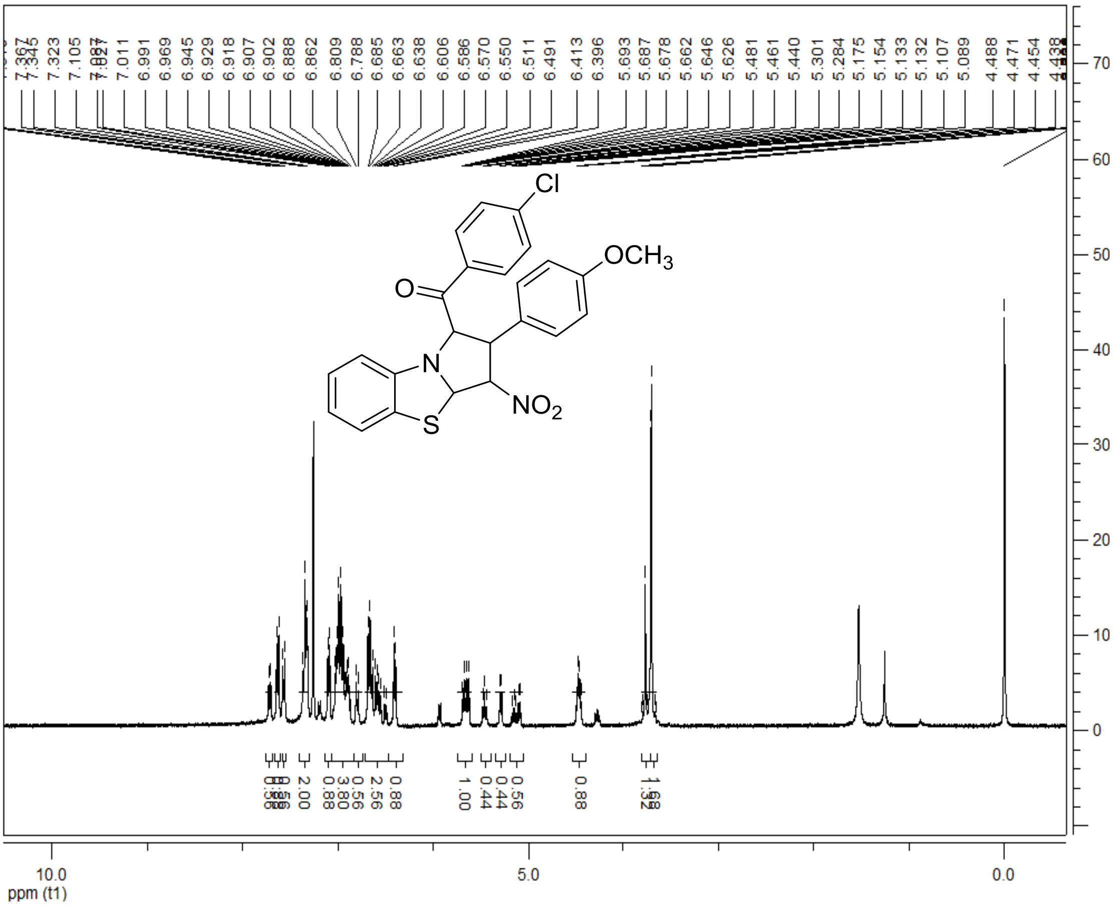

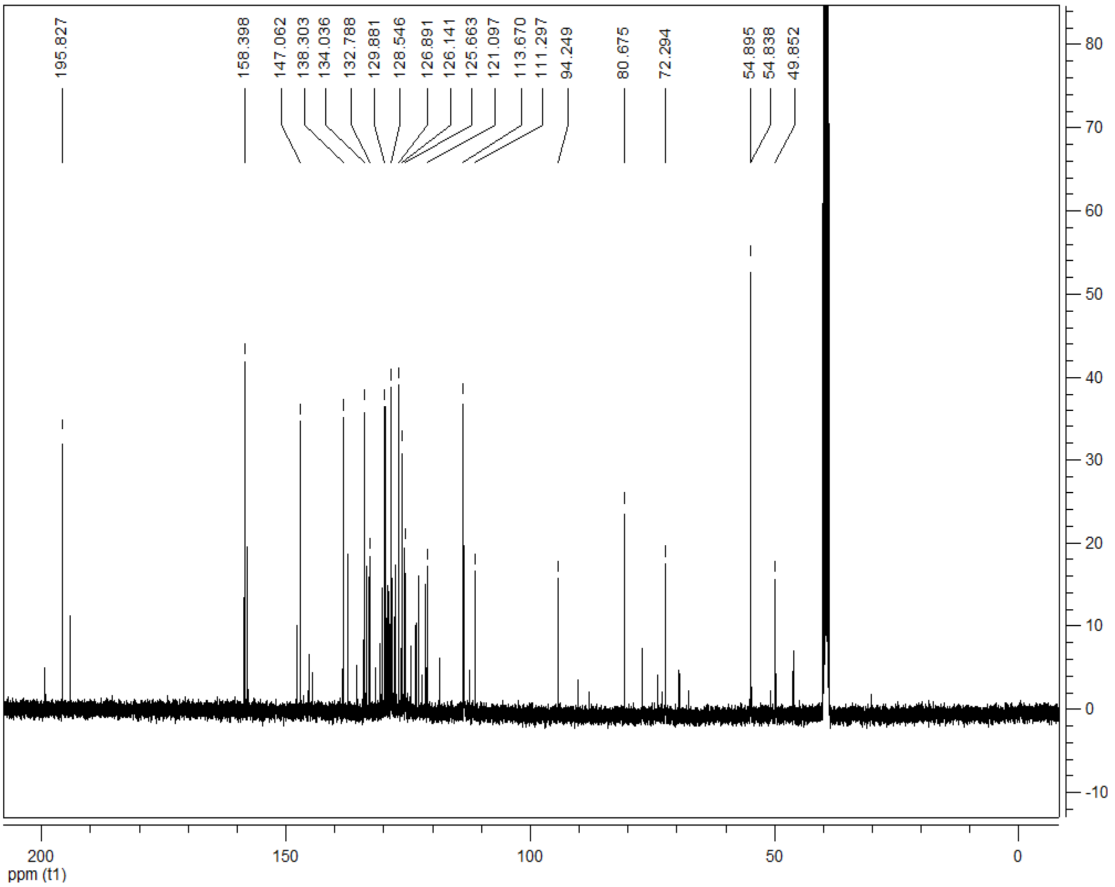
195.827
158.398
147.062
138.303
134.036
132.788
129.881
128.546
126.891
126.141
125.663
121.097
113.670
111.297
94.249
80.675
72.294
54.895
54.838
49.852
ppm (t1)

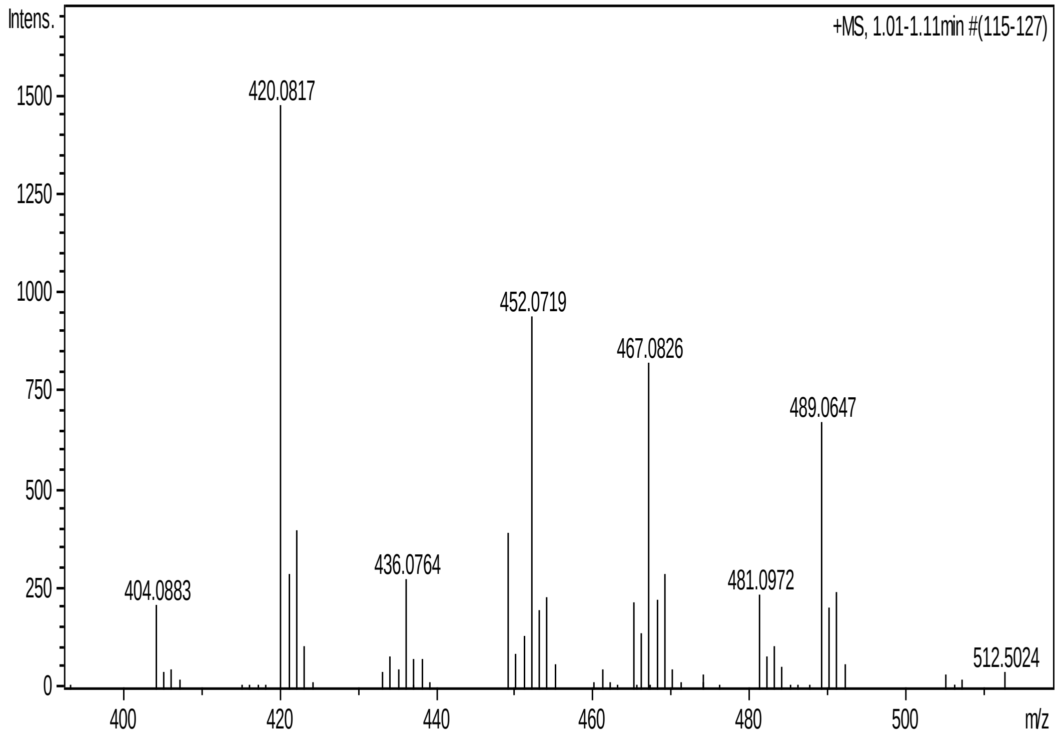
Intens.
+MS, 1.01-1.11min #(115-127)
420.0817
452.0719
467.0826
489.0647
404.0883
436.0764
481.0972
512.5024
m/z

(4-methoxyphenyl)(2-(4-methoxyphenyl)-3-nitro-1,2-dihydrobenzo[d]pyrrolo[2,1-b]thiazol-1-yl)methanone (2a): yellow solid, 24%, m.p. 230~232℃; ^{1}H NMR (600 MHz, $CDCl_3$) δ: 7.71 (d, *J* = 7.8 Hz, 2H, ArH), 7.63 (d, *J* = 7.8 Hz, 1H, ArH), 7.36~7.34 (m, 1H, ArH), 7.28~7.27 (m, 1H, ArH), 6.90~6.85 (m, 5H, ArH), 6.54~6.50 (m, 3H, ArH, CH), 5.46 (d, *J* = 10.8 Hz, 1H, CH), 3.88 (s, 3H, OCH_3) , 3.65 (s, 3H, OCH_3); ^{13}C NMR (100 MHz, DMSO-d_6) δ: 189.3, 163.6, 162.6, 158.1, 137.2, 130.8, 129.6, 129.2, 127.8, 127.7, 127.3, 124.3, 123.6, 118.5, 113.7, 113.2, 112.9, 69.5, 55.5, 54.8, 50.7; IR (KBr) υ: 3009, 2927, 2835, 1664, 1597, 1507, 1417, 1386, 1239, 1138, 1023, 975, 821, 759 cm^{-1}; MS (*m*/*z*): HRMS (ESI) Calcd. for $C_{25}H_{21}N_2O_5S$ ($[M+H]^+$): 461.1166. Found: 461.1165.

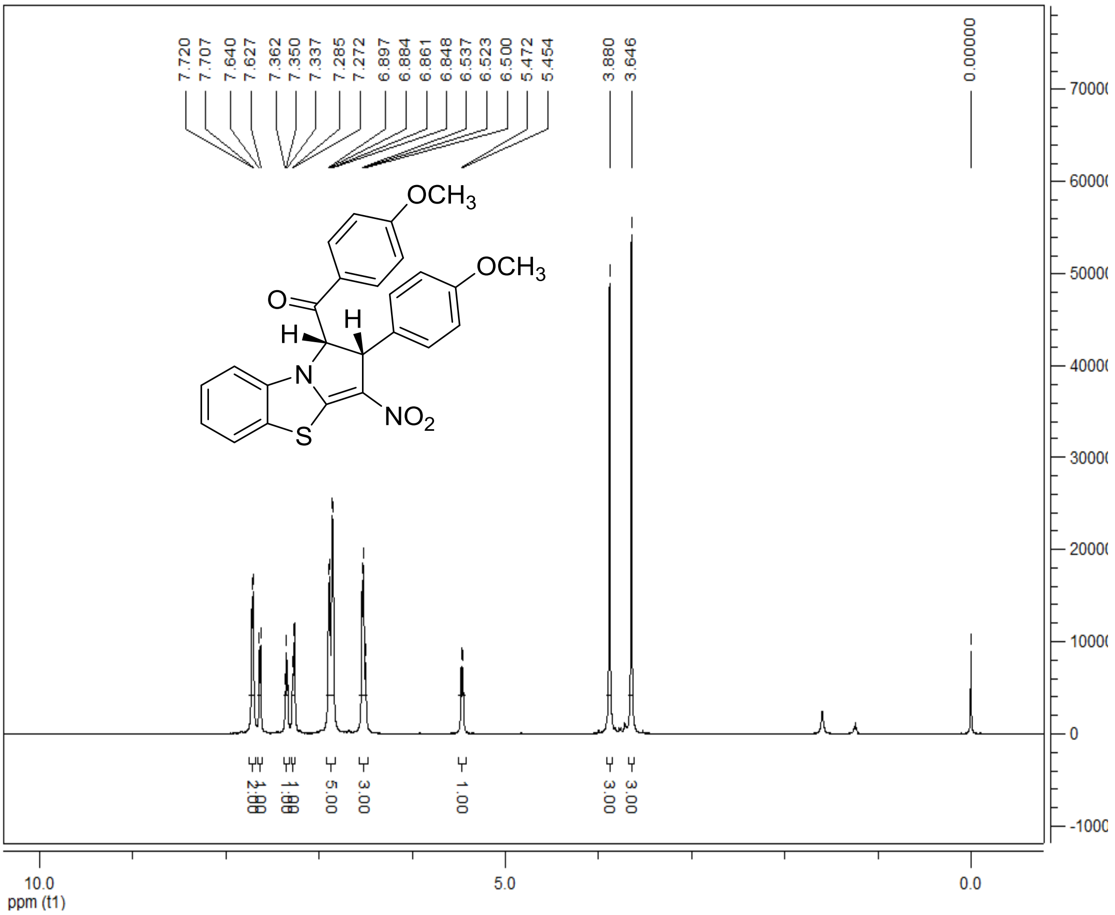

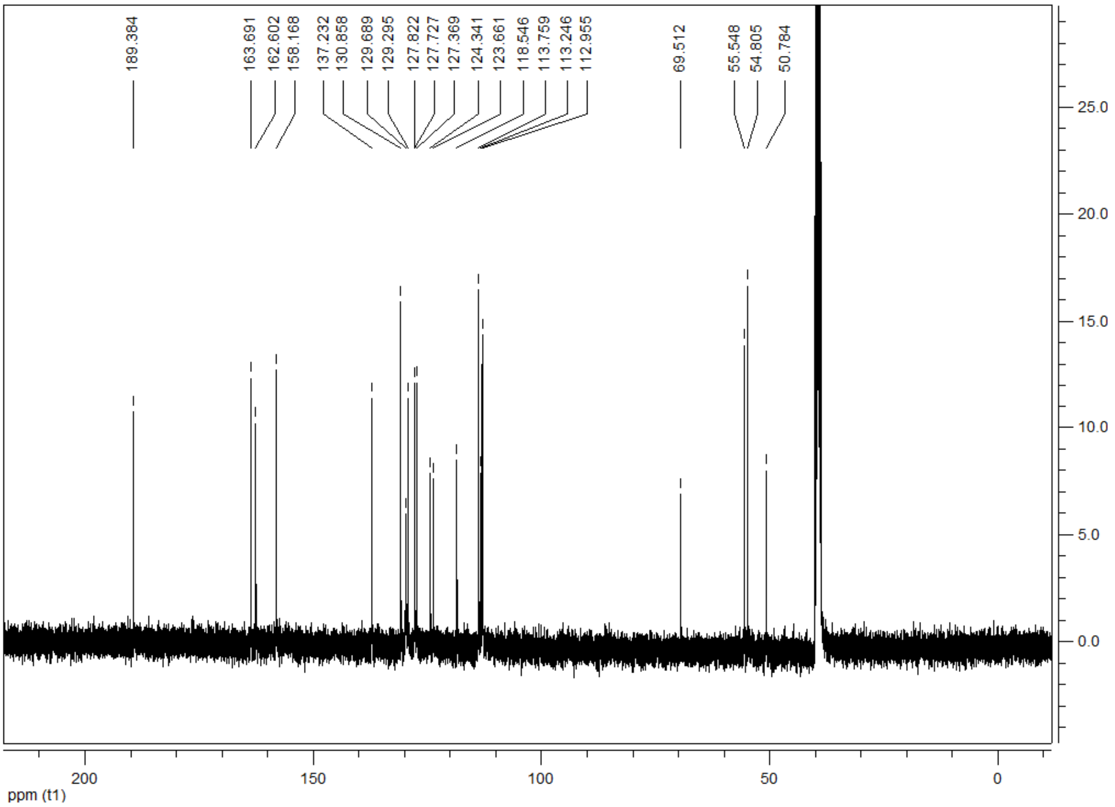
189.384
163.691
162.602
158.168
137.232
130.858
129.689
129.295
127.822
127.727
127.369
124.341
123.661
118.546
113.759
113.246
112.955
69.512
55.548
54.805
50.784
25.0
20.0
15.0
10.0
5.0
0.0
200
150
100
50
0
ppm (t1)

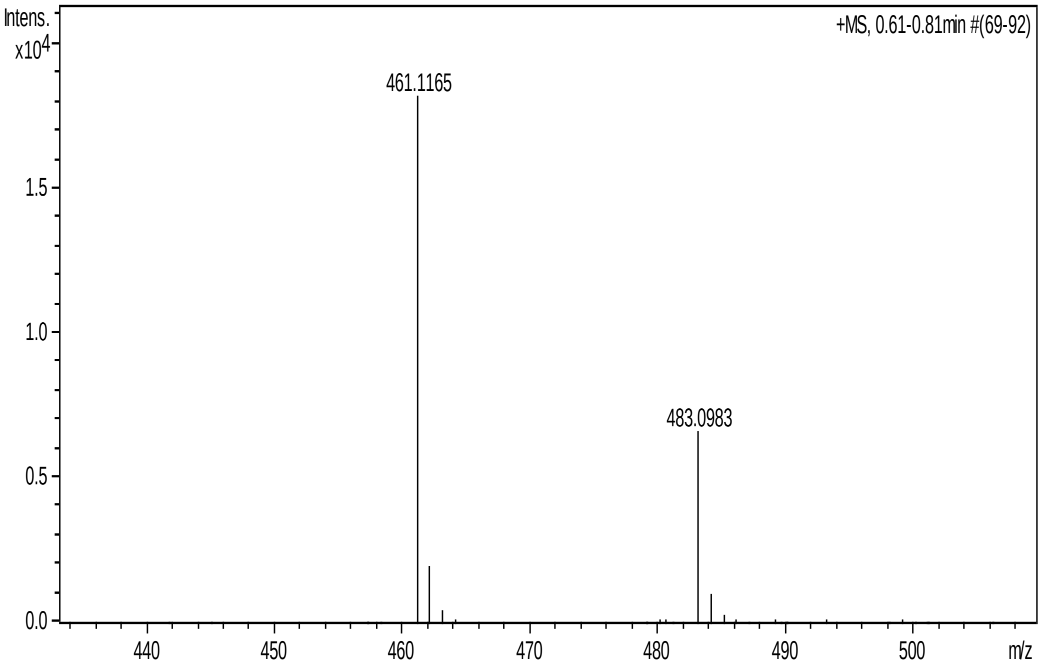
Intens.
x10^4
+MS, 0.61-0.81min #(69-92)
461.1165
483.0983
1.5
1.0
0.5
0.0
440
450
460
470
480
490
500
m/z

(4-methoxyphenyl)((1S,2R)-2-(4-methoxyphenyl)-3-nitro-1,2-dihydrobenzo[d]pyrrolo[2,1-b]thiazol-1-yl)methanone (2a'): yellow solid, 16%, m.p. 241~243℃; ^{1}H NMR (600 MHz, $CDCl_3$) δ: 7.83 (d, J = 7.8 Hz, 2H, ArH), 7.72 (d, J = 7.2 Hz, 1H, ArH), 7.37~7.35 (m, 1H, ArH), 7.29~7.28 (m, 2H, ArH), 7.25 (s, 1H, ArH), 6.98 (d, J = 7.8 Hz, 2H, ArH), 6.92 (d, J = 7.8 Hz, 2H, ArH), 6.85 (d, J = 7.2 Hz, 1H, ArH), 5.91 (s, 1H, CH), 4.83 (s, 1H, CH), 3.92 (s, 3H, OCH_3), 3.82 (s, 3H, OCH_3); ^{13}C NMR (150 MHz, $CDCl_3$) δ: 188.3, 165.0, 161.9, 159.7, 137.4, 131.7, 131.2, 130.0, 128.5, 128.0, 125.1, 123.9, 123.8, 119.2, 114.7, 114.5, 111.3, 72.4, 55.7, 55.3, 53.1; IR (KBr) υ: 3088, 2938, 2843, 1682, 1600, 1519, 1466, 1390, 1305, 1240, 1177, 1142, 1022, 830, 746 cm^{-1}; MS (m/z): HRMS (ESI) Calcd. for $C_{25}H_{20}N_2NaO_5S$ ($[M+Na]^+$): 483.0985. Found: 483.0988.

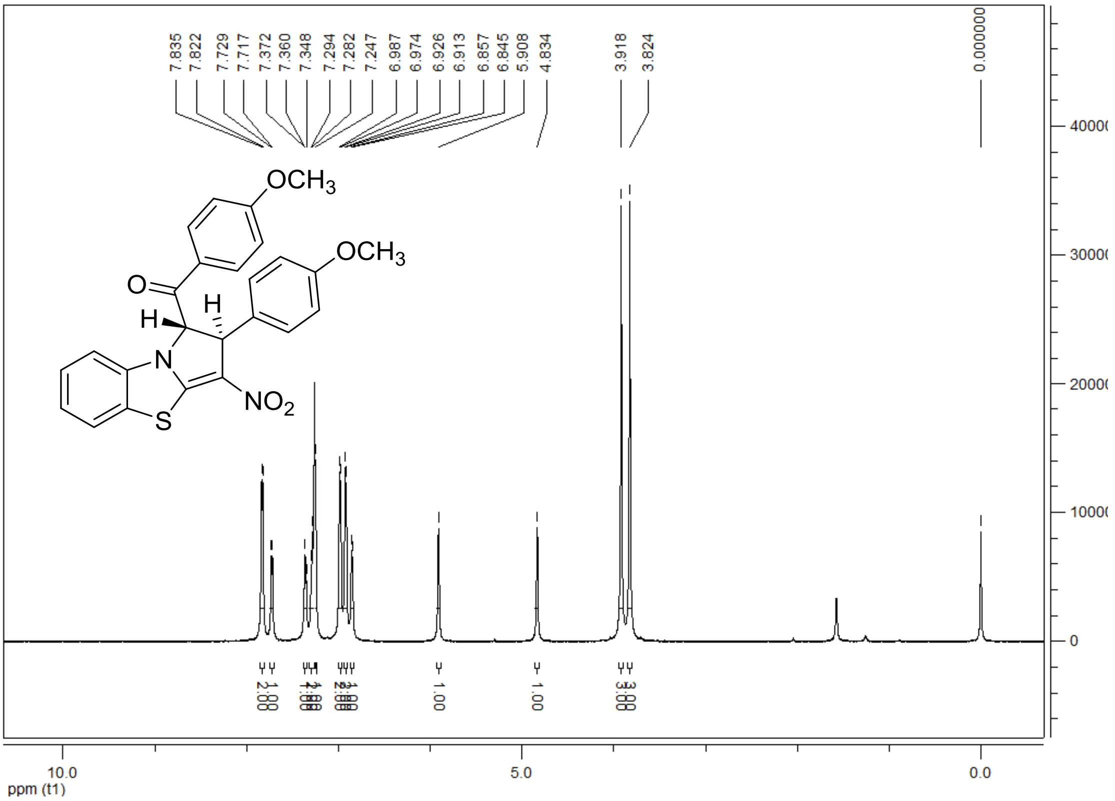

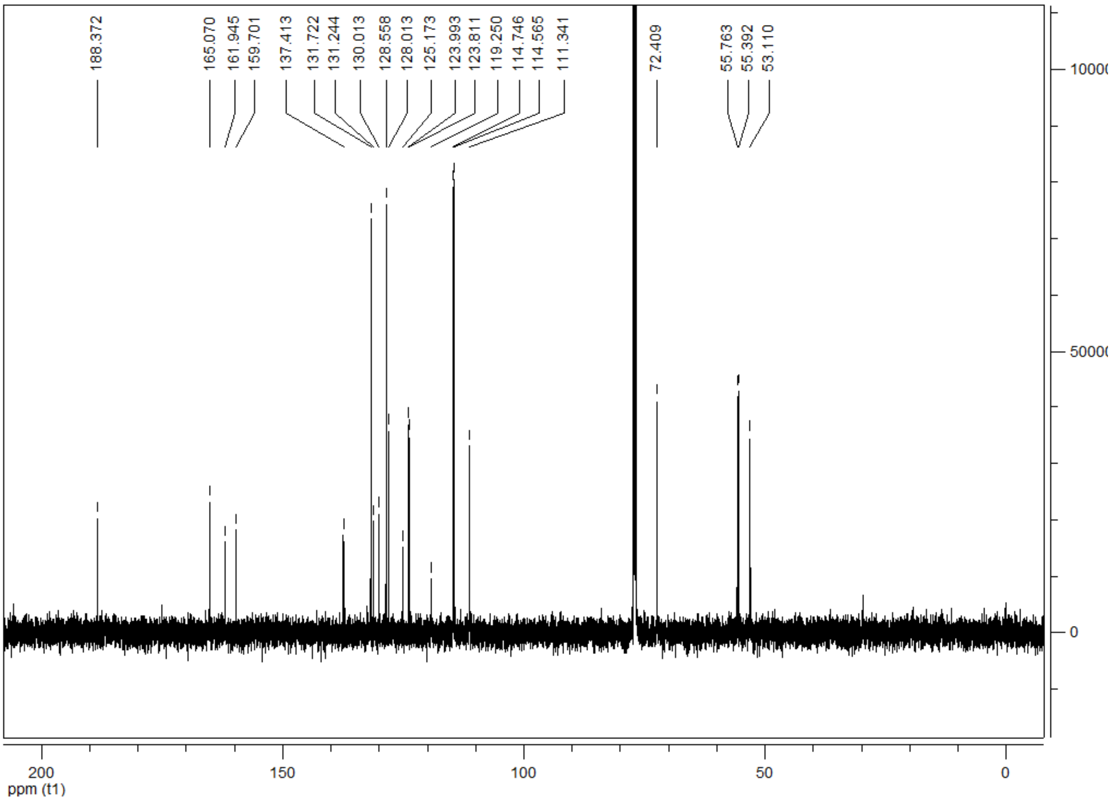
188.372
165.070
161.945
159.701
137.413
131.722
131.244
130.013
128.558
128.013
125.173
123.993
123.811
119.250
114.746
114.565
111.341
72.409
55.763
55.392
53.110
200
150
100
50
0
ppm (t1)

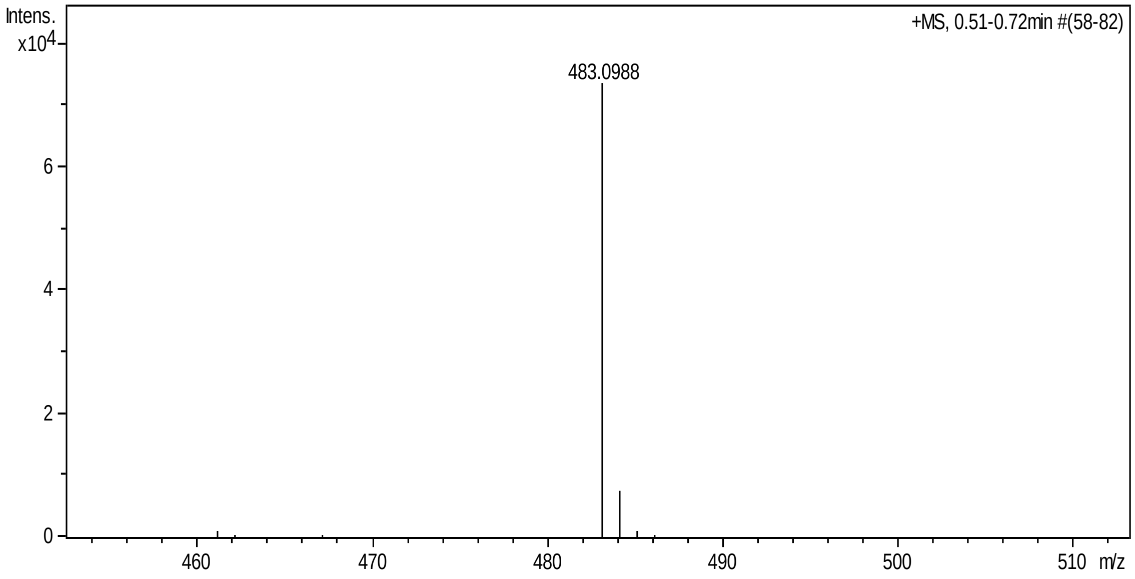
Intens.
x10^4
+MS, 0.51-0.72min #(58-82)
483.0988
460
470
480
490
500
510
m/z

3-nitro-2-(p-tolyl)-1,2-dihydrobenzo[d]pyrrolo[2,1-b]thiazol-1-yl)(phenyl)methanone (2b): yellow solid, 28%, m.p. 208~210℃; ^{1}H NMR (600 MHz, $CDCl_3$) δ: 7.69~7.65 (m, 3H, ArH), 7.60~7.57 (m, 1H, ArH), 7.42~7.40 (m, 2H, ArH), 7.37~7.34 (m, 1H, ArH), 7.30~7.27 (m, 1H, ArH), 6.84 (d, J = 7.8 Hz, 1H, ArH), 6.81~6.77 (m, 4H, ArH), 6.54 (d, J = 10.7 Hz, 1H, CH), 5.50 (d, J = 10.7 Hz, 1H, CH), 2.14 (s, 3H, CH_3); ^{13}C NMR (100 MHz, $CDCl_3$) δ: 190.5, 162.6, 137.6, 137.5, 135.1, 134.2, 131.2, 130.1, 128.8, 128.7, 128.7, 128.1, 127.9, 123.9, 123.7, 112.8, 70.2, 52.4, 20.9; IR (KBr) υ: 3064, 1692, 1650, 1586, 1537, 1458, 1401, 1343, 1297, 1233, 1145, 1104, 1028, 982, 880, 816, 741 cm^{-1}; MS (m/z): HRMS (ESI) Calcd. for $C_{24}H_{19}N_2O_3S$ ($[M+H]^+$): 415.1111. Found: 415.1113.

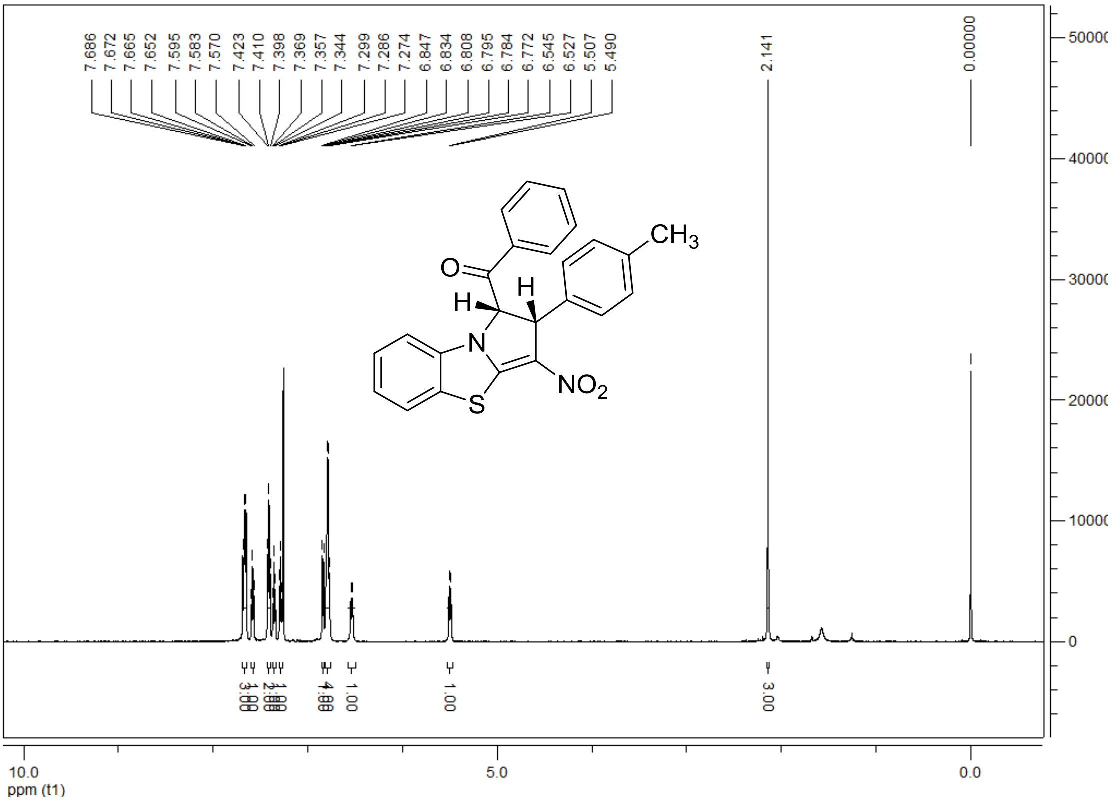

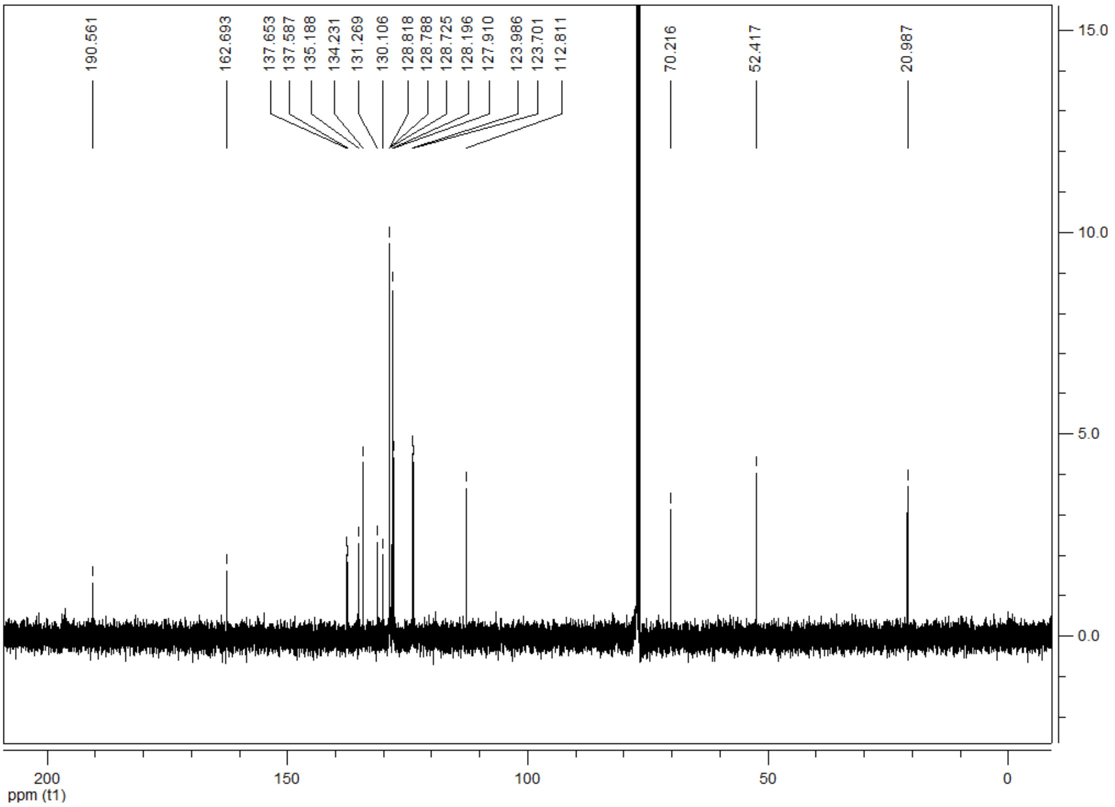
190.561
162.693
137.653
137.587
135.188
134.231
131.269
130.106
128.818
128.788
128.725
128.196
127.910
123.986
123.701
112.811
70.216
52.417
20.987
15.0
10.0
5.0
0.0
200
150
100
50
0
ppm (t1)

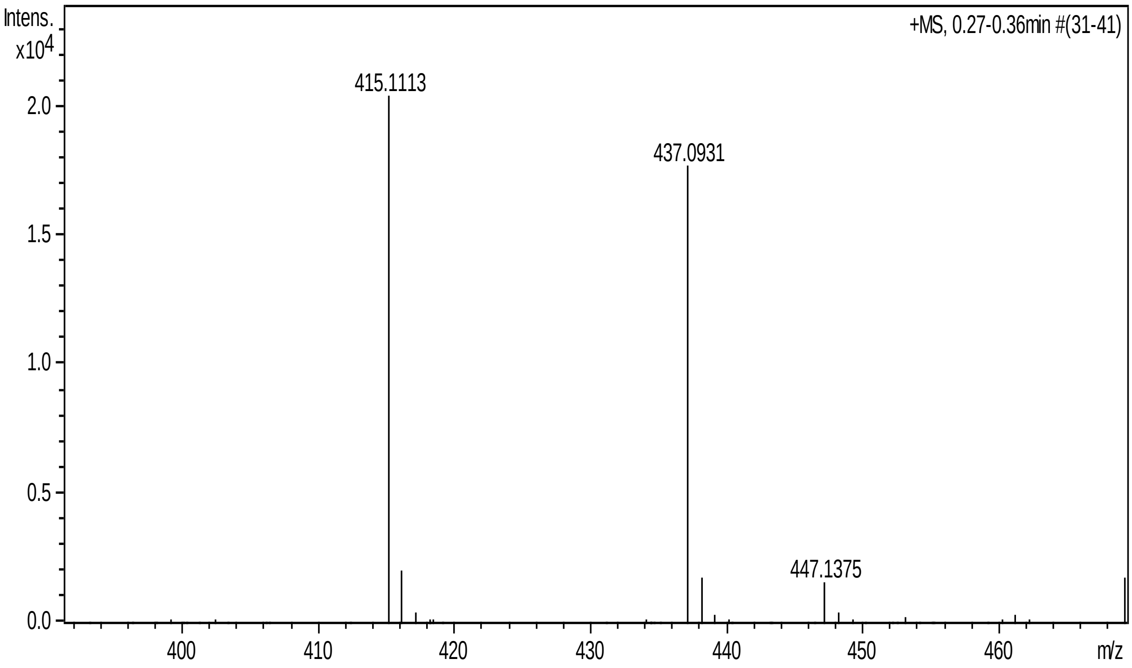
Intens.
x10^4
+MS, 0.27-0.36min #(31-41)
415.1113
437.0931
447.1375
2.0
1.5
1.0
0.5
0.0
400
410
420
430
440
450
460
m/z

3-nitro-2-(p-tolyl)-1,2-dihydrobenzo[d]pyrrolo[2,1-b]thiazol-1-yl)(phenyl)methanone (2b'): yellow solid, 15%, m.p. 265~267℃; ^{1}H NMR (400 MHz, DMSO-*d*$_6$) δ: 8.09 (d, *J* = 8.0 Hz, 1H, ArH), 7.90 (d, *J* = 7.2 Hz, 2H, ArH), 7.80~7.77 (m, 1H, ArH), 7.64~7.60 (m, 2H, ArH), 7.50~7.46 (m, 1H, ArH), 7.39~7.35 (m, 2H, ArH), 7.23~7.17 (m, 4H, ArH), 6.79 (s, 1H, CH), 4.70 (s, 1H, CH), 2.33 (s, 3H, CH_3); ^{13}C NMR (150 MHz, DMSO-*d*$_6$) δ: 191.3, 162.2, 137.2, 136.0, 134.7, 132.2, 129.4, 129.3, 129.2, 128.9, 127.9, 127.1, 124.5, 123.7, 118.3, 112.6, 71.9, 51.8, 20.5; IR (KBr) υ: 2957, 1686, 1589, 1518, 1461, 1419, 1382, 1301, 1229, 1184, 1139, 1015, 930, 829, 747 cm^{-1}; MS (*m*/*z*): HRMS (ESI) Calcd. for $C_{24}H_{18}N_2NaO_3S$ ([M+Na]$^+$): 437.0930. Found: 437.0934.

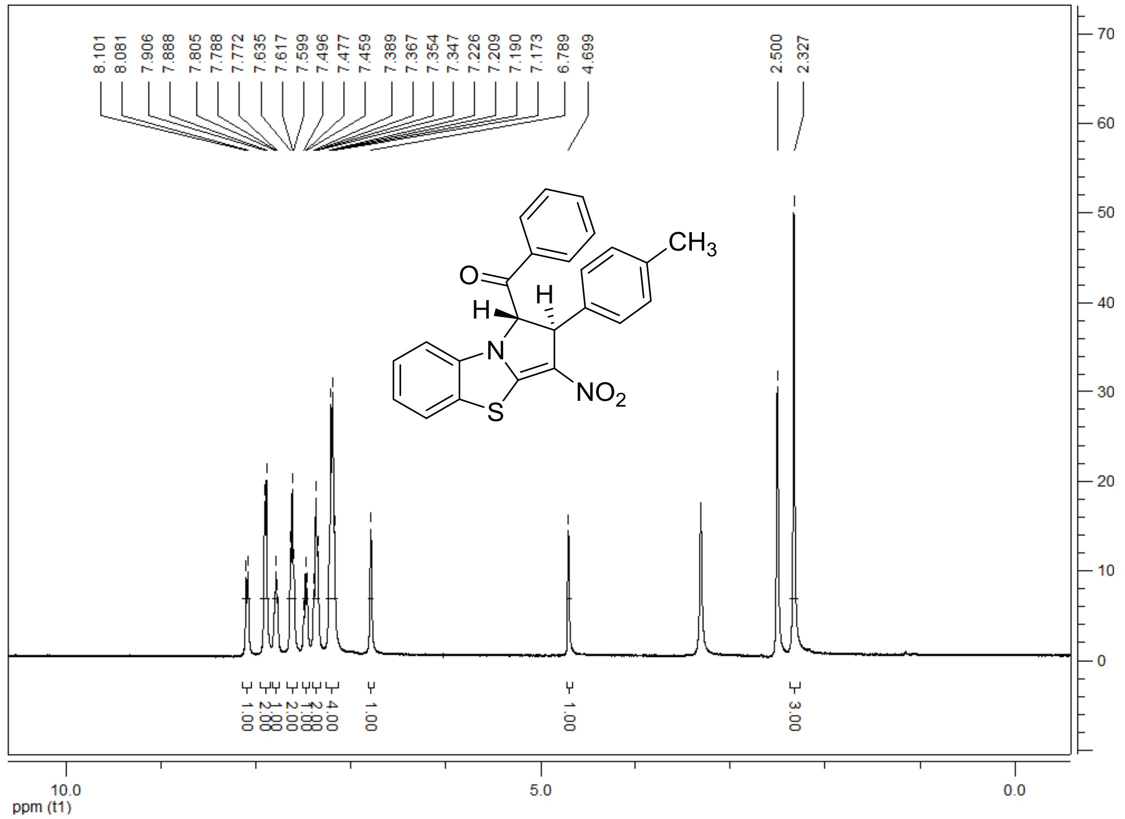

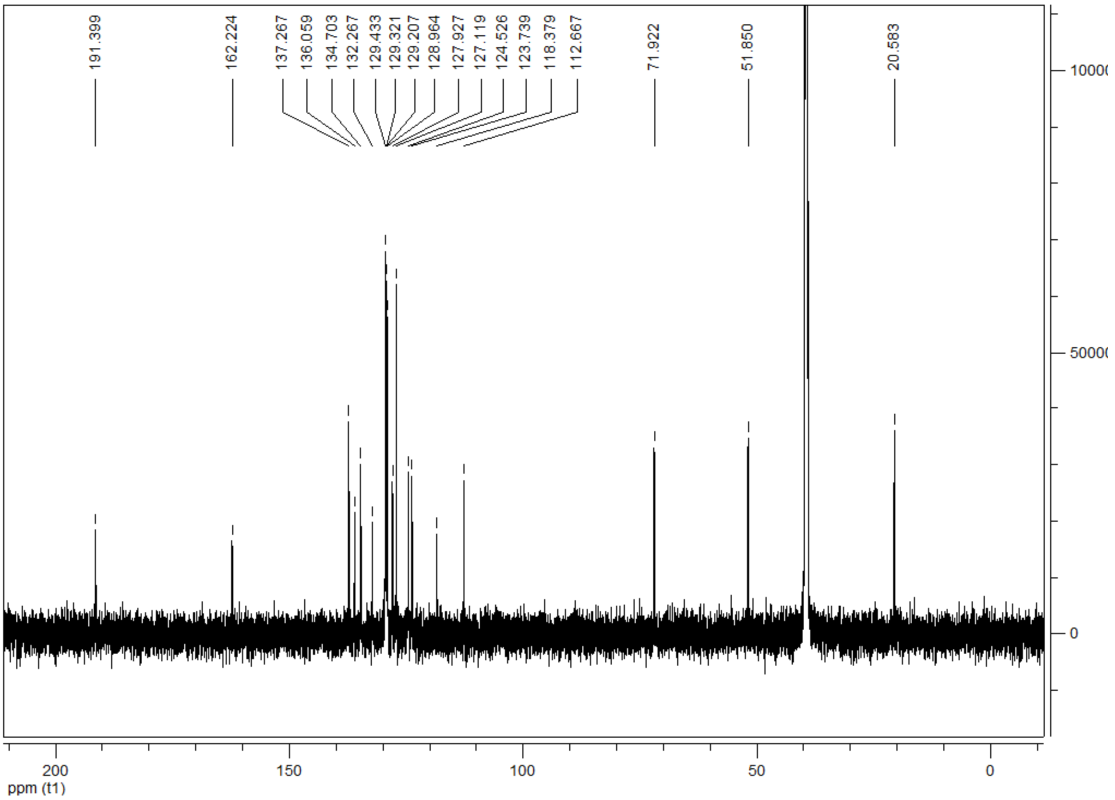
191.399
162.224
137.267
136.059
134.703
132.267
129.433
129.321
129.207
128.964
127.927
127.119
124.526
123.739
118.379
112.667
71.922
51.850
20.583
ppm (t1)

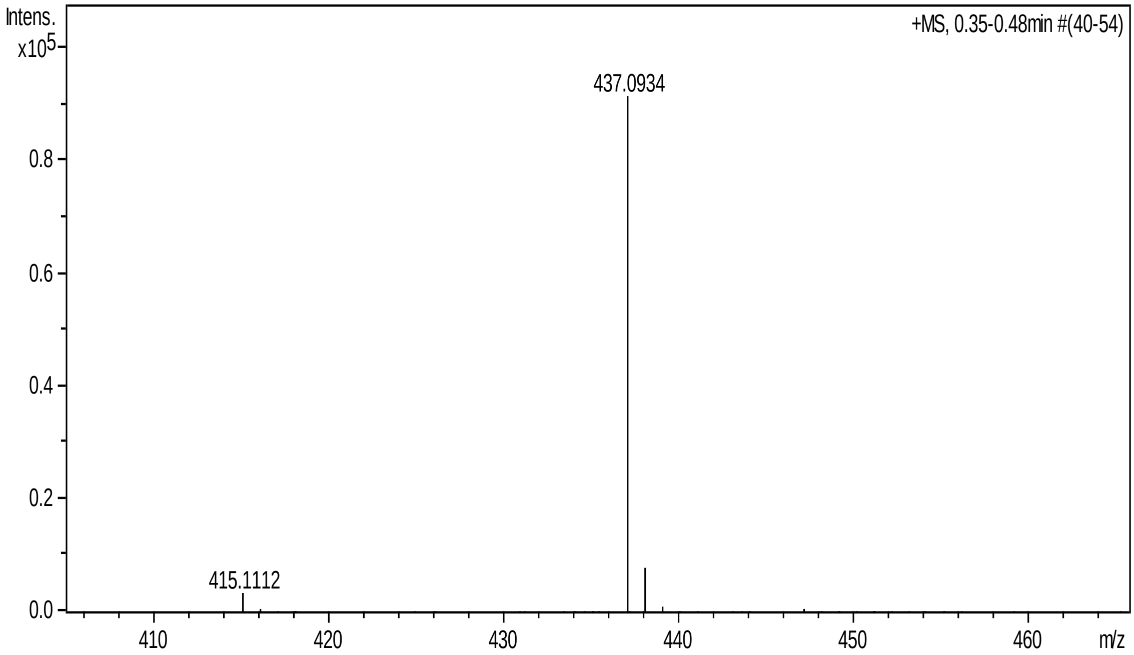
Intens.
x10^5
+MS, 0.35-0.48min #(40-54)
437.0934
415.1112
m/z

(2-(3-methoxyphenyl)-3-nitro-1,2-dihydrobenzo[d]pyrrolo[2,1-b]thiazol-1-yl)(p-tolyl)methanone (2c'): yellow solid, 14%, m.p. 235~237℃; ^{1}H NMR (400 MHz, DMSO-d_6) δ: 8.09 (d, J = 8.0 Hz, 1H, ArH), 7.82 (d, J = 8.0 Hz, 2H, ArH), 7.49~7.42 (m, 3H, ArH), 7.39~7.29 (m, 3H, ArH), 6.96 (d, J = 7.9 Hz, 1H, ArH), 6.86 (d, J = 7.4 Hz, 1H, ArH), 6.82~6.79 (m, 2H, ArH, CH), 4.70 (brs, 1H, CH), 3.75 (s, 3H, OCH_3), 2.44 (s, 3H, CH_3); ^{13}C NMR (150 MHz, DMSO-d_6) δ: 191.0, 162.5, 159.5, 145.7, 140.6, 137.3, 130.2, 129.8, 129.6, 129.4, 128.0, 124.6, 123.8, 119.3, 118.1, 113.3, 113.1, 112.7, 71.6, 55.0, 52.2, 21.3; IR (KBr) υ: 3083, 2946, 2830, 1679, 1603, 1529, 1424, 1380, 1302, 1235, 1187, 1142, 1091, 1050, 1015, 985, 870, 811, 781, 749, 710 cm^{-1}; MS (m/z): HRMS (ESI) Calcd. for $C_{25}H_{20}N_2NaO_4S$([M+Na]$^+$): 467.1036. Found: 467.1044.

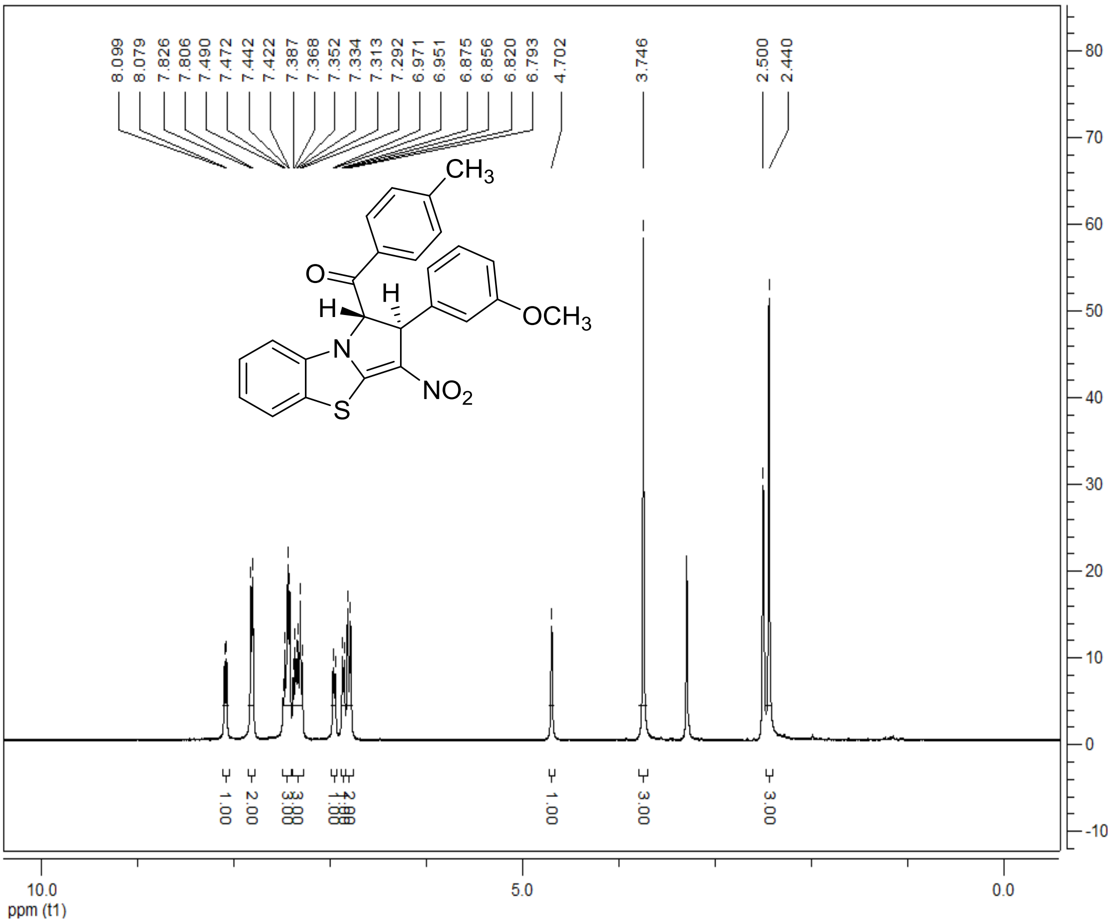

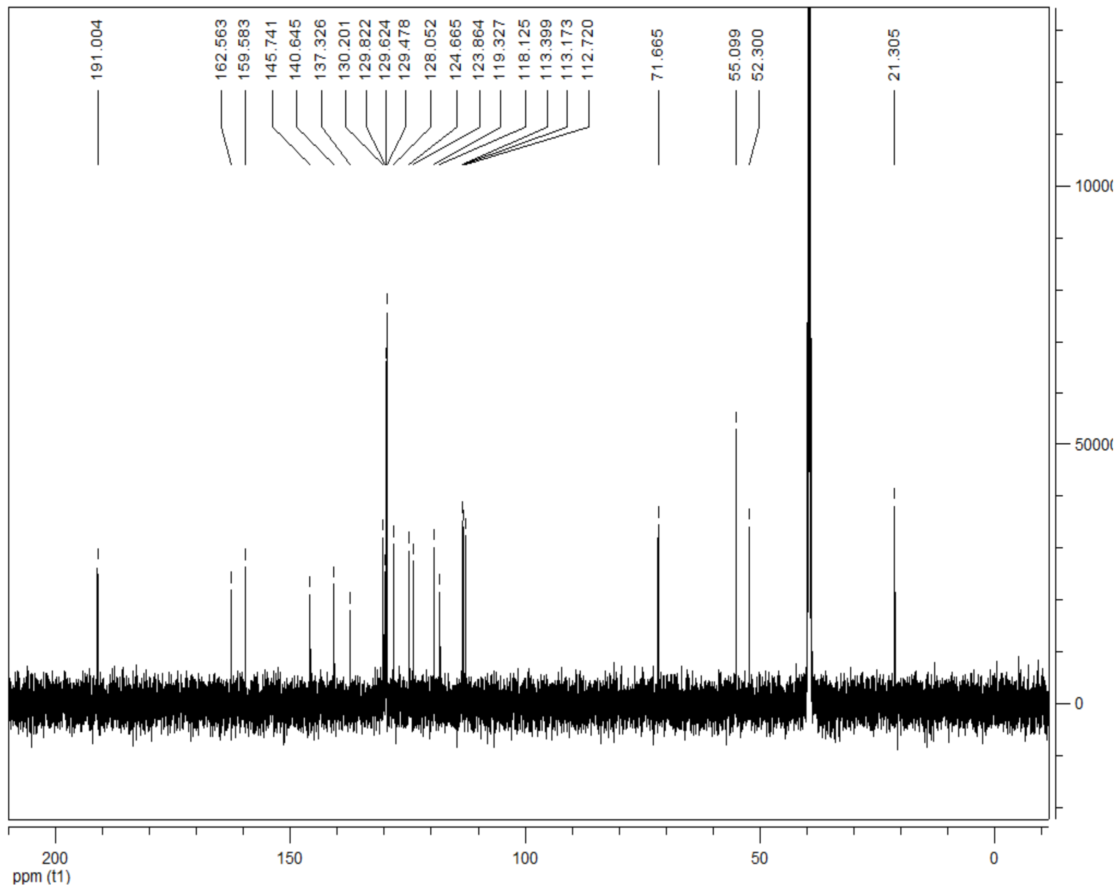
191.004
162.563
159.583
145.741
140.645
137.326
130.201
129.822
129.624
129.478
128.052
124.665
123.864
119.327
118.125
113.399
113.173
112.720
71.665
55.099
52.300
21.305
ppm (t1)

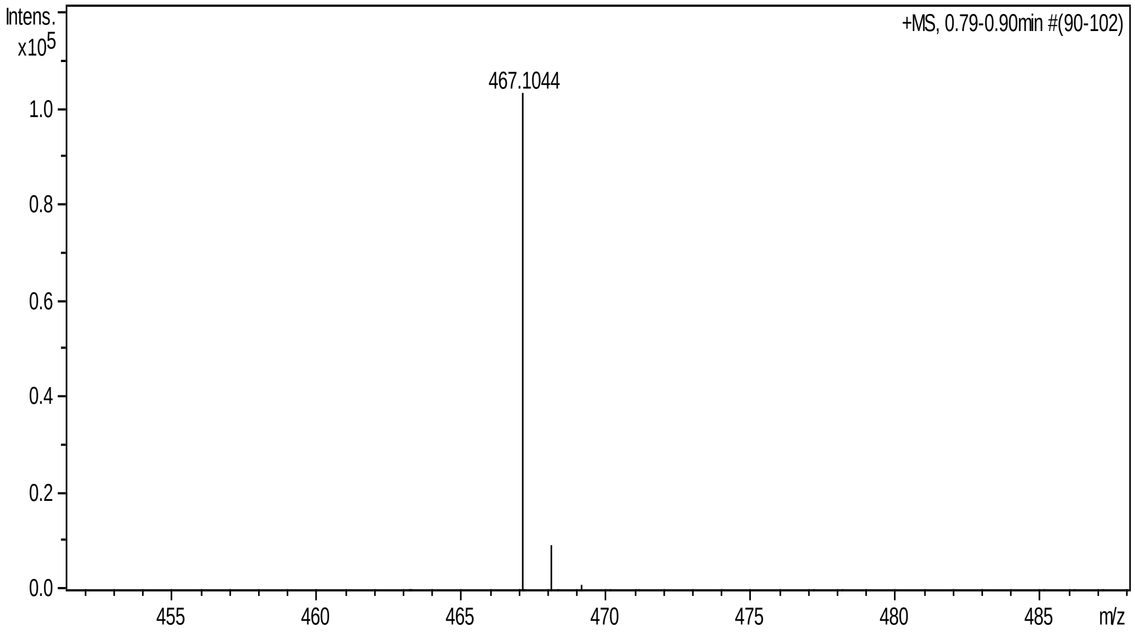
Intens.
x10^5
+MS, 0.79-0.90min #(90-102)
467.1044
m/z

(2-(4-chlorophenyl)-3-nitro-1,2-dihydrobenzo[d]pyrrolo[2,1-b]thiazol-1-yl)(phenyl)methanone (2d'): yellow solid, 15%, m.p. 249~251℃; ^{1}H NMR (400 MHz, DMSO-d_6) δ: 8.10 (d, *J* = 6.8 Hz, 1H, ArH), 7.88 (d, *J* = 6.4 Hz, 2H, ArH), 7.81~7.77 (m, 1H, ArH), 7.66~7.57 (m, 2H, ArH), 7.52~7.44 (m, 3H, ArH), 7.39~7.21 (m, 4H, ArH), 6.84 (s, 1H, CH), 4.81 (s, 1H, CH); ^{13}C NMR (100 MHz, DMSO-d_6) δ: 191.1, 162.3, 137.9, 137.1, 134.7, 132.5, 132.2, 129.3, 129.1, 128.9, 128.7, 127.9, 124.5, 123.7, 117.9, 112.6, 71.4, 51.3; IR (KBr) υ: 2962, 1681, 1639, 1523, 1419, 1382, 1300, 1230, 1181, 1139, 1092, 1013, 927, 823, 750 cm^{-1}; MS (*m*/*z*): HRMS (ESI) Calcd. for $C_{23}H_{15}ClN_2NaO_3S$ ([M+Na]$^+$): 457.0384. Found: 457.0382.

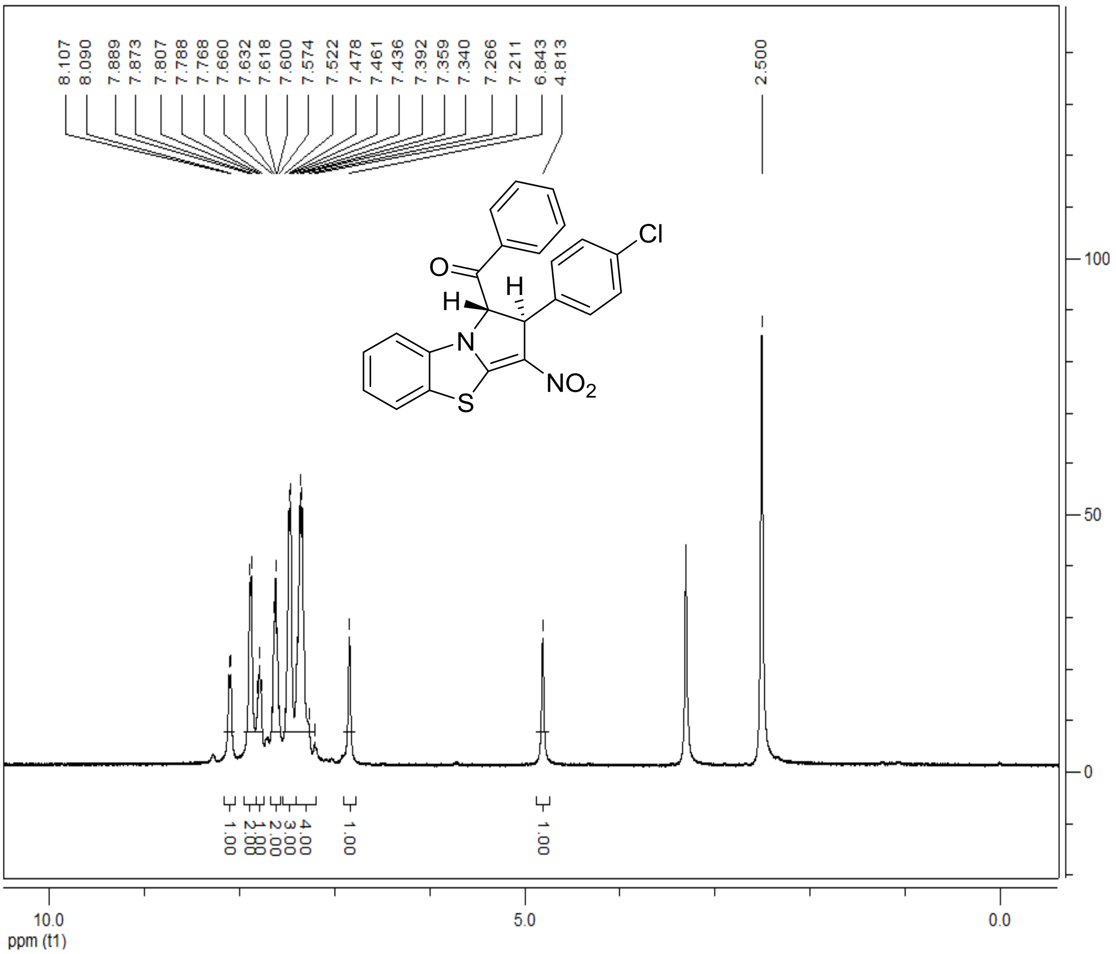

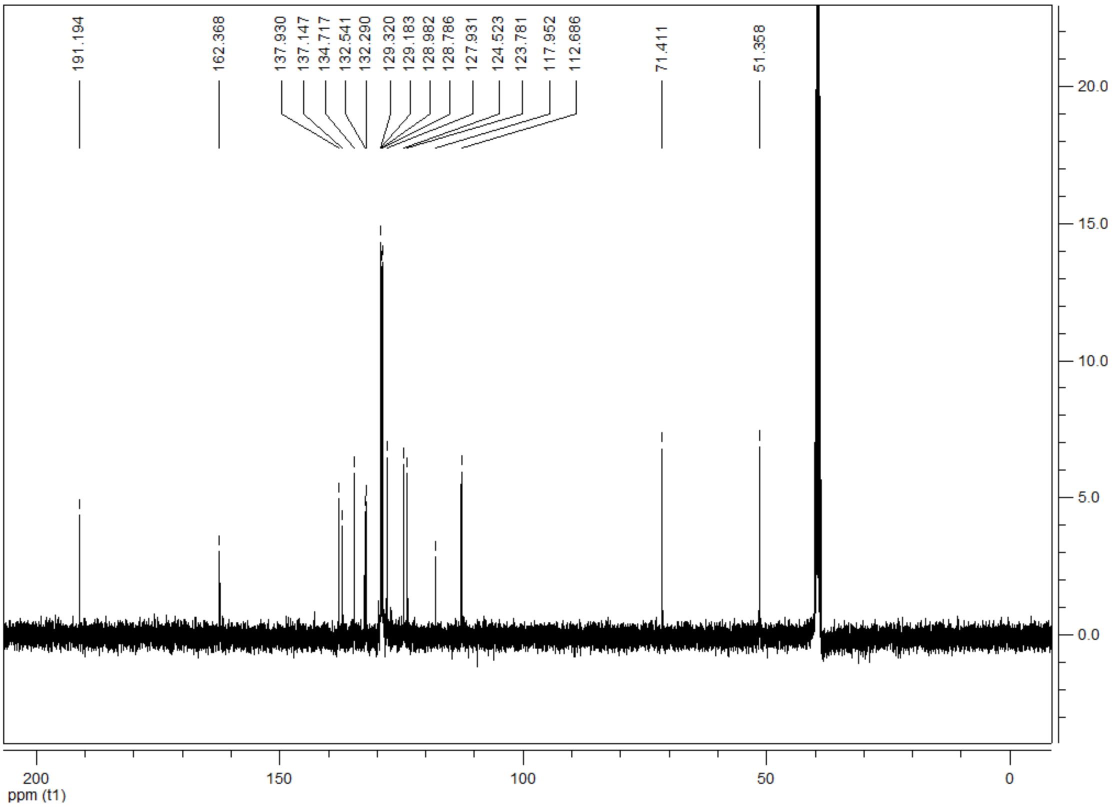
191.194
162.368
137.930
137.147
134.717
132.541
132.290
129.320
129.183
128.982
128.786
127.931
124.523
123.781
117.952
112.686
71.411
51.358
20.0
15.0
10.0
5.0
0.0
200
150
100
50
0
ppm (t1)

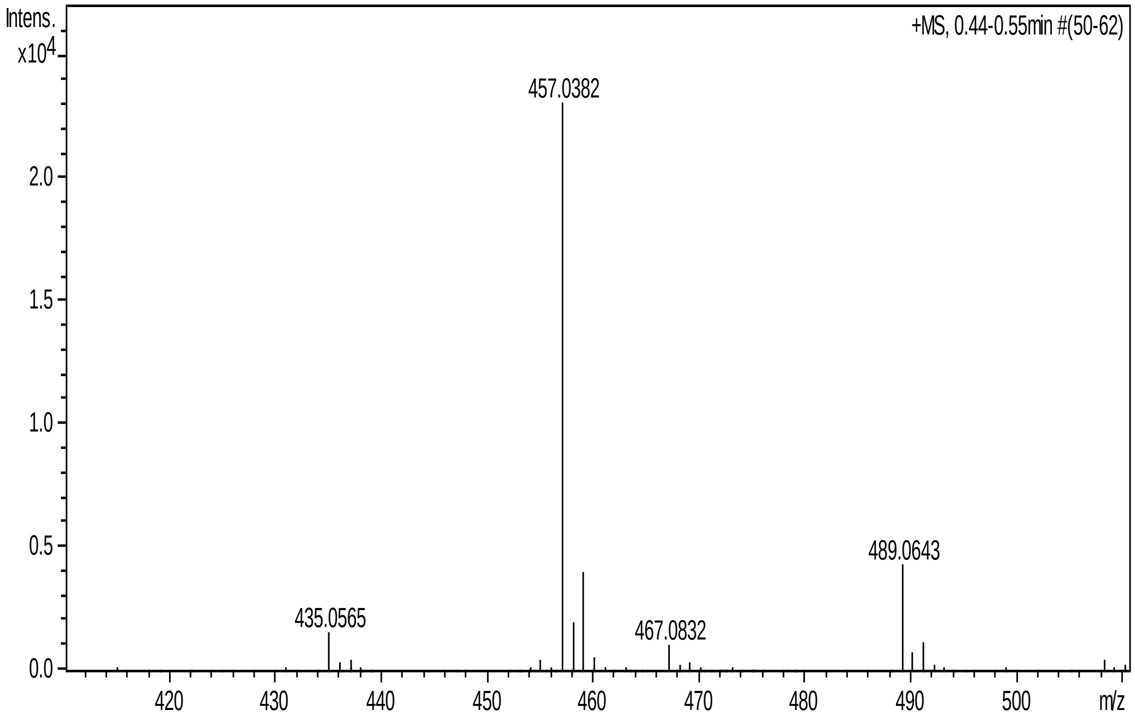
Intens.
x10^4
+MS, 0.44-0.55min #(50-62)
457.0382
489.0643
435.0565
467.0832
2.0
1.5
1.0
0.5
0.0
420
430
440
450
460
470
480
490
500
m/z

(4-Fluorophenyl)(2-(4-methoxyphenyl)-3-nitro-1,2-dihydrobenzo[d]pyrrolo[2,1-b]thiazol-1-yl)methanone (2e'): yellow solid, 20%, m.p. 260~262℃; ^{1}H NMR (600 MHz, DMSO-d_6) δ: 8.09 (d, J = 8.4 Hz, 1H, ArH), 7.97~7.95 (m, 2H, ArH), 7.49~7.47 (m, 3H, ArH), 7.38~7.34 (m, 2H, ArH), 7.23 (d, J = 8.4 Hz, 2H, ArH), 6.96 (d, J = 8.4 Hz, 2H, ArH), 6.78 (d, J = 3.0 Hz, 1H, CH), 4.72 (d, J = 3.0 Hz, 1H, CH), 3.77 (s, 3H, OCH_3); ^{13}C NMR (100 MHz, DMSO-d_6) δ: 190.0, 162.0, 158.9, 137.2, 132.3, 132.2, 130.9, 129.2, 128.4, 127.9, 124.5, 123.7, 118.4, 116.2, 116.0, 114.2, 112.6, 71.8, 55.0, 51.4; IR (KBr) υ: 3084, 2962, 1688, 1601, 1519, 1469, 1386, 1338, 1308, 1238, 1175, 1145, 1022, 984, 814, 745 cm^{-1}; MS (m/z): HRMS (ESI) Calcd. for $C_{24}H_{17}FN_2NaO_4S$ ($[M+Na]^+$): 471.0785. Found: 471.0784.

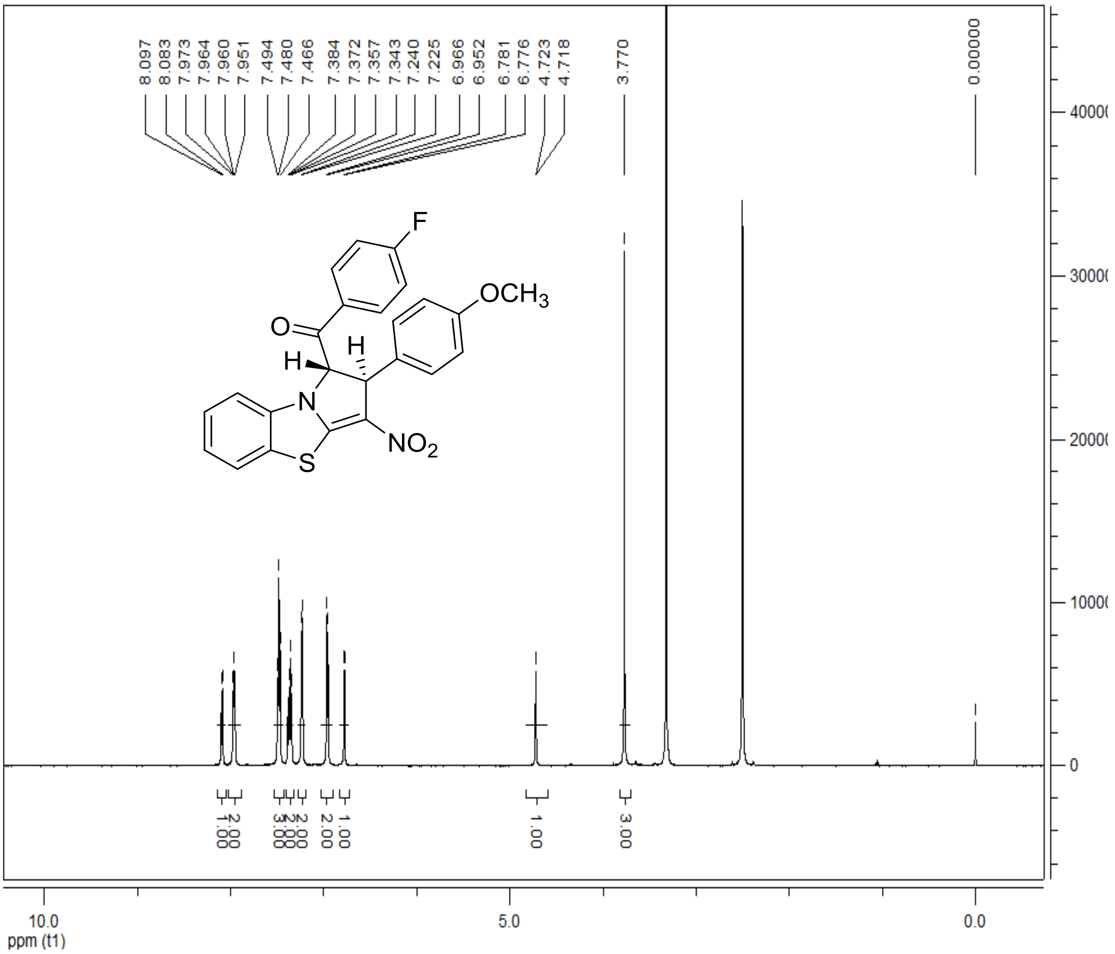

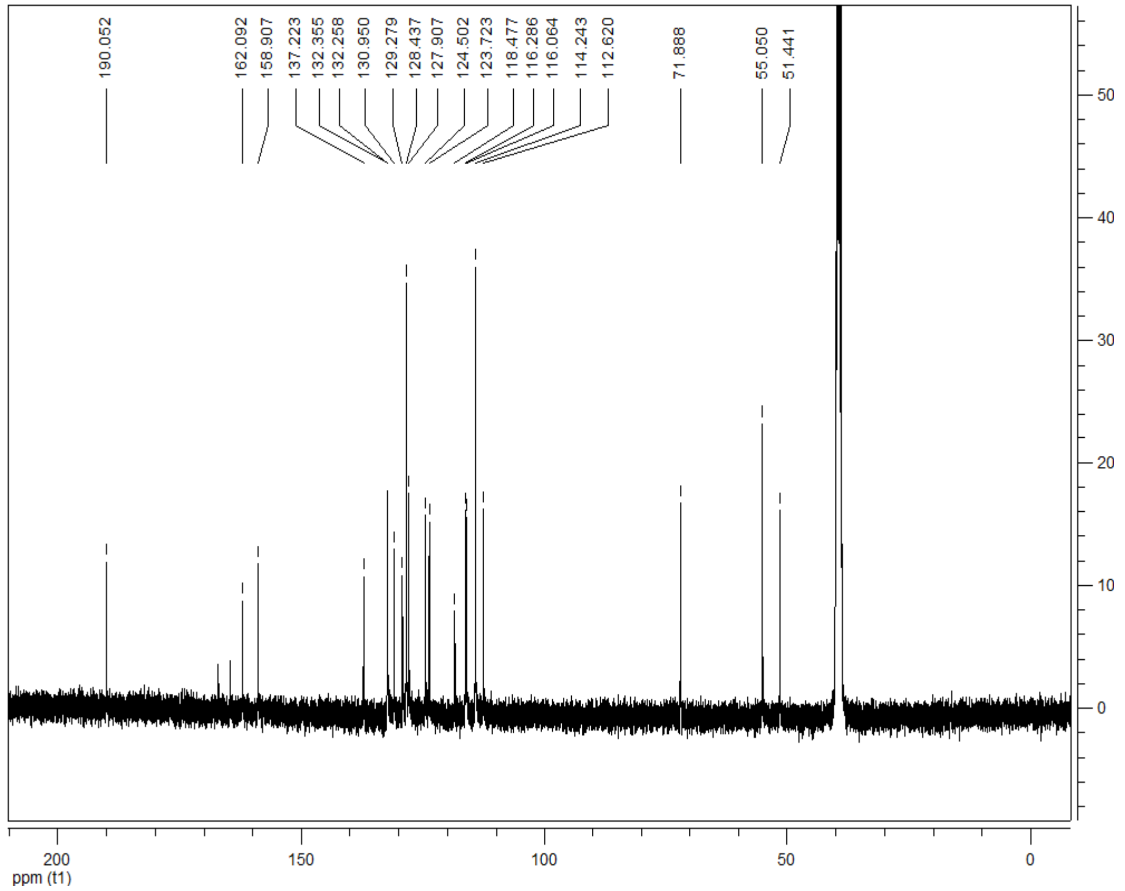
190.052
162.092
158.907
137.223
132.355
132.258
130.950
129.279
128.437
127.907
124.502
123.723
118.477
116.286
116.064
114.243
112.620
71.888
55.050
51.441
50
40
30
20
10
0
200
150
100
50
0
ppm (t1)

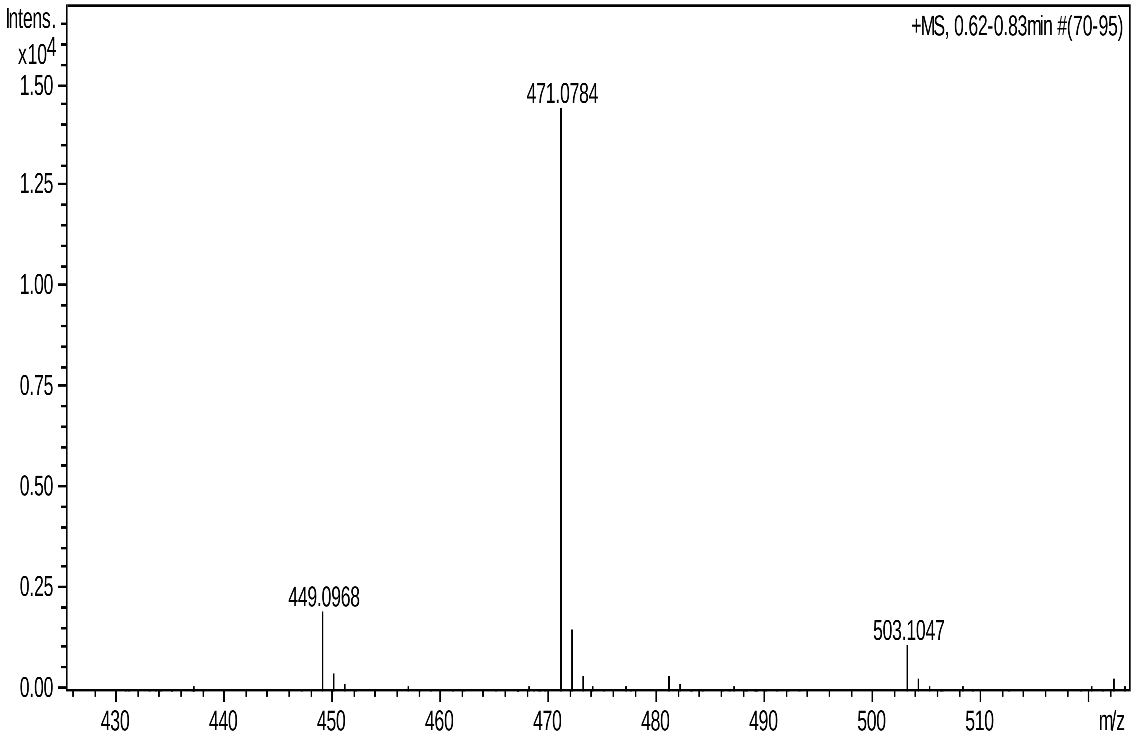
Intens.
x10^4
1.50
1.25
1.00
0.75
0.50
0.25
0.00
+MS, 0.62-0.83min #(70-95)
471.0784
449.0968
503.1047
430
440
450
460
470
480
490
500
510
m/z

(3-nitro-2-phenylbenzo[d]pyrrolo[2,1-b]thiazol-1-yl)(p-tolyl)methanone (3a):

yellow solid, 90%, m.p. 195~197℃; ^{1}H NMR (400 MHz, DMSO-d_6) δ: 8.26~8.22 (m, 1H, ArH), 7.71~7.69 (m, 1H, ArH), 7.63 (d, J = 7.6 Hz, 2H, ArH), 7.58~7.52 (m, 2H, ArH), 7.26 (brs, 2H, ArH), 7.21~7.17 (m, 3H, ArH), 7.07 (d, J = 7.6 Hz, 2H, ArH), 2.24 (s, 3H, CH_3); ^{13}C NMR (150 MHz, DMSO-d_6) δ: 186.5, 144.2, 139.5, 134.1, 133.7, 130.6, 130.5, 130.0, 130.0, 128.8, 128.7, 127.9, 127.3, 127.1, 126.1, 125.2, 125.1, 124.7, 115.9, 21.1; IR (KBr) υ: 3059, 1731, 1635, 1599, 1538, 1501, 1394, 1339, 1273, 1160, 1045, 1009, 915, 832, 730 cm^{-1}; MS (m/z): HRMS (ESI) Calcd. for $C_{24}H_{15}ClN_2NaO_4S$ ($[M+Na]^+$): 485.0333. Found: 485.0332.

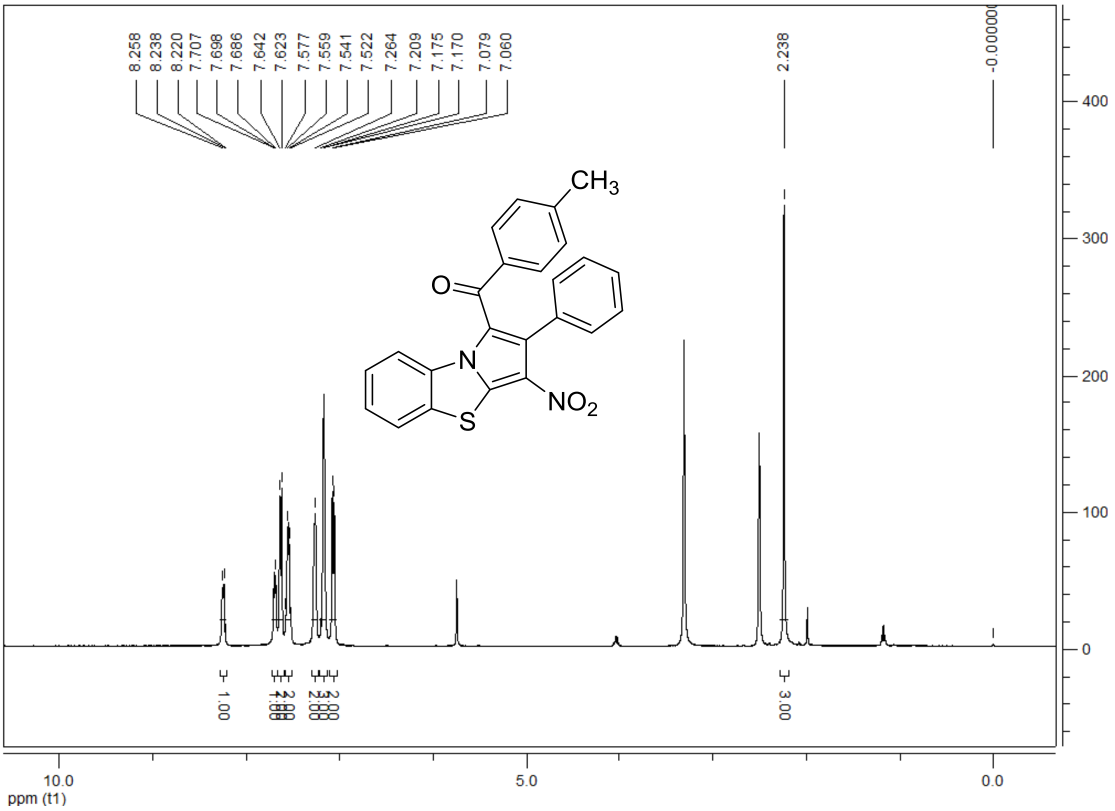

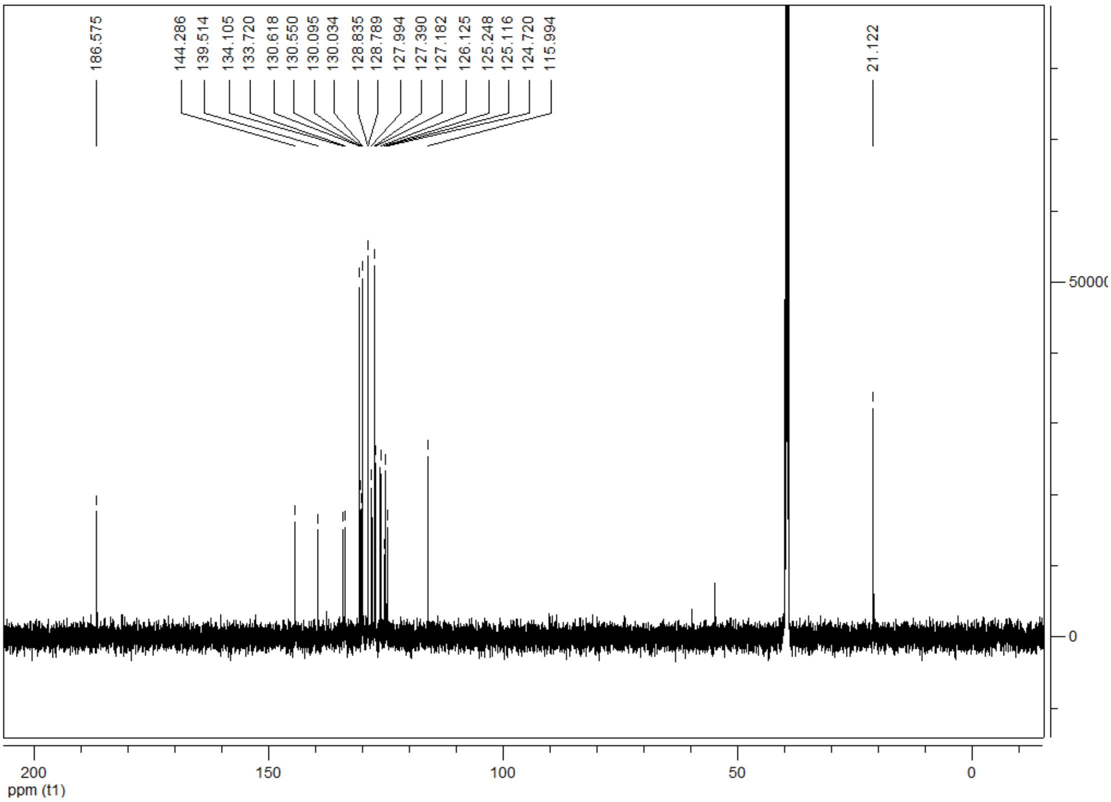
186.575
144.286
139.514
134.105
133.720
130.618
130.550
130.095
130.034
128.835
128.789
127.994
127.390
127.182
126.125
125.248
125.116
124.720
115.994
21.122
50000
0
200
150
100
50
0
ppm (t1)

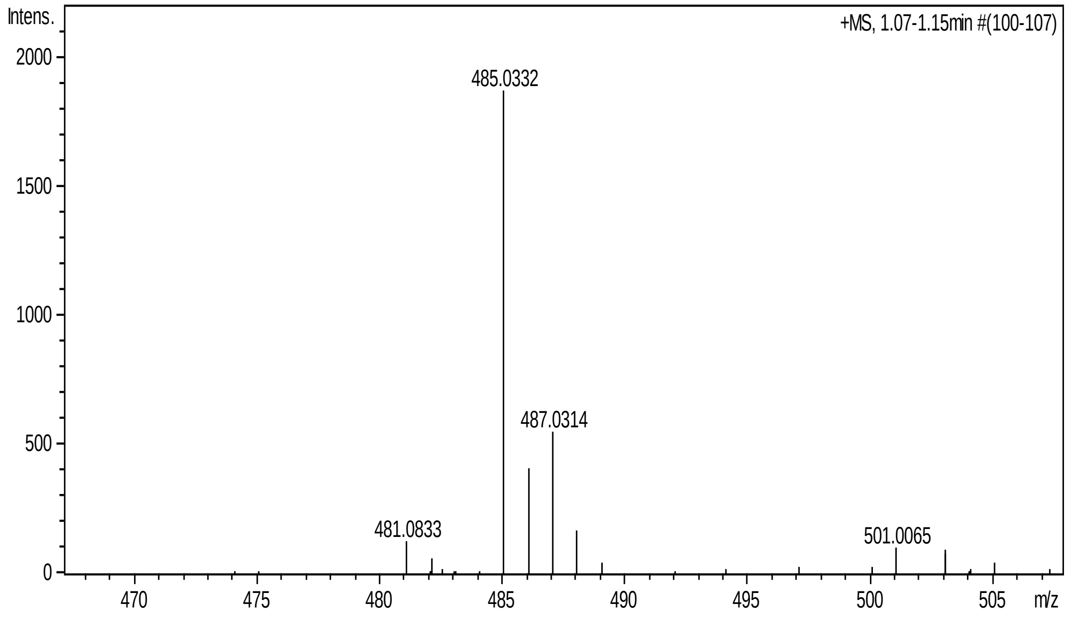
Intens.
+MS, 1.07-1.15min #(100-107)
2000
1500
1000
500
0
485.0332
487.0314
481.0833
501.0065
470
475
480
485
490
495
500
505
m/z

(2-(4-methoxyphenyl)-3-nitrobenzo[d]pyrrolo[2,1-b]thiazol-1-yl)(p-tolyl)methanone (3b): yellow solid, 88%, m.p. 167~169℃; ^{1}H NMR (400 MHz, DMSO-d_6) δ: 8.26~8.24 (m, 1H, ArH), 7.70 ~7.68 (m, 1H, ArH), 7.63 (d, J = 8.0 Hz, 2H, ArH), 7.57~7.52 (m, 2H, ArH), 7.19 (d, J = 8.0 Hz, 2H, ArH), 7.10 (d, J = 8.0 Hz, 2H, ArH), 6.73 (d, J = 8.4 Hz, 2H, ArH), 3.67 (s, 3H, OCH_3), 2.26 (s, 3H, CH_3); ^{13}C NMR (150 MHz, DMSO-d_6) δ: 186.7, 159.0, 144.2, 139.5, 134.1, 133.7, 131.9, 130.5, 130.0, 128.9, 128.6, 127.2, 126.0, 125.2, 125.1, 124.5, 122.0, 115.9, 112.9, 55.0, 21.1; IR (KBr) υ: 2926, 1615, 1513, 1472, 1390, 1342, 1282, 1250, 1168, 1019, 916, 838, 748, 708 cm^{-1}; MS (m/z): HRMS (ESI) Calcd. for $C_{25}H_{18}N_2NaO_4S$ ($[M+Na]^+$): 465.0879. Found: 465.0879.

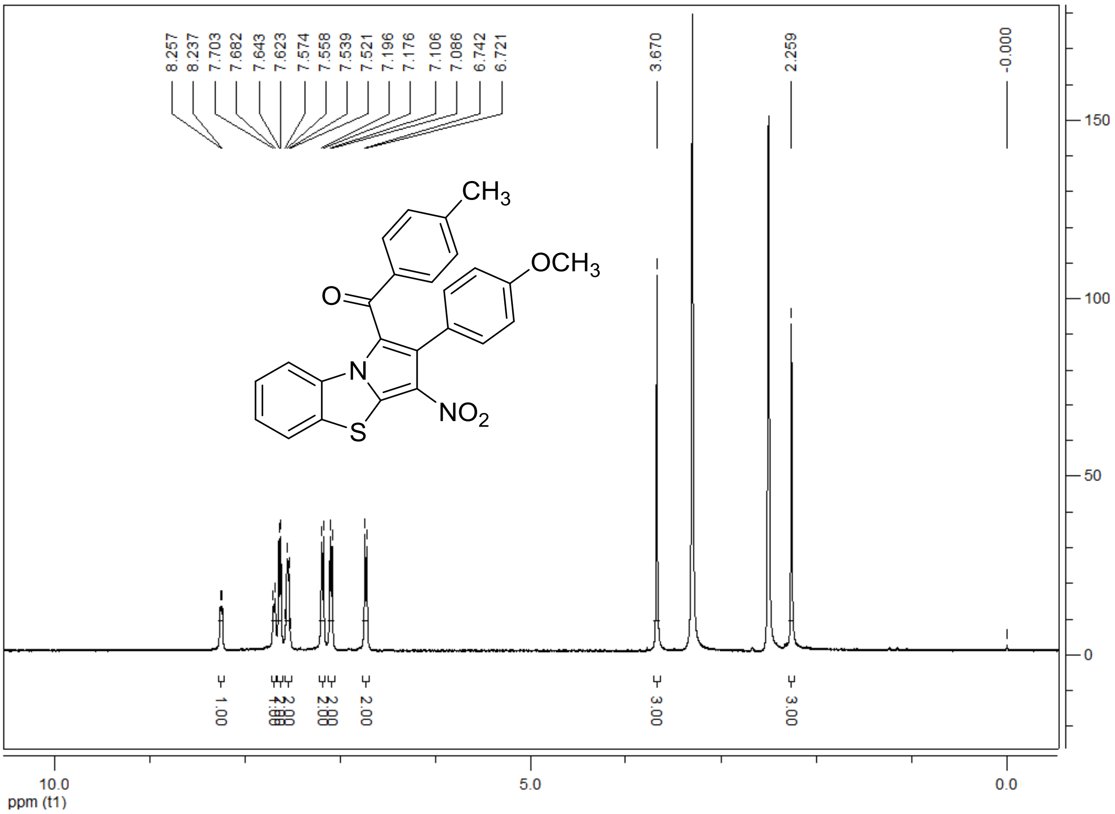

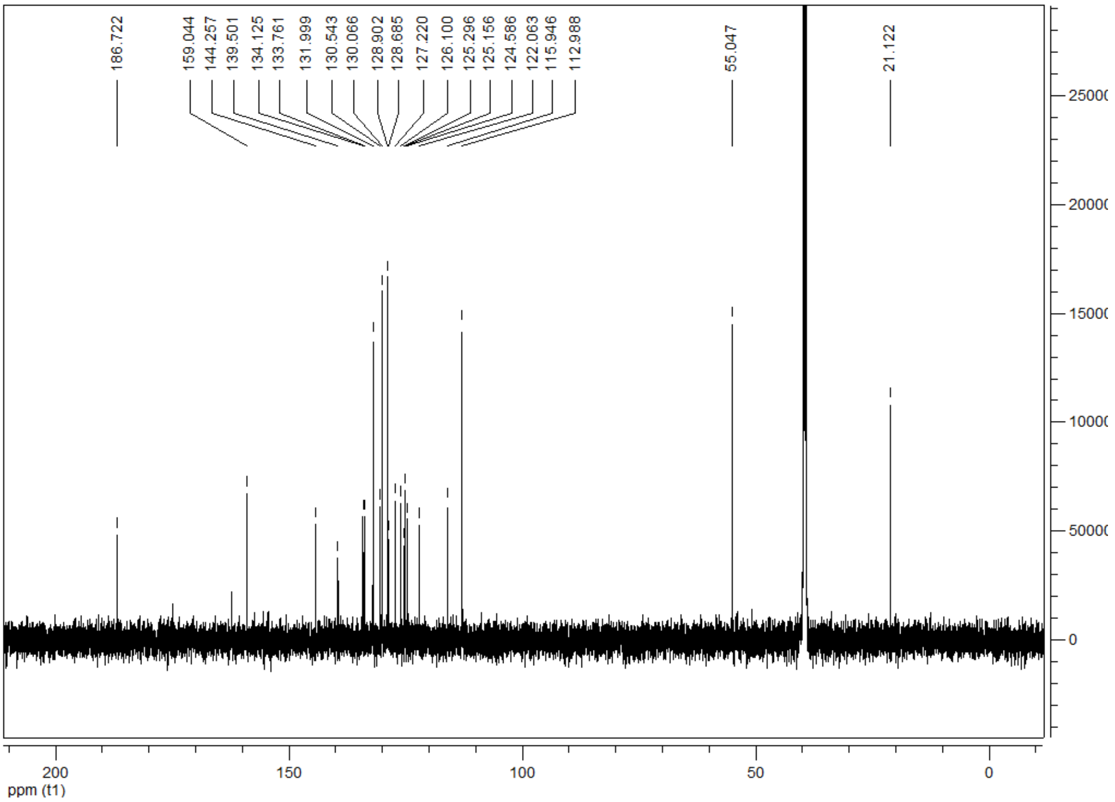
186.722
159.044
144.257
139.501
134.125
133.761
131.999
130.543
130.066
128.902
128.685
127.220
126.100
125.296
125.156
124.586
122.063
115.946
112.988
55.047
21.122
ppm (t1)

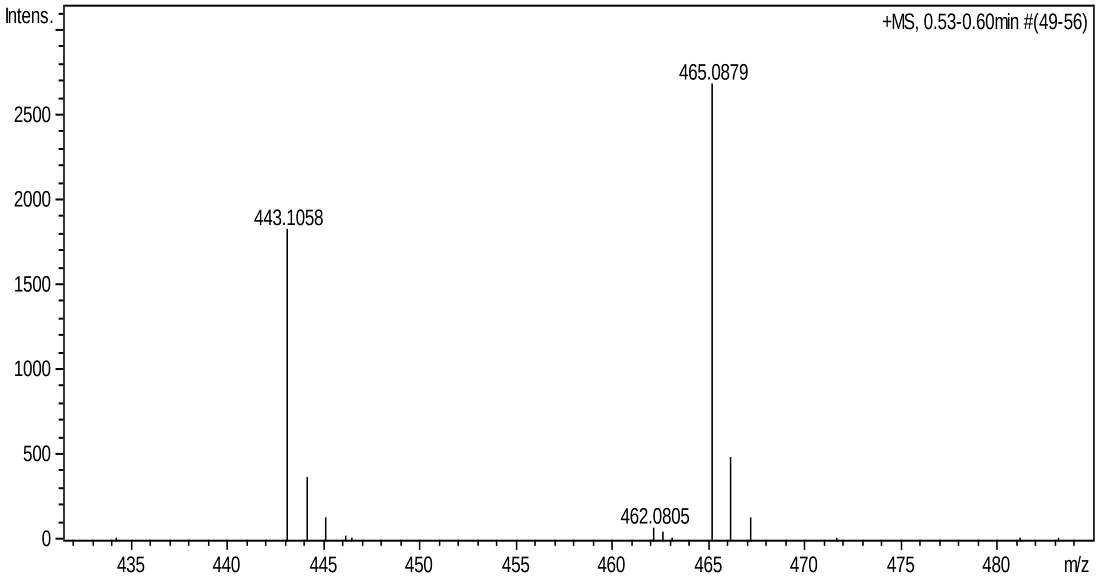
Intens.
+MS, 0.53-0.60min #(49-56)
465.0879
443.1058
462.0805
m/z

(2-(3-methoxyphenyl)-3-nitrobenzo[d]pyrrolo[2,1-b]thiazol-1-yl)(p-tolyl)methanone (3c): yellow solid, 89%, m.p. 180~182℃; ^{1}H NMR (400 MHz, DMSO-d_6) δ: 8.27~8.24 (m, 1H, ArH), 7.71~7.69 (m, 1H, ArH), 7.65 (d, J = 8.0 Hz, 2H, ArH), 7.58~7.53 (m, 2H, ArH), 7.10~7.06 (m, 3H, ArH), 6.86~6.80 (m, 2H, ArH), 6.75~6.72 (m, 1H, ArH), 3.64 (s, 3H, OCH_3), 2.26 (s, 3H, CH_3); ^{13}C NMR (150 MHz, DMSO-d_6) δ: 186.6, 158.1, 144.3, 139.4, 134.2, 133.7, 131.3, 130.5, 129.9, 128.8, 128.5, 127.2, 126.1, 125.1, 124.7, 123.0, 116.2, 115.9, 113.8, 55.0, 21.1; IR (KBr) υ: 2933, 1636, 1597, 1535, 1496, 1467, 1395, 1342, 1275, 1164, 1075, 1041, 930, 872, 830, 788, 747 cm^{-1}; MS (m/z): HRMS (ESI) Calcd. for $C_{25}H_{18}N_2NaO_4S$ ($[M+Na]^+$): 465.0879. Found: 465.088.

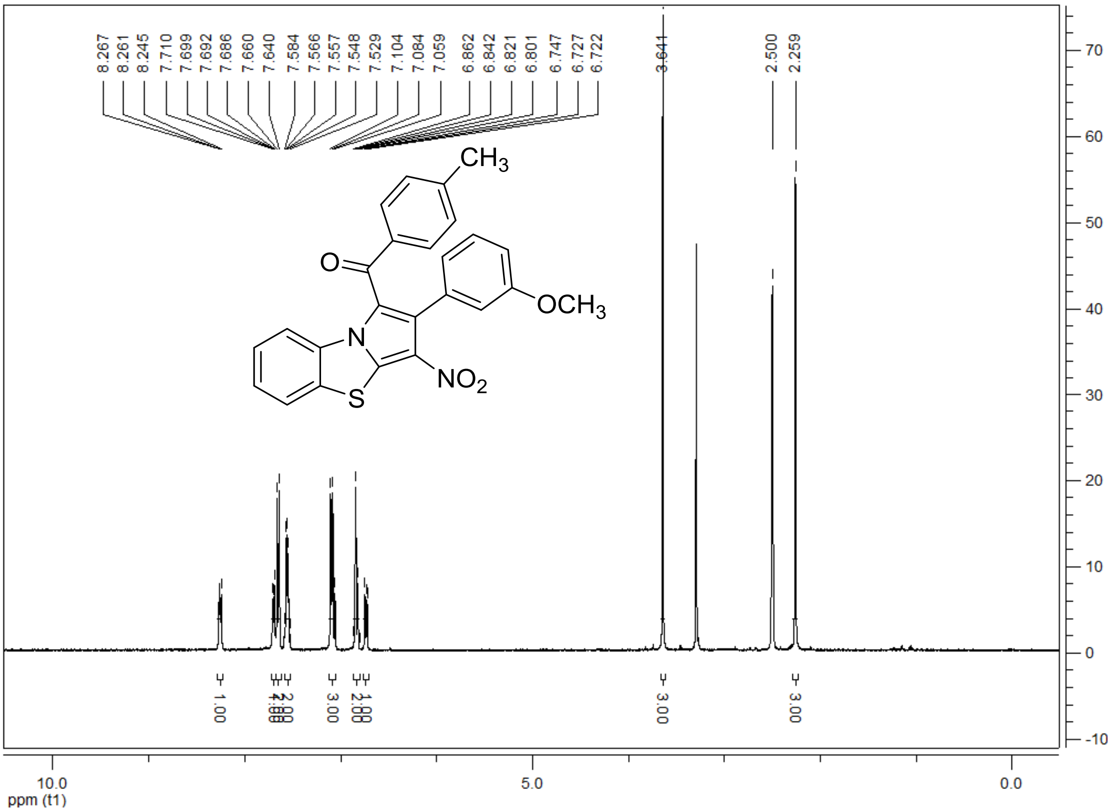

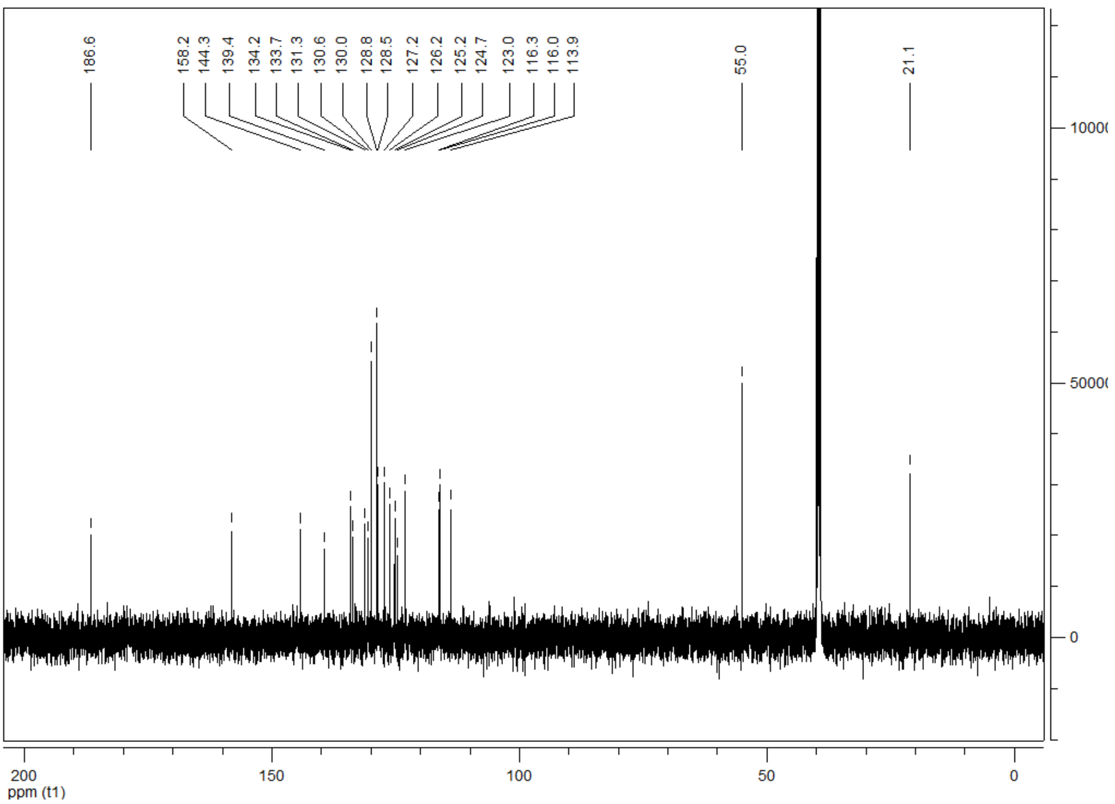
186.6
158.2
144.3
139.4
134.2
133.7
131.3
130.6
130.0
128.8
128.5
127.2
126.2
125.2
124.7
123.0
116.3
116.0
113.9
55.0
21.1
ppm (t1)

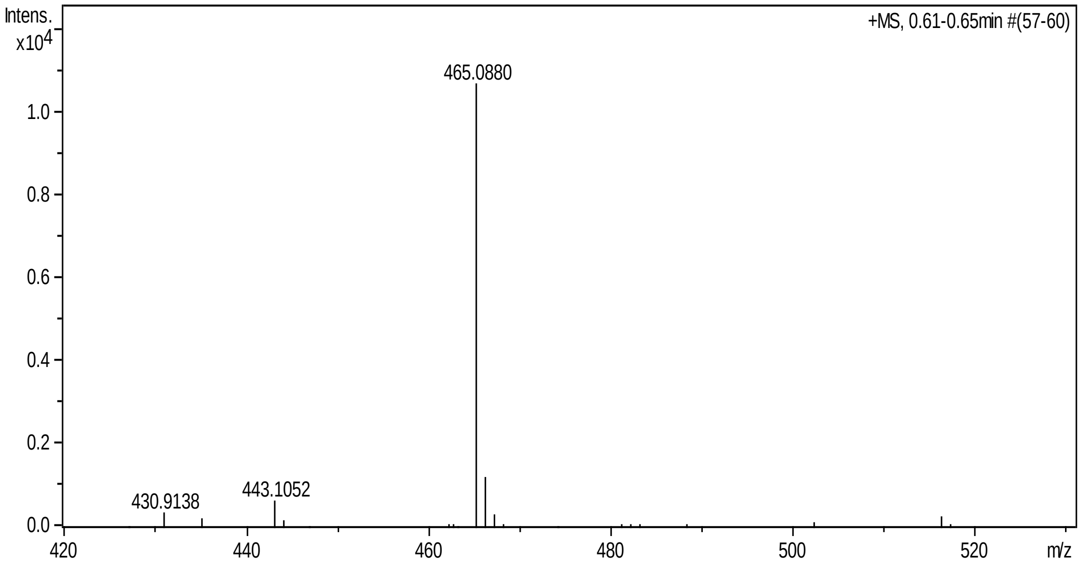
Intens.
x10^4
+MS, 0.61-0.65min #(57-60)
465.0880
443.1052
430.9138
m/z

(2-(2-hydroxyphenyl)-3-nitrobenzo[d]pyrrolo[2,1-b]thiazol-1-yl)(p-tolyl)methanone (3d): yellow solid, 85%, m.p. 224~226℃; ^{1}H NMR (400 MHz, DMSO-d_6) δ: 9.60 (s, 1H, OH), 8.27~8.22 (m, 1H, ArH), 7.82~7.78 (m, 1H, ArH), 7.64 (d, *J* = 8.0 Hz, 2H, ArH), 7.58~7.54 (m, 2H, ArH), 7.05 (d, *J* = 8.0 Hz, 2H, ArH), 7.00~6.91 (m, 2H, ArH), 6.63 (d, *J* = 8.0 Hz, 1H, ArH), 6.56~6.52 (m, 1H, ArH), 2.24 (s, 3H, CH_3); ^{13}C NMR (150 MHz, DMSO-d_6) δ: 186.0, 155.4, 143.7, 139.1, 134.2, 133.9, 131.4, 130.3, 129.7, 129.6, 128.4, 127.0, 126.6, 126.0, 124.9, 124.8, 118.0, 117.9, 116.3, 114.8, 21.1; IR (KBr) υ: 3378, 2922, 1636, 1603, 1500, 1398, 1341, 1272, 1164, 1100, 1053, 1010, 919, 833, 753, 712 cm^{-1}; MS (*m/z*): HRMS (ESI) Calcd. for $C_{24}H_{16}N_2NaO_4S$ ($[M+Na]^+$): 451.0723. Found: 451.0719.

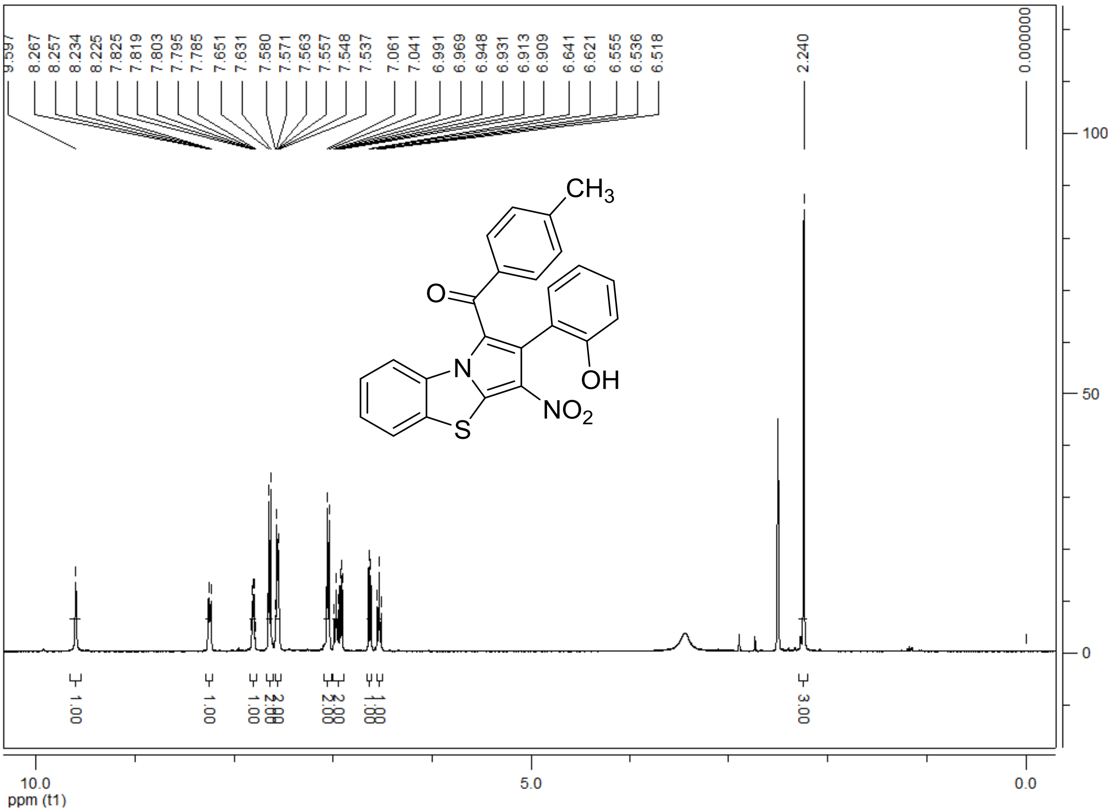

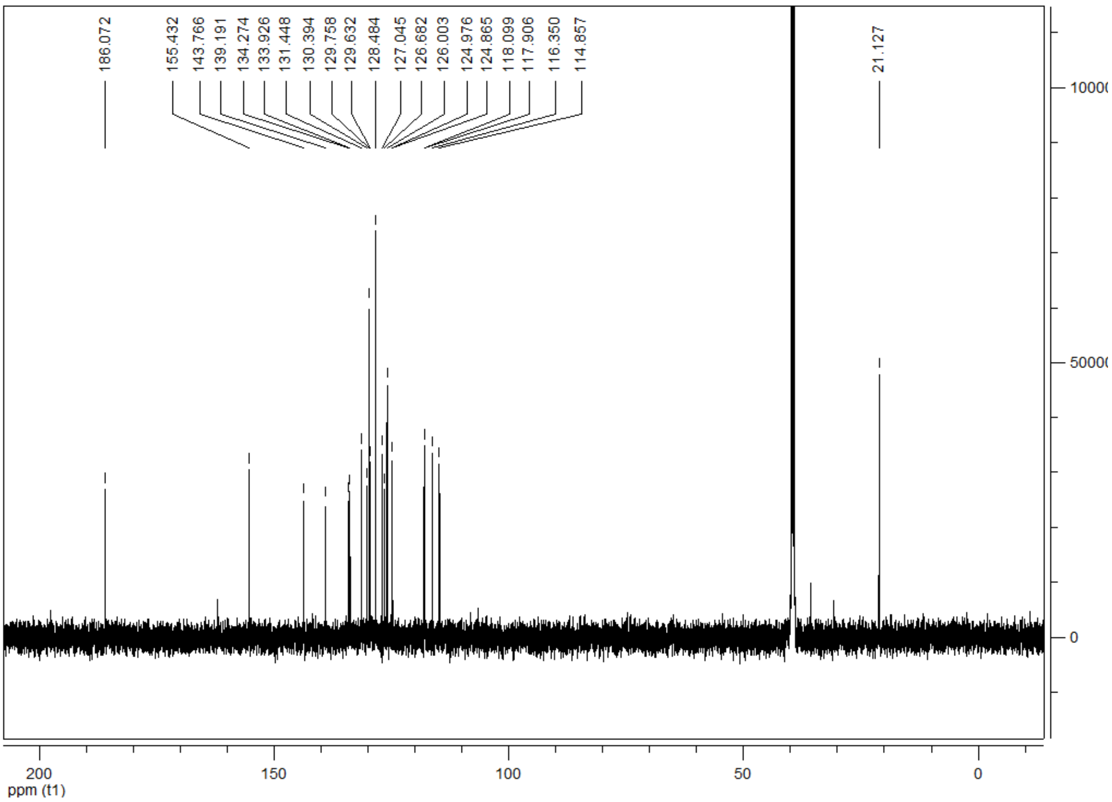
186.072
155.432
143.766
139.191
134.274
133.926
131.448
130.394
129.758
129.632
128.484
127.045
126.682
126.003
124.976
124.865
118.099
117.906
116.350
114.857
21.127
200
150
100
50
0
ppm (t1)

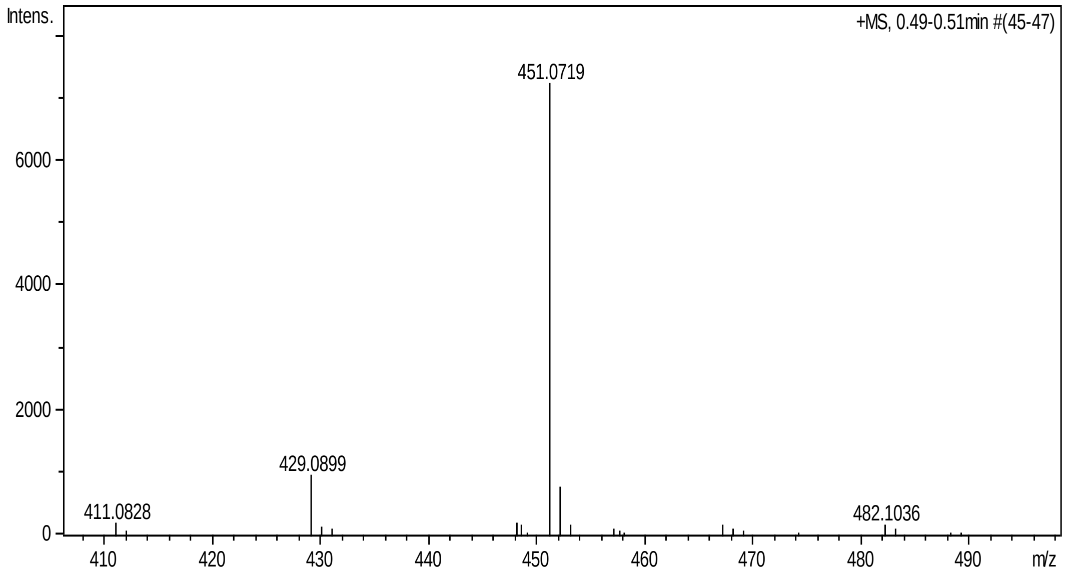
Intens.
+MS, 0.49-0.51min #(45-47)
451.0719
429.0899
411.0828
482.1036
6000
4000
2000
0
410
420
430
440
450
460
470
480
490
m/z

(2-(4-chlorophenyl)-3-nitrobenzo[d]pyrrolo[2,1-b]thiazol-1-yl)(p-tolyl)methanone (3e):

yellow solid, 85%, m.p. 208~210℃; ^{1}H NMR (400 MHz, DMSO-d_6) δ: 8.26~8.23 (m, 1H, ArH), 7.74~7.70 (m, 1H, ArH), 7.62 (d, *J* = 8.0 Hz, 2H, ArH), 7.57~7.53 (m, 2H, ArH), 7.29~7.22 (m, 4H, ArH), 7.10 (d, *J* = 8.0 Hz, 2H, ArH), 2.27 (s, 3H, CH_3); ^{13}C NMR (150 MHz, DMSO-d_6) δ: 186.3, 144.4, 139.6, 134.1, 133.7, 132.9, 132.4, 130.5, 130.0, 129.1, 128.9, 127.7, 127.4, 127.2, 126.2, 125.1, 124.9, 116.1, 21.1; IR (KBr) υ: 3140, 2919, 1914, 1638, 1604, 1501, 1465, 1400, 1337, 1270, 1164, 1088, 1051, 1009, 916, 830, 748, 705 cm^{-1}; MS (*m*/*z*): HRMS (ESI) Calcd. for $C_{24}H_{15}ClN_2NaO_3S$ ([M+Na]$^+$): 469.0384. Found: 469.0376.

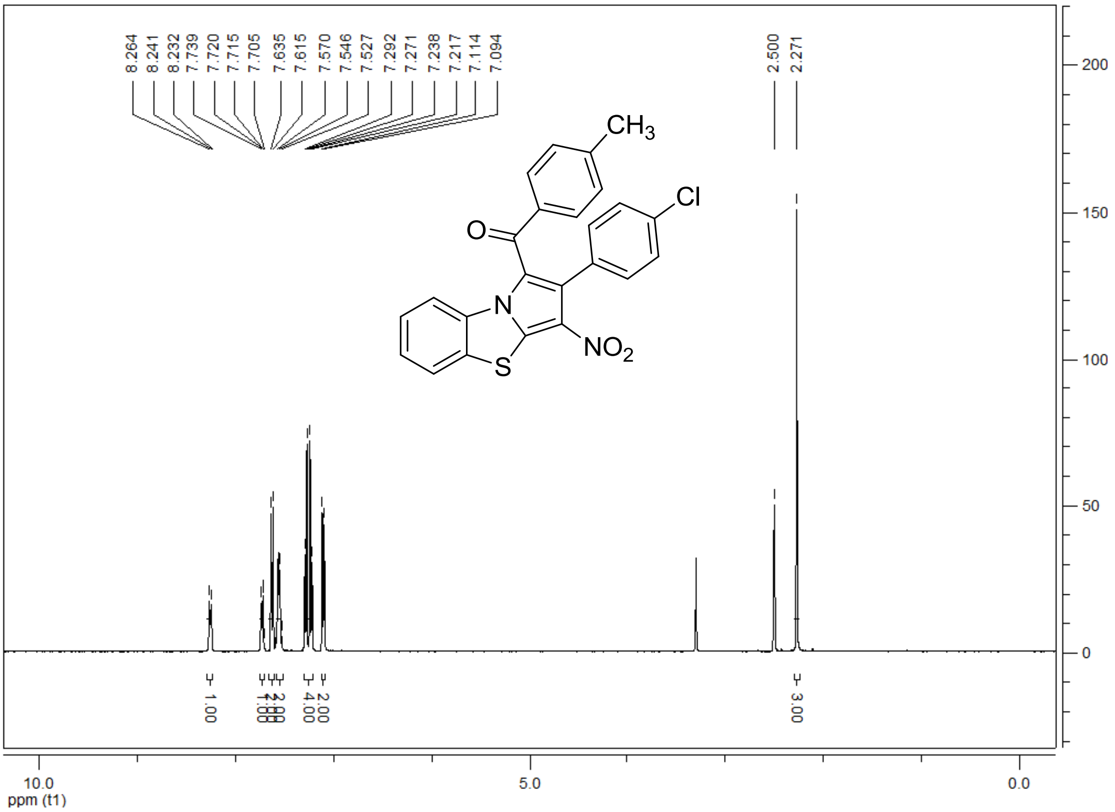

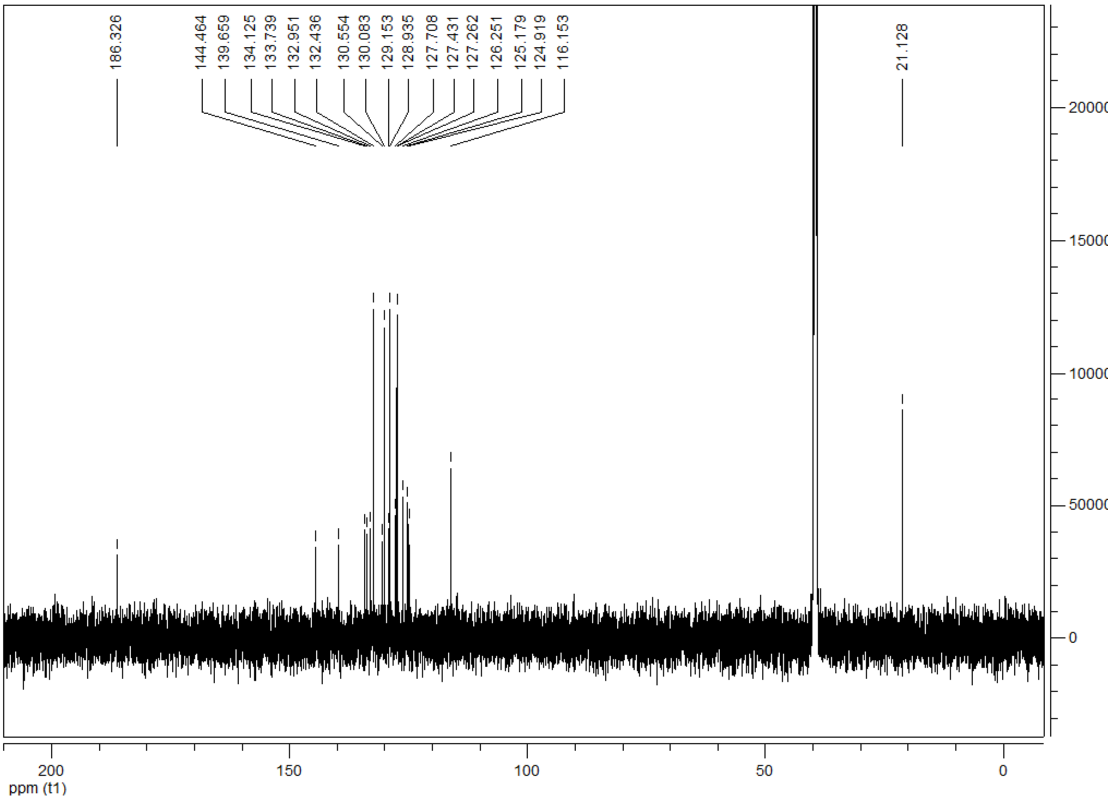
186.326
144.464
139.659
134.125
133.739
132.951
132.436
130.554
130.083
129.153
128.935
127.708
127.431
127.262
126.251
125.179
124.919
116.153
21.128
200
150
100
50
0
ppm (t1)

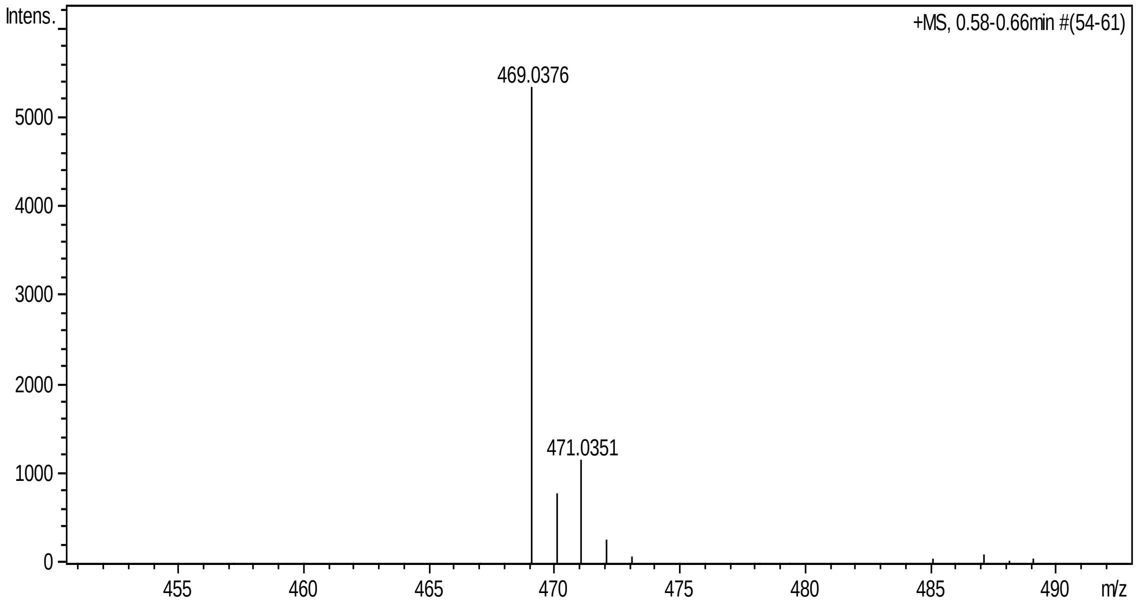
Intens.
+MS, 0.58-0.66min #(54-61)
469.0376
471.0351
5000
4000
3000
2000
1000
0
455
460
465
470
475
480
485
490
m/z

(2-(3-chlorophenyl)-3-nitrobenzo[d]pyrrolo[2,1-b]thiazol-1-yl)(p-tolyl)methanone (3f):

yellow solid, 86%, m.p. 217~219℃; ^{1}H NMR (400 MHz, DMSO-d_6) δ: 8.29~8.23 (m, 1H, ArH), 7.77~7.74 (m, 1H, ArH), 7.63 (d, J = 7.6 Hz, 2H, ArH), 7.60~7.56 (m, 2H, ArH), 7.34 (s, 1H, ArH), 7.24~7.16 (m, 3H, ArH), 7.10 (d, J = 7.6 Hz, 2H, ArH), 2.26 (s, 3H, CH_3); ^{13}C NMR (150 MHz, DMSO-d_6) δ: 186.2, 144.3, 139.6, 134.1, 133.6, 132.2, 132.0, 130.5, 130.4, 129.9, 129.2, 129.1, 128.8, 127.8, 127.4, 127.1, 126.2, 125.1, 116.2, 21.1; IR (KBr) υ: 3064, 1637, 1600, 1535, 1497, 1403, 1341, 1275, 1201, 1164, 1091, 1053, 1016, 924, 837, 785, 740 cm^{-1}; MS (m/z): HRMS (ESI) Calcd. for $C_{24}H_{15}ClN_2NaO_3S$ ([M+Na]$^+$): 469.0384. Found: 469.0380.

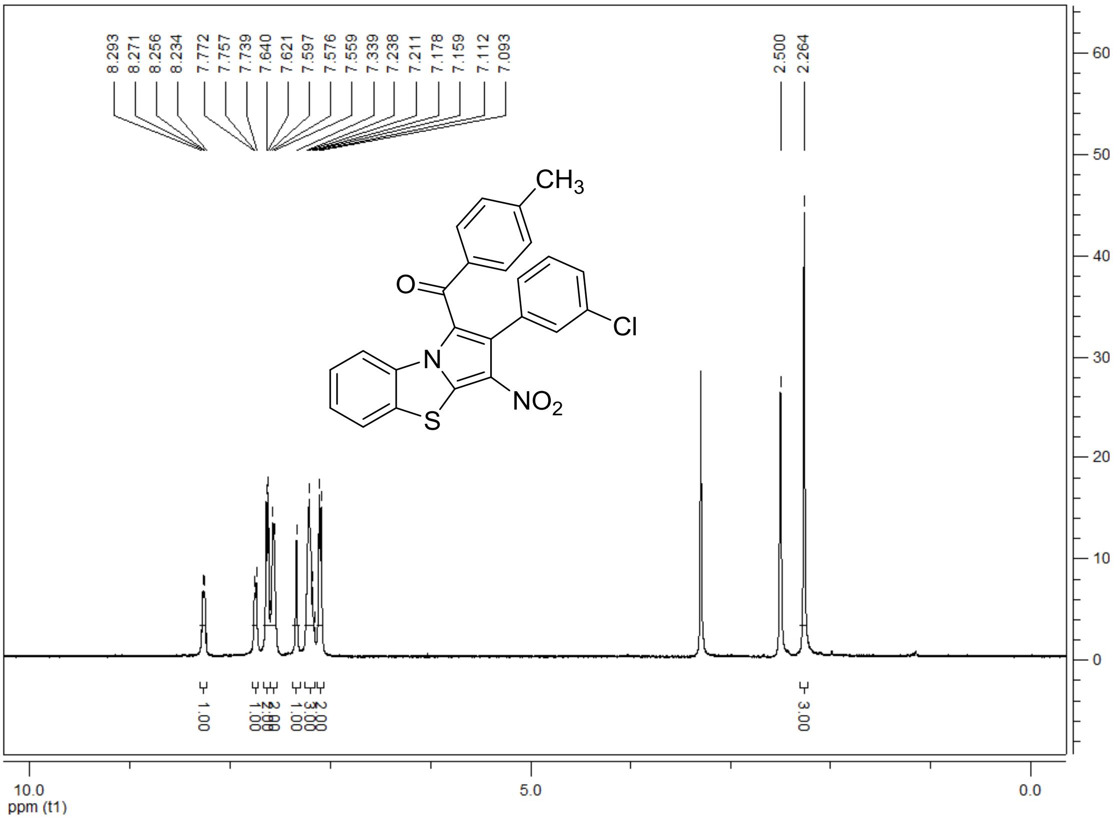

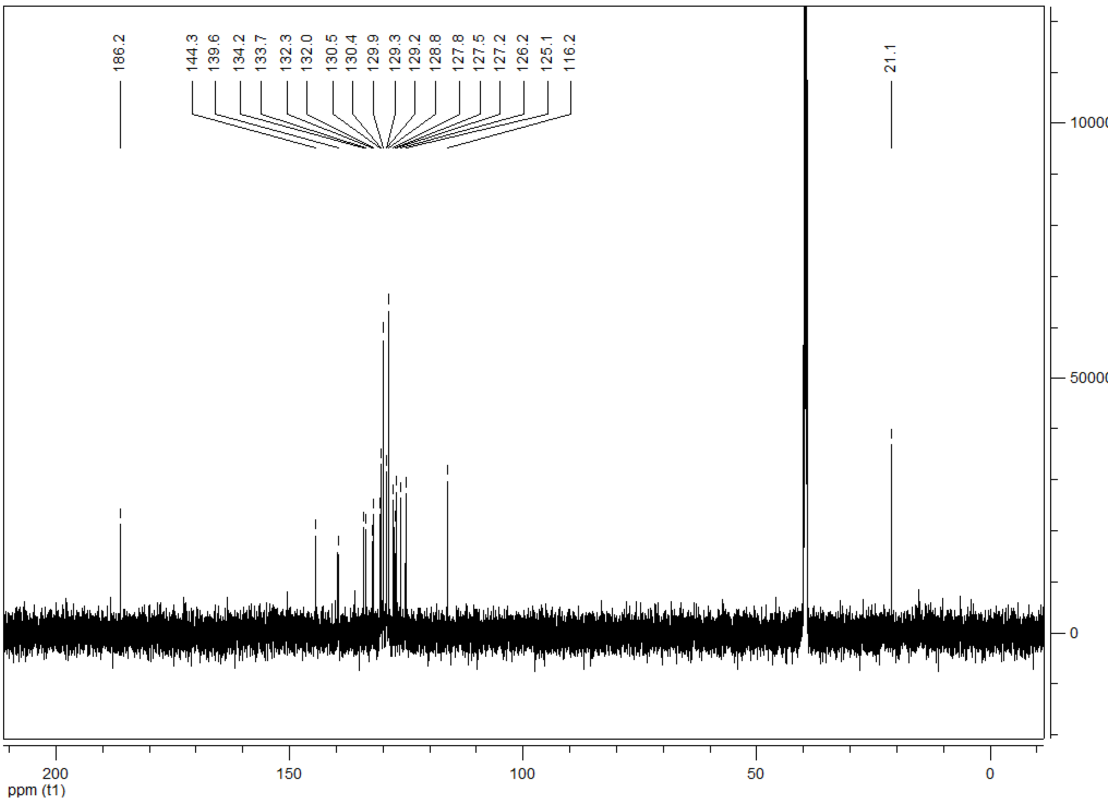

186.2
144.3
139.6
134.2
133.7
132.3
132.0
130.5
130.4
129.9
129.3
129.2
128.8
127.8
127.5
127.2
126.2
125.1
116.2
21.1
200
150
100
50
0
ppm (t1)

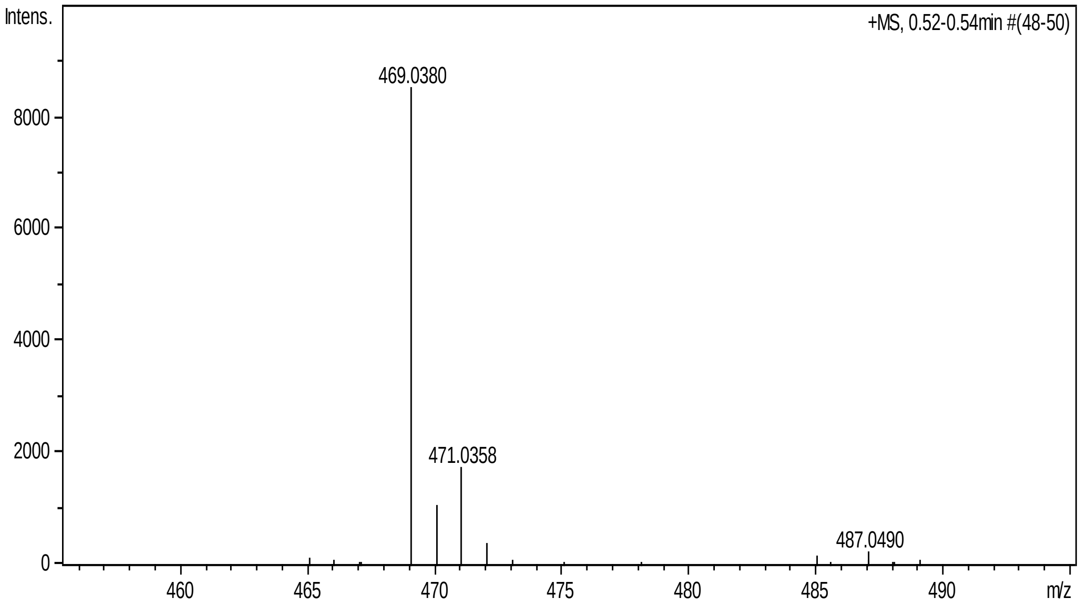

Intens.
+MS, 0.52-0.54min #(48-50)
469.0380
471.0358
487.0490
8000
6000
4000
2000
0
460
465
470
475
480
485
490
m/z

(2-(4-bromophenyl)-3-nitrobenzo[d]pyrrolo[2,1-b]thiazol-1-yl)(p-tolyl)methanone (3g): yellow solid, 88%, m.p. 204~206℃; ^{1}H NMR (400 MHz, DMSO-d_6) δ: 8.27~8.25 (m, 1H, ArH), 7.74~7.71 (m, 1H, ArH), 7.62 (d, J = 8.0 Hz, 2H, ArH), 7.57~7.53 (m, 2H, ArH), 7.37 (d, J = 8.4 Hz, 2H, ArH), 7.21 (d, J = 8.4 Hz, 2H, ArH), 7.11 (d, J = 8.0 Hz, 2H, ArH), 2.28 (s, 3H, CH_3); ^{13}C NMR (150 MHz, DMSO-d_6) δ: 186.3, 144.4, 139.6, 134.1, 133.7, 132.6, 130.5, 130.3, 130.0, 129.5, 128.9, 127.7, 127.2, 126.2, 125.2, 125.1, 124.8, 121.6, 116.1, 21.1; IR (KBr) υ: 3070, 2923, 1637, 1535, 1499, 1466, 1409, 1339, 1275, 1167, 1062, 1088, 918, 832, 750 cm^{-1}; MS (m/z): HRMS (ESI) Calcd. for $C_{24}H_{15}BrN_2NaO_3S$ ($[M+Na]^+$): 512.9879. Found: 512.9869.

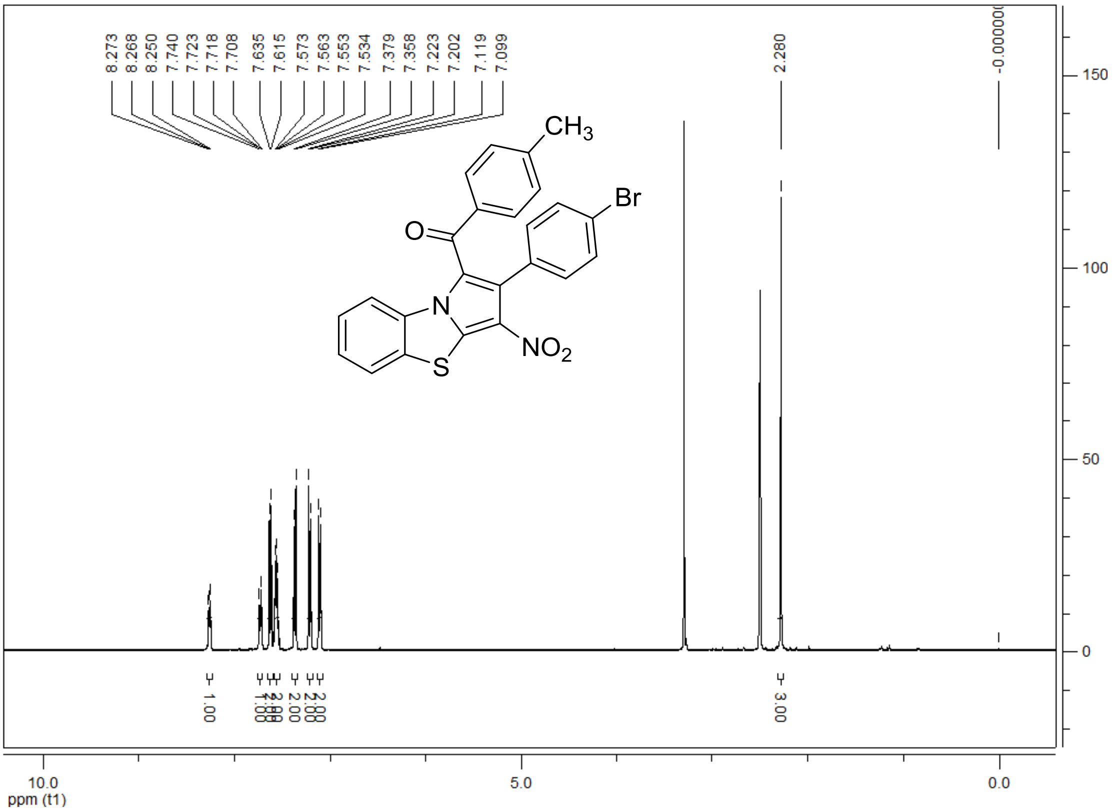

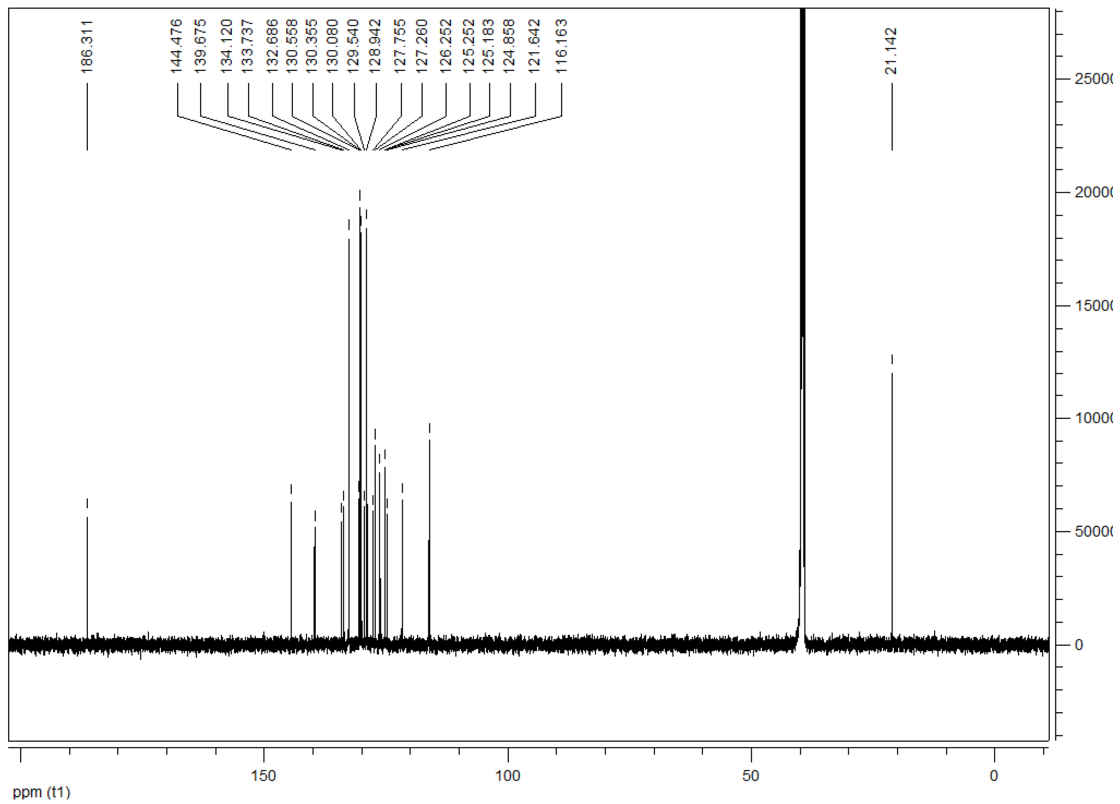
186.311
144.476
139.675
134.120
133.737
132.686
130.558
130.355
130.080
129.540
128.942
127.755
127.260
126.252
125.252
125.183
124.858
121.642
116.163
21.142
150
100
50
0
ppm (t1)

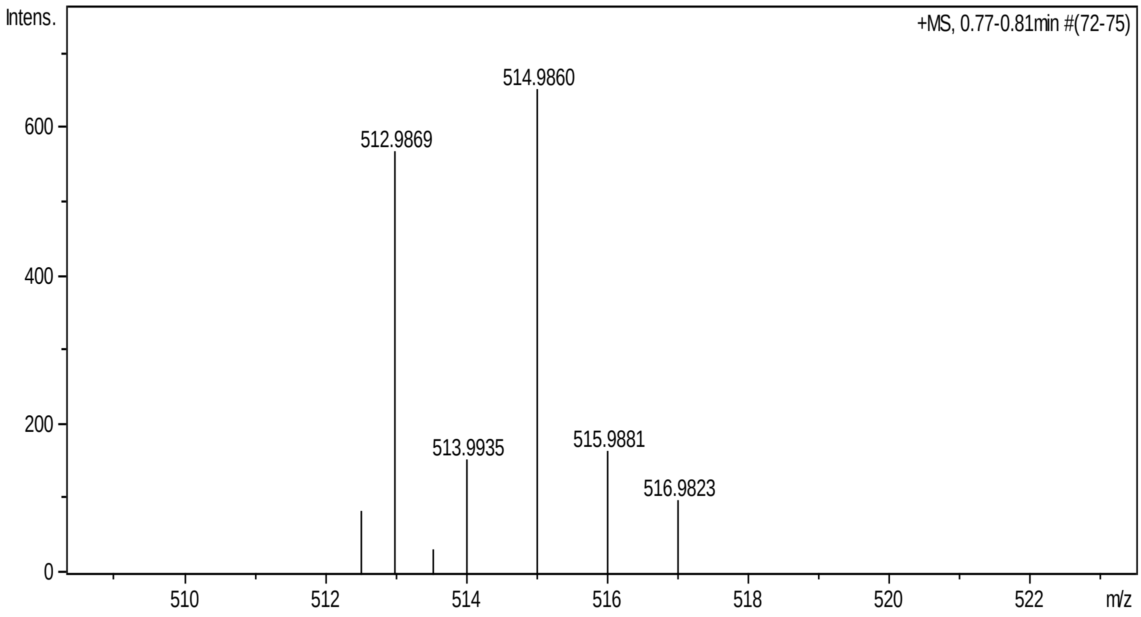
Intens.
+MS, 0.77-0.81min #(72-75)
514.9860
512.9869
513.9935
515.9881
516.9823
600
400
200
0
510
512
514
516
518
520
522
m/z

(3-nitro-2-(4-nitrophenyl)benzo[d]pyrrolo[2,1-b]thiazol-1-yl)(p-tolyl)methanone (3h)

yellow solid, 82%, m.p. 219~221℃; ^{1}H NMR (400 MHz, DMSO-d_6) δ: 8.28 (d, *J* = 7.6 Hz, 1H, ArH), 8.02 (d, *J* = 8.4 Hz, 2H, ArH), 7.76 (d, *J* = 8.0 Hz, 1H, ArH), 7.63~7.56 (m, 6H, ArH), 7.08 (d, *J* = 7.6 Hz, 2H, ArH), 2.22 (s, 3H, CH_3); ^{13}C NMR (150 MHz, DMSO-d_6) δ: 185.9, 146.7, 144.5, 139.9, 137.5, 134.1, 133.6, 132.1, 130.5, 130.0, 128.9, 127.2, 126.9, 126.3, 125.3, 125.1, 122.3, 116.3, 21.0; IR (KBr) υ: 1638, 1513, 1468, 1385, 1342, 1276, 1166, 1105, 1049, 1009, 919, 840, 758 cm^{-1}; MS (*m/z*): HRMS (ESI) Calcd. for $C_{24}H_{15}N_3NaO_5S$ ($[M+Na]^+$): 480.0625. Found: 480.0621.

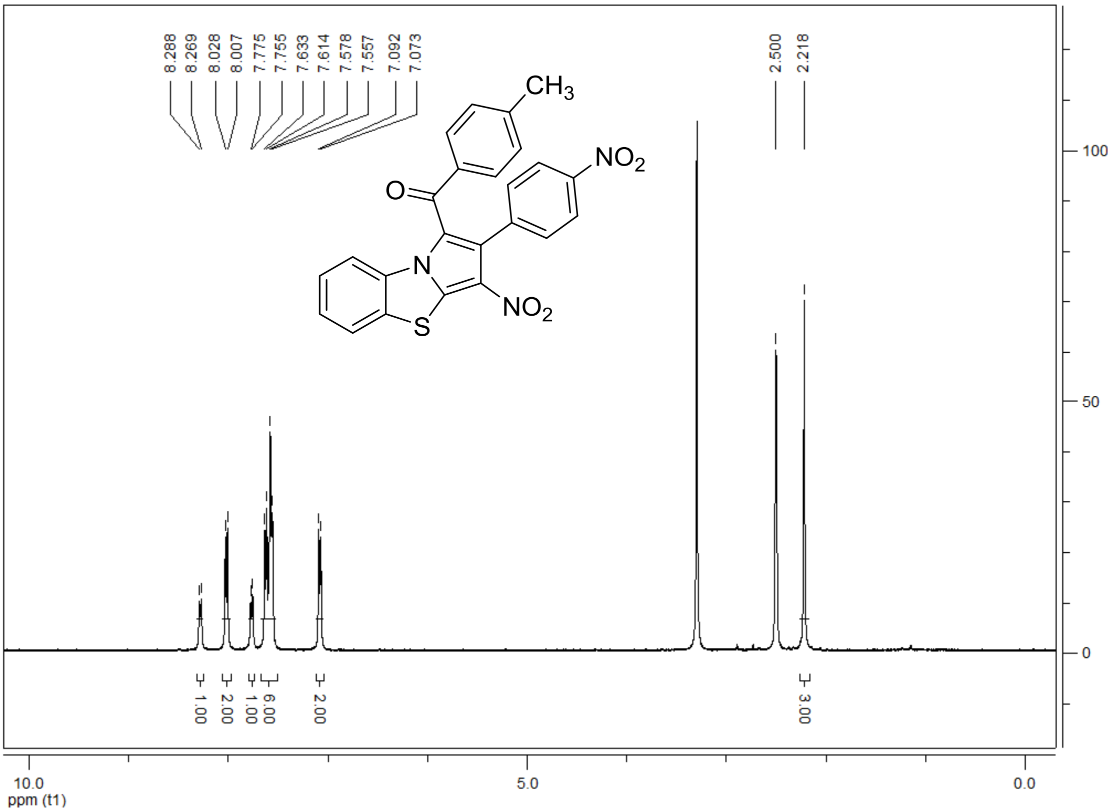

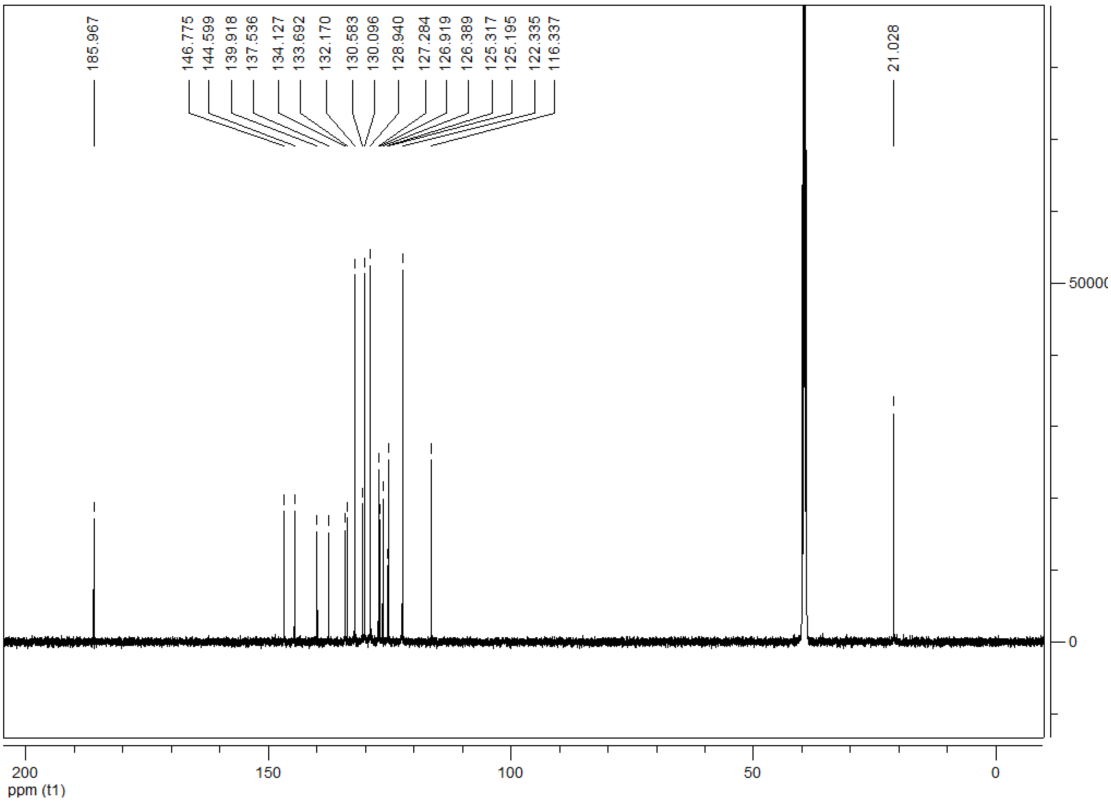
185.967
146.775
144.599
139.918
137.536
134.127
133.692
132.170
130.583
130.096
128.940
127.284
126.919
126.389
125.317
125.195
122.335
116.337
21.028
50000
0
200
150
100
50
0
ppm (t1)

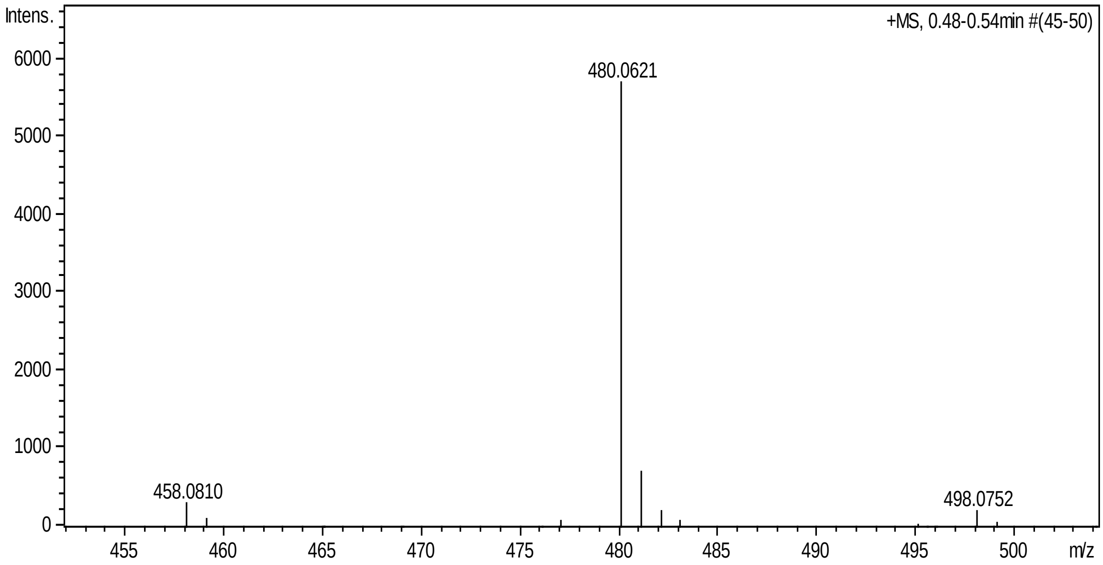
Intens.
+MS, 0.48-0.54min #(45-50)
480.0621
458.0810
498.0752
6000
5000
4000
3000
2000
1000
0
455
460
465
470
475
480
485
490
495
500
m/z

(3-nitro-2-(3-nitrophenyl)benzo[d]pyrrolo[2,1-b]thiazol-1-yl)(p-tolyl)methanone (3i):

yellow solid, 78%, m.p. 206~208℃; ^{1}H NMR (600 MHz, DMSO-d_6) δ: 8.28 (d, J = 8.4 Hz, 1H, ArH), 8.02 (d, J = 8.4 Hz, 2H, ArH), 7.77 (d, J = 7.8 Hz, 1H, ArH), 7.63 (d, J = 7.8 Hz, 2H, ArH), 7.60~7.56 (m, 4H, ArH), 7.08 (d, J = 7.8 Hz, 2H, ArH), 2.22 (s, 3H, CH_3); ^{13}C NMR (150 MHz, DMSO-d_6) δ: 185.9, 146.7, 144.5, 139.9, 137.5, 134.1, 133.6, 132.1, 130.5, 130.0, 128.9, 127.2, 126.9, 126.3, 125.2, 125.1, 122.3, 116.3, 21.0; IR (KBr) υ: 3075, 1638, 1513, 1468, 1385, 1342, 1276, 1167, 1049, 1006, 919, 839, 757 cm^{-1}; MS (m/z): HRMS (ESI) Calcd. for $C_{24}H_{15}N_3NaO_5S$ ($[M+Na]^+$): 480.0625. Found: 480.0610.

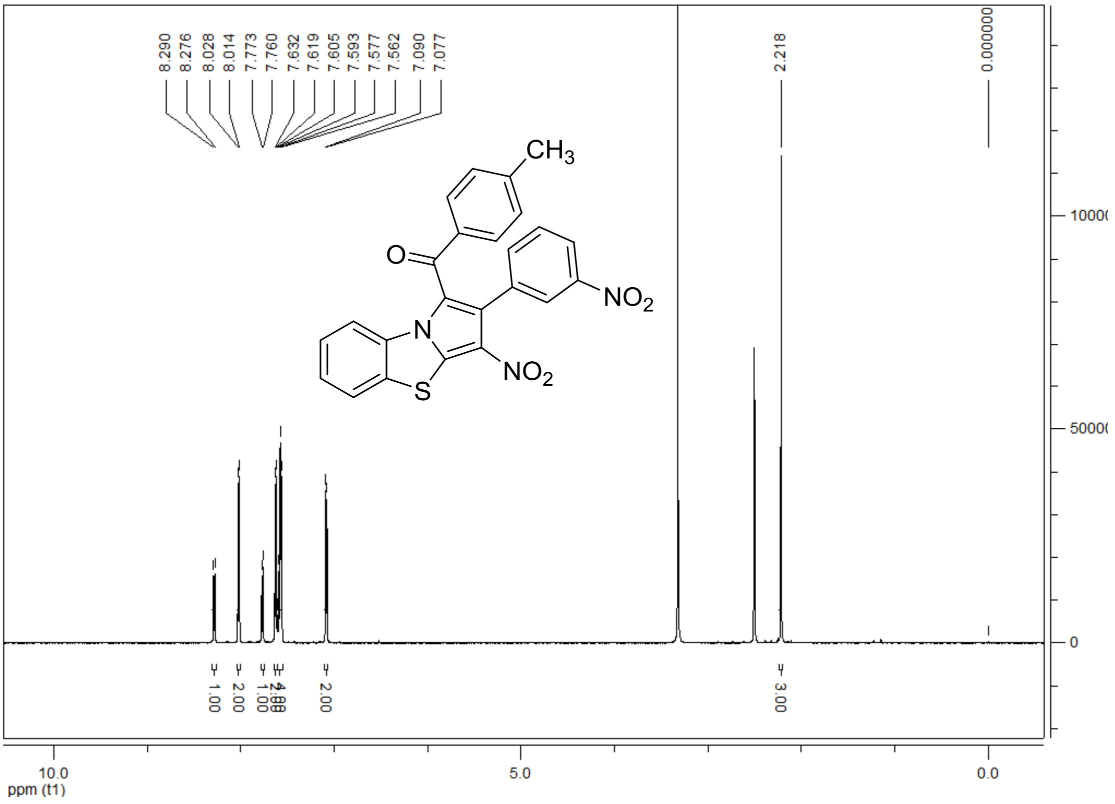

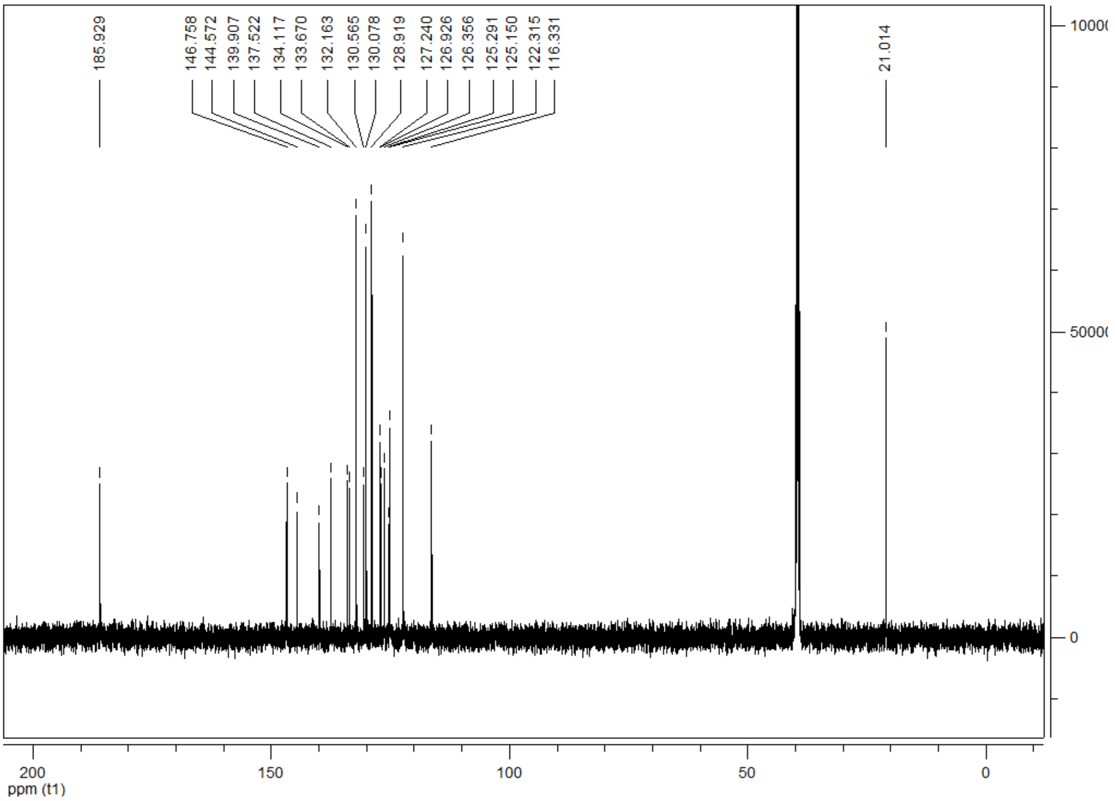
185.929
146.758
144.572
139.907
137.522
134.117
133.670
132.163
130.565
130.078
128.919
127.240
126.926
126.356
125.291
125.150
122.315
116.331
21.014
ppm (t1)

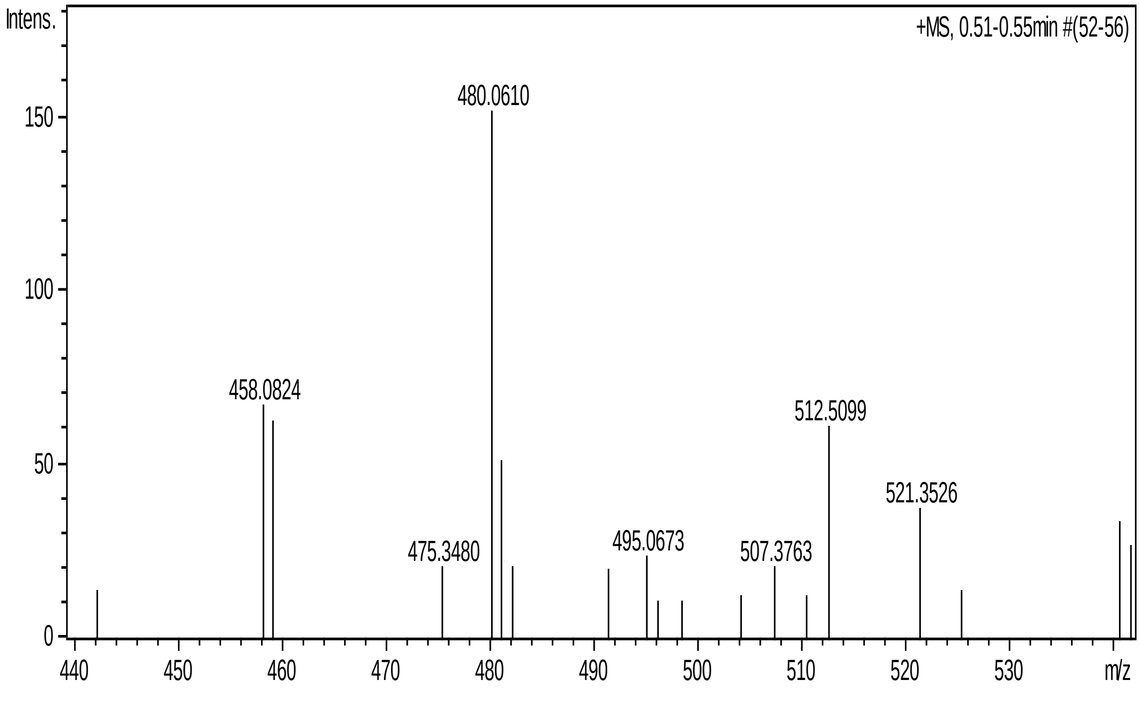
Intens.
+MS, 0.51-0.55min #(52-56)
480.0610
458.0824
512.5099
521.3526
475.3480
495.0673
507.3763
m/z

(4-methoxyphenyl)(2-(4-methoxyphenyl)-3-nitrobenzo[d]pyrrolo[2,1-b]thiazol-1-yl)methanone (3j):

yellow solid, 92%, m.p. 174~176℃; ^{1}H NMR (600 MHz, $CDCl_3$) δ: 7.91~7.89 (m, 1H, ArH), 7.85~7.83 (m, 1H, ArH), 7.70 (d, *J* = 9.0 Hz, 2H, ArH), 7.47~7.43 (m, 2H, ArH), 7.21 (d, *J* = 8.4 Hz, 2H, ArH), 6.70~6.67 (m, 4H, ArH), 3.78 (s, 3H, OCH_3), 3.72 (s, 3H, OCH_3); ^{13}C NMR (100 MHz, $CDCl_3$) δ: 185.9, 163.8, 159.5, 139.4, 134.4, 132.5, 132.2, 131.1, 129.7, 129.5, 126.9, 125.8, 124.9, 123.8, 122.3, 116.8, 113.5, 113.2, 55.4, 55.1; IR (KBr) υ: 3005, 2829, 1740, 1603, 1513, 1471, 1386, 1342, 1249, 1165, 1017, 952, 918, 836, 749 cm^{-1}; MS (*m*/*z*): HRMS (ESI) Calcd. for $C_{25}H_{18}N_2NaO_5S$ ($[M+Na]^+$): 481.0829. Found: 481.0830.

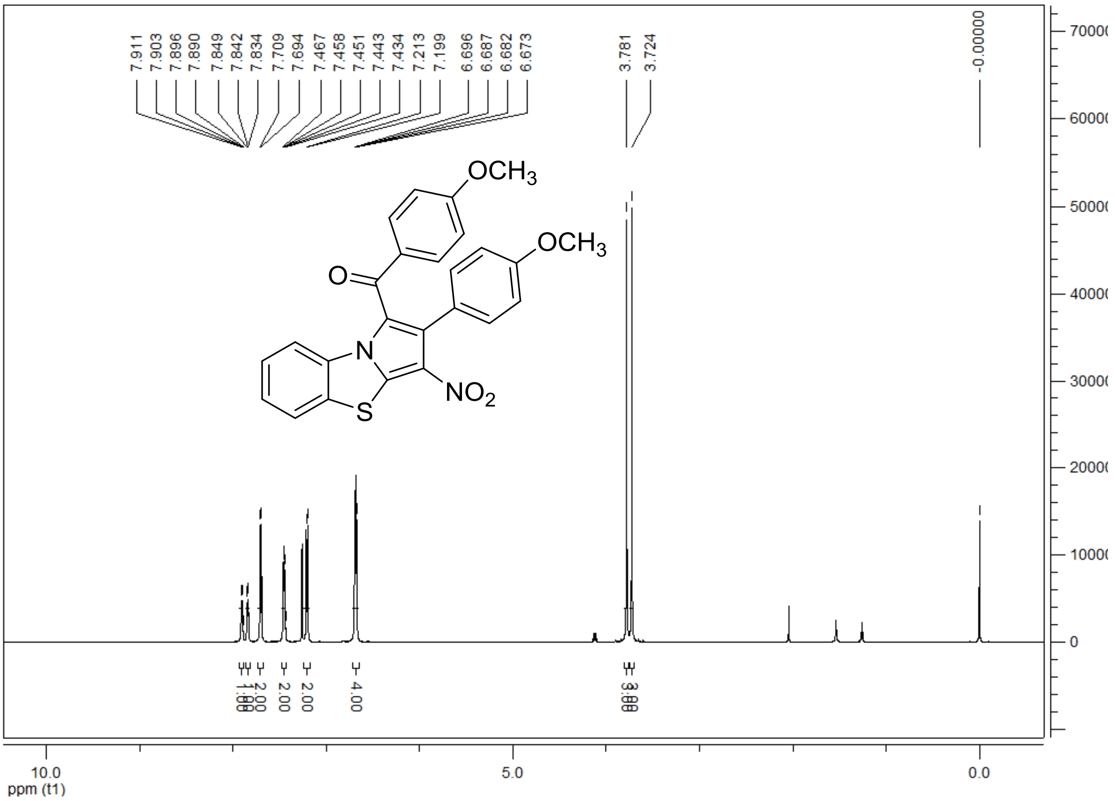

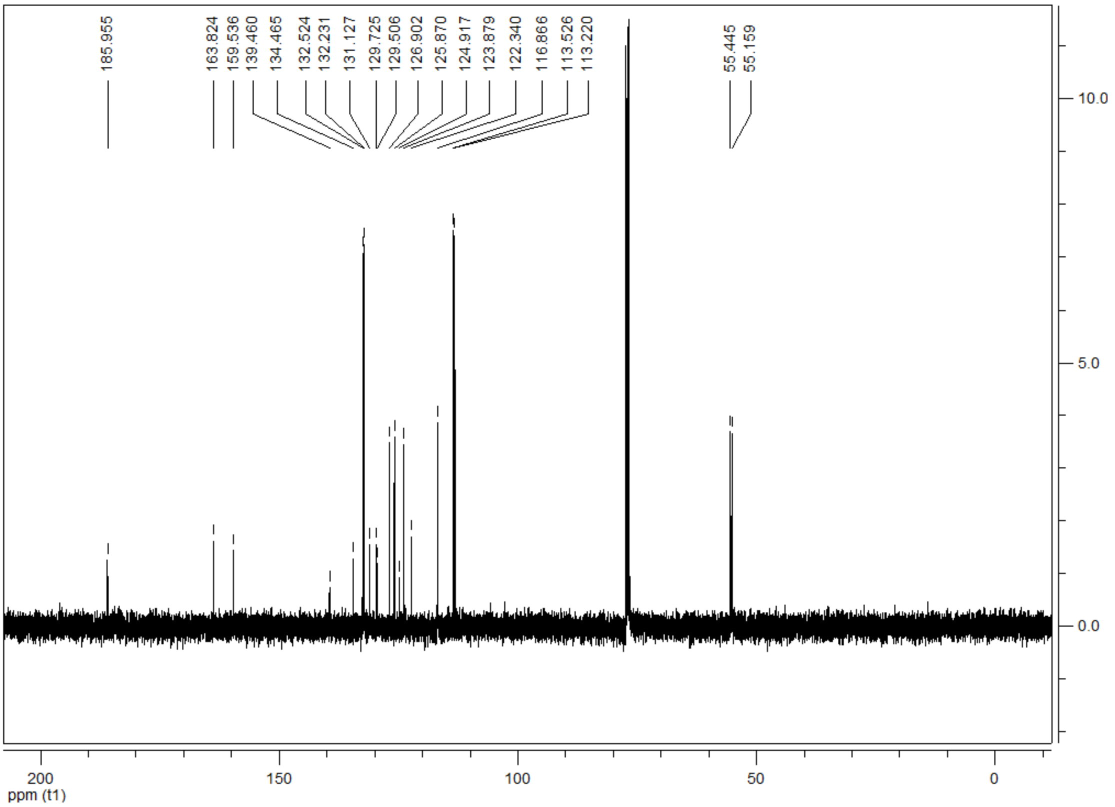
185.955
163.824
159.536
139.460
134.465
132.524
132.231
131.127
129.725
129.506
126.902
125.870
124.917
123.879
122.340
116.866
113.526
113.220
55.445
55.159
10.0
5.0
0.0
200
150
100
50
0
ppm (t1)

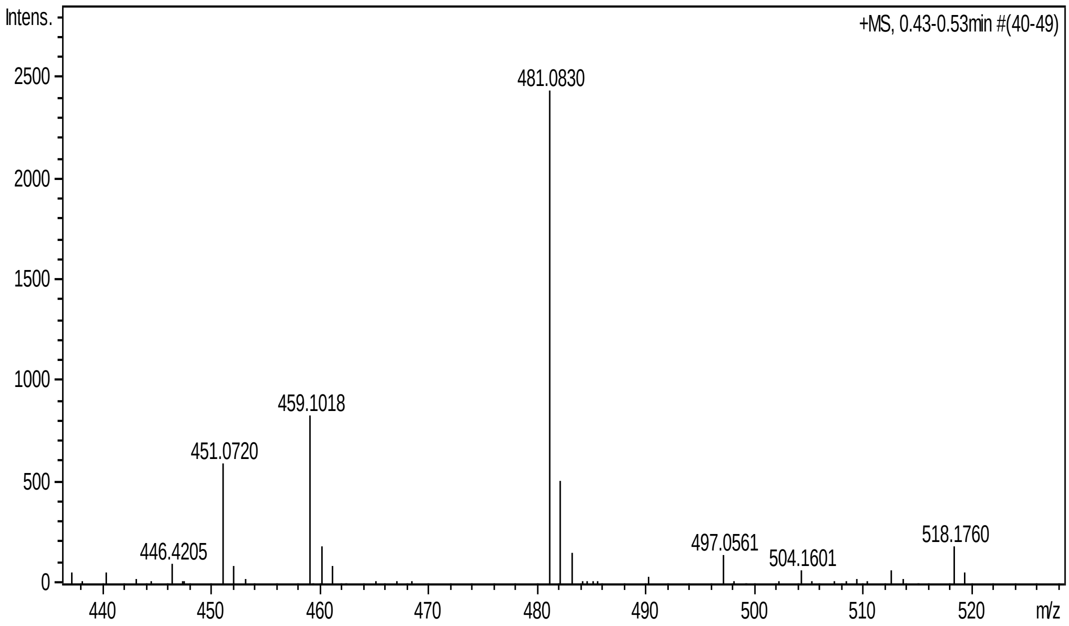
Intens.
+MS, 0.43-0.53min #(40-49)
2500
2000
1500
1000
500
0
481.0830
459.1018
451.0720
446.4205
497.0561
504.1601
518.1760
440
450
460
470
480
490
500
510
520
m/z

(2-(4-chlorophenyl)-3-nitrobenzo[d]pyrrolo[2,1-b]thiazol-1-yl)(4-methoxyphenyl)methanone (3k) : yellow solid, 82%, m.p. 188~190℃; ^{1}H NMR (400 MHz, DMSO-d_6) δ: 8.28~8.22 (m, 1H, ArH), 7.74~7.68 (m, 3H, ArH), 7.56~7.55 (m, 2H, ArH), 7.31~7.25 (m, 4H, ArH), 6.83 (d, J = 8.4 Hz, 2H, ArH), 3.77 (s, 3H, OCH_3); ^{13}C NMR (150 MHz, DMSO-d_6) δ: 185.0, 163.7, 139.3, 133.6, 132.8, 132.5, 132.3, 130.5, 129.2, 127.4, 127.2, 127.0, 126.1, 125.1, 125.1, 124.7, 115.9, 113.7, 55.6; IR (KBr) υ: 3072, 1639, 1595, 1501, 1466, 1402, 1340, 1259, 1189, 1160, 1092, 1016, 918, 841, 791, 749, 705 cm^{-1}; MS (m/z): HRMS (ESI) Calcd. for $C_{24}H_{15}ClN_2NaO_4S$ ($[M+Na]^+$): 485.0333. Found: 485.0332.

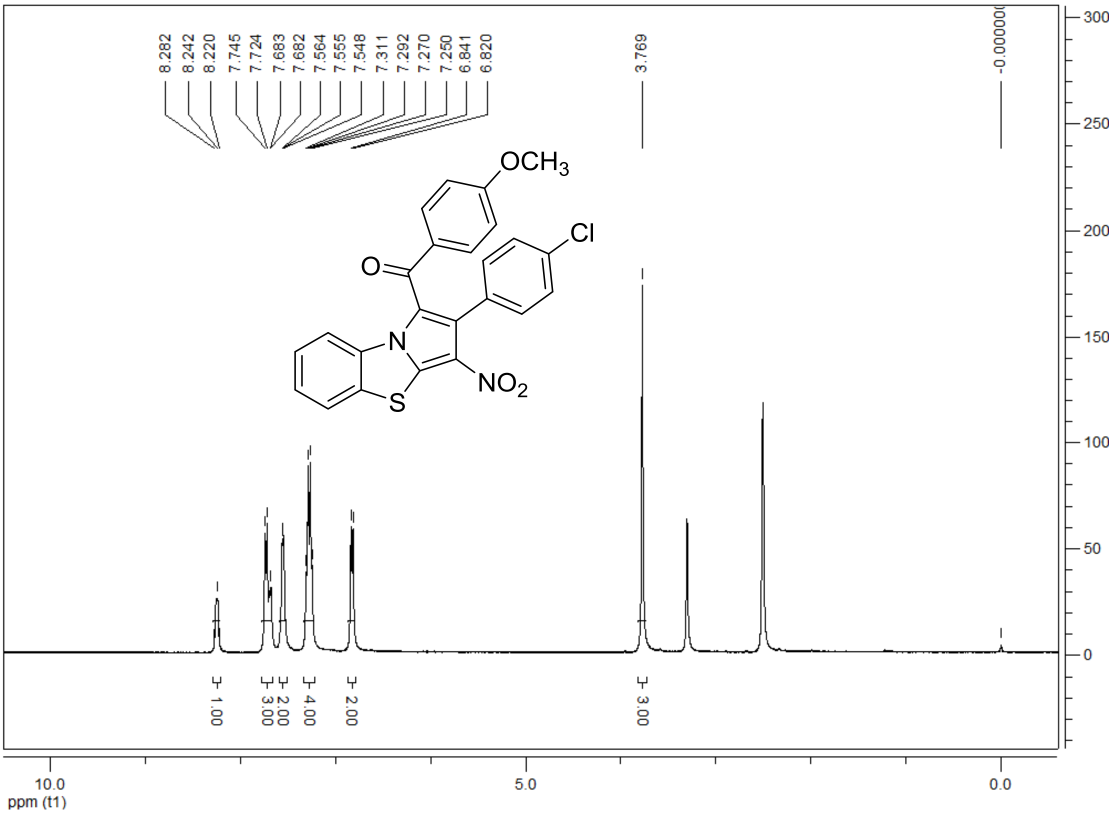

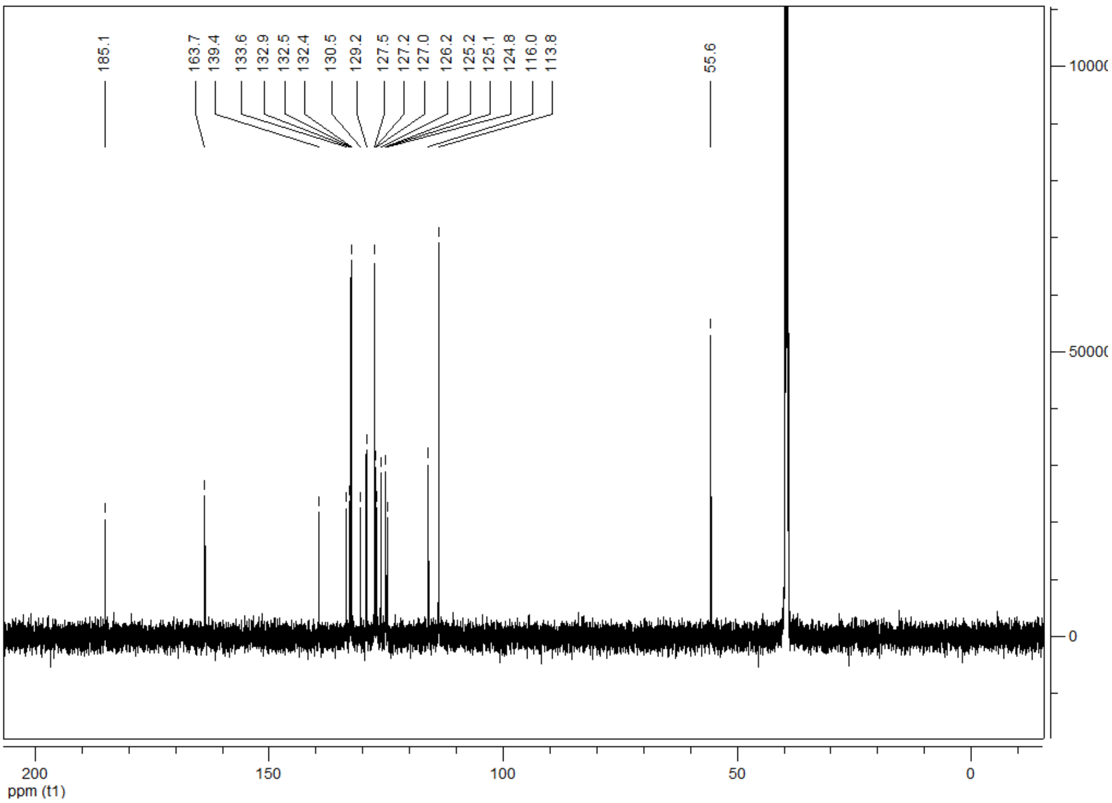
185.1
163.7
139.4
133.6
132.9
132.5
132.4
130.5
129.2
127.5
127.2
127.0
126.2
125.2
125.1
124.8
116.0
113.8
55.6
200
150
100
50
0
ppm (t1)
50000
0

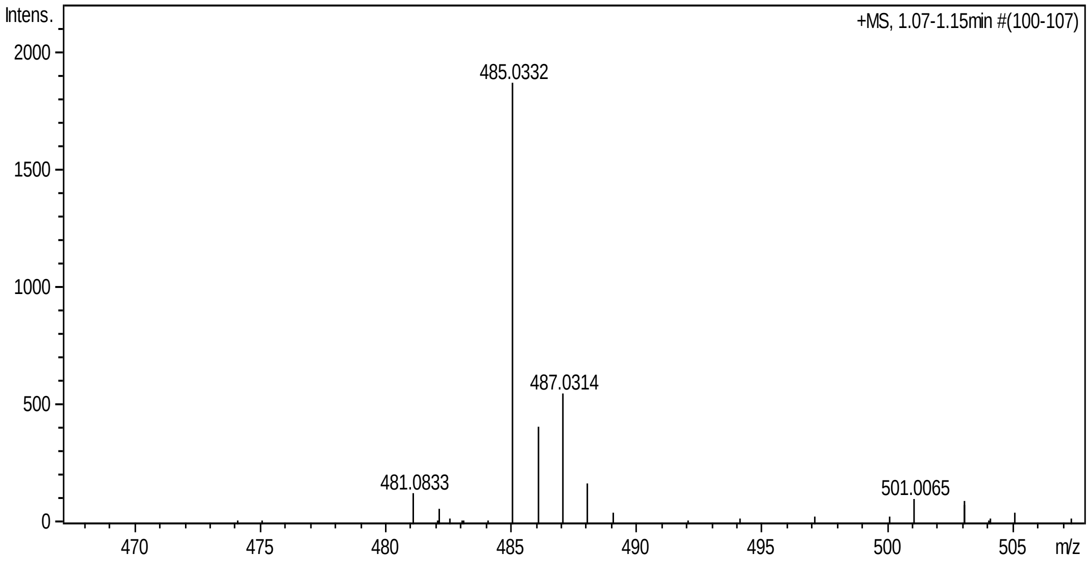
Intens.
+MS, 1.07-1.15min #(100-107)
2000
1500
1000
500
0
485.0332
487.0314
481.0833
501.0065
470
475
480
485
490
495
500
505
m/z

(2-(4-bromophenyl)-3-nitrobenzo[d]pyrrolo[2,1-b]thiazol-1-yl)(4-methoxyphenyl)methanone (3l): yellow solid, 83%, m.p. 166~168℃; ^{1}H NMR (400 MHz, DMSO-d_6) δ: 8.26~8.24 (m, 1H, ArH), 7.74~7.68 (m, 3H, ArH), 7.59~7.55 (m, 2H, ArH), 7.40~7.39 (m, 2H, ArH), 7.24~7.22 (m, 2H, ArH), 6.86~6.80 (m, 2H, ArH), 3.77 (s, 3H, OCH_3); ^{13}C NMR (150 MHz, DMSO-d_6) δ: 185.1, 163.7, 139.3, 133.6, 132.6, 132.5, 130.5, 130.4, 129.6, 129.2, 127.2, 127.0, 126.2, 125.1, 124.7, 121.5, 115.9, 113.8, 55.6; IR (KBr) υ: 3071, 2835, 1706, 1635, 1595, 1503, 1465, 1399, 1340, 1257, 1164, 1063, 1014, 918, 842, 789, 748 cm^{-1}; MS (*m*/*z*): HRMS (ESI) Calcd. for $C_{24}H_{15}BrN_2NaO_4S$ ($[M+Na]^+$): 528.9828. Found: 528.9820.

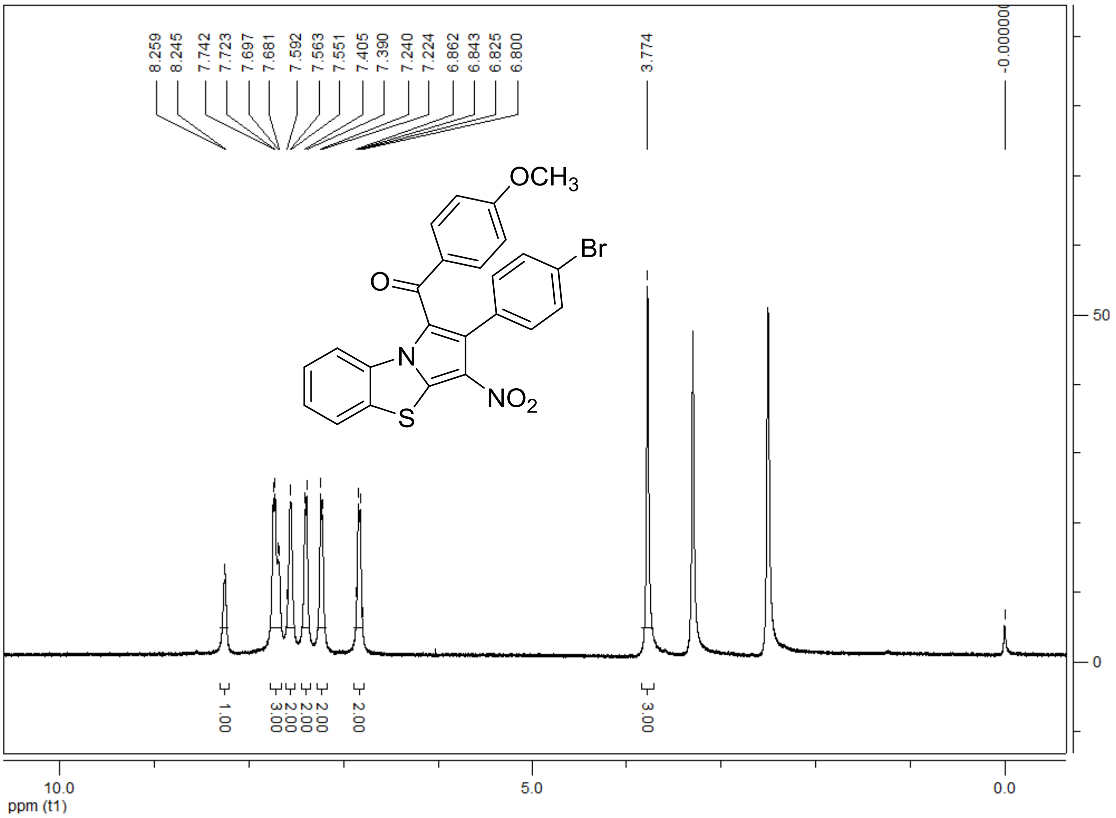

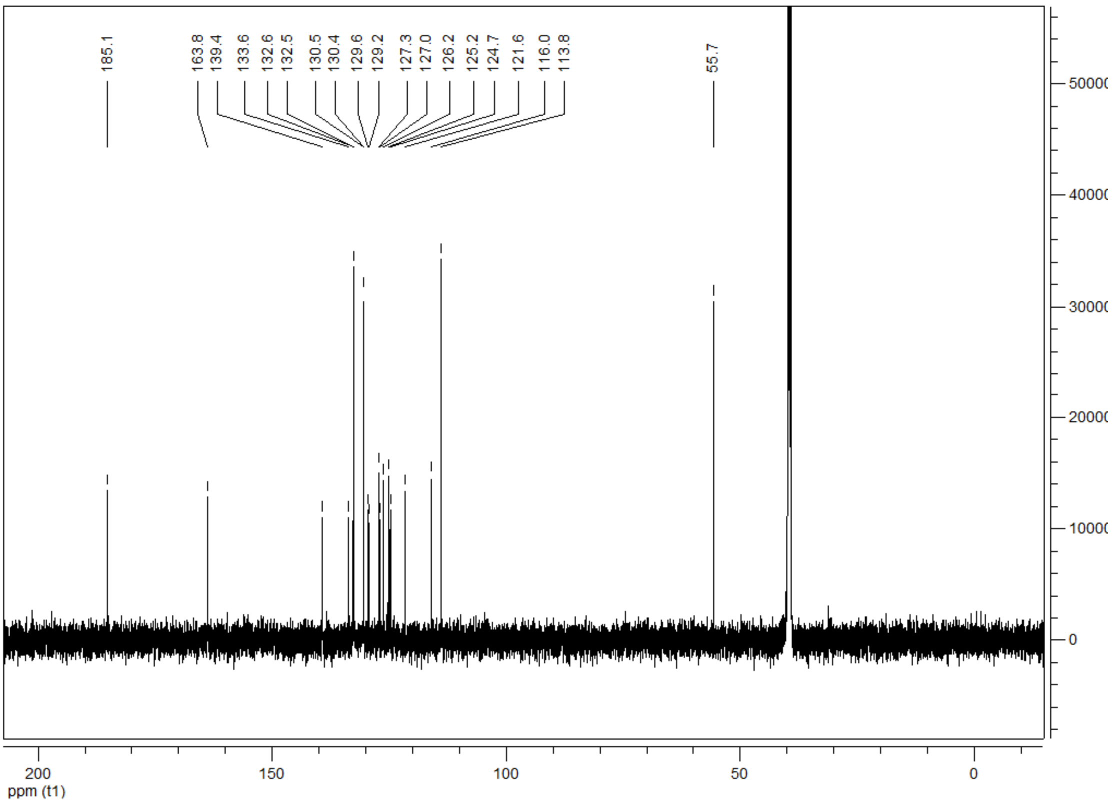
185.1
163.8
139.4
133.6
132.6
132.5
130.5
130.4
129.6
129.2
127.3
127.0
126.2
125.2
124.7
121.6
116.0
113.8
55.7
ppm (t1)

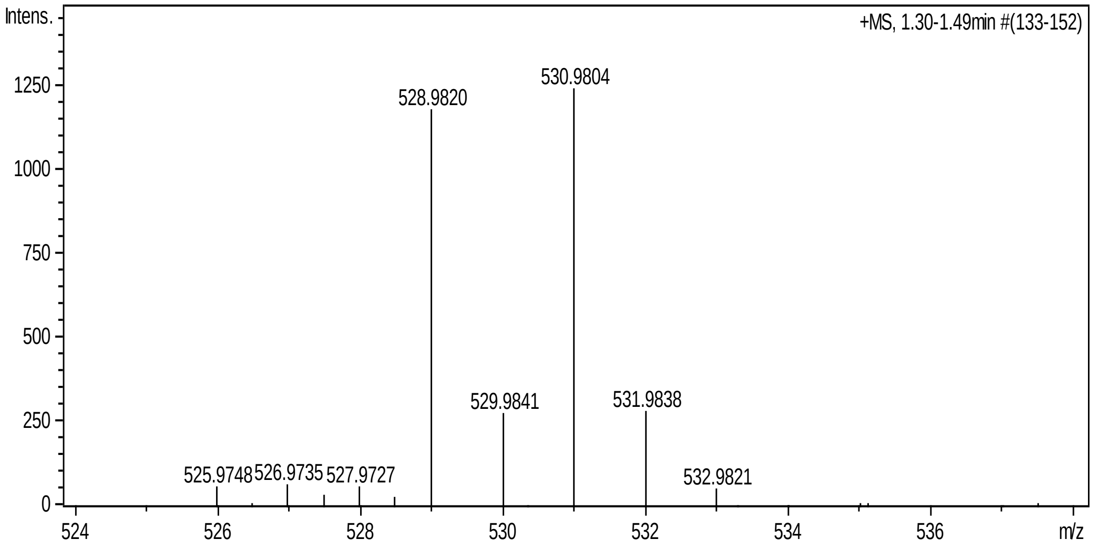
Intens.
+MS, 1.30-1.49min #(133-152)
528.9820
530.9804
529.9841
531.9838
525.9748
526.9735
527.9727
532.9821
m/z

(4-methoxyphenyl)(3-nitro-2-(4-nitrophenyl)benzo[d]pyrrolo[2,1-b]thiazol-1-yl)methanone (3m): yellow solid, 76%, m.p. 198~200℃; ^{1}H NMR (600 MHz, DMSO-d_6) δ: 8.28 (d, J = 7.2 Hz, 1H, ArH), 8.05 (d, J = 9.0 Hz, 2H, ArH), 7.74~7.71 (m, 3H, ArH), 7.59~7.55 (m, 4H, ArH), 6.81 (d, J = 8.4 Hz, 2H, ArH), 3.72 (s, 3H, OCH_3); ^{13}C NMR (150 MHz, DMSO-d_6) δ: 184.7, 163.8, 146.7, 139.5, 137.5, 133.5, 132.5, 132.0, 130.5, 129.2, 127.2, 126.3, 126.1, 125.2, 125.1, 125.0, 122.3, 116.1, 113.8, 55.6; IR (KBr) υ: 3078, 1637, 1597, 1509, 1468, 1386, 1344, 1259, 1159, 1107, 1021, 920, 841, 728 cm^{-1}; MS (m/z): HRMS (ESI) Calcd. for $C_{24}H_{15}N_3NaO_6S$ ($[M+Na]^+$): 496.0574. Found: 496.0573.

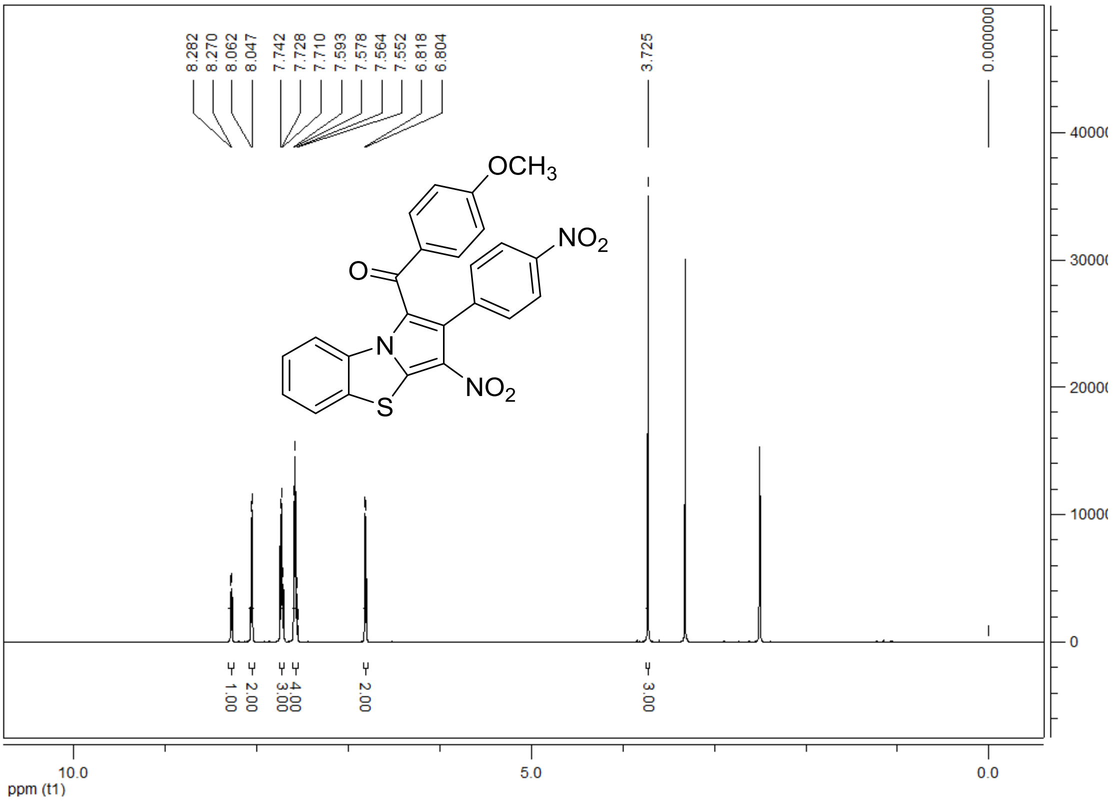

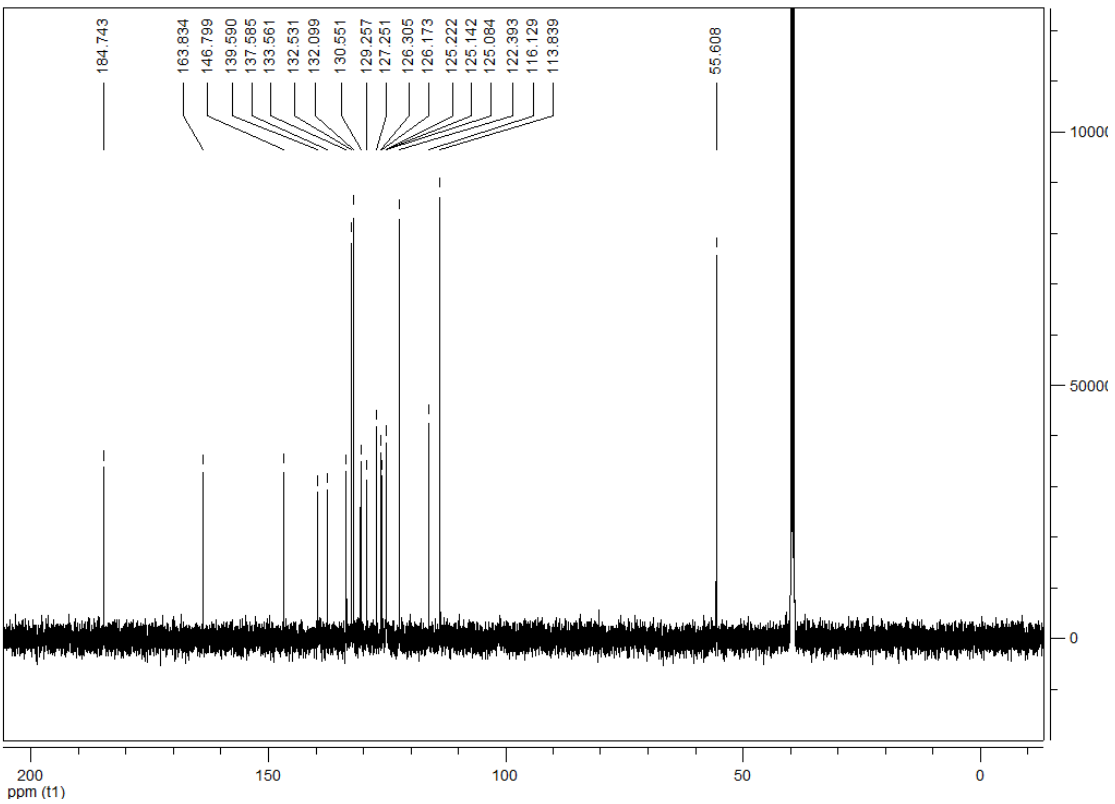
184.743
163.834
146.799
139.590
137.585
133.561
132.531
132.099
130.551
129.257
127.251
126.305
126.173
125.222
125.142
125.084
122.393
116.129
113.839
55.608
200
150
100
50
0
ppm (t1)

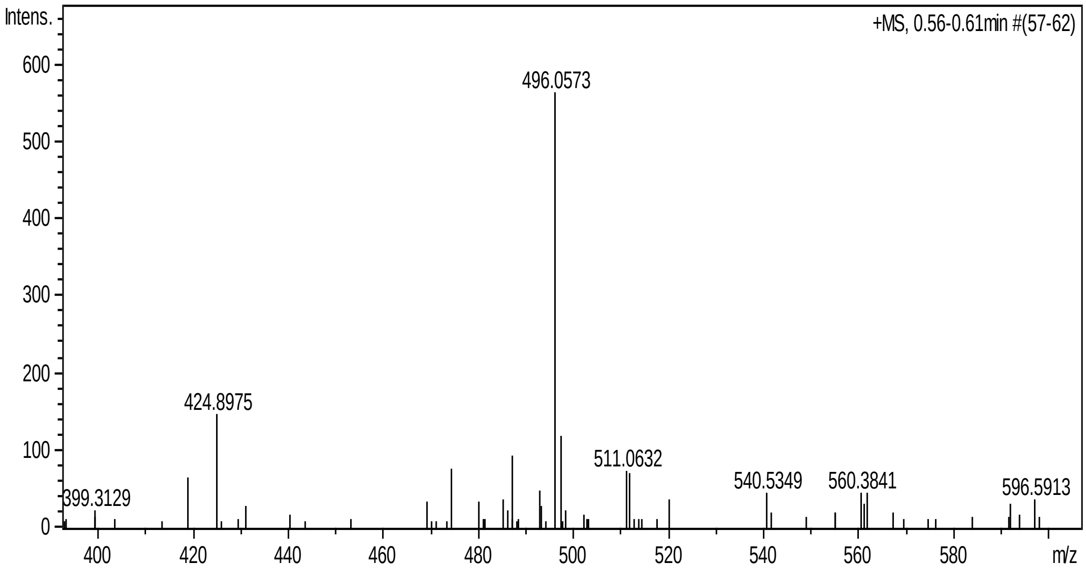
Intens.
+MS, 0.56-0.61min #(57-62)
496.0573
424.8975
511.0632
540.5349
560.3841
596.5913
399.3129
m/z

(4-methoxyphenyl)(3-nitro-2-(3-nitrophenyl)benzo[d]pyrrolo[2,1-b]thiazol-1-yl)methanone (3n): yellow solid, 80%, m.p. 199~201℃; ^{1}H NMR (600 MHz, DMSO-d_6) δ: 8.28 (d, J = 7.2 Hz, 1H, ArH), 8.06 (d, J = 8.4 Hz, 2H, ArH), 7.74~7.71 (m, 3H, ArH), 7.59~7.56 (m, 4H, ArH), 6.81 (d, J = 9.0 Hz, 2H, ArH), 3.73 (s, 3H, OCH_3); ^{13}C NMR (150 MHz, DMSO-d_6) δ: 184.7, 163.8, 146.7, 139.5, 137.5, 133.5, 132.5, 132.0, 130.5, 129.2, 127.2, 126.2, 126.1, 125.2, 125.1, 125.0, 122.3, 116.1, 113.8, 55.6; IR (KBr) υ: 3078, 1636, 1596, 1508, 1468, 1386, 1344, 1295, 1256, 1196, 1160, 1107, 1022, 921, 839, 764, 726 cm^{-1}; MS (m/z): HRMS (ESI) Calcd. for $C_{24}H_{15}N_3NaO_6S$ ([M+Na]$^+$): 496.0574. Found: 496.0570.

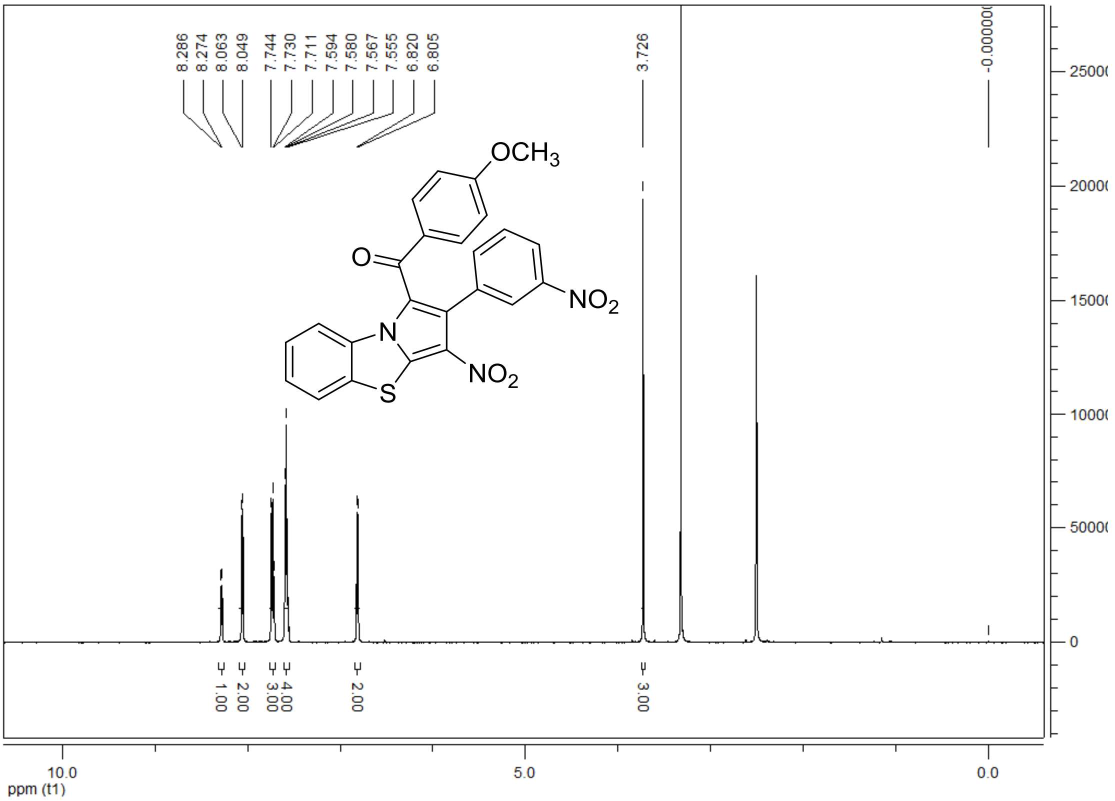

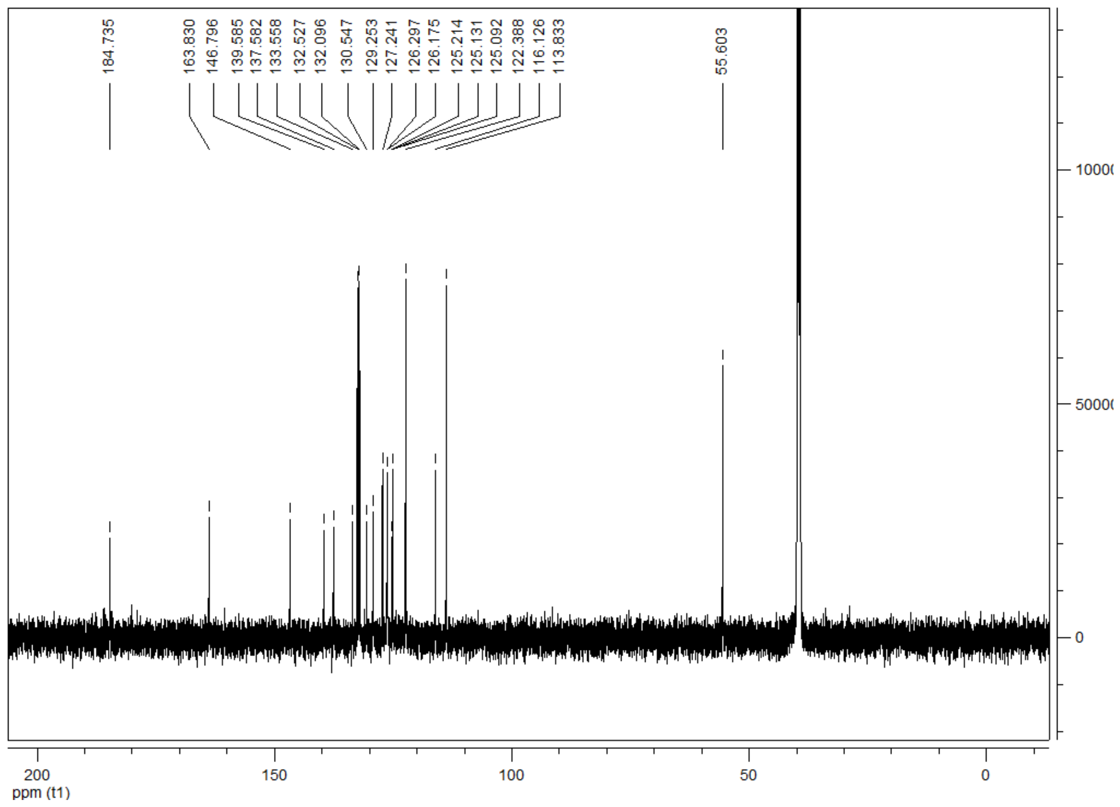
184.735
163.830
146.796
139.585
137.582
133.558
132.527
132.096
130.547
129.253
127.241
126.297
126.175
125.214
125.131
125.092
122.388
116.126
113.833
55.603
ppm (t1)

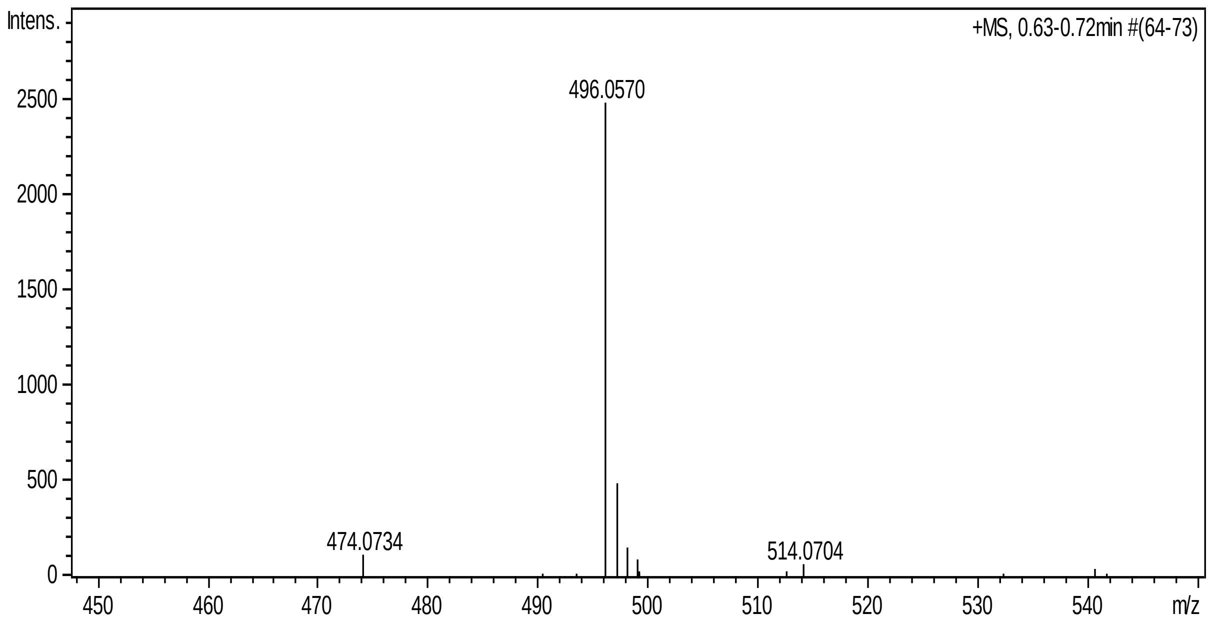
Intens.
+MS, 0.63-0.72min #(64-73)
496.0570
474.0734
514.0704
m/z

(3-nitro-2-(p-tolyl)benzo[d]pyrrolo[2,1-b]thiazol-1-yl)(phenyl)methanone (3o):

yellow solid, 84%, m.p. 186~188℃; ^{1}H NMR (600 MHz, DMSO-d_6) δ: 8.25 (d, J = 7.8 Hz, 1H, ArH), 7.77 (d, J = 8.4 Hz, 1H, ArH), 7.71 (d, J = 7.8 Hz, 2H, ArH), 7.58~7.54 (m, 2H, ArH), 7.46~7.43 (m, 1H, ArH), 7.27~7.24 (m, 2H, ArH), 7.13 (d, J = 7.8 Hz, 2H, ArH), 6.95 (d, J = 7.8 Hz, 2H, ArH), 2.17 (s, 3H, CH_3); ^{13}C NMR (150 MHz, DMSO-d_6) δ: 186.8, 139.7, 137.3, 136.7, 133.8, 133.2, 130.5, 130.4, 129.7, 129.6, 128.1, 127.8, 127.0, 126.9, 126.0, 125.2, 124.8, 124.7, 116.2, 20.6; IR (KBr) υ: 3068, 1782, 1630, 1585, 1509, 1462, 1394, 1339, 1273, 1190, 1168, 1047, 1005, 917, 843, 800, 745 cm^{-1}; MS (m/z): HRMS (ESI) Calcd. for $C_{24}H_{16}N_2NaO_3S$ ([M+Na]$^+$): 435.0774. Found: 435.0772.

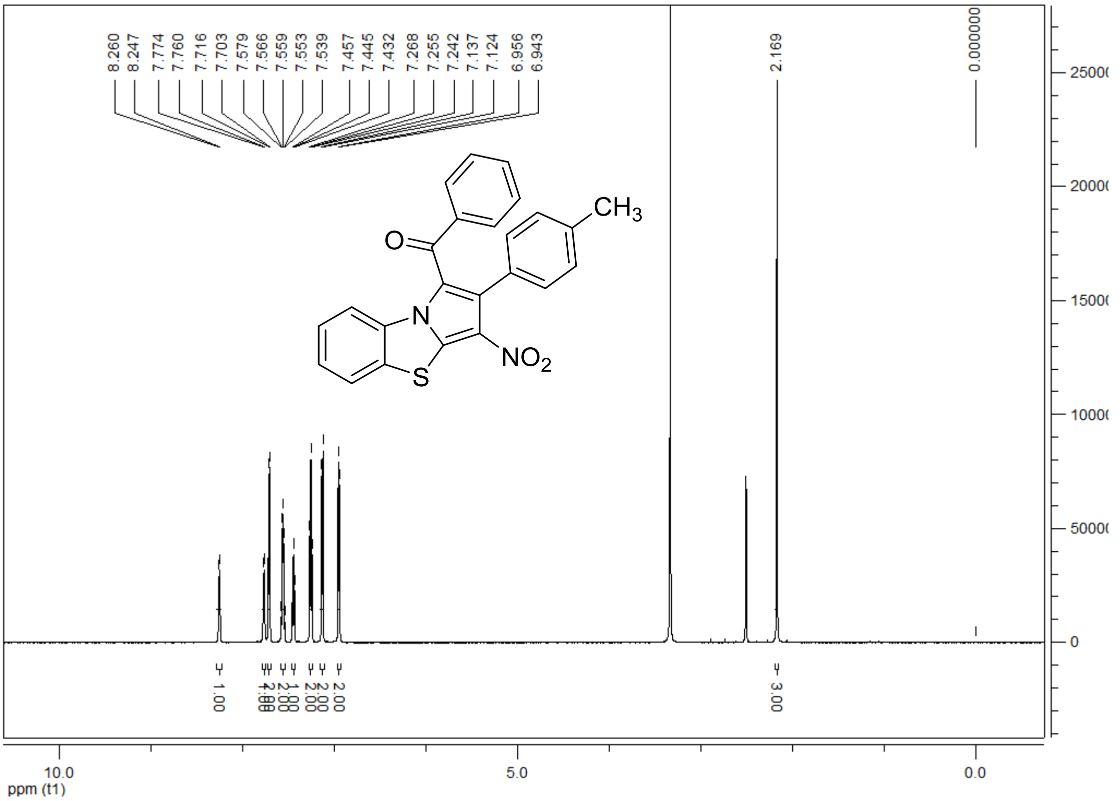

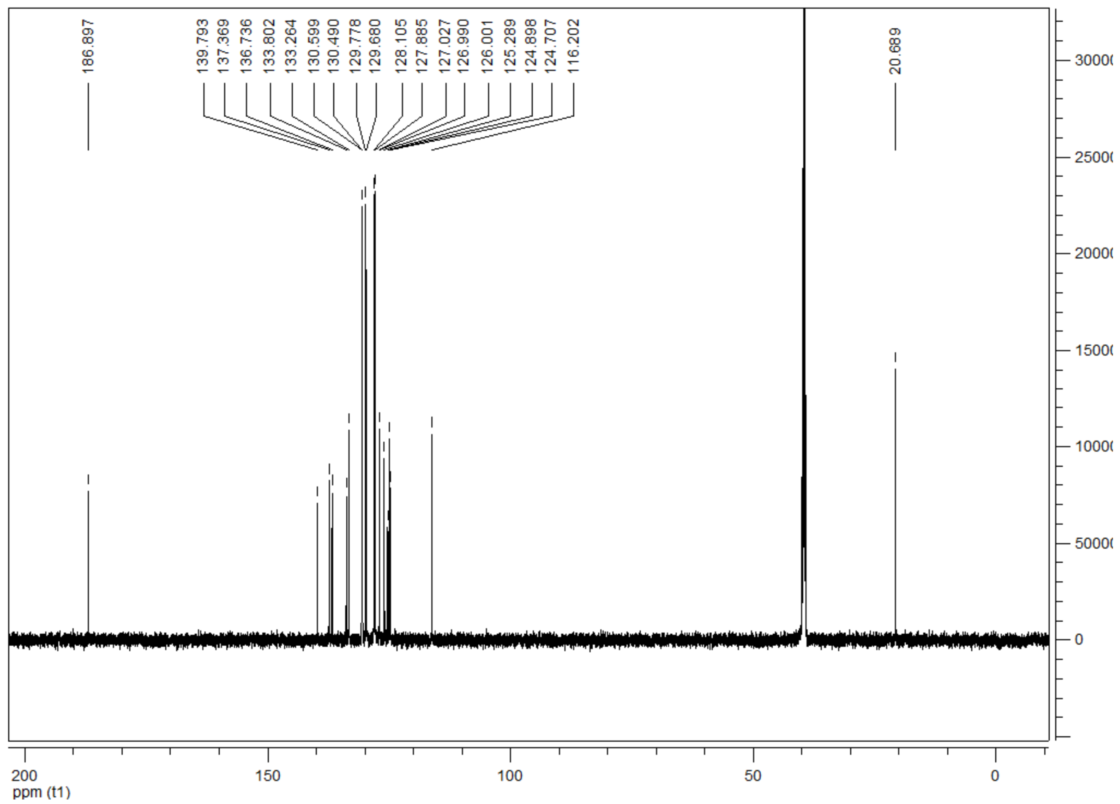
186.897
139.793
137.369
136.736
133.802
133.264
130.599
130.490
129.778
129.680
128.105
127.885
127.027
126.990
126.001
125.289
124.898
124.707
116.202
20.689
200
150
100
50
0
ppm (t1)

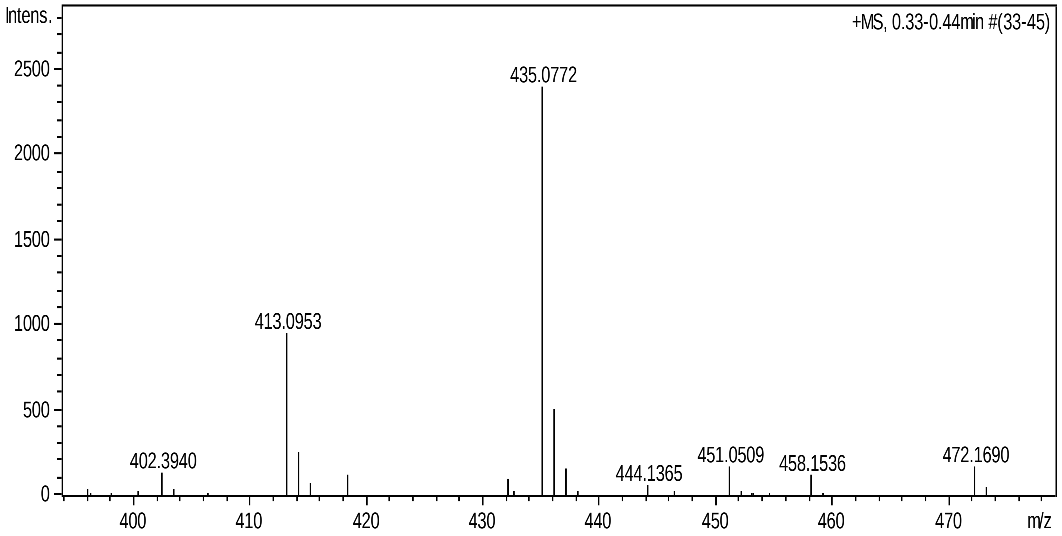
Intens.
+MS, 0.33-0.44min #(33-45)
435.0772
413.0953
402.3940
444.1365
451.0509
458.1536
472.1690
m/z

(2-(4-methoxyphenyl)-3-nitrobenzo[d]pyrrolo[2,1-b]thiazol-1-yl)(phenyl)methanone (3p): yellow solid, 83%, m.p. 149~151℃; ^{1}H NMR (600 MHz, DMSO-d_6) δ: 8.26~8.24 (m, 1H, ArH), 7.81~7.79 (m, 1H, ArH), 7.70 (d, J = 7.2 Hz, 2H, ArH), 7.57~7.56 (m, 2H, ArH), 7.46~7.43 (m, 1H, ArH), 7.27~7.24 (m, 2H, ArH), 7.16 (d, J = 8.4 Hz, 2H, ArH), 6.69 (d, J = 8.4 Hz, 2H, ArH), 3.64 (s, 3H, OCH_3); ^{13}C NMR (150 MHz, DMSO-d_6) δ: 186.9, 159.0, 139.9, 136.7, 133.8, 133.2, 132.1, 130.4, 129.7, 129.6, 128.0, 127.0, 126.0, 125.3, 124.9, 124.7, 122.0, 116.2, 112.8, 55.0; IR (KBr) υ: 3063, 2829, 1694, 1638, 1511, 1465, 1389, 1341, 1249, 1170, 1015, 918, 846, 805, 744 cm^{-1}; MS (m/z): HRMS (ESI) Calcd. for $C_{24}H_{16}N_2NaO_4S$ ($[M+Na]^+$): 451.0723. Found: 451.0725.

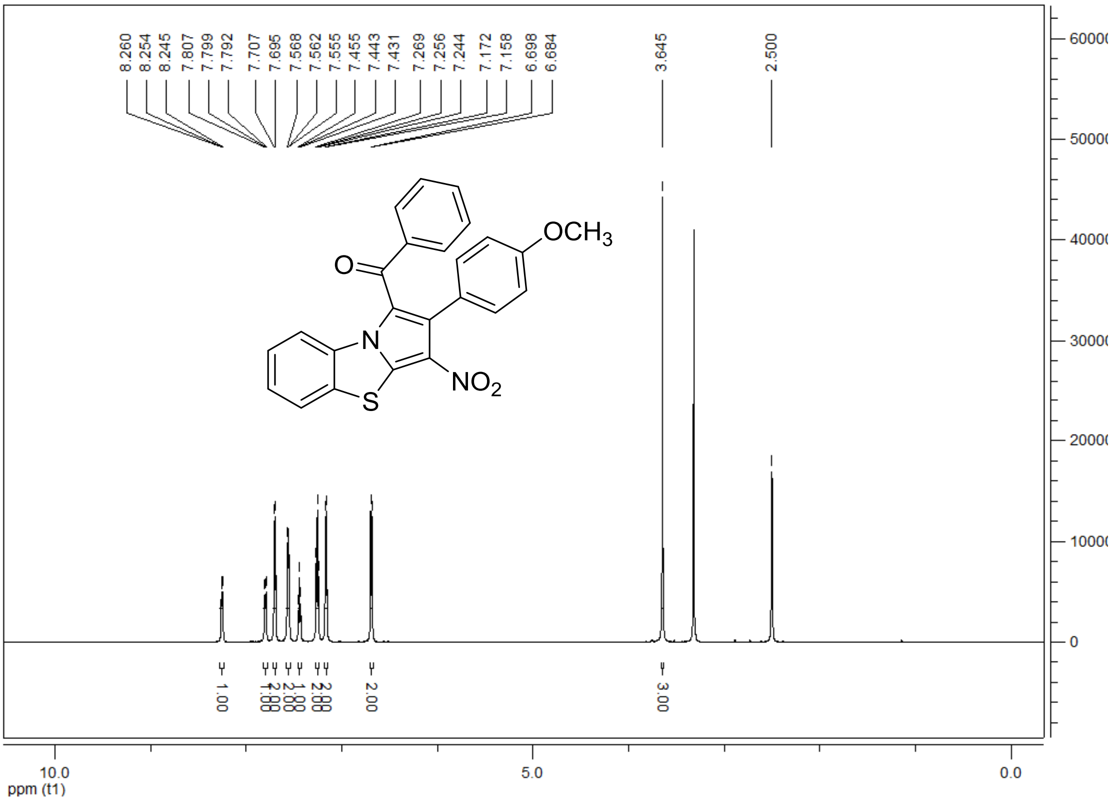

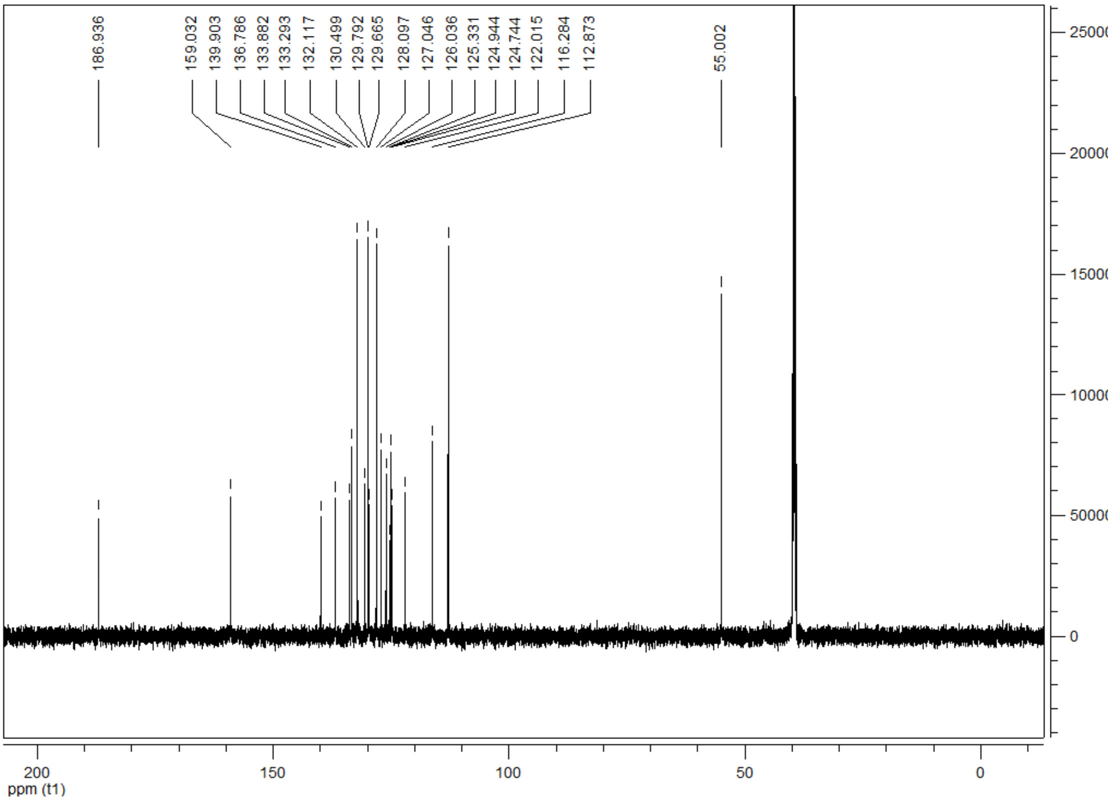
186.936
159.032
139.903
136.786
133.882
133.293
132.117
130.499
129.792
129.665
128.097
127.046
126.036
125.331
124.944
124.744
122.015
116.284
112.873
55.002
200
150
100
50
0
ppm (t1)

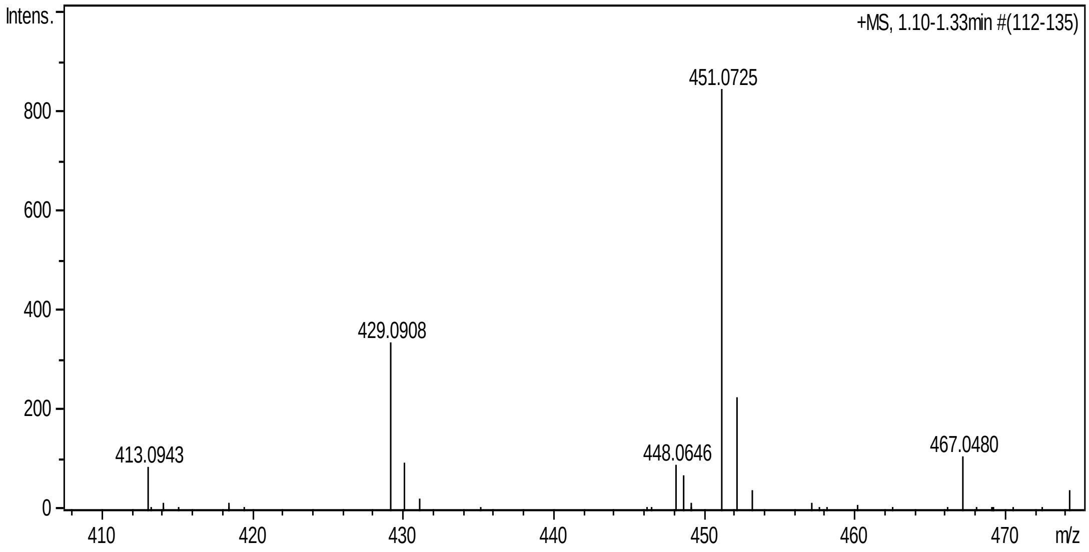
Intens.
+MS, 1.10-1.33min #(112-135)
451.0725
429.0908
413.0943
448.0646
467.0480
m/z

(2-(4-chlorophenyl)-3-nitrobenzo[d]pyrrolo[2,1-b]thiazol-1-yl)(phenyl)methanone (3q): yellow solid, 80%, m.p. 220~222℃; ^{1}H NMR (400 MHz, DMSO-d_6) δ: 8.28~8.26 (m, 1H, ArH), 7.84~7.82 (m, 1H, ArH), 7.70 (d, J = 7.6 Hz, 2H, ArH), 7.59~7.55 (m, 2H, ArH), 7.49~7.45 (m, 1H, ArH), 7.29~7.26 (m, 4H, ArH), 7.20~7.18 (m, 2H, ArH); ^{13}C NMR (100 MHz, DMSO-d_6) δ: 186.5, 139.9, 136.6, 133.7, 133.3, 132.8, 132.3, 130.4, 129.7, 128.9, 128.4, 128.0, 127.2, 127.0, 126.1, 125.2, 124.9, 124.9, 116.3; IR (KBr) υ: 3054, 1788, 1641, 1591, 1536, 1500, 1462, 1399, 1335, 1272, 1165, 1086, 1055, 1006, 917, 852, 793, 749, 700 cm^{-1}; MS (m/z): HRMS (ESI) Calcd. for $C_{23}H_{13}ClN_2NaO_3S$ ([M+Na]$^{+}$): 455.0228. Found: 455.0222.

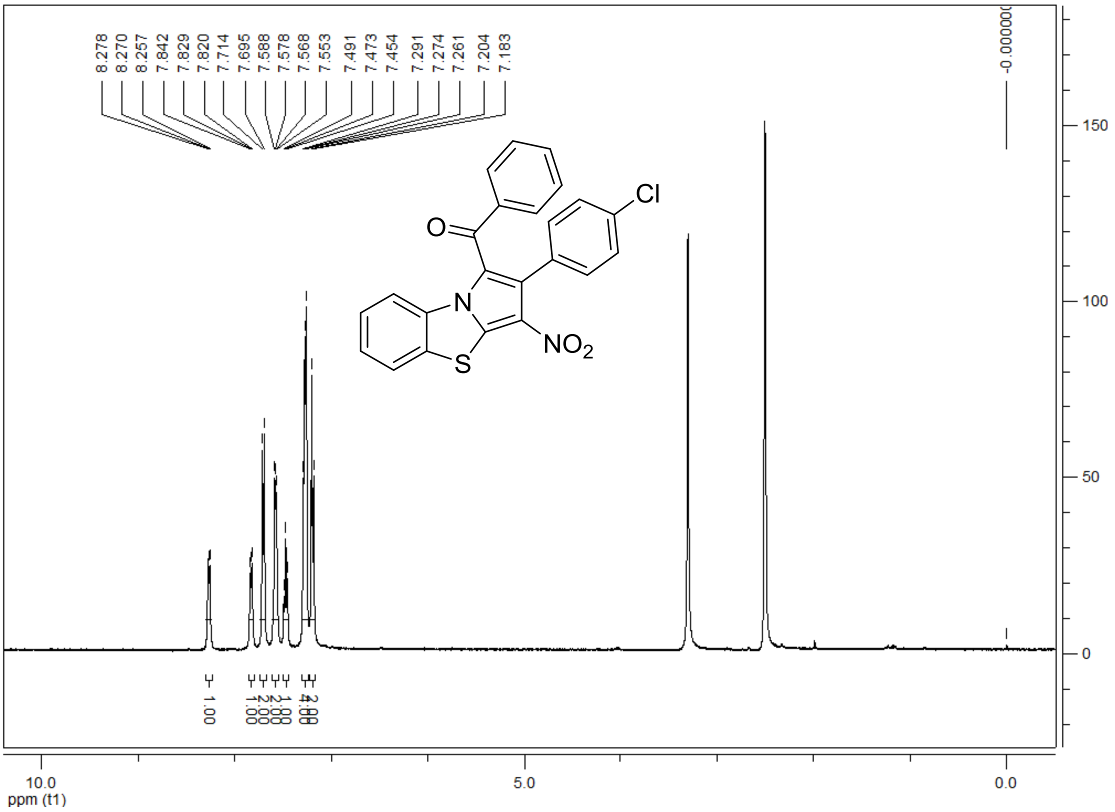

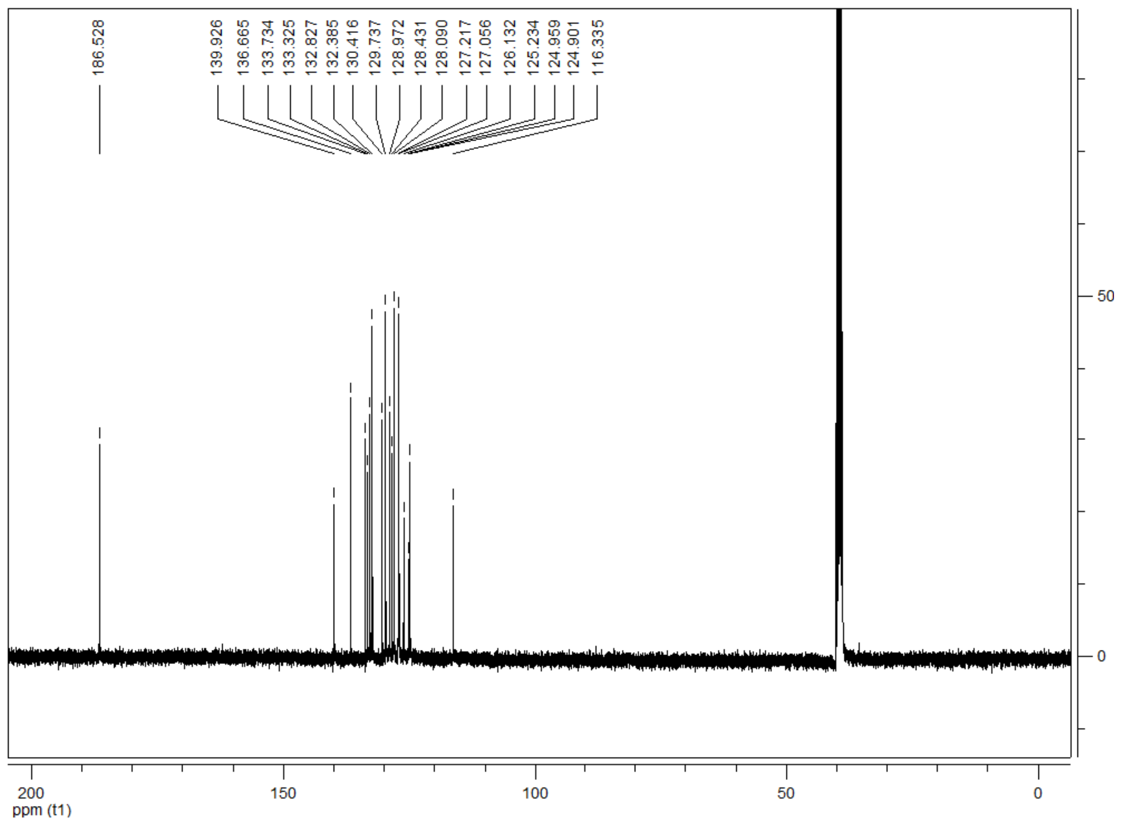
186.528
139.926
136.665
133.734
133.325
132.827
132.385
130.416
129.737
128.972
128.431
128.090
127.217
127.056
126.132
125.234
124.959
124.901
116.335
50
0
200
150
100
50
0
ppm (t1)

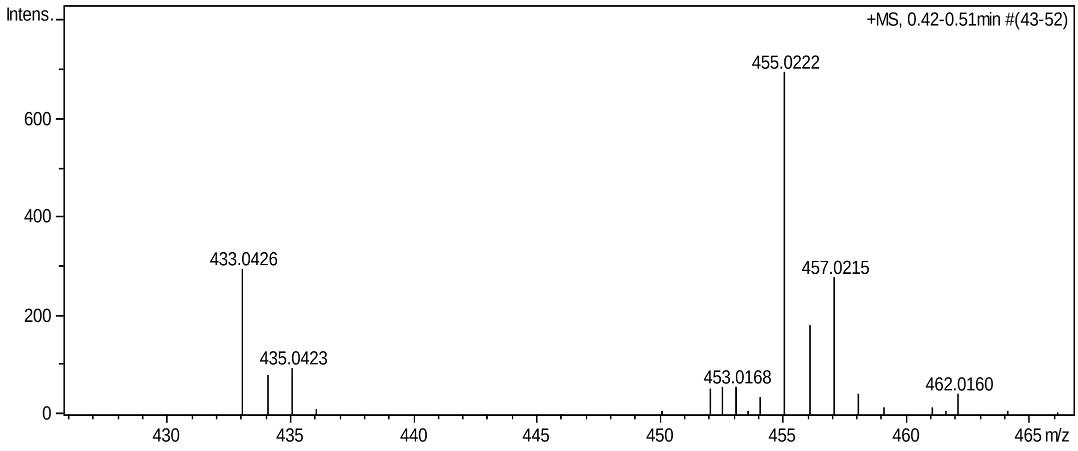
Intens.
+MS, 0.42-0.51min #(43-52)
455.0222
433.0426
457.0215
435.0423
453.0168
462.0160
600
400
200
0
430
435
440
445
450
455
460
465 m/z

4-fluorophenyl)(2-(4-methoxyphenyl)-3-nitrobenzo[d]pyrrolo[2,1-b]thiazol-1-yl)methanone (3s): yellow solid, 83%, m.p. 190~192℃; ^{1}H NMR (600 MHz, DMSO-d_6) δ: 8.26~8.25 (m, 1H, ArH), 7.86~7.85 (m, 1H, ArH), 7.78~7.76 (m, 2H, ArH), 7.58~7.57 (m, 2H, ArH), 7.16 (d, J = 7.2 Hz, 2H, ArH), 7.09~7.06 (m, 2H, ArH), 6.72 (d, J = 7.8 Hz, 2H, ArH), 3.67 (s, 3H, OCH_3); ^{13}C NMR (150 MHz, DMSO-d_6) δ: 185.4, 165.6, 163.9, 159.1, 140.1, 133.9, 133.5, 132.8, 132.1, 130.4, 129.9, 127.0, 126.0, 125.3, 124.9, 124.6, 121.9, 116.4, 115.2, 115.0, 112.9, 55.0; IR (KBr) υ: 2832, 1641, 1601, 1509, 1473, 1410, 1342, 1262, 1167, 1013, 921, 834, 748, 704 cm^{-1}; MS (m/z): HRMS (ESI) Calcd. for $C_{24}H_{15}FN_2NaO_4S$ ([M+Na]$^+$): 469.0629. Found: 469.0618.

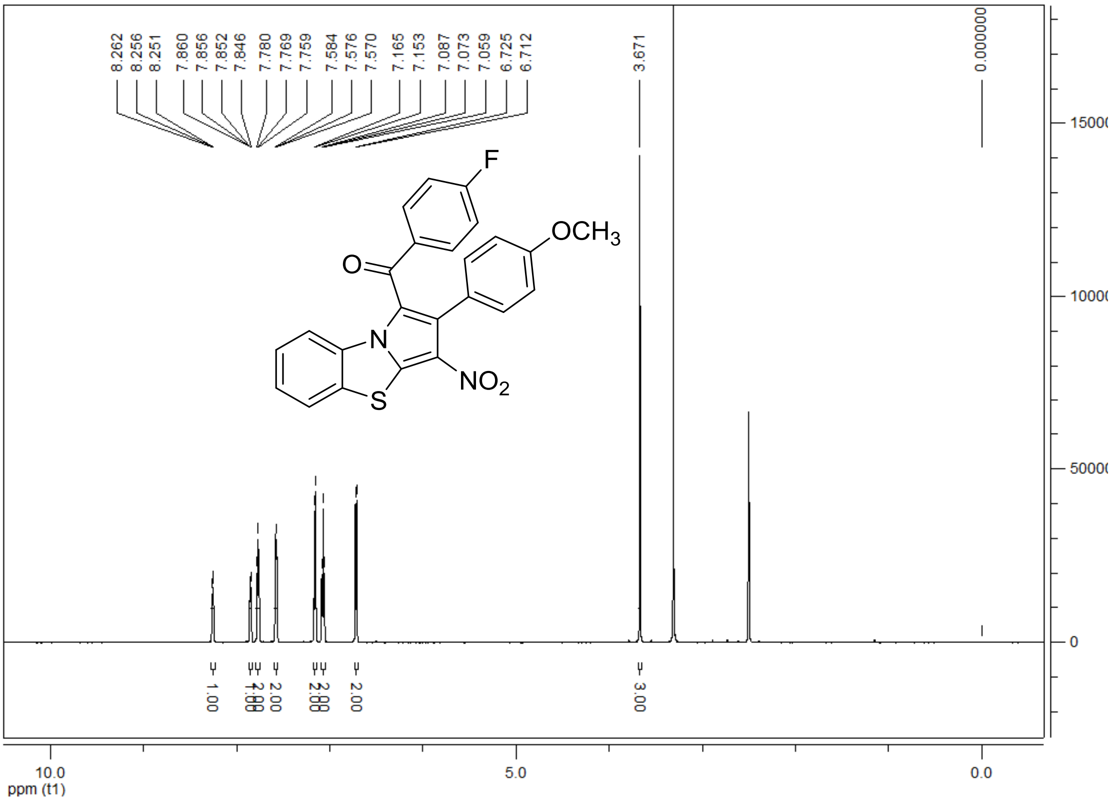

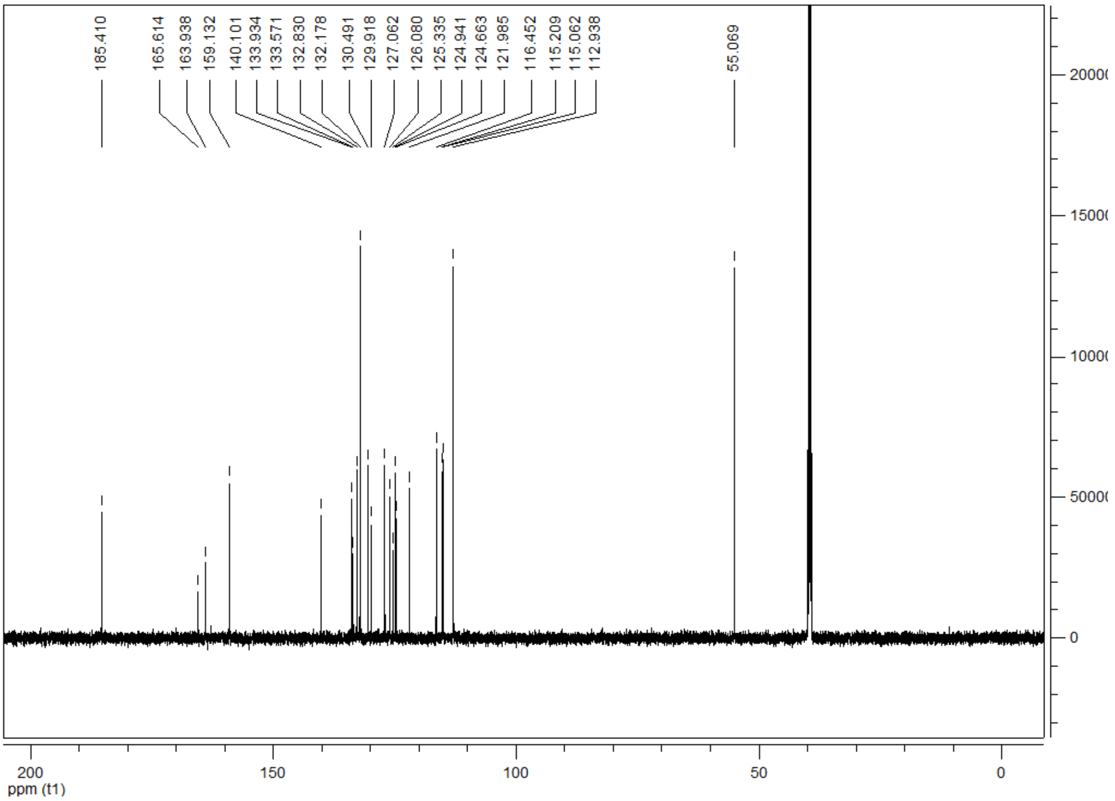
185.410
165.614
163.938
159.132
140.101
133.934
133.571
132.830
132.178
130.491
129.918
127.062
126.080
125.335
124.941
124.663
121.985
116.452
115.209
115.062
112.938
55.069
ppm (t1)

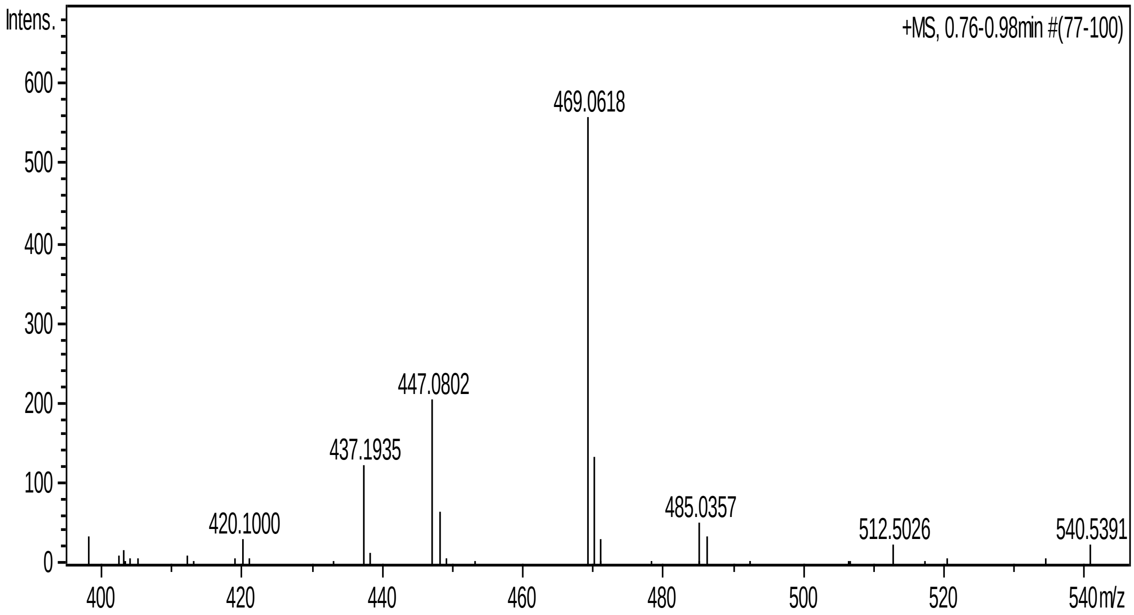
Intens.
+MS, 0.76-0.98min #(77-100)
469.0618
447.0802
437.1935
420.1000
485.0357
512.5026
540.5391
m/z

(4-chlorophenyl)(3-nitro-2-(p-tolyl)benzo[d]pyrrolo[2,1-b]thiazol-1-yl)methanone (3t): yellow solid, 82%, m.p. 205~207℃; ^{1}H NMR (600 MHz, DMSO-d_6) δ: 8.27~8.25 (m, 1H, ArH), 7.88~7.86 (m, 1H, ArH), 7.67 (d, *J* = 8.4 Hz, 2H, ArH), 7.59~7.57 (m, 2H, ArH), 7.29 (d, *J* = 8.4 Hz, 2H, ArH), 7.11 (d, *J* = 7.8 Hz, 2H, ArH), 6.97 (d, *J* = 7.8 Hz, 2H, ArH), 2.20 (s, 3H, CH_3); ^{13}C NMR (150 MHz, DMSO-d_6) δ: 185.4, 140.1, 137.8, 137.4, 135.4, 133.8, 131.3, 130.5, 130.3, 130.2, 127.9, 127.7, 126.9, 126.8, 125.9, 125.1, 124.8, 124.5, 116.4, 20.5; IR (KBr) υ: 3061, 1637, 1582, 1514, 1475, 1395, 1341, 1271, 1163, 1087, 1008, 916, 836, 745 cm^{-1}; MS (*m/z*): HRMS (ESI) Calcd. for $C_{24}H_{15}ClN_2NaO_3S$ ([M+Na]$^+$): 469.0384. Found: 469.0380.

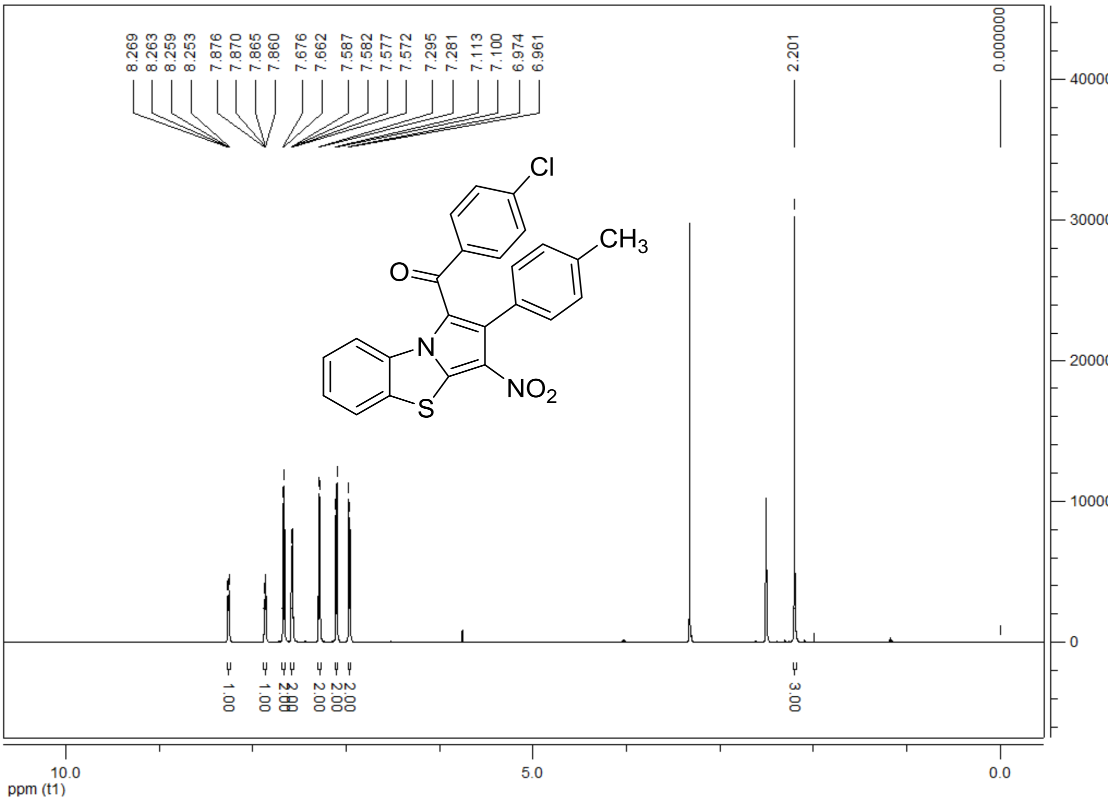

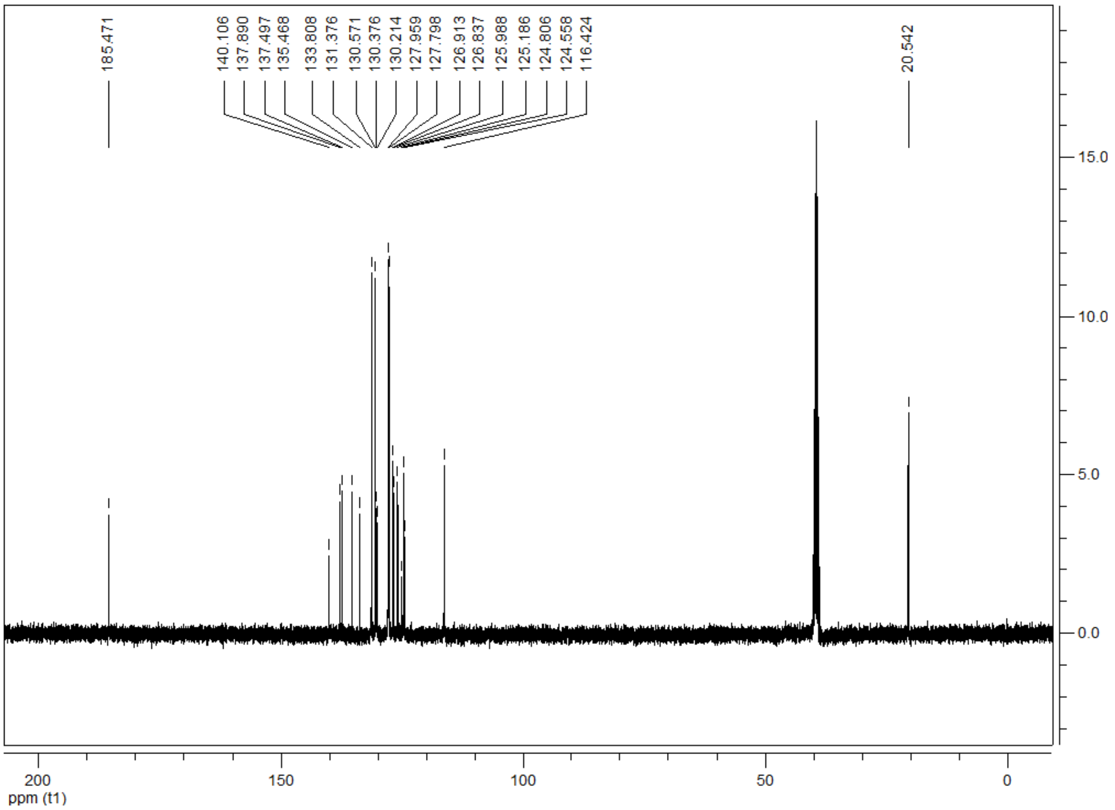
185.471
140.106
137.890
137.497
135.468
133.808
131.376
130.571
130.376
130.214
127.959
127.798
126.913
126.837
125.988
125.186
124.806
124.558
116.424
20.542
15.0
10.0
5.0
0.0
200
150
100
50
0
ppm (t1)

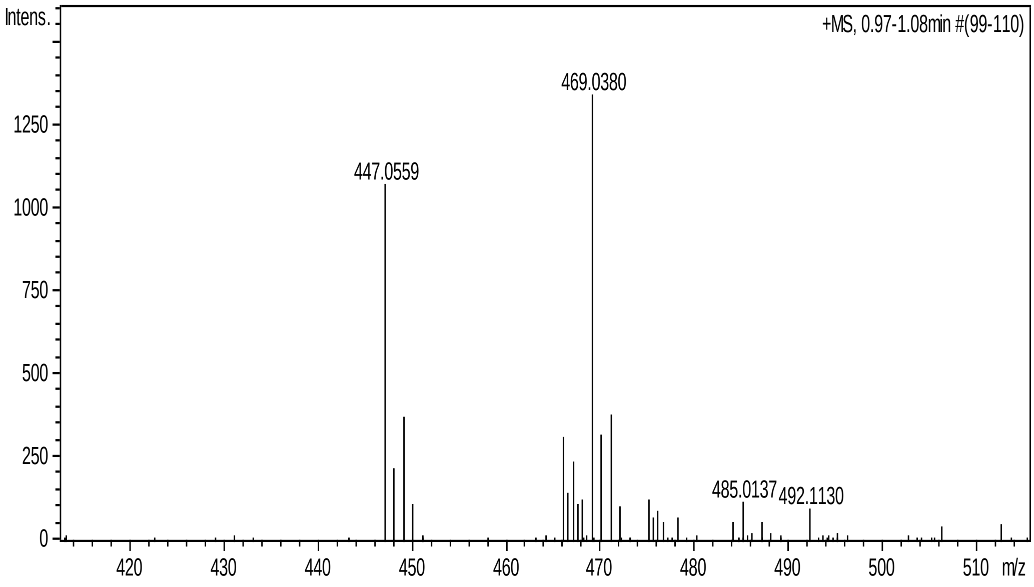
Intens.
+MS, 0.97-1.08min #(99-110)
469.0380
447.0559
485.0137
492.1130
1250
1000
750
500
250
0
420
430
440
450
460
470
480
490
500
510
m/z

(4-fluorophenyl)(3-nitro-2-(p-tolyl)benzo[d]pyrrolo[2,1-b]thiazol-1-yl)methanone (3r):

yellow solid, 80%, m.p. 213~215℃; ^{1}H NMR (600 MHz, DMSO-*d*$_6$) δ: 8.27~8.26 (m, 1H, ArH), 7.83~7.82 (m, 1H, ArH), 7.79~7.76 (m, 2H, ArH), 7.58~7.57 (m, 2H, ArH), 7.12 (d, *J* = 7.2 Hz, 2H, ArH), 7.08~7.06 (m, 2H, ArH), 6.97 (d, *J* = 7.8 Hz, 2H, ArH), 2.20 (s, 3H, CH_3); ^{13}C NMR (150 MHz, DMSO-*d*$_6$) δ: 185.4, 140.0, 137.5, 133.9, 133.5, 132.8, 130.6, 130.5, 129.9, 127.9, 127.1, 127.0, 126.1, 125.3, 125.0, 124.6, 116.4, 115.2, 115.1, 20.6; IR (KBr) υ: 3069, 1638, 1593, 1515, 1477, 1392, 1340, 1278, 1173, 1099, 1009, 920, 860, 844, 818, 786, 752, 738, 704 cm^{-1}; MS (*m*/*z*): HRMS (ESI) Calcd. for $C_{24}H_{15}FN_2NaO_3S$ ([M+Na]$^+$): 453.0680. Found: 453.0680.

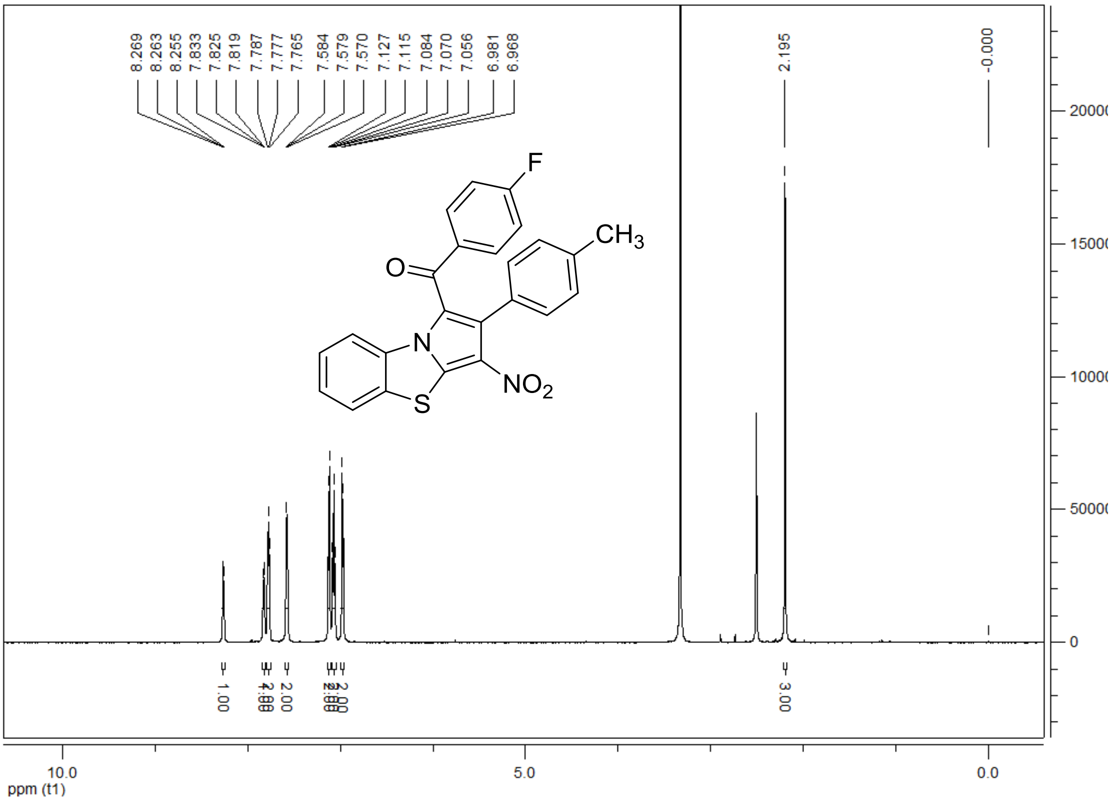

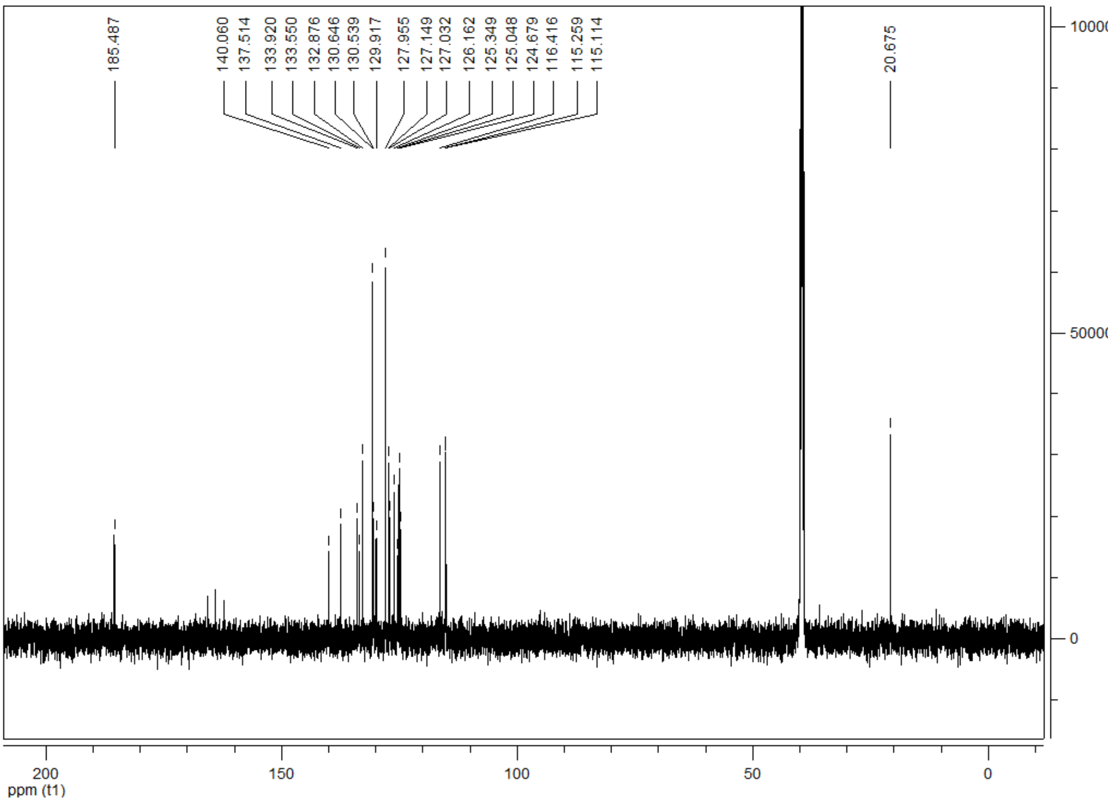
185.487
140.060
137.514
133.920
133.550
132.876
130.646
130.539
129.917
127.955
127.149
127.032
126.162
125.349
125.048
124.679
116.416
115.259
115.114
20.675
ppm (t1)

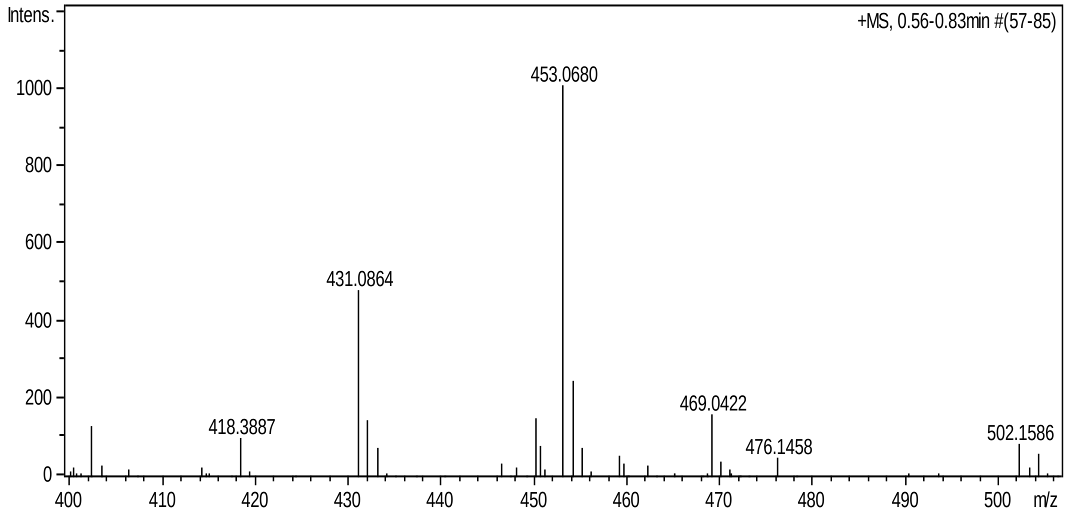
Intens.
+MS, 0.56-0.83min #(57-85)
453.0680
431.0864
418.3887
469.0422
476.1458
502.1586
m/z

(4-chlorophenyl)(2-(4-methoxyphenyl)-3-nitrobenzo[d]pyrrolo[2,1-b]thiazol-1-yl)methanone (3u): yellow solid, 87%, m.p. 183~185℃; ^{1}H NMR (600 MHz, DMSO-d_6) δ: 8.26~8.25 (m, 1H, ArH), 7.90~7.88 (m, 1H, ArH), 7.66 (d, J = 8.4 Hz, 2H, ArH), 7.58~7.57 (m, 2H, ArH), 7.29 (d, J = 8.4 Hz, 2H, ArH), 7.14 (d, J = 8.4 Hz, 2H, ArH), 6.71 (d, J = 8.4 Hz, 2H, ArH), 3.67 (s, 3H, OCH_3); ^{13}C NMR (150 MHz, DMSO-d_6) δ: 185.6, 159.2, 140.3, 137.9, 135.6, 133.9, 132.2, 131.5, 130.4, 130.3, 128.0, 127.0, 126.1, 125.3, 124.9, 124.7, 121.9, 116.5, 112.9, 55.1; IR (KBr) υ: 3001, 2833, 1697, 1638, 1578, 1509, 1467, 1389, 1333, 1253, 1168, 1084, 1011, 913, 836, 747 cm^{-1}; MS (m/z): HRMS (ESI) Calcd. for $C_{24}H_{15}ClN_2NaO_4S$ ([M+Na]$^+$): 485.0333. Found: 485.0333.

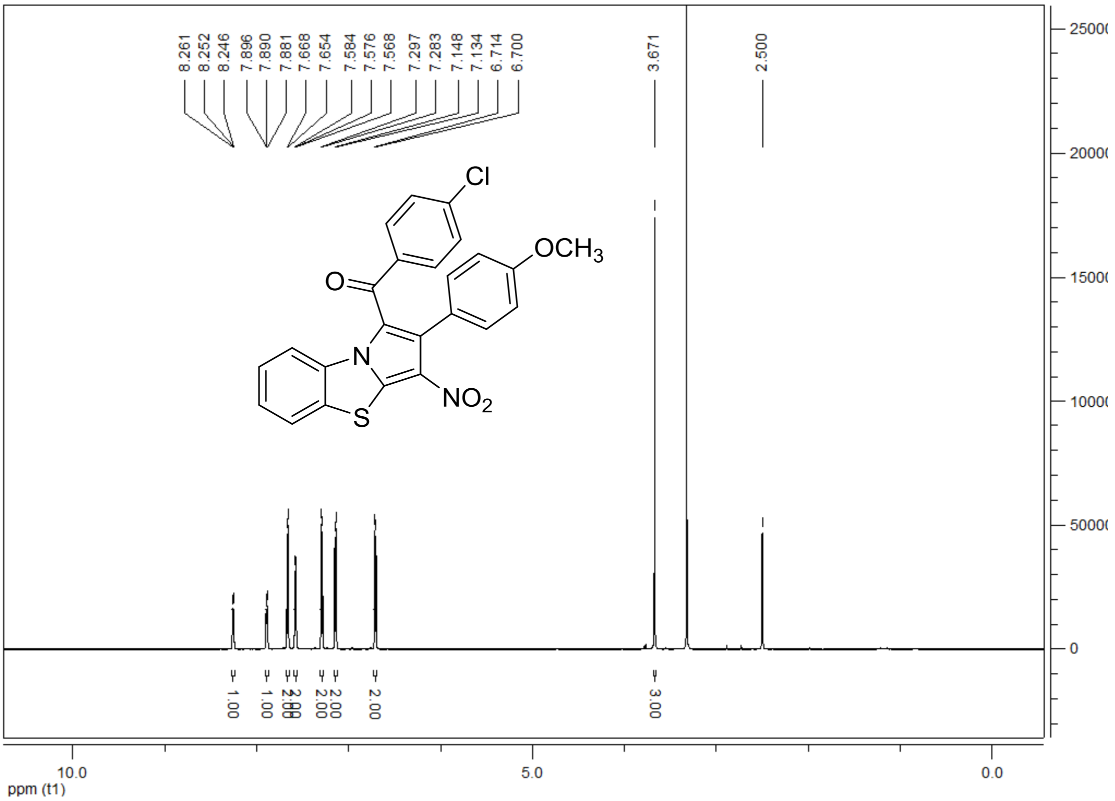

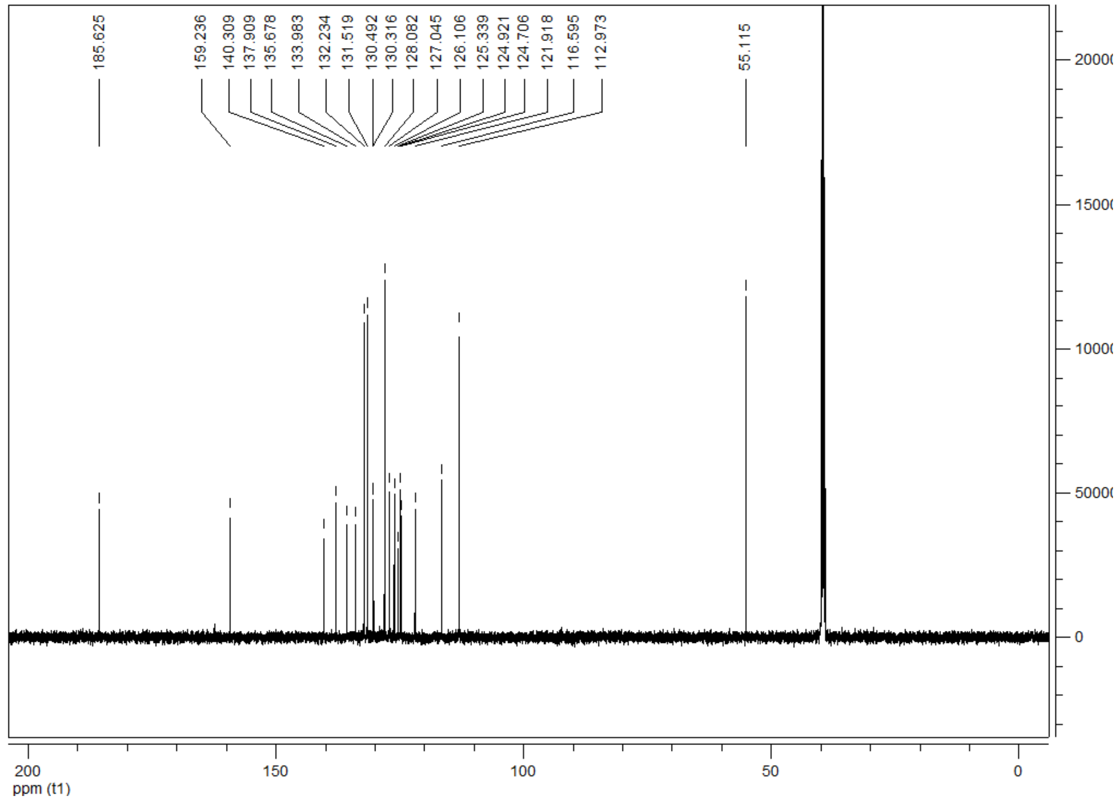

185.625
159.236
140.309
137.909
135.678
133.983
132.234
131.519
130.492
130.316
128.082
127.045
126.106
125.339
124.921
124.706
121.918
116.595
112.973
55.115
200
150
100
50
0
ppm (t1)

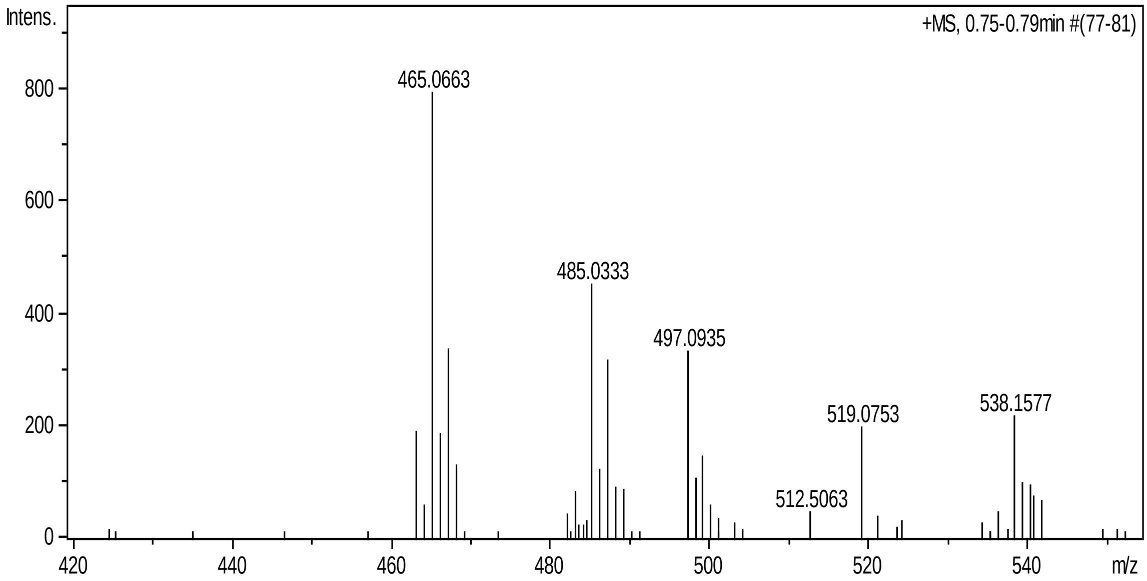

Intens.
+MS, 0.75-0.79min #(77-81)
465.0663
485.0333
497.0935
519.0753
538.1577
512.5063
800
600
400
200
0
420
440
460
480
500
520
540
m/z

ethyl 3-nitro-2-(p-tolyl)benzo[d]pyrrolo[2,1-b]thiazole-1-carboxylate (3v):

yellow solid, 79%, m.p. 177~179℃; ^{1}H NMR (600 MHz, DMSO-d_6) δ: 8.54 (d, J = 8.4 Hz, 1H, ArH), 8.22 (d, J = 7.8 Hz, 1H, ArH), 7.65~7.63 (m, 1H, ArH), 7.60~7.57 (m, 1H, ArH), 7.29~7.24 (m, 4H, ArH), 4.08 (q, J = 7.2 Hz, 2H, CH), 2.40 (s, 3H, CH_3), 0.84 (t, J = 7.2 Hz, 3H, CH_3); ^{13}C NMR (150 MHz, DMSO-d_6) δ: 159.9, 139.6, 137.2, 134.3, 131.3, 130.3, 129.5, 128.4, 127.9, 126.9, 126.1, 125.7, 124.7, 118.4, 117.4, 60.9, 20.8, 13.0; IR (KBr) υ: 2985, 1708, 1647, 1513, 1471, 1400, 1341, 1266, 1165, 1143, 1082, 1020, 873, 753 cm^{-1}; MS (m/z): HRMS (ESI) Calcd. for $C_{20}H_{16}N_2NaO_4S$ ([M+Na]$^+$): 403.0723. Found: 403.0714.

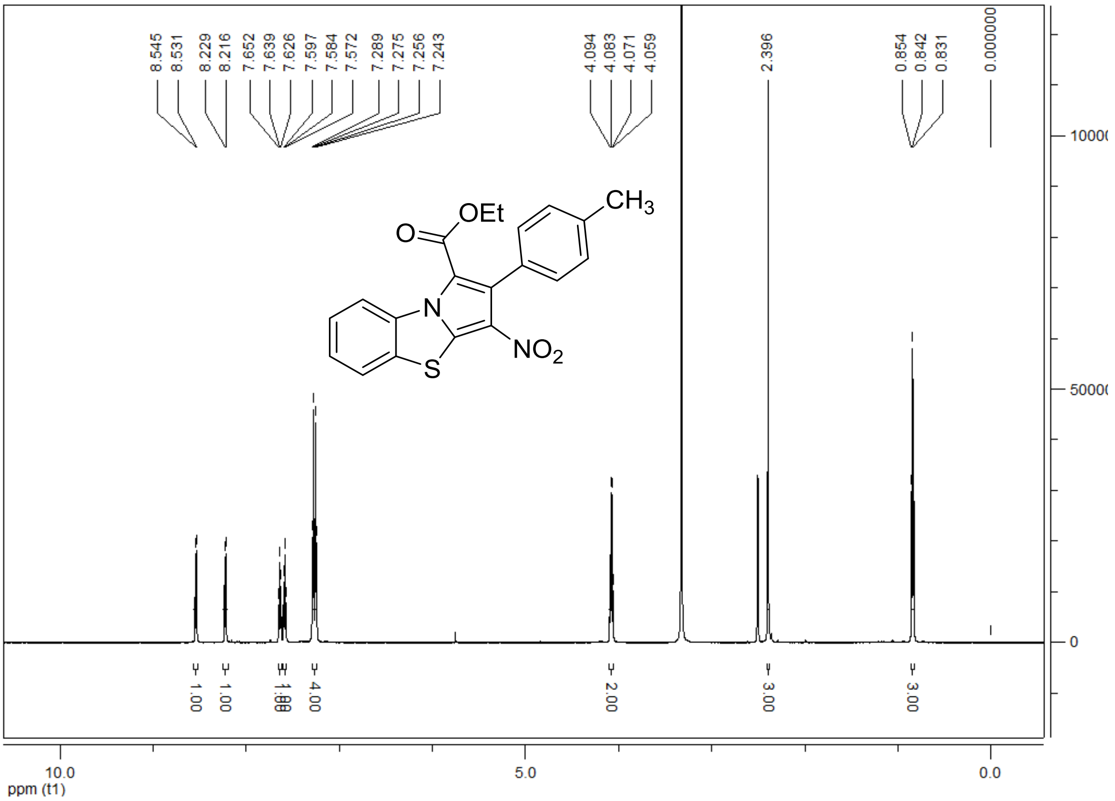

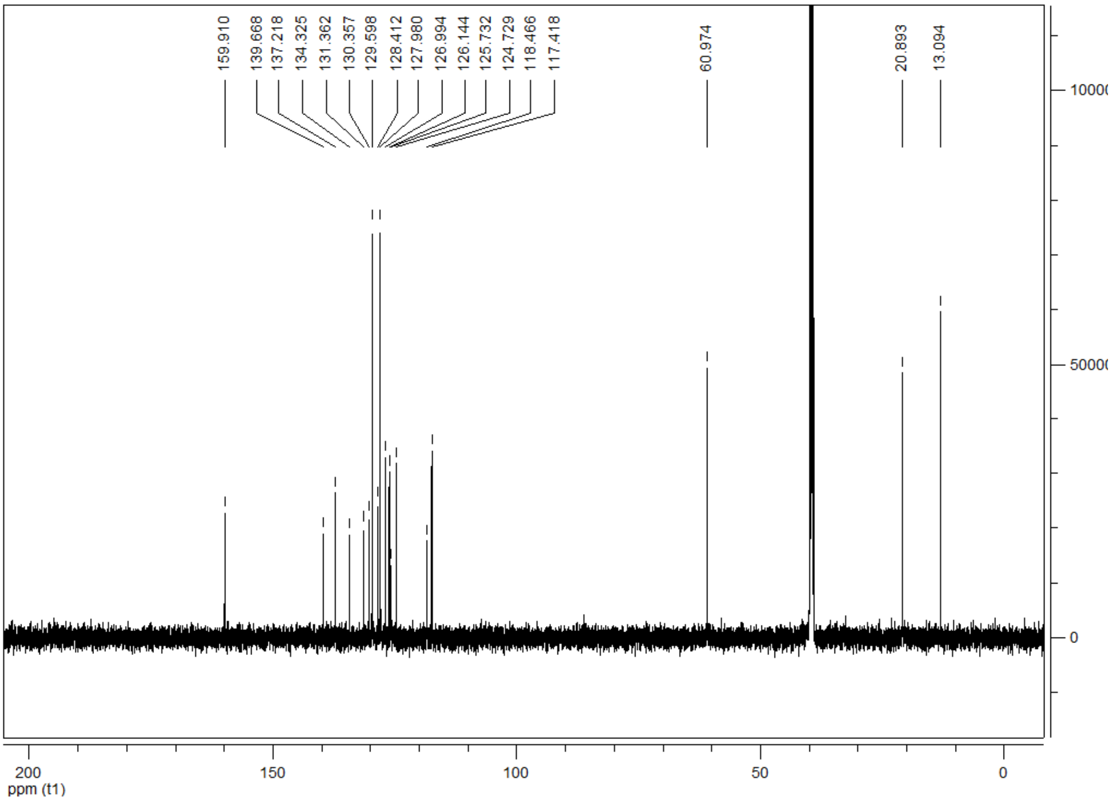
159.910
139.668
137.218
134.325
131.362
130.357
129.598
128.412
127.980
126.994
126.144
125.732
124.729
118.466
117.418
60.974
20.893
13.094
200
150
100
50
0
ppm (t1)

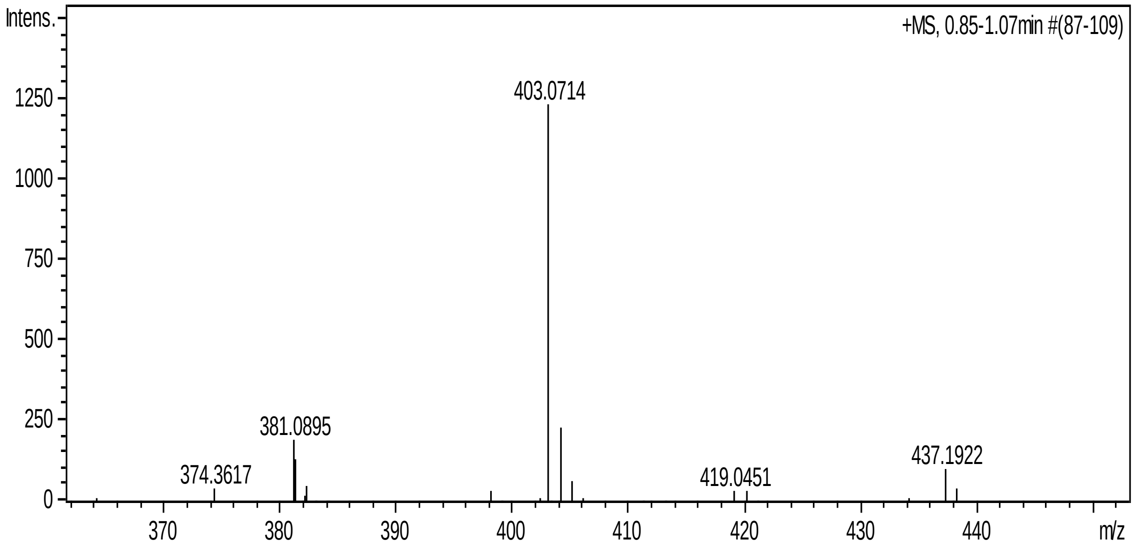
Intens.
+MS, 0.85-1.07min #(87-109)
403.0714
381.0895
374.3617
419.0451
437.1922
1250
1000
750
500
250
0
370
380
390
400
410
420
430
440
m/z

ethyl 2-(4-methoxyphenyl)-3-nitrobenzo[d]pyrrolo[2,1-b]thiazole-1-carboxylate (3w):

yellow solid, 81%, m.p. 169~171℃; ^{1}H NMR (600 MHz, DMSO-d_6) δ: 8.54 (d, J = 8.4 Hz, 1H, ArH), 8.23 (d, J = 7.8 Hz, 1H, ArH), 7.66~7.63 (m, 1H, ArH), 7.60~7.58 (m, 1H, ArH), 7.33 (d, J = 8.4 Hz, 2H, ArH), 7.00 (d, J = 8.4 Hz, 2H, ArH), 4.10 (q, J = 7.2 Hz, 2H, CH), 3.83 (s, 3H, OCH_3), 0.89 (t, J = 7.2 Hz, 3H, CH_3); ^{13}C NMR (150 MHz, DMSO-d_6) δ: 159.9, 159.1, 139.7, 134.3, 131.1, 131.1, 130.3, 126.9, 126.1, 125.7, 124.7, 123.2, 118.4, 117.4, 112.8, 60.9, 55.1, 13.2; IR (KBr) υ: 2981, 2895, 1708, 1610, 1511, 1466, 1393, 1341, 1253, 1164, 1073, 1022, 873, 755 cm^{-1}; MS (m/z): HRMS (ESI) Calcd. for $C_{20}H_{16}N_2NaO_5S$ ([M+Na]$^+$): 419.0672. Found: 419.0662.

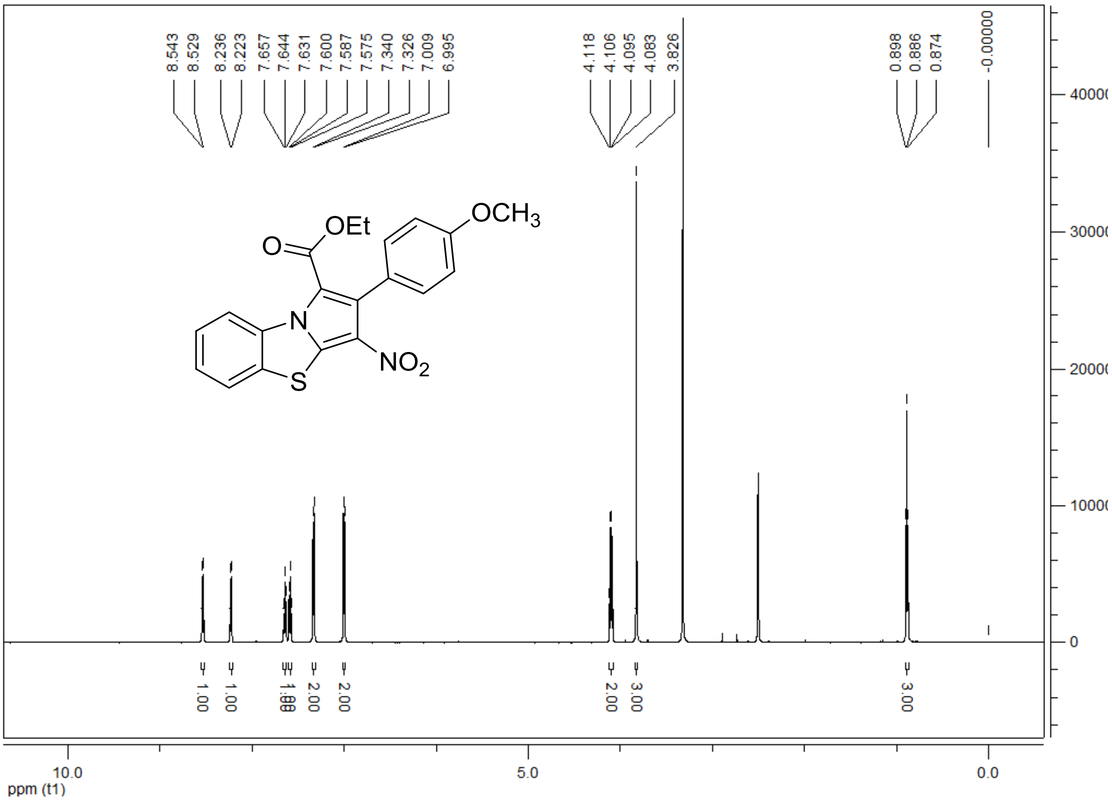

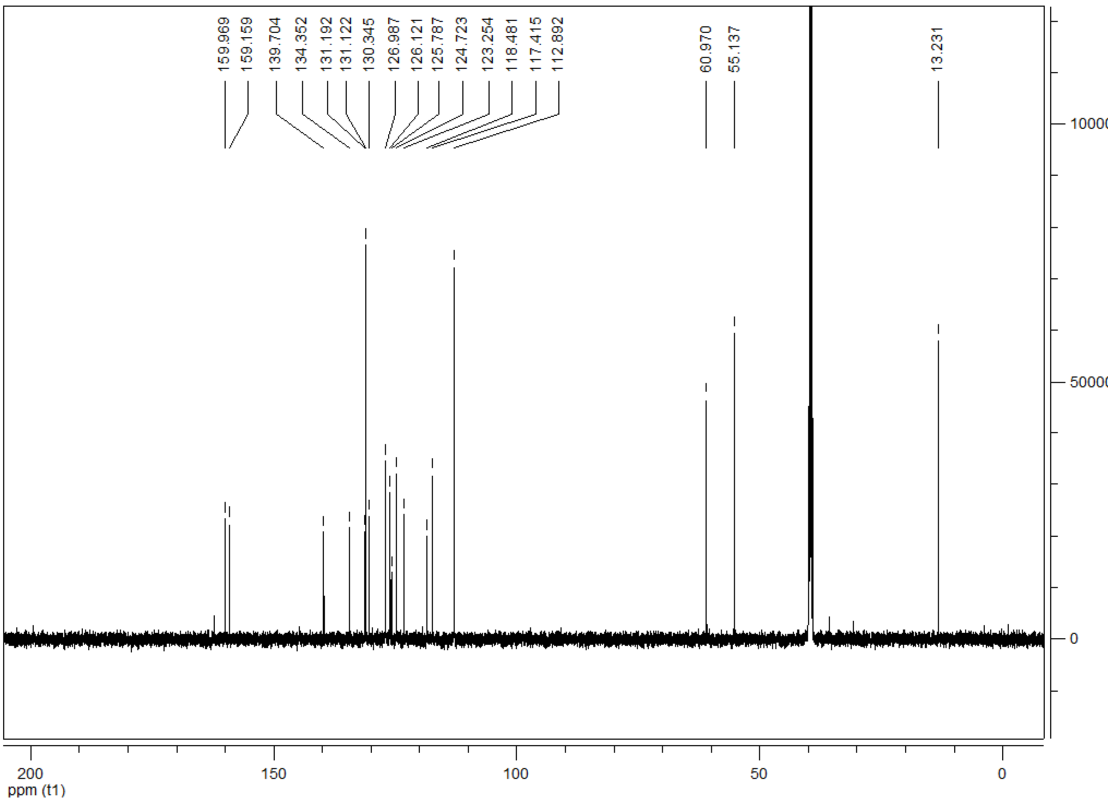

159.969
159.159
139.704
134.352
131.192
131.122
130.345
126.987
126.121
125.787
124.723
123.254
118.481
117.415
112.892
60.970
55.137
13.231
200
150
100
50
0
ppm (t1)

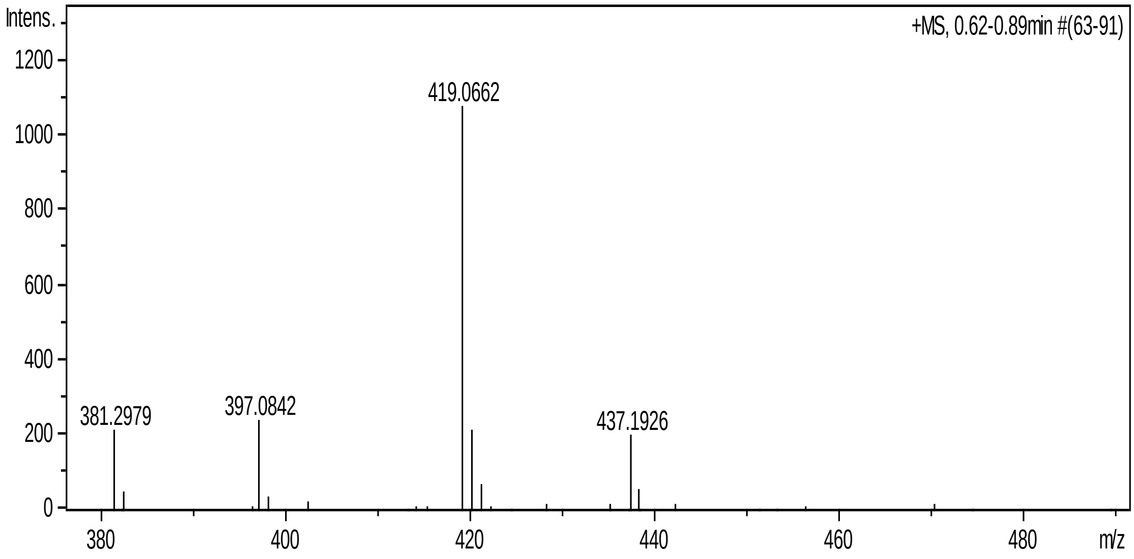

Intens.
+MS, 0.62-0.89min #(63-91)
419.0662
381.2979
397.0842
437.1926
m/z

(2-(4-chlorophenyl)-3-methylbenzo[d]pyrrolo[2,1-b]thiazol-1-yl)(phenyl)methanone (4a): yellow solid, 86%, m.p. 161~163℃; ^{1}H NMR (400 MHz, DMSO-d_6) δ: 8.16~8.14 (m, 1H, ArH), 8.02 (d, J = 7.2 Hz, 1H, ArH), 7.54 (d, J = 7.6 Hz, 2H, ArH), 7.46~7.38 (m, 2H, ArH), 7.36~7.32 (m, 1H, ArH), 7.18~7.12 (m, 4H, ArH), 7.07~7.05 (m, 2H, ArH), 2.07 (s, 3H, CH_3); ^{13}C NMR (150 MHz, DMSO-d_6) δ: 184.8, 138.3, 136.9, 134.9, 134.1, 132.6, 131.8, 131.7, 131.5, 130.0, 129.5, 127.6, 127.4, 125.8, 124.7, 124.4, 124.1, 116.0, 109.0, 9.9; IR (KBr) υ: 3062, 1747, 1462, 1367, 1155, 1076, 966, 882 cm^{-1}; MS (m/z): HRMS (ESI) Calcd. for $C_{24}H_{16}ClNNaOS$ ([M+Na]$^{+}$): 424.0533. Found: 424.0529.

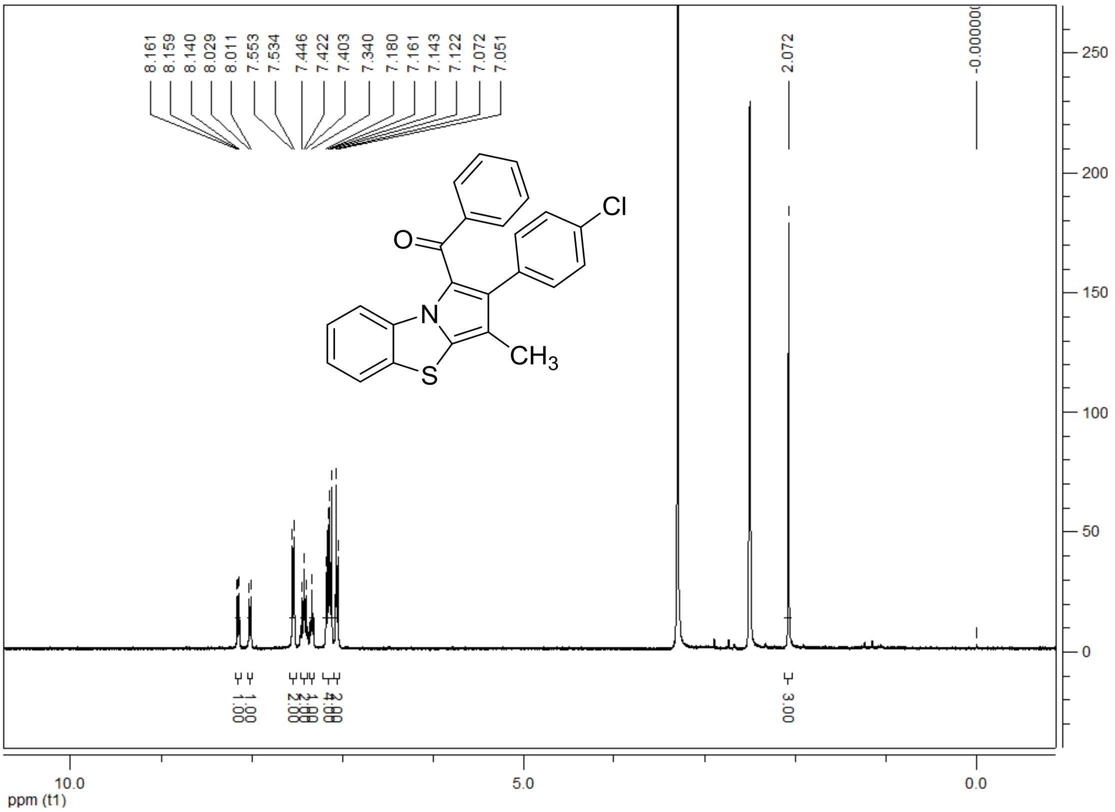

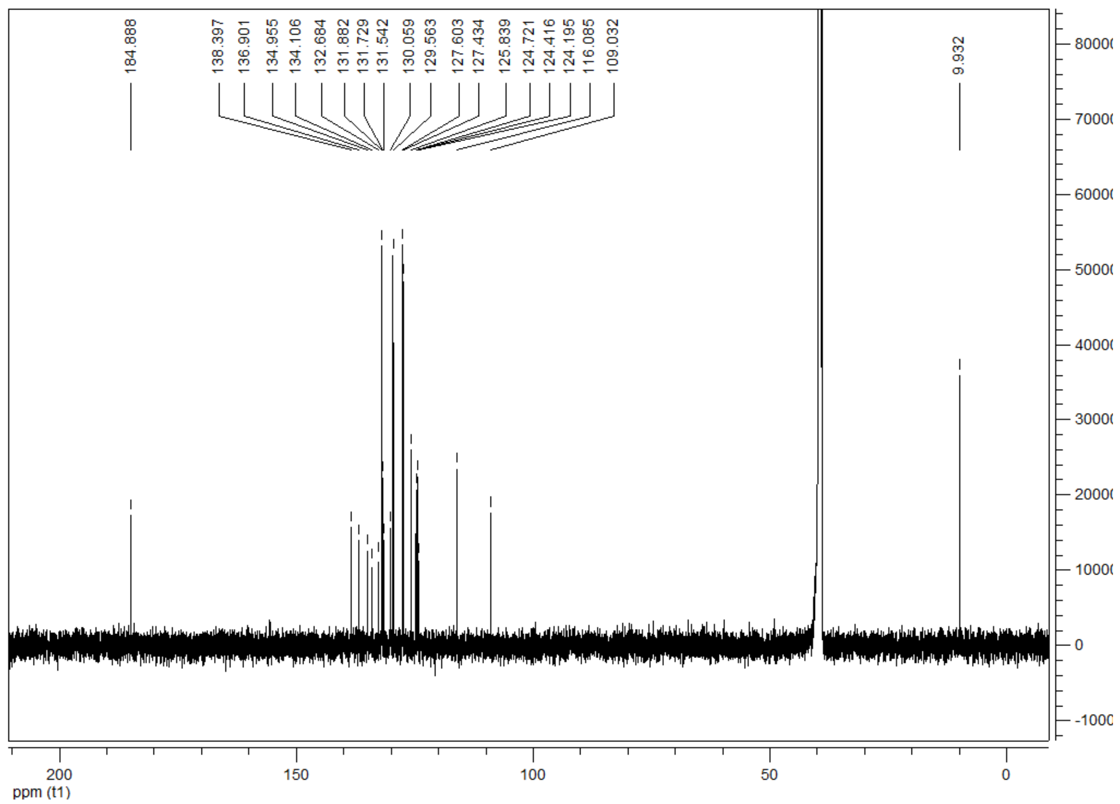

184.888
138.397
136.901
134.955
134.106
132.684
131.882
131.729
131.542
130.059
129.563
127.603
127.434
125.839
124.721
124.416
124.195
116.085
109.032
9.932
ppm (t1)

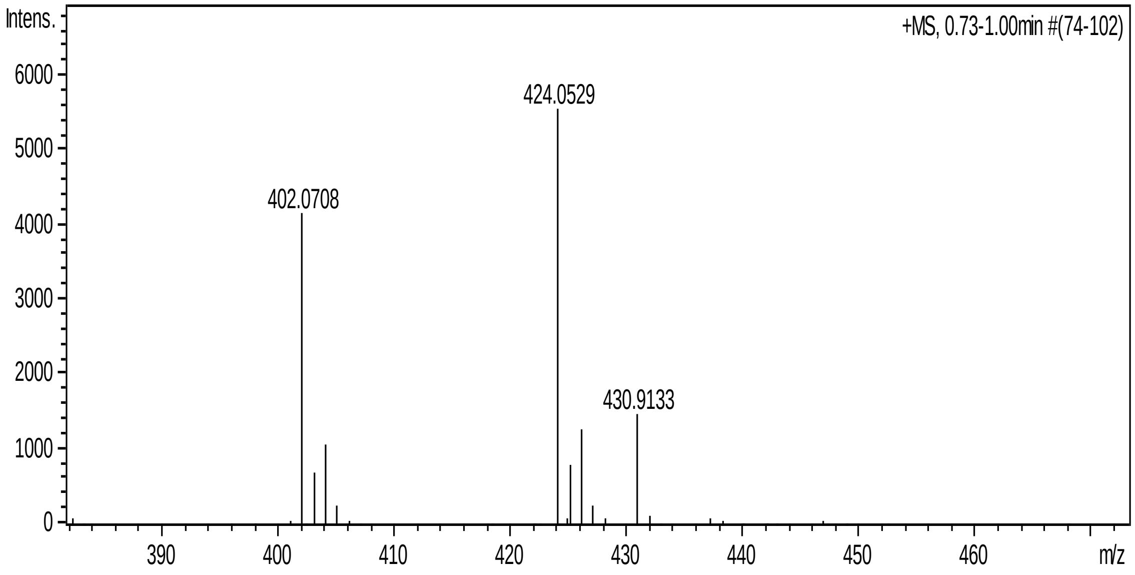

Intens.
+MS, 0.73-1.00min #(74-102)
424.0529
402.0708
430.9133
m/z

(3-methyl-2-phenylbenzo[d]pyrrolo[2,1-b]thiazol-1-yl)(p-tolyl)methanone (4b):

yellow solid, 89%, m.p. 132~134℃; ^{1}H NMR (400 MHz, DMSO-d_6) δ: 8.00 (brs, 2H, ArH), 7.47 (d, *J* = 8.0 Hz, 2H, ArH), 7.43~7.35 (m, 2H, ArH), 7.11~7.07 (m, 5H, ArH), 6.94 (d, *J* = 7.6 Hz, 2H, ArH), 2.18 (s, 3H, CH_3), 2.08 (s, 3H, CH_3); ^{13}C NMR (100 MHz, DMSO-d_6) δ: 233.7, 184.9, 141.9, 137.4, 135.6, 134.8, 133.7, 133.0, 130.0, 129.6, 128.0, 127.4, 126.5, 125.7, 124.4, 124.3, 123.9, 115.6, 108.6, 20.8, 10.0; IR (KBr) υ: 3097, 2965, 1749, 1453, 1372, 1284, 1203, 1072, 866 cm^{-1}; MS (*m/z*): HRMS (ESI) Calcd. for $C_{25}H_{19}NNaOS$ ([M+Na]$^+$): 404.1080. Found: 404.1078.

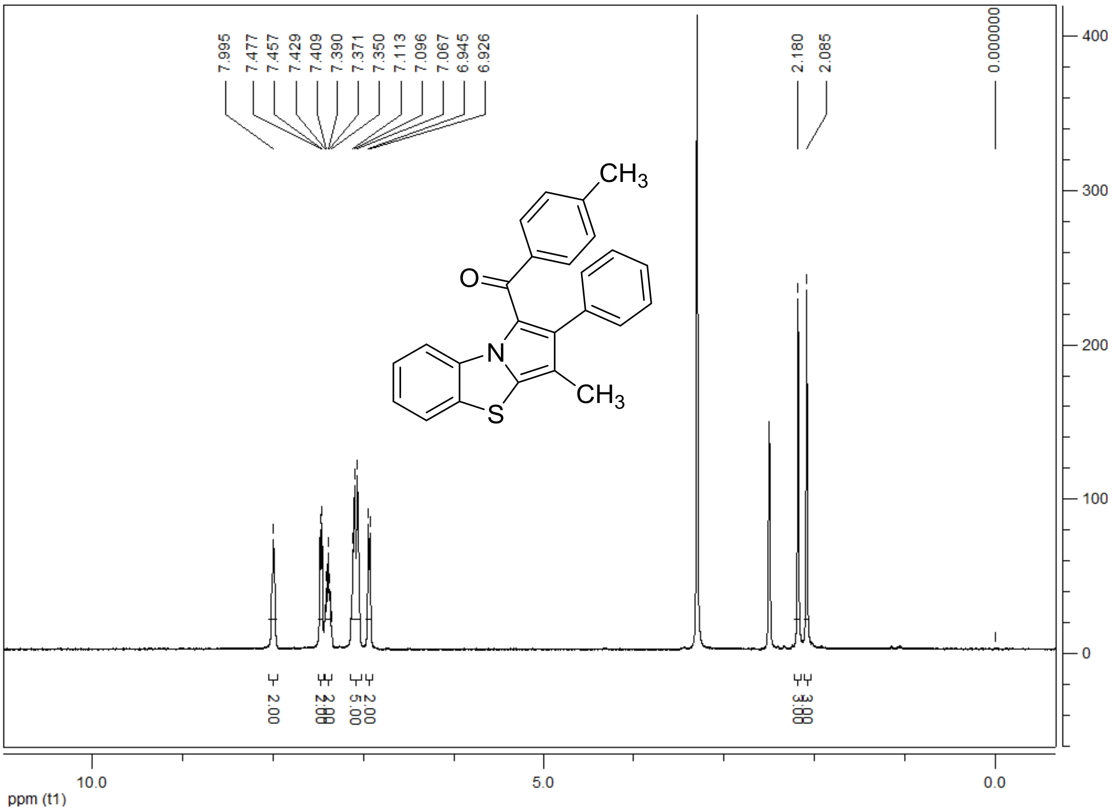

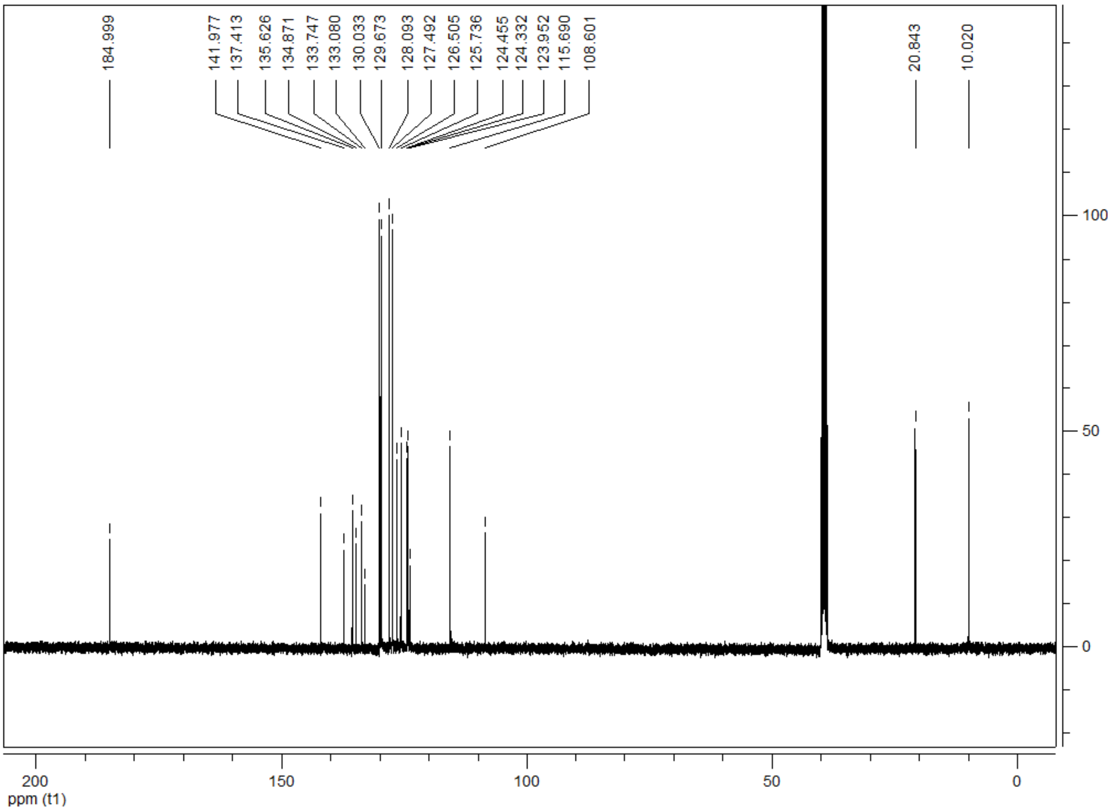
184.999
141.977
137.413
135.626
134.871
133.747
133.080
130.033
129.673
128.093
127.492
126.505
125.736
124.455
124.332
123.952
115.690
108.601
20.843
10.020
100
50
0
200
150
100
50
0
ppm (t1)

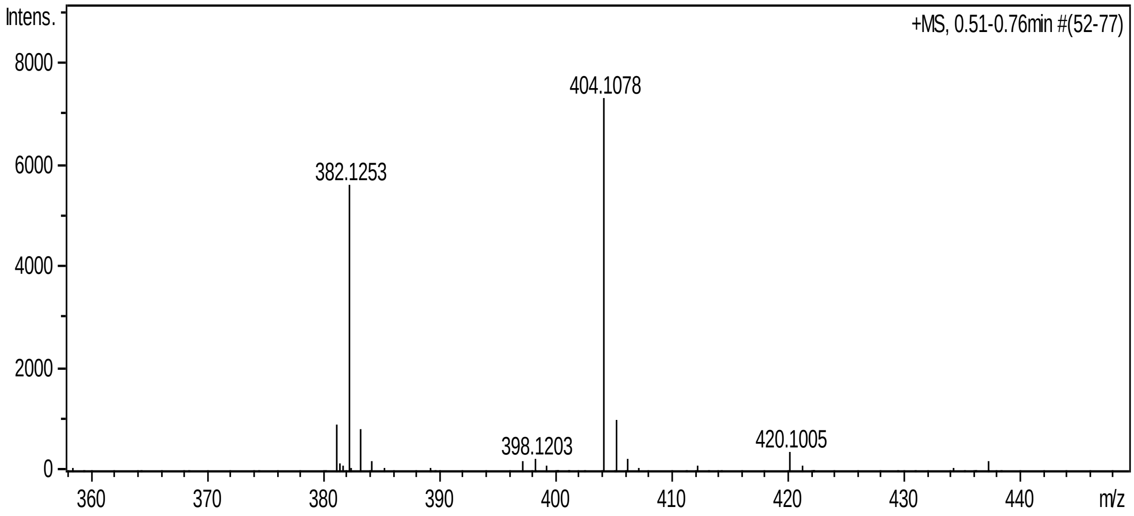
Intens.
+MS, 0.51-0.76min #(52-77)
8000
6000
4000
2000
0
404.1078
382.1253
398.1203
420.1005
360
370
380
390
400
410
420
430
440
m/z

(4-methoxyphenyl)(3-methyl-2-phenylbenzo[d]pyrrolo[2,1-b]thiazol-1-yl)methanone (4c): yellow solid, 85%, m.p. 103~105℃; ^{1}H NMR (400 MHz, DMSO-d_6) δ: 7.99~7.94 (m, 2H, ArH), 7.57~7.56 (m, 2H, ArH), 7.40~7.36 (m, 2H, ArH), 7.14~7.08 (m, 5H, ArH), 6.68~6.66 (m, 2H, ArH), 3.68 (s, 3H, OCH_3), 2.09 (s, 3H, CH_3); ^{13}C NMR (100 MHz, DMSO-d_6) δ: 184.1, 162.1, 136.8, 134.7, 133.8, 132.5, 131.8, 130.7, 130.0, 130.0, 127.5, 126.5, 125.7, 124.3, 124.3, 123.7, 115.5, 112.9, 108.3, 55.2, 10.0; IR (KBr) υ: 3000, 1754, 1557, 1461, 1363, 1253, 1079, 960, 750 cm^{-1}; MS (*m/z*): HRMS (ESI) Calcd. for $C_{25}H_{19}NNaO_2S$ ($[M+Na]^+$): 420.1029. Found: 420.1026.

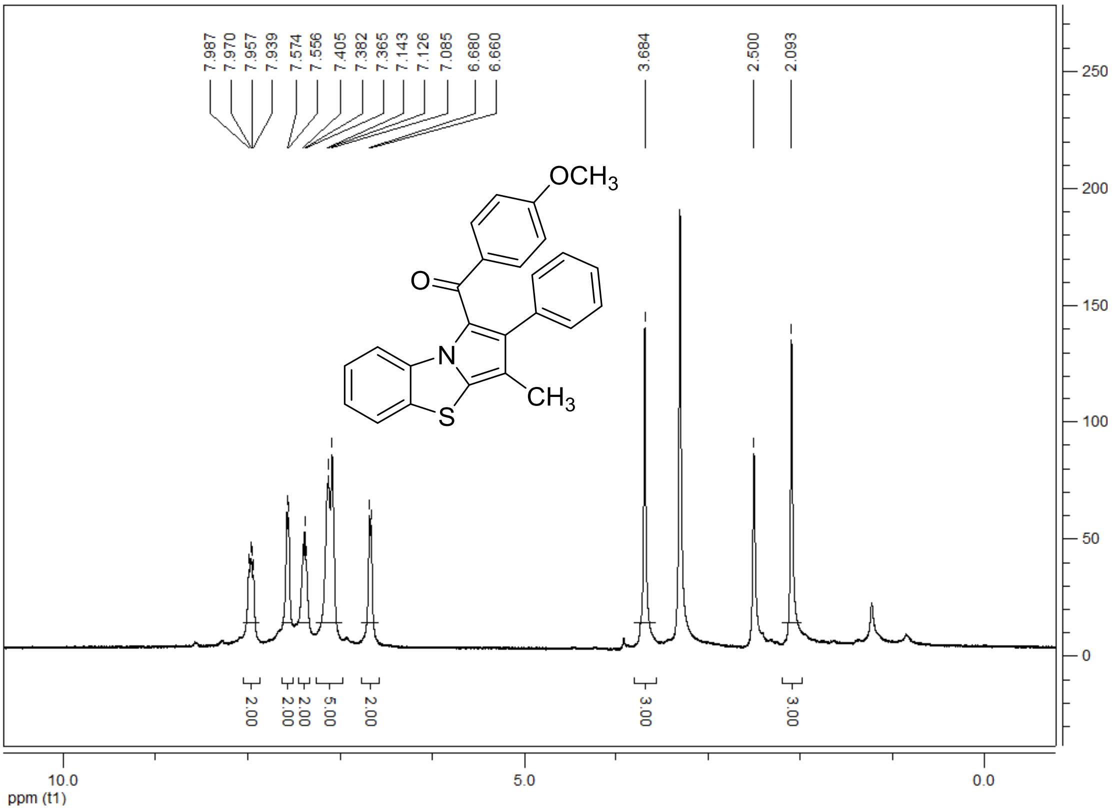

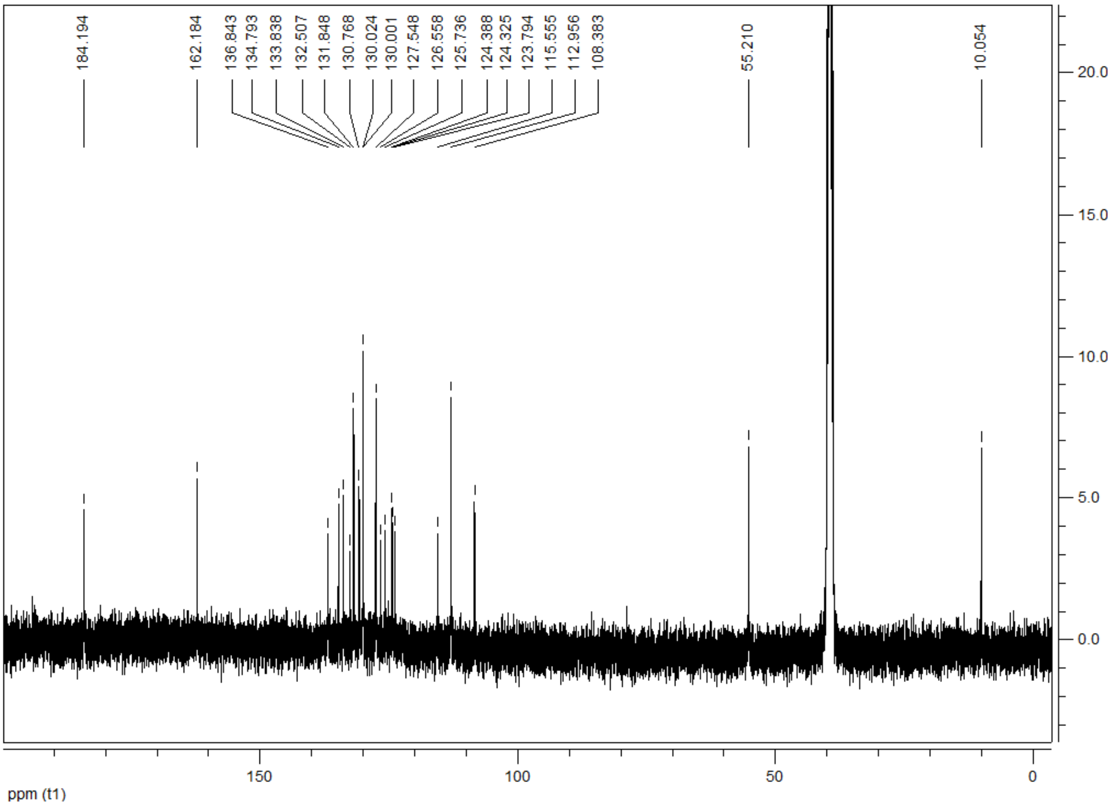
184.194
162.184
136.843
134.793
133.838
132.507
131.848
130.768
130.024
130.001
127.548
126.558
125.736
124.388
124.325
123.794
115.555
112.956
108.383
55.210
10.054
20.0
15.0
10.0
5.0
0.0
150
100
50
0
ppm (t1)

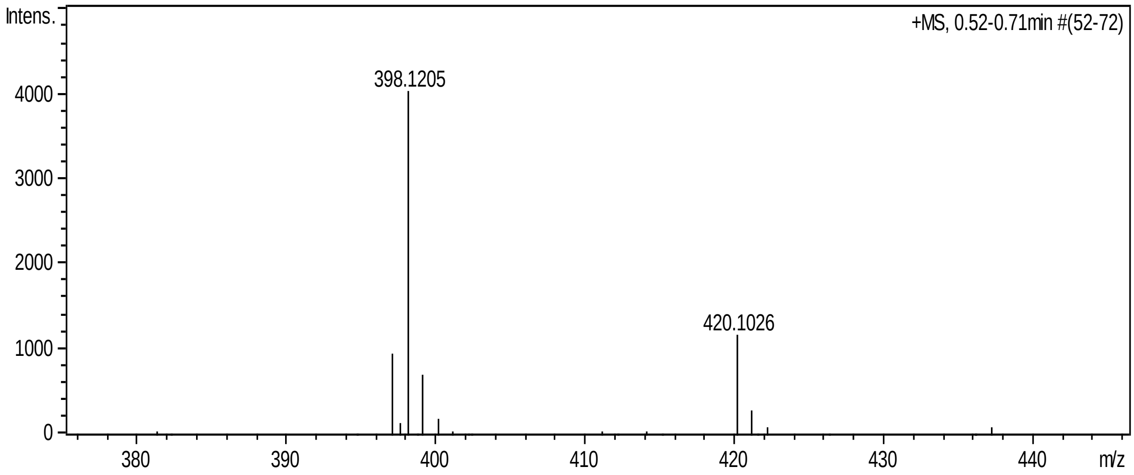
Intens.
+MS, 0.52-0.71min #(52-72)
398.1205
420.1026
4000
3000
2000
1000
0
380
390
400
410
420
430
440
m/z

(4-methoxyphenyl)(3-methyl-2-(p-tolyl)benzo[d]pyrrolo[2,1-b]thiazol-1-yl)methanone (4d): yellow solid, 87%, m.p. 128~130℃; ^{1}H NMR (400 MHz, DMSO-d_6) δ: 7.98~7.89 (m, 2H, ArH), 7.57~7.56 (m, 2H, ArH), 7.39~7.35 (m, 2H, ArH), 6.96 (s, 4H, ArH), 6.70~6.68 (m, 2H, ArH), 3.70 (s, 3H, OCH_3), 2.18 (s, 3H, CH_3) , 2.08 (s, 3H, CH_3); ^{13}C NMR (150 MHz, DMSO-d_6) δ: 184.4, 162.3, 136.8, 135.7, 134.9, 132.4, 131.9, 130.9, 130.8, 130.0, 130.0, 128.2, 125.8, 124.4, 123.8, 115.5, 113.1, 108.4, 55.3, 20.5, 10.1; IR (KBr) υ: 3001, 1751, 1583, 1521, 1461, 1369, 1301, 1252, 1159, 960, 827, 747 cm^{-1}; MS (m/z): HRMS (ESI) Calcd. for $C_{26}H_{22}NO_2S$ ($[M+H]^+$): 412.1366. Found: 412.1366.

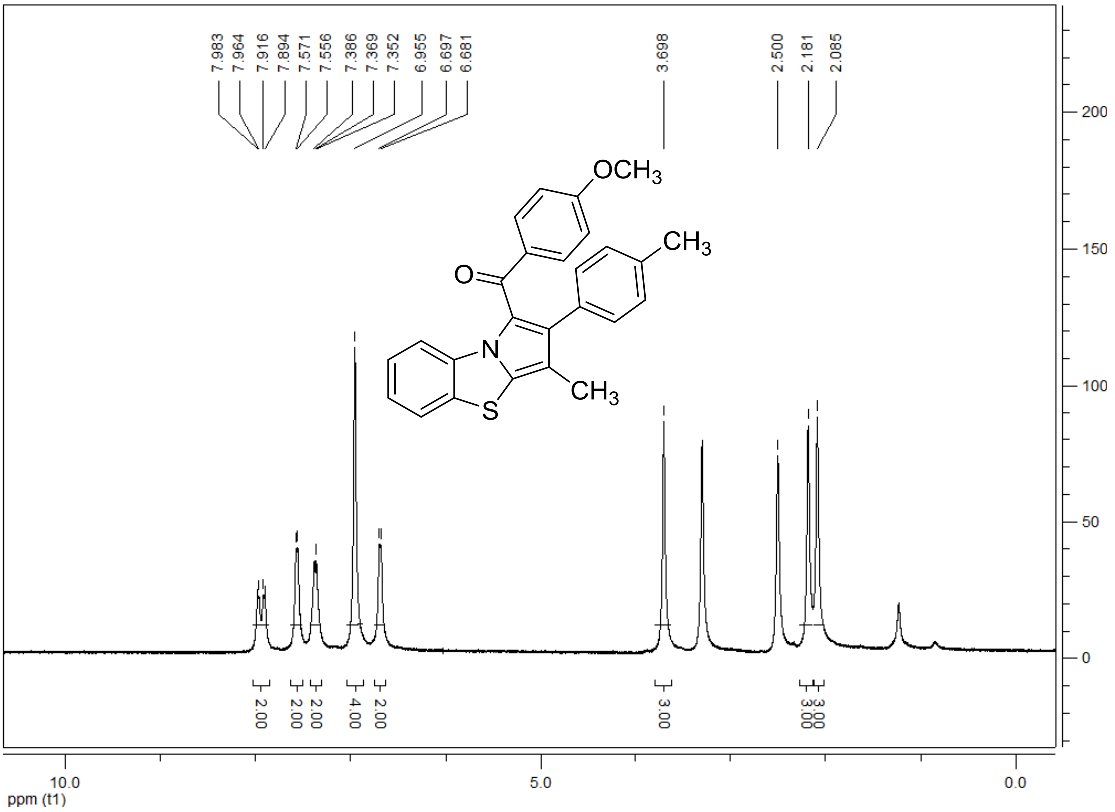

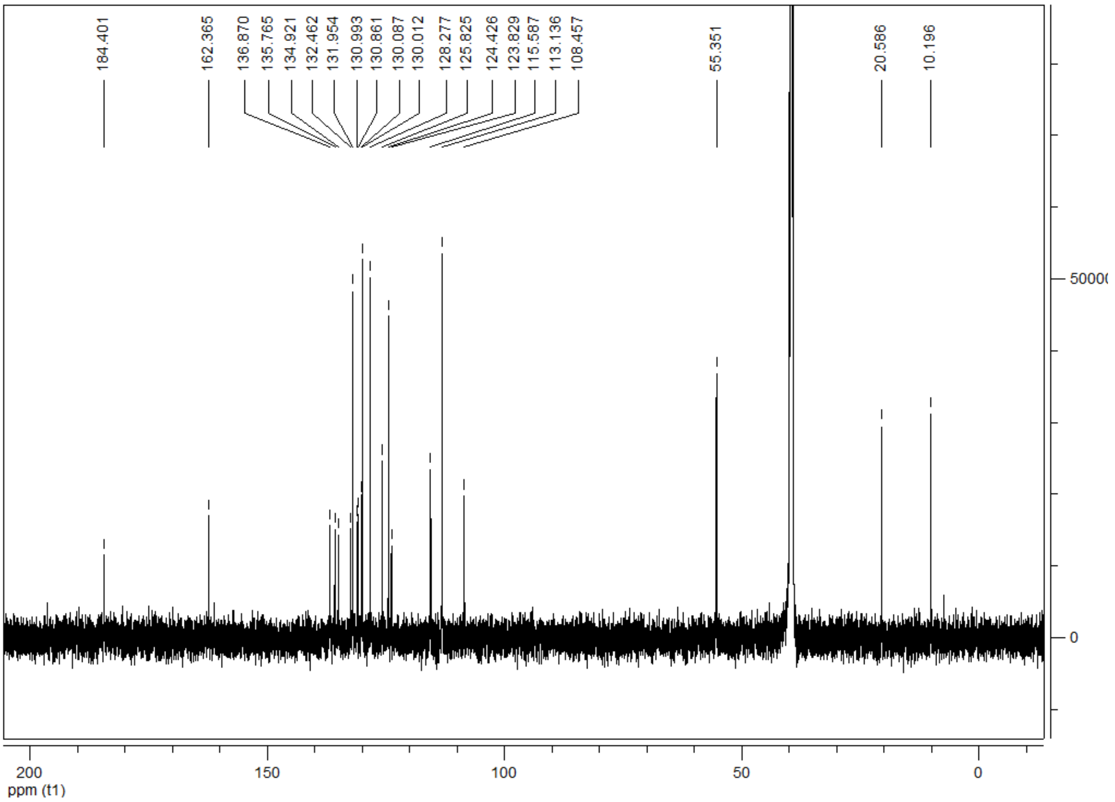
184.401
162.365
136.870
135.765
134.921
132.462
131.954
130.993
130.861
130.087
130.012
128.277
125.825
124.426
123.829
115.587
113.136
108.457
55.351
20.586
10.196
50000
0
200
150
100
50
0
ppm (t1)

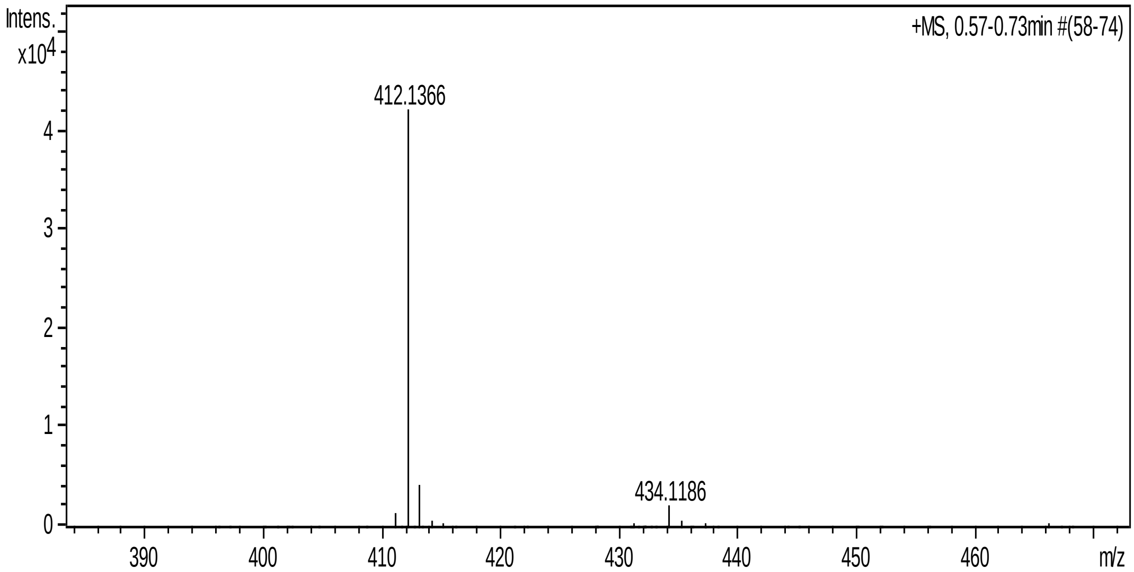
Intens.
x10^4
+MS, 0.57-0.73min #(58-74)
412.1366
434.1186
4
3
2
1
0
390
400
410
420
430
440
450
460
m/z

(2-(4-chlorophenyl)-3-methylbenzo[d]pyrrolo[2,1-b]thiazol-1-yl)(4-methoxyphenyl)methanone (4e): yellow solid, 85%, m.p. 166~168℃; ^{1}H NMR (400 MHz, DMSO-d_6) δ: 7.98 (brs, 2H, ArH), 7.54 (s, 2H, ArH), 7.38 (brs, 2H, ArH), 7.18~7.08 (m, 4H, ArH), 6.71 (s, 2H, ArH), 3.71 (s, 3H, OCH_3), 2.08 (s, 3H, CH_3); ^{13}C NMR (150 MHz, DMSO-d_6) δ: 184.0, 162.4, 135.7, 134.8, 132.9, 131.9, 131.8, 131.5, 130.8, 130.1, 127.6, 125.8, 124.6, 124.4, 124.0, 115.8, 113.1, 108.5, 55.3, 10.0; IR (KBr) υ: 3065, 1743, 1588, 1508, 1458, 1368, 1303, 1250, 1156, 1078, 966, 837, 750 cm^{-1}; MS (*m/z*): HRMS (ESI) Calcd. for $C_{25}H_{18}ClNNaO_2S$ ($[M+Na]^+$): 454.0639. Found: 454.0635.

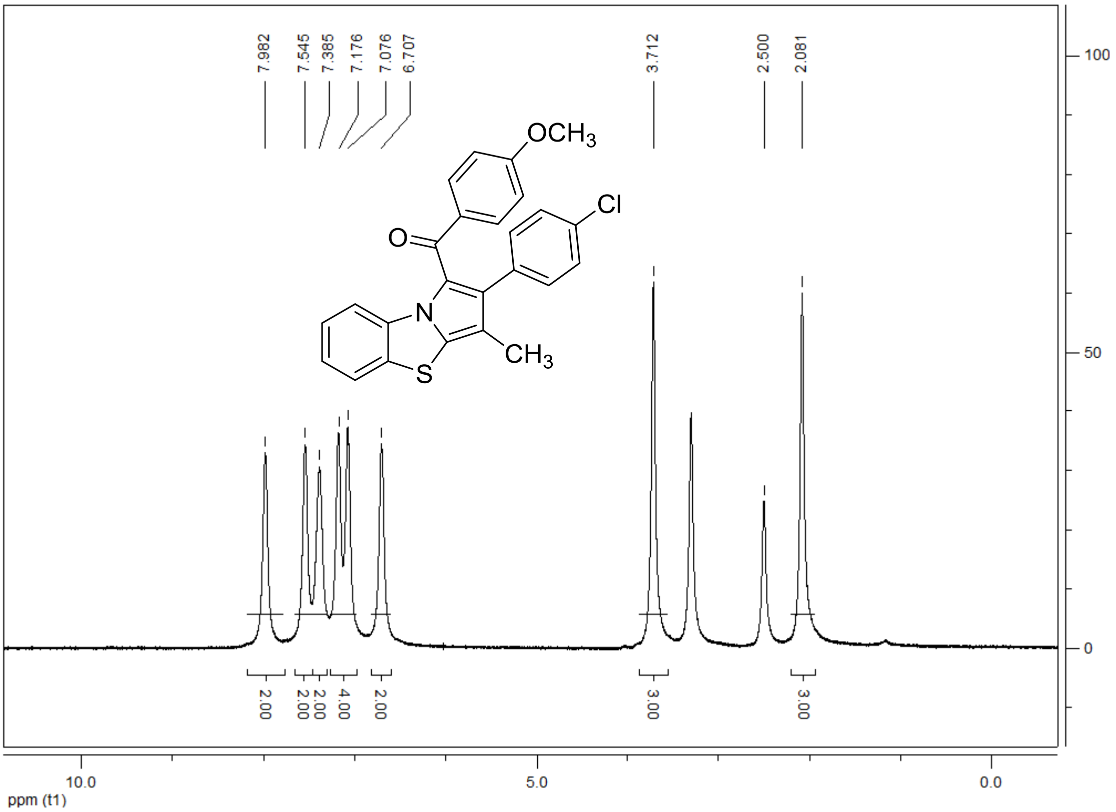

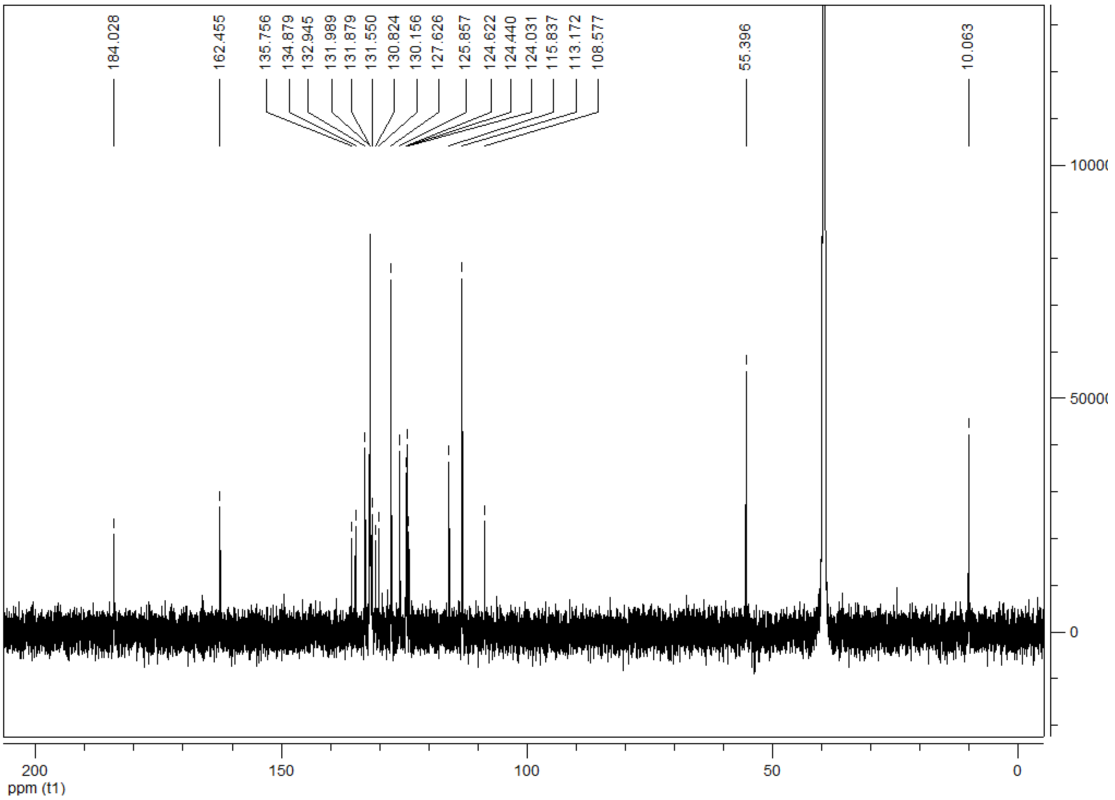
184.028
162.455
135.756
134.879
132.945
131.989
131.879
131.550
130.824
130.156
127.626
125.857
124.622
124.440
124.031
115.837
113.172
108.577
55.396
10.063
200
150
100
50
0
ppm (t1)

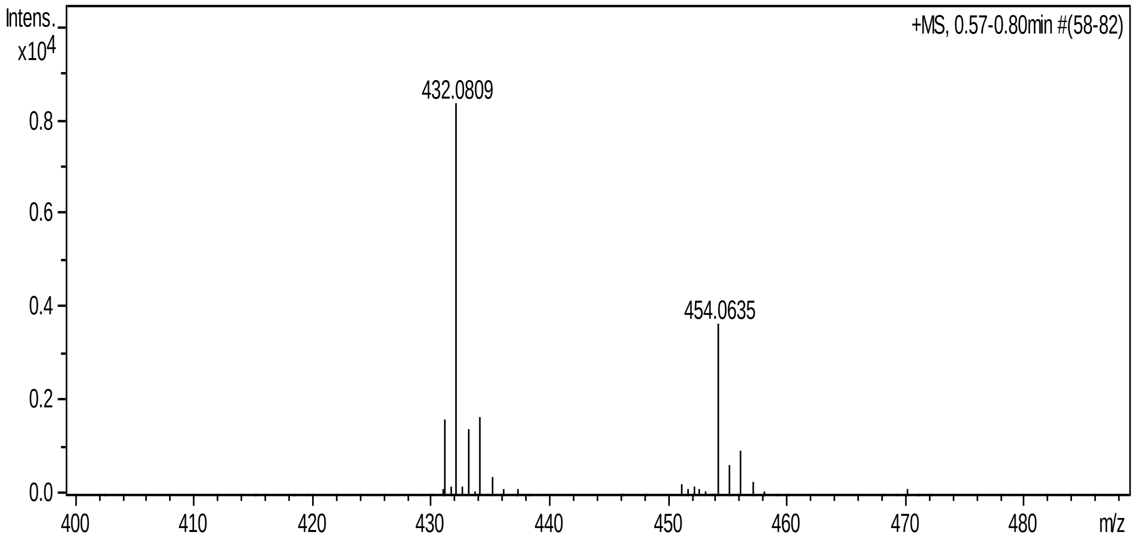
Intens.
x10⁴
+MS, 0.57-0.80min #(58-82)
432.0809
454.0635
0.8
0.6
0.4
0.2
0.0
400
410
420
430
440
450
460
470
480
m/z

(4-chlorophenyl)(2-(4-chlorophenyl)-3-methylbenzo[d]pyrrolo[2,1-b]thiazol-1-yl)methanone (4f): yellow solid, 90%, m.p. 157~159℃; ^{1}H NMR (400 MHz, DMSO-*d*$_6$) δ: 8.23 (d, *J* = 8.0 Hz, 1H, ArH), 8.02 (d, *J* = 8.0 Hz, 1H, ArH), 7.52~7.39 (m, 4H, ArH), 7.20~7.16 (m, 4H, ArH), 7.05~7.03 (m, 2H, ArH), 2.06 (s, 3H, CH_3); ^{13}C NMR (150 MHz, DMSO-*d*$_6$) δ: 183.2, 137.3, 137.2, 136.4, 134.9, 134.8, 132.5, 131.9, 131.7, 131.2, 130.0, 127.5, 127.4, 125.8, 124.7, 124.3, 124.0, 116.2, 109.2, 9.8; IR (KBr) υ: 3054, 1750, 1575, 1507, 1461, 1370, 1293, 1208, 1166, 1086, 1005, 960, 915, 833, 740 cm^{-1}; MS (*m*/*z*): HRMS (ESI) Calcd. for $C_{24}H_{15}Cl_2NNaOS$ ([M+Na]$^+$): 458.0144. Found: 458.0142.

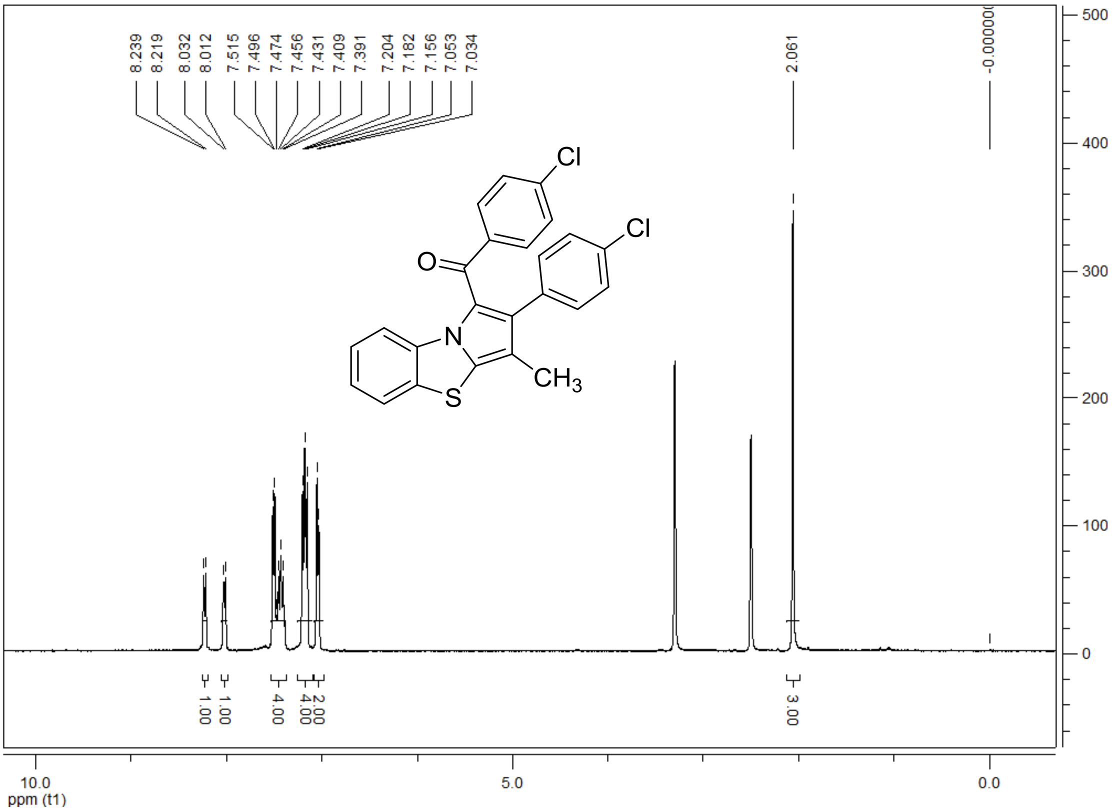

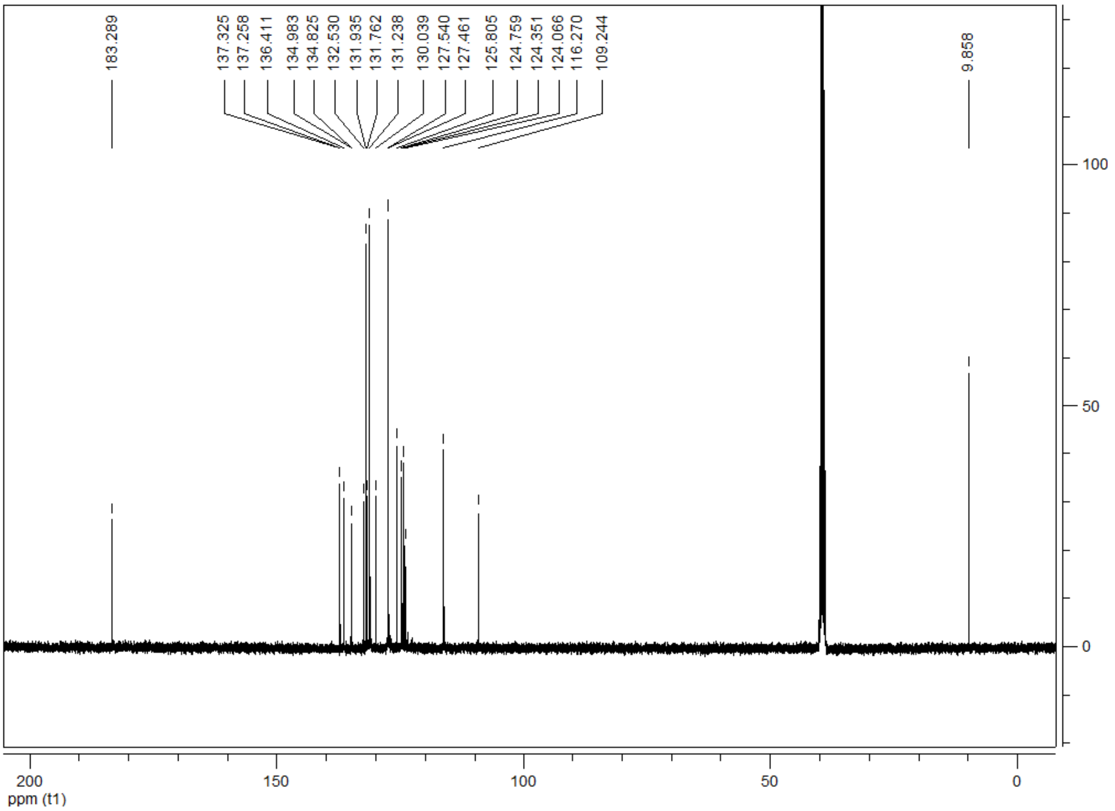
183.289
137.325
137.258
136.411
134.983
134.825
132.530
131.935
131.762
131.238
130.039
127.540
127.461
125.805
124.759
124.351
124.066
116.270
109.244
9.858
100
50
0
200
150
100
50
0
ppm (t1)

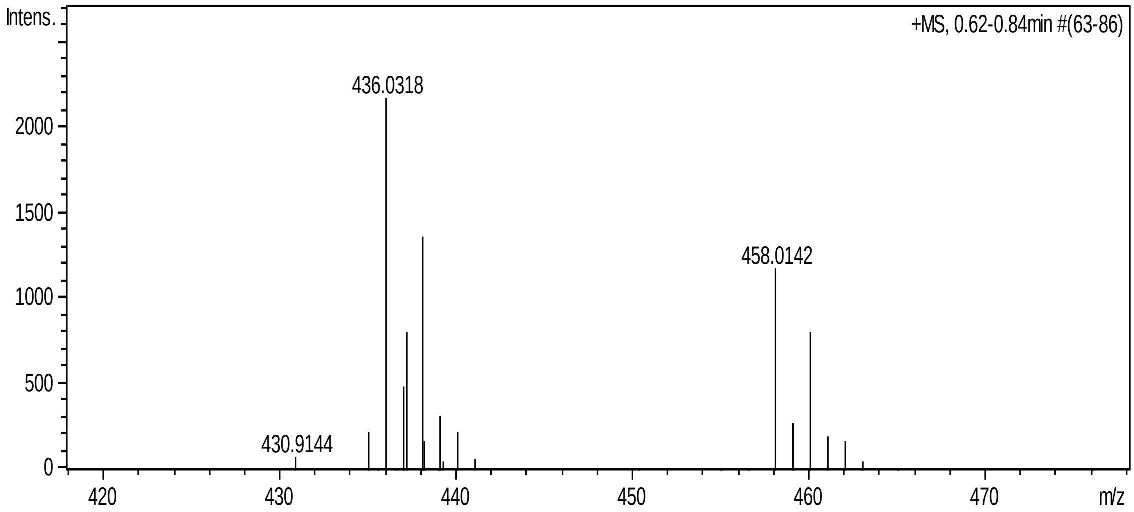
Intens.
+MS, 0.62-0.84min #(63-86)
436.0318
458.0142
430.9144
2000
1500
1000
500
0
420
430
440
450
460
470
m/z
